# The evolutionary path of the epithelial sodium channel δ-subunit in Cetartiodactyla points to a role in sodium sensing

**DOI:** 10.1038/s42003-025-08436-7

**Published:** 2025-07-04

**Authors:** Fynn Zahnow, Chiara Jäger, Yassmin Mohamed, Gianluca Vogelhuber, Fabian May, Alexandra Maria Ciocan, Arianna Manieri, Stephan Maxeiner, Gabriela Krasteva-Christ, Oskar Schnappauf, Matthew R. D. Cobain, Lars Podsiadlowski, José Luis Crespo-Picazo, Daniel García-Párraga, Mike Althaus

**Affiliations:** 1 Institute for Functional Gene Analytics, Bonn-Rhein-Sieg University of Applied Sciences, Rheinbach, Germany; 2 Institute of Anatomy and Cell Biology, Saarland University, Homburg, Germany; 3 Center for Gender-specific Biology and Medicine (CGBM), Saarland University, Homburg, Germany; 4 Department of Biological and Environmental Science, University of Jyväskylä, Jyväskylä, Finland; 5 Leibniz Institute for the Analysis of Biodiversity Change (LIB), Bonn, Germany; 6 Research Department, Fundación Oceanogràfic de la Comunitat Valenciana, Valencia, Spain

**Keywords:** Molecular evolution, Kidney

## Abstract

The epithelial sodium channel (ENaC) is essential for osmoregulation in tetrapod vertebrates. There are four ENaC-subunits (α, β, γ, δ) which form αβγ- or δβγ-ENaCs. While αβγ-ENaC is a ‘maintenance protein’ controlling sodium homeostasis, δβγ-ENaC might represent a ‘stress protein’ monitoring high sodium concentrations. The δ-subunit emerged with water-to-land transition of vertebrates. We examined ENaC evolution in Cetartiodactyla, a group including even-toed ungulates and cetaceans (whales, dolphins and porpoises) which returned to marine environments in the Eocene. Genes for α-, β-, and γ-ENaC are intact across Cetartiodactyla. While *SCNN1D* (δ-ENaC) is intact in terrestrial Artiodactyla, it is a pseudogene in cetaceans. A unique fusion of *SCNN1D* exons 11 and 12 is observed in the Antilopinae. Transcripts of α-, β-, and γ-ENaC are present in kidney, lung and skin tissues of Bottlenose dolphins, underscoring αβγ-ENaC’s maintenance role. Bottlenose dolphins and Beluga whales do not show behavioural differences between sodium-containing and sodium-free stimuli, supporting a function of δ-ENaC as a sodium sensing protein which might have become obsolete in high-salinity marine environments. Consistently, there is reduced selection pressure or pseudogenisation of *SCNN1D* in other marine mammals. Erosion of *SCNN1D* might therefore be a consequence of environmental transition in marine mammals.

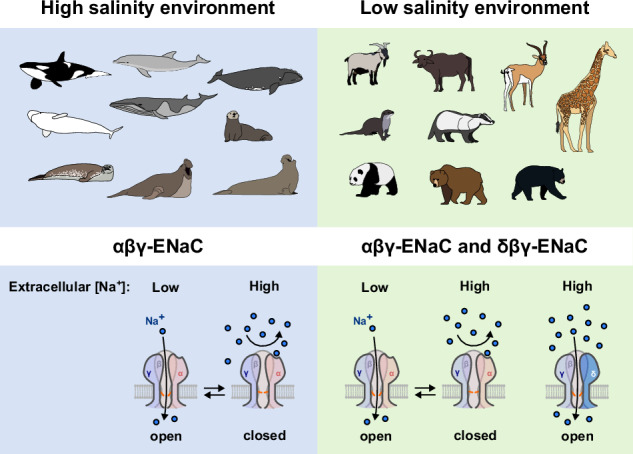

## Abstract (web summary)

Zahnow et al. investigate the evolution of epithelial sodium channels (ENaCs) in mammals returning to marine life. ENaCs are built of αβγ- or δβγ-subunits. Their study suggests the *SCNN1D* gene encoding the δ-subunit suffers pseudogenization events in marine mammals due to high-salinity environment.

## Introduction

Transitions between terrestrial and aquatic environments were key events in mammalian evolution. Extant cetaceans (whales, dolphins and porpoises) evolved from terrestrial artiodactyls (even-toed ungulates), which transitioned into the oceans in the Eocene^1^. The loss or pseudogenisation of protein-coding genes is a frequent genomic change observed in cetaceans^2^. For example, gene loss has been described for hair- and epidermis-specific genes^2^, enamel-specific genes^3^, genes involved in the control of haemoglobin oxygen affinity^2^, lipid metabolism and/or oxidative stress response^4^ or taste receptor genes^5,6^. While some genes may simply become less relevant when species transition into new environments, gene loss or pseudogenisation has also been suggested to trigger broader phenotypic changes^7^, which may provide adaptive innovations that allow cetaceans to thrive in marine environments.

The transition of cetacean ancestors to marine habitats was accompanied by major dietary changes from herbivory/omnivory to carnivory^1^ as well as the evolution of mechanisms to maintain electrolyte homoeostasis in a hypersaline marine environment^8^. While terrestrial herbivorous artiodactyls are challenged with maintaining stable sodium concentrations in their extracellular fluids in a sodium-scarce environment, marine cetaceans are faced with environmental sodium concentrations >3 times greater than their plasma sodium levels^9^ and little to no access to freshwater.

A key protein in the control of sodium homoeostasis in mammals is the epithelial sodium channel (ENaC)^10^. Canonical ENaC is a heterotrimeric sodium-selective ion channel which is composed of three subunits encoded by the genes *SCNN1A* (α-ENaC subunit), *SCNN1B* (β-ENaC subunit) and *SCNN1G* (γ-ENaC subunit). αβγ-ENaC is located in the apical membranes of epithelial cells in the distal nephrons of the kidneys, lungs and colon^10^. In the distal nephron, aldosterone enhances ENaC expression and thereby matches dietary sodium intake with its excretion rate. In rodents, αβγ-ENaC is also expressed in magnocellular neurons of the paraventricular nucleus and supraoptic nucleus of the hypothalamus and might serve as a sodium sensor in the control of vasopressin release^11^. Consistent with sensory functions of this ion channel, αβγ-ENaC has been shown to mediate appetitive sodium taste in mice^12,13^.

An additional *SCNN1D* gene, encoding the δ-ENaC subunit, emerged with the water-to-land transition in tetrapod vertebrates^14,15^. This subunit can assemble with β- and γ-ENaC, yielding δβγ-ENaCs with distinct biophysical properties and mechanisms regulating their function. δβγ-ENaCs consistently generate larger transmembrane ion currents in heterologous expression systems than αβγ-ENaCs^16–18^. ENaC activity is rapidly controlled by an auto-regulatory mechanism termed sodium self-inhibition (SSI). SSI is triggered by binding of sodium ions to a cation binding site formed by amino acid residues in the extracellular β6-β7-loop of the α-ENaC or δ-ENaC subunits^19–21^, which subsequently reduces channel open probability. It is hypothesised that SSI rapidly adjusts ENaC open probability to fluctuating extracellular sodium concentrations, particularly in the distal nephron, where this mechanism might prevent excess sodium absorption when urinary sodium concentrations are high^22^. Furthermore, SSI renders ENaC isoforms sensitive to the extracellular pH since protons antagonise SSI^20^. However, SSI of δβγ-ENaC is species-dependent, with mammalian δβγ-ENaCs showing a markedly reduced SSI due to lack of key structural motifs forming the sodium binding site in the extracellular domain of the δ-ENaC subunit^17,20^. Due to SSI, activity of mammalian αβγ-ENaC does not increase under extracellular sodium concentrations higher than plasma sodium concentrations, whereas mammalian δβγ-ENaC operates over broader sodium concentration ranges in heterologous expression systems^17^. Consistently, αβγ-ENaC mediates attractive taste to low sodium concentrations in mice^12,13^. In humans, it has been hypothesised that δβγ-ENaC in taste buds could sense high sodium concentrations that exceed plasma levels^23^. While αβγ-ENaC is a key ‘maintenance protein’ controlling overall body sodium homoeostasis, δβγ-ENaC likely represents a ‘stress protein’ monitoring high sodium concentrations in tetrapod vertebrates. We, therefore, questioned how ENaC isoforms might operate in hypersaline marine environments and investigated the evolutionary path of ENaC subunit coding genes in cetaceans and terrestrial Artiodactyla as well as other marine mammals. We show that genes for α-, β-, and γ-ENaC are intact across all investigated species, but there is reduced selection pressure or pseudogenisation of *SCNN1D* in marine mammals. Erosion of *SCNN1D* might therefore be a consequence of environmental transition in marine mammals.

## Results and discussion

### Distribution of functional ENaC-coding genes in Cetartiodactyla

Using genomic sequences available in the National Center for Biotechnology Information (NCBI) database, we analysed all four *SCNN1* genes in 45 representative species of 22 cetartiodactylan families. To facilitate inter-species comparisons, exon nomenclature was followed as previously suggested^17^, with exons 2 and 13 encoding the first and second transmembrane domain of each ENaC subunit, respectively. In most species, exon 2 contains the translation start codon (with the exception of some *SCNN1D* genes in terrestrial Artiodactyla, where the start codon is located on an upstream exon 1, Supplementary Data 1^24^,), while the STOP codon is located on exon 13. Thus, exons 2-13 encode the highly conserved core region of each ENaC subunit. Additional exons upstream of exon 2 are more variable and might be subject to alternative splicing. In this study, we, therefore, concentrated on the core region of each ENaC subunit and analysed the overall gene structure, exon sizes, splice donor/acceptor sites and continuous open reading frames (ORFs), in comparison with the corresponding sequences of the Alpaca (*Vicugna pacos*), a terrestrial species closely related to cetaceans, as a reference. Because multiple NCBI mRNA sequences were predicted by algorithms and contain corrections (including removal of insertions/deletions and STOP codons), we constructed all sequences using genomic DNA of each respective species as template^24^. We confirmed the constructed coding sequences using transcriptomic data available in the NCBI Sequence Read Archive (Supplementary Table 1).

**Fig. 1:**
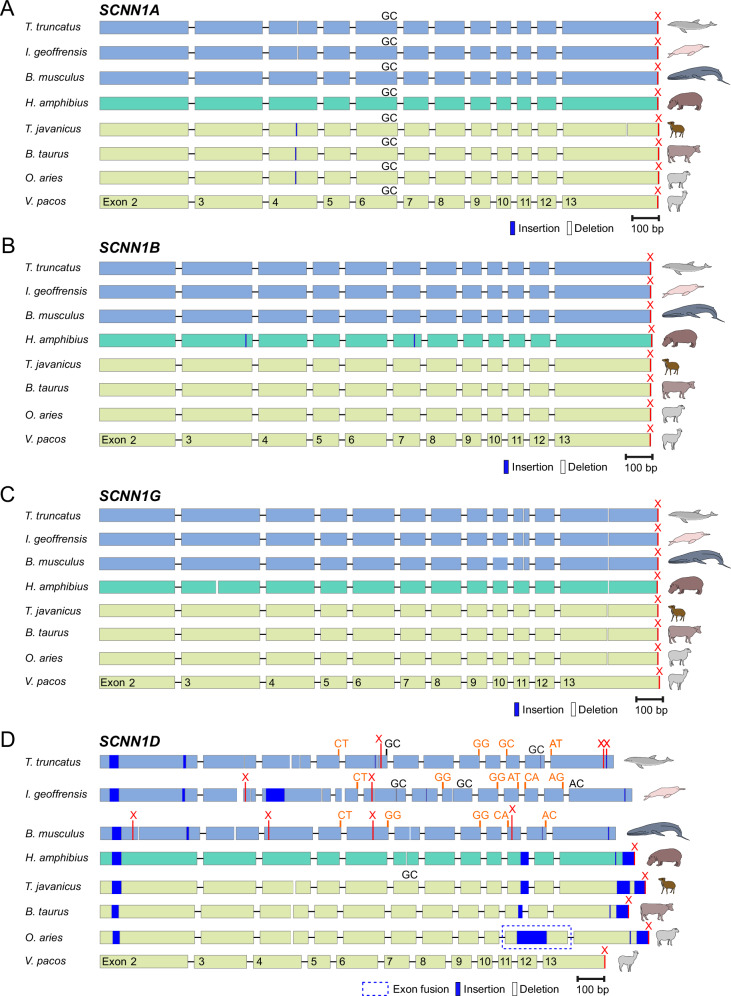
Structure of *SCNN1* genes in select Cetartiodactyla. **A**–**D** The genes *SCNN1A*, *SCNN1B*, *SCNN1G* and *SCNN1D*, respectively. Exons 2–13 are numbered according to the nomenclature by Gettings et al.^17^ with exons 2 and 13 encoding the first and second transmembrane domain of each ENaC subunit, respectively. Only exons are drawn to scale. Exon sizes are outlined with respect to the Alpaca (*Vicugna pacos*) as a reference and insertions and deletions are highlighted. The size of exon 13 is shown up to the corresponding STOP codon in *V. pacos*. Non-canonical splice donor/acceptor motifs (AC/GC) flanking the exons are indicated in black font, whereas mutated splice sites are indicated in orange. Stop codons are marked with a red X. The dashed box indicates a fusion of exons 11 and 12 of the *SCNN1D* gene due to mutation of the splice donor following exon 11 in *Ovis aries*. Exon colour code: light blue = marine species; turquoise = amphibious species; green = terrestrial species.

**Fig. 2:**
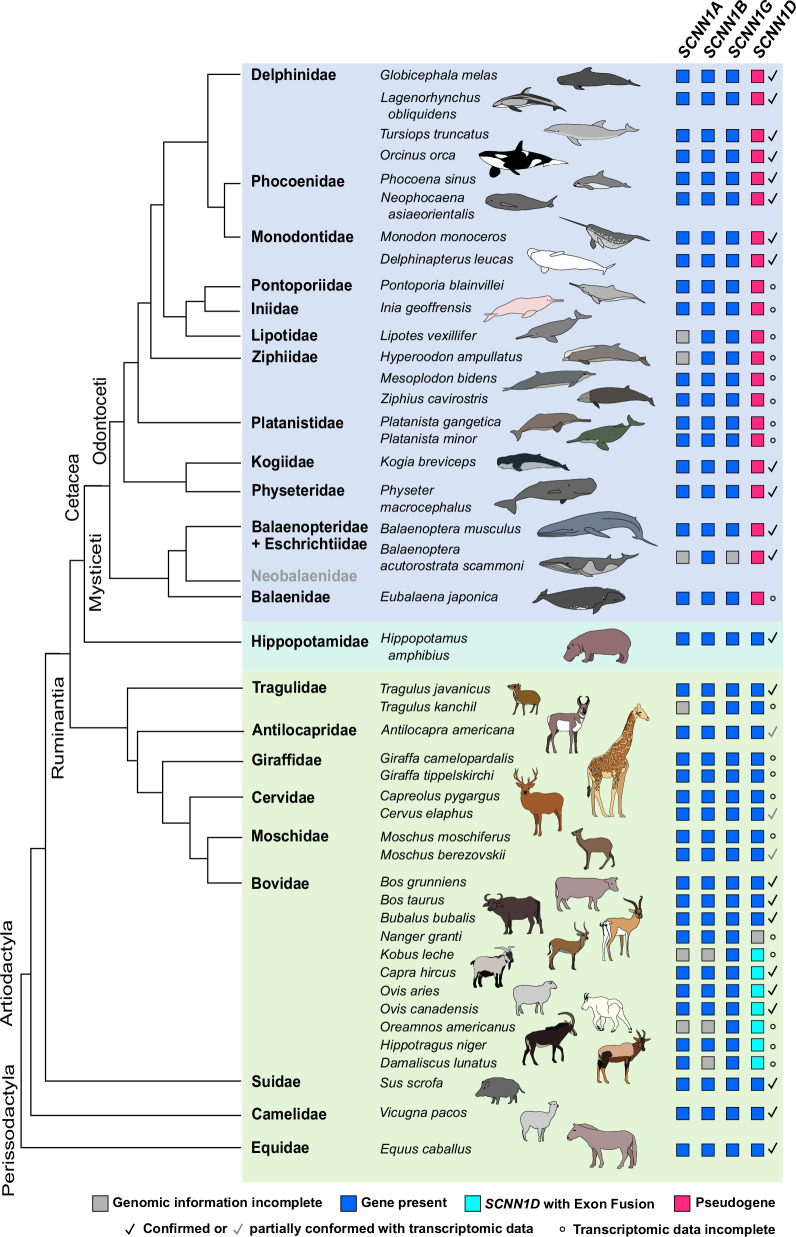
Presence of ENaC-coding *SCNN1* genes in Cetartiodactyla. Presence or pseudogenisation of *SCNN1* genes was investigated in the highlighted species using the National Center for Biotechnology Information (NCBI) database. Blue squares indicate intact genes yielding continuous open reading frames (ORFs), whereas magenta squares indicate pseudogenes containing multiple insertions/deletions and splice donor/acceptor site mutations causing preliminary STOP codons. Turquoise squares highlight species containing a fusion of exons 11 and 12 due to a mutation of the splice donor following exon 11, which did not disrupt the ORF. Grey squares highlight incomplete genomic information. There is currently no available genomic information of species representing the family Neobalaenidae. Check mark symbols = Coding sequences or erosion of *SCNN1D* was confirmed using transcriptomic data available at the NCBI Sequence Read Archive. Accession numbers are provided in Supplementary Table 1. ° = transcriptomic data incomplete. The phylogenetic tree was created based on Cabrera et al.^69^. Background colour code: light blue = marine species; turquoise = amphibious species; green = terrestrial species.

The structures of the *SCNN1A*, *SCNN1B* and *SCNN1G* genes were highly conserved in the analysed cetacean and terrestrial artiodactylan species (Fig. 1, Supplementary Data 1^24^). However, we observed clade-specific gene alterations. For example, in *SCNN1A* we detected a 3-base microdeletion under preservation of the reading frame in exon 4 of the Odontoceti (toothed whales; represented by *Tursiops truncatus* and *Inia geoffrensis* in Fig. 1A), except for the Indian river dolphins (Platanistidae), and sperm whales (Kogiidae and Physeteridae) (Supplementary Data 1). The 3-base microdeletion in the river dolphin families Pontoporiidae, Iniidae and Ziphiidae, but not the Platanistidae, is consistent with the hypothesis that Platanistidae are an early diverging group of the Odontoceti, distant from the other river dolphin families^25,26^ (Fig. 2). By contrast, exon 4 carried an insertion of 3 bases in the Ruminantia (e.g. *Tragulus javanicus*, *Bos taurus*, *Ovis aries*) (Fig. 1A, Supplementary Data 1). All analysed Cetartiodactyla contained a rare non-canonical GC splice donor motif^27^ flanking exon 6 (Fig. 1A, Supplementary Data 1), which was confirmed in the Bottlenose dolphin (*T. truncatus*) by transcriptomic data. Nevertheless, the analysed *SCNN1A*, *SCNN1B* and *SCNN1G* sequences yielded continuous ORFs (Supplementary Alignments 1–3). An alignment of the obtained amino acid sequences to human orthologues revealed high similarities, including: (i) the presence of key structural and regulatory sequence motifs such as an N-terminally located HG-motif impacting channel open probability, (ii) a conserved cation binding site initiating SSI in the α-ENaC subunit (*SCNN1A*), (iii) furin cleavage sites in the α-ENaC (*SCNN1A*) and γ-ENaC (*SCNN1G*) subunits, (iv) key cysteines involved in stabilising tertiary structure, (v) a GSS/A motif playing a role in ion size discrimination, as well as (vi) C-terminally located PPPxY motifs controlling ENaC membrane abundance^10,28^. We confirmed high degrees of conservation in all core structural elements of each subunit by projecting the degree of sequence conservation based on the amino acid alignments (Supplementary Alignments 1–3) onto the Cryo-EM-derived structure for human ENaC subunits^21,29^ (Supplementary Fig.  1). These data clearly demonstrate the presence of functional genes encoding canonical αβγ-ENaC in the analysed Cetartiodactyla (Fig. 2).

### *SCNN1D* is a pseudogene in Cetacea

In contrast to the *SCNN1A*, *SCNN1B* and *SCNN1G* genes, the *SCNN1D* gene encoding the δ-ENaC subunit displayed significant differences and clade-specific structural changes (Fig. 1, Supplementary Data 1^24^). Consistent with our previous analysis of the *SCNN1D* gene in rodents^17^, the gene is condensed due to smaller intron sizes, in comparison to the *SCNN1A*, *SCNN1B* and *SCNN1G* genes (Supplementary Data 1^24^). The *SCNN1D* gene contains multiple insertions/deletions and mutations of splice donor/acceptor sites in all analysed cetacean species, causing frame-shifts and premature STOP codons, thereby disrupting the ORFs. A comprehensive map of these alterations is provided in Supplementary Data 1. By contrast, the *SCNN1D* genes were intact and yielded continuous ORFs in the investigated terrestrial Artiodactyla (Fig. 1, Supplementary Alignment 4). The presence of a presumably functional *SCNN1D* gene in the Hippopotamus (*H. amphibius*) suggests that *SCNN1D* pseudogenised early in the evolution of Cetacea (Figs. 1 and 2, Supplementary Data 1^24^). It is a pseudogene in both the toothed whales (Odontoceti) and baleen whales (Mysticeti), suggesting that loss of a functional *SCNN1D* gene is a general feature of cetaceans since more than 36.4 million years^30–32^. All investigated cetaceans share an identical mutated splice donor following exon 5 and share mutations in the splice donor following exon 9, as well as the splice acceptor ahead of exon 11 (Supplementary Data 1). This suggests that a single pseudogenisation event may have happened at the base of cetacean origin and additional inactivating mutations and insertions/deletions accumulated later in the different cetacean families.

### Structural alterations in *SCNN1D* of Antilopinae

**Fig. 3:**
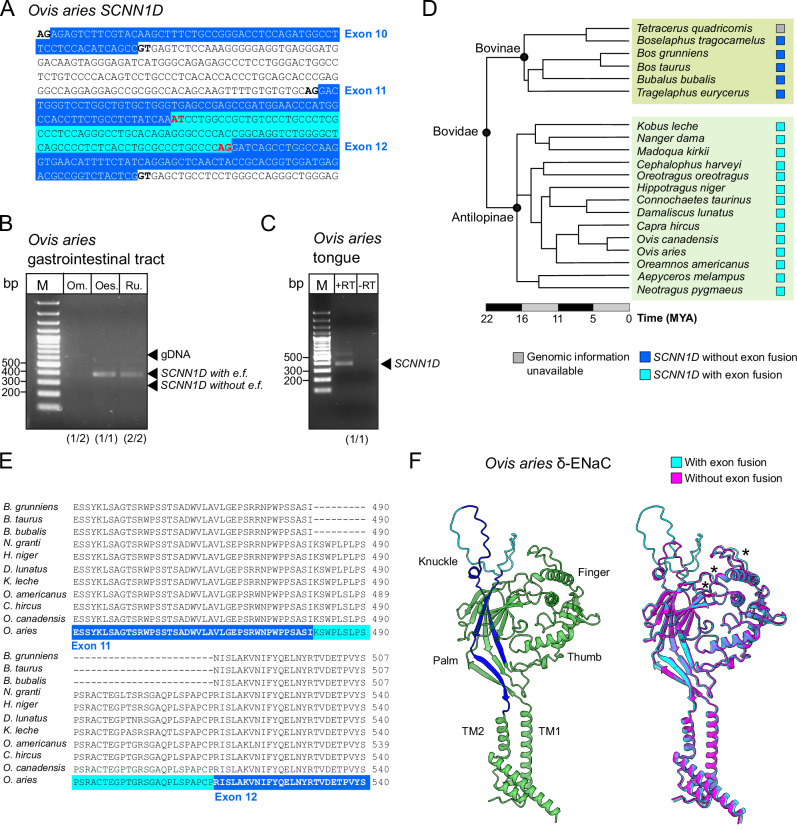
Altered *SCNN1D* genes in Antilopinae. **A** Mutation of the splice donor following exon 11 to ‘AT’ in *Ovis aries* (representative of the Antilopinae) causes fusion of exons 11 and 12 to a ‘super exon’. **B** Reverse transcriptase (RT)-PCR confirmed the exon fusion in cDNA derived from mRNA isolated from O. *aries* gastrointestinal tissues. Om. Omasum, Oes. Oesophagus, Ru. Rumen. Arrows highlight expected amplicon sizes based on absence or presence of the exon fusion (e.f.) or contamination with genomic DNA (gDNA). bp base pairs. M = DNA size marker. **C** Reverse transcriptase (RT)-PCR confirmed expression of *SCNN1D* in sheep tongue tissue. bp base pairs. M = DNA size marker. **D** Presence of the *SCNN1D* exon fusion in Bovidae. *SCNN1D* with exon fusion (turquoise squares) is exclusively found in representative species of the Antilopinae, but not in the Bovinae. MYA million years ago. **E** Partial δ-ENaC amino acid sequence alignment of representative species of the Bovidae. The sequence highlighted in blue indicates amino acids encoded by exons 11 and 12, the sequence highlighted in turquoise indicates additional amino acids due to incorporation of the former intron. **F** *O. aries* δ-ENaC structures predicted by ColabFold suggest location of the additional amino acids in the ‘knuckle’ domain. For clarity, predictions of the intracellular N-/C-termini are not displayed. Left: The predicted structures follow the canonical ENaC subunit architecture. Dark blue parts are derived from former exons 11 and 12, turquoise parts highlight additional ‘intron-derived’ amino acids. Right: Alignment of the *O. aries* δ-ENaC structure containing the exon fusion (turquoise) to a hypothetical *O. aries* δ-ENaC lacking the exon fusion (magenta). The exon fusion does not impair the general channel architectures but might alter the position of loops (highlighted by asterisks) between the ‘knuckle’ and ‘finger’ domains.

We observed a fusion of exons 11 and 12 of the *SCNN1D* gene due to mutation of splice donor sites in the Bovidae (Figs. 1,  2, and 3A, B, Supplementary Data 1^24^). This exon fusion causes incorporation of the former intron separating exons 11 and 12 into the coding mRNA sequences but does not disrupt the ORFs. *SCNN1D* transcripts have previously been detected in the digestive tract of sheep (*Ovis aries*)^33^. We, therefore, isolated total mRNA of *O. aries* digestive tract tissues and confirmed the presence of the *SCNN1D* exon fusion using RT-PCR and sequencing of the PCR products (Fig. 3A, B). Transcripts of *SCNN1D* were also detected in *O. aries* tongue tissue (Fig. 3C). Interestingly, we previously observed the same exon 11/12 fusion in *SCNN1D* in the rodent infraorder Hystricognathi^17^, suggesting that this genetic alteration evolved at least twice and independently in mammals. To further investigate the evolutionary origin of the exon 11/12 fusion in the Bovidae, we analysed 19 representative species of 12 families (Fig. 3D): The Boselaphini (*B. tragocamelus*), Tragelaphini (*T. eurycerus*) and Bovini (*B. grunniens, B. taurus, B. bubalis*), grouped as the Bovinae, and the Aepycerotini (*A. melampus*), Neotragini (*N. pygmaeus*), Antilopini (*N. dama, M. kirkii*), Reduncini (*K. leche*), Oreotragini (*O. oreotragus*), Cephalophini (*C. harveyi*), Alcelaphini (*D. lunatus, C. taurinus*), Hippotragini (*H. niger*) and Caprini (*C. hircus, O. aries, O. canadensis, O. americanus*), grouped as the Antilopinae^34^. The exon fusion was present in all investigated Antilopinae but absent in the Bovinae. The exon fusion can therefore be considered an autapomorphic feature of the Antilopinae and is a feature of this clade since at least 16 million years (Timetree, accessed 12/2023).

The fusion of exons 11/12 and inclusion of the former intron incorporates additional amino acids into the δ-ENaC protein. Amino acid alignment of select Bovidae indicates that the incorporated 33 amino acids are highly conserved (Fig. 3E). ColabFold predictions of the sheep (*O. aries*) δ-ENaC^24^ yielded a protein structure that is consistent with the Cryo-EM derived ‘clenched-hand holding a ball of β-sheets’ structures of human ENaC subunits^29^ (Fig. 3F). The additional 33 amino acids are placed in a flexible loop at the top surface of the protein, termed the ‘knuckle’ domain. Removal of the additional amino acids^24^ does not cause major structural rearrangements in the core protein, but subtle changes are predicted in the ‘finger’ domain and the region connecting the ‘finger’ and ‘knuckle’ domains (Fig. 3F). These regions contain important ENaC regulatory motifs, such as the ‘gating relief of inhibition by proteolysis’ (GRIP) domains and sodium binding sites^21,29^. Furthermore, recent Cryo-EM derived structures suggest inter-subunit interactions between the ‘knuckle’ and ‘finger’ domains of neighbouring ENaC subunits that are associated with different functional properties of the channels^35^. The incorporation of the additional amino acids in the ‘knuckle’ domain might have therefore altered biophysical or regulatory mechanisms in δ-ENaC of the Antilopinae, but putative gain-of-function or loss-of-function effects remain to be confirmed in electrophysiological experiments before conclusions on the physiological consequences of the observed alteration in this clade can be drawn. Interestingly, the previously observed *SCNN1D* exon 11/12 fusion in the rodent infraorder Hystricognathi^17^ affects the same extracellular ‘knuckle’ region of the ion channel. However, the incorporated intron in the Hystricognathi is considerably smaller than that of the Antilopinae, illustrating the structural flexibility of the extracellular ‘knuckle’ region of the ion channel.

### Chemosensory traits correlate with *SCNN1D* erosion in cetaceans

Overall, our bioinformatic analyses clearly demonstrate pseudogenisation of *SCNN1D* in cetaceans, while the gene is maintained, including clade-specific alterations, in terrestrial Artiodactyla. What drove the loss of δ-ENaC in whales and dolphins, and what is the reason for its maintenance in their terrestrial sister groups? With respect to sodium homoeostasis, two major physiological changes appeared with the transition from a terrestrial to a marine environment: a change from herbivory/omnivory to carnivory^1^ and the independence from freshwater sources^8^. Carnivores generally ingest food with similar biochemical compositions to their own bodies, thereby making specific chemical senses less necessary, while herbivores ingest a larger variety of potentially harmful plant compounds^36^. Consistently, pseudogenisation of taste receptor genes has been described in several cetacean species but not in terrestrial Artiodactyla^5,6^. While extinct cetacean ancestors such as *Ambulocetus* likely relied on freshwater^8^, modern marine cetaceans do not rely on freshwater sources, and they are well adapted to maintain electrolyte homoeostasis in a hypersaline marine environment^37^. Hence, a necessity to discriminate seawater from freshwater (based on sodium content), or a ‘stress response’ warning of sodium concentrations greater than plasma concentrations, appears less necessary in cetaceans, while it might be vital in terrestrial Artiodactyla. Furthermore, herbivorous diet generally demands more stringent sodium-conserving mechanisms: sodium concentrations in plant tissue are lower than in animal tissue and herbivores often look for sites for sodium supplementation in their environments (mineral licks)^38^. Digestion of plant material also requires significant amounts of saliva (with sodium as the main cation) ensuring an optimal environment for microbial digestion, and sodium reabsorption in the kidneys and gastrointestinal tract is pivotal to avoid a sodium deficit in ruminants^39^. Renal and gastrointestinal adaptations, as well as adaptations with respect to chemical sodium senses might therefore explain the pseudogenisation of *SCNN1D* in cetaceans and its preservation in terrestrial Artiodactyla.

**Fig. 4:**
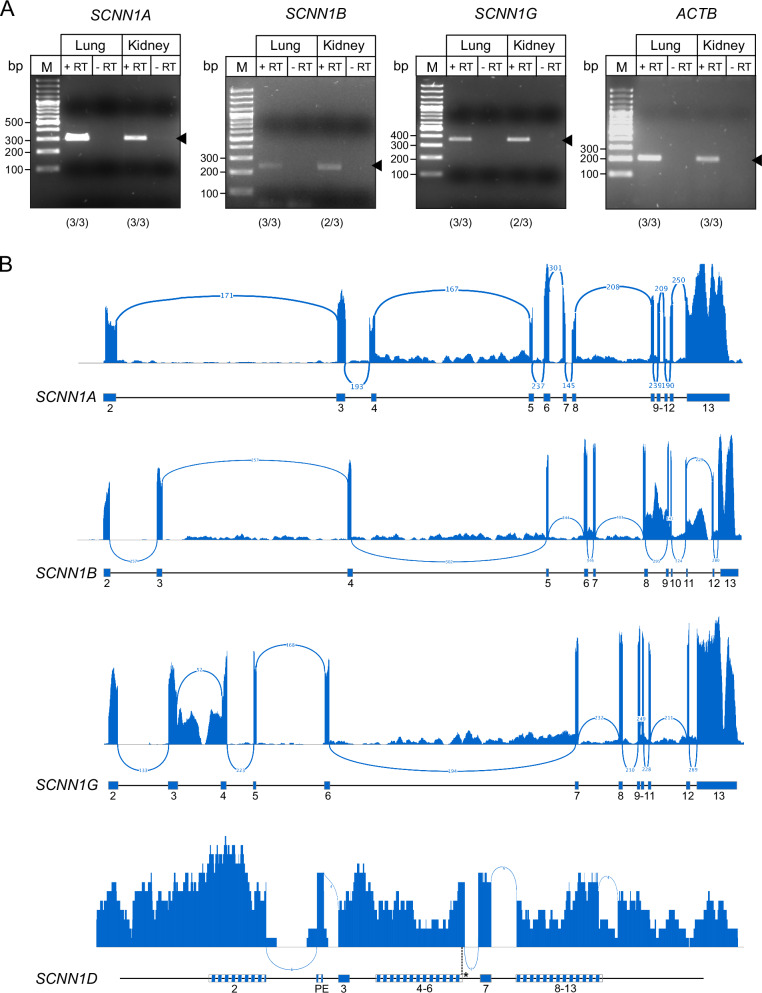
Expression of ENaC-coding genes in Bottlenose dolphin (*Tursiops truncatus*) tissues. **A** RT-PCRs were performed with cDNA synthesised from tissues derived from three animals. Arrow heads point towards the expected amplicon sizes. Numbers in parentheses indicate positive amplicons out of the total number of animals. PCR results from all animals are provided in ref. ^24^. β-actin (*ACTB*) was used as controls. RT reverse tanscriptase, M = DNA size marker; bp base pairs. **B** Splicing profiles of *SCNN1A*, *SCNN1B*, *SCNN1G*, and *SCNN1D* in *Tursiops truncatus* skin tissue based on RNA-seq data (GEO: SRX7005031). Sashimi plots were generated using Integrative Genomics Viewer (IGV v2.19.2) from splice junction-aware RNA-seq alignments. A minimum of 30 supporting reads was set as the junction threshold for *SCNN1A*, *SCNN1B*, and *SCNN1G*, and a threshold of 2 reads for *SCNN1D* due to lower expression. Splice junctions are annotated with the number of supporting reads. PE denotes an out-of-frame pseudo-exon inclusion event observed in *SCNN1D*. An asterisk (*) marks a novel donor splice site (+7 nt) upstream of exon 6 in *SCNN1D*. Loss of predicted exon structure is observed in *SCNN1D*, suggesting either exon skipping or incomplete transcript assembly. Gene structure and exon numbering follow canonical annotations as also shown in Fig. 1.

Consistent with our bioinformatics data, previous studies demonstrated the presence of *SCNN1A*, *SCNN1B* and *SCNN1G* genes encoding αβγ-ENaC in some cetacean species, while the genes encoding sweet and bitter receptors pseudogenised^5,6^. These studies led to the conclusion that ‘salty’ is the only taste modality cetaceans can sense. However, αβγ-ENaC is not only key for maintaining vertebrate sodium homoeostasis but (i) also enhances the electrochemical gradients for potassium excretion via ‘Renal Outer Medullary Potassium’ (ROMK) channels in the principal cells of the distal nephrons, (ii) it controls the volume of liquid lining lung epithelia^14^ and (iii) is important for epidermal barrier maintenance^40^. We isolated mRNA from kidney and lung tissues of three Bottlenose dolphins (*Tursiops truncatus*) and confirmed expression of αβγ-ENaC in these tissues by RT-PCR (Fig. 4A^24^). Furthermore, transcripts for αβγ-ENaC were detected in transcriptomic data derived from skin tissue (Fig. 4B). Although αβγ-ENaC has been shown to mediate appetitive sodium taste to low sodium concentrations in mice^12,13^, the presence of functional *SCNN1A*, *SCNN1B* and *SCNN1G* genes in cetaceans does therefore not necessarily associate with the ability to taste sodium, given the importance of this ion channel in dolphin kidneys, lungs and skin. We were also able to detect fragments of *SCNN1D* transcripts in *T. truncatus* skin tissue (Fig. 4B). This supported the generally disrupted exon/intron structure and revealed the presence of an additional out-of-frame pseudo-exon in the *SCNN1D* gene.

Whether or not cetaceans have a functional gustatory system and can taste sodium remains controversial^41,42^. Behavioural studies demonstrated sodium tasting abilities in Bottlenose dolphins^43,44^ however, these studies were performed on very few animals, were not blinded and have been criticised due to their Go/No-Go experimental design^45^. We, therefore, performed a series of blinded behavioural experiments on Bottlenose dolphins (*T. truncatus*) and Beluga whales (*Delphinapterus leucas*) under human care to investigate their sodium tasting abilities.

**Fig. 5:**
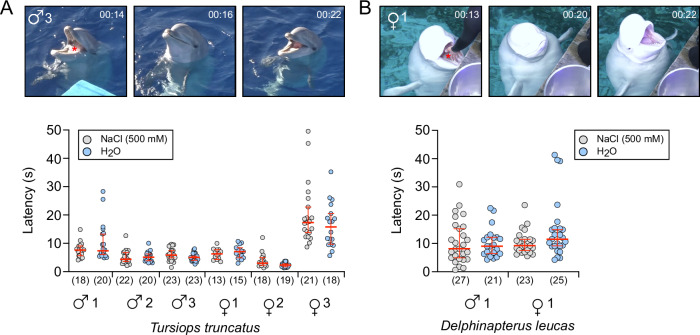
Behavioural assessment of salt tasting abilities in cetaceans. **A**, **B** The latency of begging behaviour (s) in response to feeding of 3 gelatine blocks with or without 500 mM NaCl was determined for 6 Bottlenose dolphins (**A**) and 2 Beluga whales (**B**). The images are video stills from the recorded sessions, indicating the time of gelatine block feeding until the display of begging behaviour. Gelatine blocks are marked with a red asterisk. Numbers in parentheses indicate “*n*”. Red lines indicate medians ±95% confidence intervals. For statistical analyses, the dataset was modelled with a mixed effects structure in a Bayesian framework. Statistical data are provided in Supplementary Data 2 and^24^. Numbers in parentheses indicate “*n*”. Red lines indicate medians ±95% confidence intervals (Mann–Whitney U-Test).

We modified an experimental approach that has been employed to study spontaneous responses and preferences to chemical stimuli in cetaceans^45^. Cetaceans under human care are routinely offered gelatine blocks to provide hydration and nutritional enrichment. We provided gelatine blocks prepared with or without 500 mM NaCl (similar to seawater concentrations^46^) to six Bottlenose dolphins (three males and three females) and two Beluga whales (one male and one female) and determined the latency of begging for further gelatine blocks as a measure of discrimination between gelatine with or without added NaCl (Fig. 5A, B). We considered the gelatine without added NaCl the atypical exposure for marine cetaceans. Experiments were blinded and the order of gelatine block application was randomised. In total, there were 230 latency observations for Bottlenose dolphins and 96 latency observations for Beluga whales. Latency times varied between individual animals but were highly similar between gelatine with or without NaCl (Fig. 5A, B, Supplementary Data 2^24^). Latency data were logarithmically transformed and modelled with a mixed effects structure in a Bayesian framework, which demonstrated that the latency times did not statistically vary with the presence or absence of NaCl in the gelatine blocks in either Bottlenose dolphins or Beluga whales (Supplementary Data 2^24^). Thus, these experiments did not reveal measurable behavioural differences to stimuli with or without sodium in seawater-equivalent concentrations in cetaceans. However, we would like to emphasise that a limitation of these experiments is the underlying assumption that the animals would display measurable behavioural differences (shift in latency time) if they were able to distinguish between stimuli with or without NaCl. While previous studies with Bottlenose dolphins detected changes in latency of begging behaviour^45^ in response to chemical stimuli, the absence of equivalent behavioural changes in response to NaCl cannot be excluded.

A lack of sodium taste in cetaceans would be consistent with the pseudogenisation of *SCNN1D* and functional δ-ENaC in cetaceans. The emergence of this ENaC subunit with the transition from aquatic to terrestrial environments in the Devonian and its pseudogenisation with the transition from terrestrial to marine environments in the Eocene generally suggests a role for δ-ENaC in sodium homoeostasis in low-salinity and terrestrial habitats. A counterargument to this hypothesis is illustrated by extant river dolphin species which live in low-salinity environments and also lack a functional *SCNN1D* gene (Figs. 1 and 2). However, these species evolved from marine ancestors^25^, indicating that they did not regain δ-ENaC after migration from marine to freshwater environments. Furthermore, ENaC-independent physiological mechanisms for the detection of salinity might have evolved in cetaceans. For example, mechanical effects on odontoblastic processes in the Narwhal tusk have been hypothesised to mediate sensory responses to salinity gradients in these cetaceans^47^.

### Distribution of functional ENaC-coding genes in other marine mammals

**Fig. 6:**
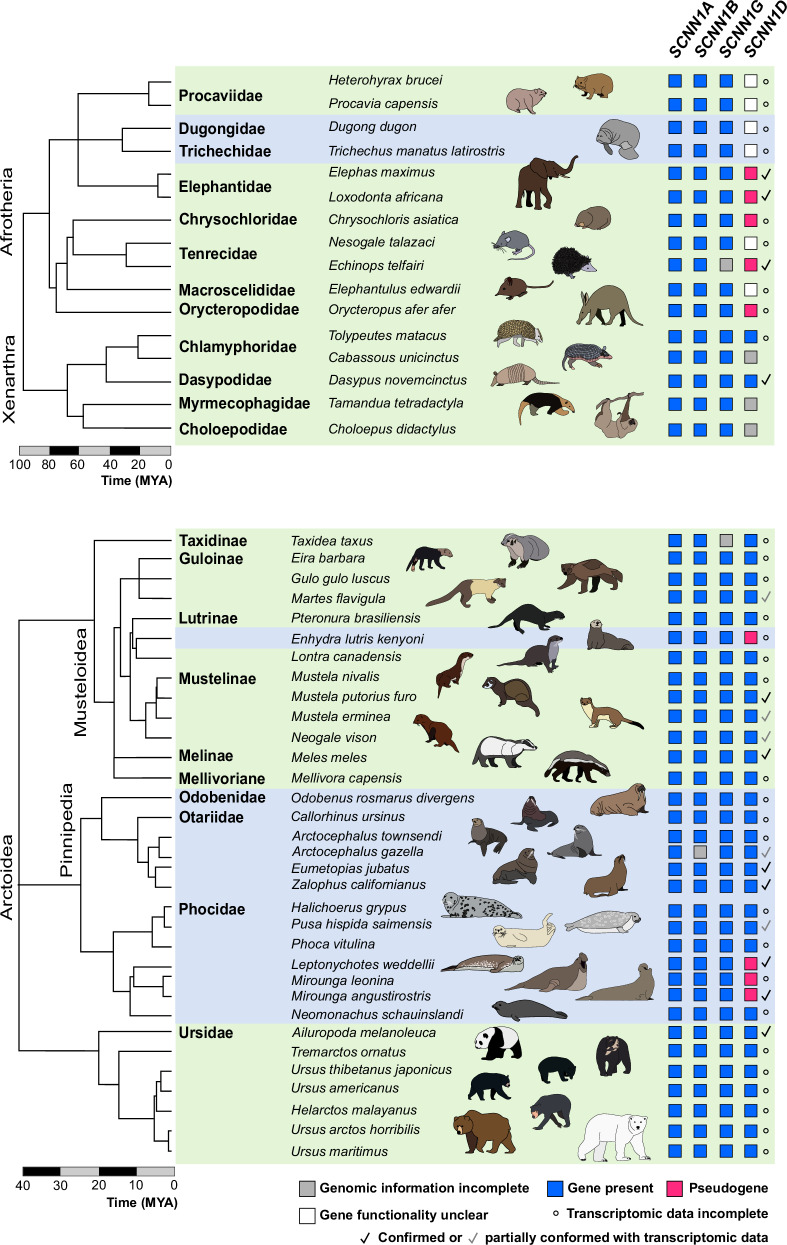
Presence of ENaC-coding *SCNN1* genes in Afrotheria and Carnivora. Presence or pseudogenisation of *SCNN1* genes was investigated in the highlighted species using the National Center for Biotechnology Information (NCBI) database. Blue squares indicate intact genes yielding continuous open reading frames (ORFs), whereas magenta squares indicate pseudogenes containing multiple insertions/deletions and splice donor/acceptor site mutations causing preliminary STOP codons. Grey squares highlight incomplete genomic information. Check mark symbols = coding sequences or erosion of *SCNN1D* was confirmed using transcriptomic data available at the NCBI Sequence Read Archive. ° = No transcriptomic data available. Accession numbers are provided in Supplementary Table 1. There is no transcriptomic data available for *Enhydra lutris*, but a STOP codon in exon 3 was confirmed in two sub-species, *E. lutris kenyoni* and *E. lutris nereis*. The phylogenetic trees and time indices were obtained from Timetree (http://timetree.org). Background colour code: light blue = marine species; green = terrestrial species.

Secondary transitions to marine environments have occurred multiple times and independently in mammals. We therefore investigated whether there is evidence for *SCNN1D* erosion in other marine lineages, including Sirenia (manatees and dugongs), Pinnipedia (seals) and the sea otter. Consistent with the analysed Cetartiodactyla, *SCNN1A*, *SCNN1B* and *SCNN1G* remain intact in all investigated species (Fig. 6, Supplementary Data 3^24^). In the investigated Afrotheria, *SCNN1D* is a pseudogene (due to the presence of STOP codons) in the elephants, Cape golden mole, the lesser hedgehog tenrec and the aardvark. For the aquatic Sirenia, an *SCNN1D* ORF could be constructed, but the presence of a potentially disrupted splice site between exons 2 and 3 could indicate pseudogenisation. Since there is currently no transcriptomic data available that covers this region of the *SCNN1D* gene, its functionality in Sirenia remains unclear (Fig. 6). The gene is intact in the two analysed Xenarthra (Fig. 6). Interestingly, the ancestors of the Paenungulata, a clade comprising the Sirenia, Proboscidea (elephants) and Hyracoidea (hyraxes), were likely amphibious^48^ and fossils of early sirenians and proboscideans are found in marine sediments^49^, suggesting that *SCNN1D* might have been lost before the transition back to a terrestrial environment. However, since there are no conserved inactivating mutations detectable within *SCNN1D* of the Paenungulata (Supplementary Data 3), together with the uncertainty on its functionality in Sirenia, this hypothesis remains to be tested. Furthermore, the gene also pseudogenised in the investigated species belonging to the afrotherian clade Afroinsectiphilia (aardvark, elephant shrews, tenrecs).

Within the Mustelidae, a clade of the Carnivora including otters, badgers, martens and weasels, *SCNN1D* pseudogenised only in the sea otter (*Enhydra lutris kenyoni*) (Fig. 6, Supplementary Data 3), consistent with the loss of *SCNN1D* in species adopting a marine lifestyle. In the Pinnipedia, we detected pseudogenisation of *SCNN1D* in three species belonging to the Phocidae, two elephant seal species (*Mirounga leonina* and *Mirounga angustirostris*) as well as the Weddel seal (*Leptonychotes weddellii*), while it remained intact in the Otariidae (eared seals). There are no shared inactivating mutations in the elephant seals and Weddel seal, suggesting that *SCNN1D* pseudogenised independently. In the Ursidae (bears), *SCNN1D* remains intact in all investigated species, including the polar bear (*Ursus maritimus*).

### Clade-specific changes in selective pressures on *SCNN1* genes

In order to detect changes on selective pressures on the *SCNN1* genes, we used codon alignments of the four genes of all investigated species, in combination with rooted trees of the four *SCNN1* genes. We applied tests for site-specific and branch-specific effects^24^. The result of the BUSTED (branch-site unrestricted statistical test for episodic diversification) test for episodic diversification was significant for the genes *SCNN1A* and *SCNN1D* (Table 1). FEL (fixed effects likelihood) and SLAC (single-likelihood ancestor counting) tests flagged a small number of sites (less than ten) to be under positive selection in all the four genes under study. The number of sites under negative (= stabilising) selection comprised about 50% (FEL) or 40% (SLAC) of the codons in genes *SCNN1A*, *SCNN1B* and *SCNN1G*, while it was substantially less in *SCNN1D* (36% in FEL and 23% in SLAC test). This suggests that *SCNN1D* is evolving under relatively relaxed conditions, at least for a few branches. Testing with SLAC explicitly for single branches it became apparent that pinnipeds (just 2 sites under negative selection) are strikingly different from terrestrial branches of Artiodactyla (54 sites under negative selection) or Carnivora (49 sites under negative selection)^24^. Thus, the loss of this gene in some pinnipeds makes sense as it seems to evolve under much more relaxed conditions in this branch. To address this hypothesis, we tested for relaxed or intensified selection (RELAX) (Table 2). The test was initially showing an intensified selection on *SCNN1D* for Pinnipedia, which was surprising giving the pseudogenisation of that gene in three species belonging to this clade. Separate analysis of the two branches of Phocidae (including the three pseudogenes) and Otariidae demonstrated a strong signal for intensified selection in the Otariidae, while selection seems to be relaxed in Phocidae (Table 2). The reason for this difference between the branches remains to be investigated. The other three ENaC genes did not show signals for relaxed or intensified selection in any of the pinniped families. In cetaceans, where no alignment for *SCNN1D* could be generated due to erosion of the gene, *SCNN1A* and *SCNN1G* alignments show a significant signal for relaxed selection on these genes, contrasting with their physiological importance in renal, lung and epidermal physiology. Since the alterations of *SCNN1D* in Afrotheria are predominantly disruptions in splice sites, we reconstructed exon sequences irrespective of the splice sites and aligned them with the functional *SCNN1D* genes of the other taxa. The *SCNN1D* gene seems to be generally under relaxed selection in Afrotheria, as well as when just Sirenia are tested (Table 2). This further supports its pseudogenisation in Afrotheria. There is also evidence for relaxed selection on *SCNN1A* and *SCNN1G* in Afrotheria, while in Sirenia this signal is only significant in *SCNN1A*.

**Table 1:** Statistical tests on site-wise selection

Gene	No. codons	busted	FEL (pos)	FEL (neg)	SLAC (pos)	SLAC (neg)
*SCNN1A*	716	*p* = 0.049*	8	360	7	241
*SCNN1B*	653	*p* = 0.08	5	375	7	235
*SCNN1G*	672	*p* = 0.5	7	393	2	289
*SCNN1D*	685	*p* < 0.001*	6	245	5	156

busted: *p*-value for any sites under positive selection, FEL/SLAC: number of sites under pos./neg. selection, * 0.05 > *p* > 0.01, ** 0.01 > *p* > 0.001, *** 0.001 > *p*.

**Table 2:** Likelihood ratio test (LRT) tests of relaxed or intensified selection (RELAX) in specific branches versus background

Gene	Cetacea	Pin: Otariidae	Pin: Phocidae	Sea otter	Sirenia	Afrotheria
*SCNN1A*	*p* = 0.0011**	*p* = 0.24	*p* = 0.11	*p* = 0.0037**	*p* = 0.0072**	*p* = 0.023*
*SCNN1B*	*p* = 0.93	*p* = 0.57	*p* = 0.11	*p* = 0.76	*p* = 0.14	*p* = 0.37
*SCNN1G*	*p* < 0.001***	*p* = 0.30	*p* = 0.32	*p* = 0.032*	p = 0.096	*p* = 0.027**
*SCNN1D*	ND	*p* < 0.001***	*p* < 0.001***	*p* = 0.12	*p* = 0.012*	*p* < 0.001***

Bold values show significant intensification of selection.

*Pin* pinnipedia.,* 0.05 > *p* > 0.01, ** 0.01 > *p* > 0.001, *** 0.001 > *p*.

Likelihood-ratio tests for all four genes show that dn/ds ratio is significantly better modelled when a non-uniform ratio is assumed, comparing different branches (Table 3). We especially checked the aquatic branches independently from the terrestrial ones. Although dn/ds ratios are generally below 1 for the genes in total (meaning that in general negative selection is predominant over all sites), pinnipeds and cetaceans have slightly higher dn/ds ratios in comparison to the background (= the terrestrial taxa). The sea otter has values greater than 1 in genes *SCNN1A* and *SCNN1G,* suggesting strong positive selection on these genes. Although below 1, the pseudogene *SCNN1D* of the sea otter (which could be reconstructed due to minimal alterations and absence of deletions) has the highest value in comparison to the other branches. A test for relaxed or intensified selection (RELAX) also shows intensified selection of *SCNN1A* and *SCNN1G* in the sea otter, while there is no signal in the pseudogene *SCNN1D* (Table 2). As described above, the Antilopinae underwent an extra insertion in *SCNN1D* (fusion of exons 11/12). Interestingly, here *SCNN1D* shows a smaller dn/ds ratio compared to the background, implying a stronger negative selection (Table 3).

**Table 3:** Likelihood ratio test (LRT) of uniform (M0) versus branch-specific dn/ds ratios (M2) and dn/ds values for background and selected branches

Gene	LRT	dn/ds	dn/ds	dn/ds	dn/ds	dn/ds	dn/ds	dn/ds
	M0 vs M2	all (M0)	backgr.	Cetacea	Pinnipedia	Sirenia	Sea otter	Antilopinae
*SCNN1A*	*p* < 0.001***	0.126	0.111	0.308	0.187	0.091	1.719	ND
*SCNN1B*	*p* = 0.002**	0.096	0.089	0.148	0.102	0.121	0.057	ND
*SCNN1G*	*p* < 0.001***	0.126	0.11	0.301	0.185	0.089	1.91	ND
*SCNN1D*	*p* < 0.001***	0.208	0.208	ND	0.263	ND	0.433	0.137

* 0.05 > *p* > 0.01, ** 0.01 > *p* > 0.001. *** 0.001 > *p*.

**Fig. 7:**
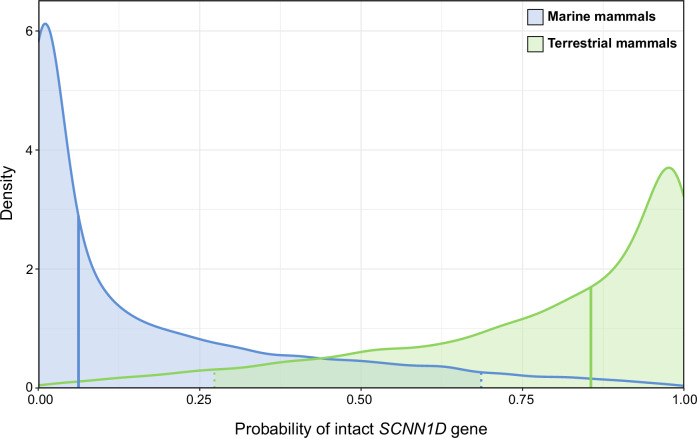
The effect of marine versus non-marine habitats on *SCNN1D* pseudogenisation in mammals. The presence of a functional *SCNN1D* gene was modelled as a response to marine (blue) and terrestrial (green) while accounting for phylogenetic relatedness between mammal species, with posterior distributions indicating that non-marine mammals have a higher probability of possessing a functional *SCNN1D* gene (pMCMC = 0.046). Posterior medians plotted as solid vertical lines and 95% highest probability interval as dashed lines.

To finally test whether pseudogenisation of *SCNN1D* is generally related to the return of seawater habitats in mammals, we ran a phylogenetic generalised linear mixed model covering the mammal clades that include marine mammal representatives (Figs. 2 and 6). The probability of a functional *SCNN1D* gene was considerably higher for non-marine mammals (median of 0.856 [0.273 – 1 95% HPDI]) than marine mammals (0.062 [0 – 0.686]) (pMCMC = 0.046, Fig. 7^24^). This was despite a strong phylogenetic signal (marginal − *r*^2^ = 0.434 [0.015 – 0.569], conditional − *r*^2^ = 0.939 [0.837 − 1]).

In summary, the analysis of *SCNN1D* in marine mammals reveals the following patterns: (1) *SCNN1D* tends to pseudogenise in lineages that transitioned to marine environments, including cetaceans, potentially sirenians, pinnipeds (Phocidae) and the sea otter. (2) Pseudogenisation or preservation of *SCNN1D* is not consistent with herbivorous or carnivorous diet, thereby suggesting that dietary/gastrointestinal adaptations are not sufficient to explain the loss of this gene. (3) *SCNN1D* is also a pseudogene in some terrestrial mammals, including the Afroinsectiphilia (Fig. 6), as well as five rodent lineages^17^. It has been hypothesised that δβγ-ENaC represents a ‘stress protein’ monitoring high sodium concentrations. Selection pressure for such a sodium sensing mechanism may not only decrease upon transition into a high-salinity marine environment, but it may, for example, also be reduced when renal sodium handling abilities of terrestrial species renders high salt intake less problematic. Such scenarios might explain the loss of *SCNN1D* in terrestrial species.

## Conclusion

Overall, αβγ-ENaC is a key ‘maintenance protein’ controlling the overall body sodium homoeostasis in mammals. Furthermore, it is important for renal potassium handling, control of the volume and composition of lung lining liquids and epidermal barrier maintenance^14^. These physiological functions are required in terrestrial and marine mammals and explain the presence of αβγ-ENaC transcripts in kidney and lung tissues from marine species, e.g. Bottlenose dolphins. By contrast, functional electrophysiological studies on heterologously expressed δβγ-ENaCs suggest that this isoform is primed for operation under high extracellular sodium concentrations due to reduced regulatory constraints such as SSI. In humans, δ-ENaC is not expressed in the kidney, but transcripts are found in taste buds^23,50,51^ as well as immune cells, which may have an ENaC-mediated mechanism sensing high extracellular sodium concentrations^52^. These lines of evidence point towards a function as a ‘stress protein’ monitoring high sodium concentrations in tetrapod vertebrates. This function might have become obsolete in animals which migrated to high-salinity marine environments. The pseudogenisation of *SCNN1D* might therefore be a consequence of transition to marine environments rather than an adaptive loss that led to phenotypic innovation facilitating environmental transition.

## Methods

### Bioinformatic analyses of Cetartiodactyla genomes

*SCNN1* gene sequences were analysed using a previously published approach^17,53^: The exon sequences of the *SCNN1A*, *SCNN1B*, *SCNN1G* and *SCNN1D* genes of the Alpaca (*Vicugna pacos*) were used as a template for the initial search for *SCNN1* genes in Cetartiodactyla since all four *SCNN1* genes are intact in this species and it represents a sister-group to the cetaceans. To facilitate comparability between datasets, the same species was used as a reference when *SCNN1* genes were investigated in other mammals (Carnivora, Afrotheria, Xenarthra). The Alpaca exon sequences were used to blast the whole genome contig database of the National Center for Biotechnology Information (NCBI BLAST) for the investigated species with default settings for blast and megablast searches. Exons 2-13 (according to the nomenclature described in Gettings et al.^17^) of all *SCNN1* genes were aligned to the Alpaca template sequences using Multalin^54^. For each investigated species, details on exon and intron sizes, splice donor/acceptor sites as well as insertions/deletions with respect to the Alpaca templates are provided in Supplementary Data 1,  3 and^24^. In some cases, putative alternative splice sites were predicted using the ASSP alternative splice site predictor^55^. Coding sequences were translated using the Expasy Translate Tool to retrieve ORFs. Exon/intron boundaries as well as coding sequences for *SCNN1D* were validated in all investigated species using transcriptomic data obtained from the NCBI Sequence Read Archive (SRA). A full list of analysed datasets is provided in Supplementary Table 1. Putative coding sequences were blasted against the corresponding SRA entry and intron-spanning reads were analysed to verify the coding sequences. RNA-seq data from *Tursiops truncatus* skin tissue (GEO accession SRX7005031) were processed using the nf-core/rnaseq pipeline (v3.14) with default parameters. The reference genome and corresponding GTF (gene transfer format) annotation were retrieved from the NCBI genome repository. Read alignment was performed using STAR (Spliced Transcripts Alignment to a Reference) and read depth over the *SCNN1* gene loci was calculated using samtools depth. The average coverage was 106.742× for *SCNN1A* (NC_047044.1:96317511–96338564), 17.8481× for *SCNN1B* (NC_047048.1:20458994–20536072), 4.30532× for *SCNN1D* (NC_047034.1:183381001–183390698), and 31.5547× for *SCNN1G* (NC_047048.1:20587754–20614704). Alternative splicing was visualised using Sashimi plots in Integrative Genomic Viewer (IGV, v2.19.2) with a minimum splice junction threshold of 30 supporting reads for *SCNN1A*, *SCNN1B*, and *SCNN1G*, and a reduced threshold of 2 reads for *SCNN1D* due to low expression levels. Junctions are annotated with supporting read counts, and gene structures follow canonical annotation.

Amino acid sequences were aligned using Clustal Omega. Structure predictions of δ-ENaC subunits from sheep (*O. aries*) displayed in Fig. 3 were performed with ColabFold v1.5.5^56^ based on amino acid sequences derived from genomic analyses^24^. Molecular graphics and analyses were performed with UCSF ChimeraX^57^, developed by the Resource for Biocomputing, Visualisation, and Informatics at the University of California, San Francisco, with support from National Institutes of Health R01-GM129325 and the Office of Cyber Infrastructure and Computational Biology, National Institute of Allergy and Infectious Diseases. The employed databases and online resources are listed in Supplementary Table 2.

### Phylogenetic analyses of *SCNN1* genes

Time-calibrated phylogenetic trees were generated using Timetree (accessed November 2023)^58^. For the analysis of selection pressure, we used codon alignments of the four genes in combination with rooted trees of the four *SCNN1* genes^24^.

Codon alignments were done with Seaview, using the built-in muscle tool^59^. In seaview nucleotide sequence sets can be toggled to amino acid sequences, aligned as such, and toggled back to nucleotides. We applied tests for site-specific and branch-specific effects. BUSTED, FEL, RELAX and SLAC were conducted with the HyPhy package^60^. With BUSTED (branch-site unrestricted statistical test for episodic diversification) we tested if there is at least one site under positive selection in the whole phylogeny^61^. FEL (fixed effects likelihood^62^) tests for deviation from an even distribution of dn/ds ratio across the phylogeny. RELAX (relaxed selection test^63^) uses dn/ds ratio for testing a test branch versus a reference branch (or all background branches) for relaxed or intensified selection. SLAC (single-likelihood ancestor counting^62^) analyses dn/ds ratio on a per site basis, suggesting sites under positive or negative selection pressure. General dn/ds ratio for whole genes was analysed with codeml from the PAML package^64^. Hypothesis testing for different settings (uniform dn/ds versus branch-wise variation) was then done using the likelihood ratio test (LRT) as implemented in EasyCodeML^65^.

### Analysis of *SCNN1D* pseudogenisation related to saltwater habitats

A phylogenetic generalized linear mixed model (GLMM) covering the mammal clades that include marine mammal representatives (Figs. 2 and 6) was run to test whether pseudogenisation of the *SCNN1D* is related to the return of saltwater habitats in mammals. The presence of a functional *SCNN1D* gene was modelled in a Bayesian framework as a binary response (1 representing a functional gene and 0 pseudogenisation with the logit link function) to mammal habitat (marine vs. non-marine) with phylogenetic relatedness as a random effect. The model was run via the brms package^66^ in R using default priors except for intercepts, which were given a prior of student_*t*(4, 0, 1.5) that is near-uniform on the logit scale. The phylogenetic tree was obtained from Timetree.org (93 species total). The model was run with 3 chains for 6000 iterations, with a warm-up of 3000 and thinning of 1, providing 9000 posterior draws. The R script for running and plotting phylogenetic GLMM, the R data file containing the brms model, the Timetree phylogenetic tree file, as well as a list containing species names and *SCNN1D* response (intact or no) are provided in ref. ^24^.

### Tissue mRNA isolation and reverse transcriptase (RT)–PCR

Sheep (*O. aries*) gastrointestinal tissue samples were kindly provided by Dr. Franziska Liebe, Institute for Veterinary Anatomy, Free University Berlin, Germany. Tissue isolation was under governance by the Berlin Veterinary Health Inspectorate (Landesamt für Gesundheit und Soziales Berlin) and approved under the animal experimentation number G0098/21. Sheep tongue tissue samples were kindly provided by Prof. Franziska Dengler, Department of Biochemical Sciences, Institute of Physiology, University of Veterinary Medicine, Vienna, Austria. Tissue isolation was under governance by the Federal Ministry Republic of Austria, Education, Science and Research, and approved under the animal experimentation number BMBWF-68.205/0100-V/3b/2018. Bottlenose dolphin (*T. truncatus*) kidney and lung tissue samples were kindly provided by the tissue bank of Fundación Oceanogràfic, Valencia, Spain and Mundomar Benidorm, Alicante, Spain. Tissue samples were stored in NEB’s DNA/RNA stabiliser solution at −80 °C (New England Biolabs, Frankfurt, Germany) according to the manufacturer’s instructions.

Bottlenose dolphin tissues were homogenised with the Precellys Evolution Touch homogeniser (Bertin Technologies), using the parameters for hard tissue samples (3 × 20 s at 6800 rpm, 30 s pause). Homogenisation was repeated 4 times, with a 5 min incubation on ice after the 2nd cycle. RNA was isolated with the Monarch® Total RNA Miniprep Kit (New England Biolabs) according to the manufacturer’s protocol. Prior isolation, Proteinase K was added to the homogenates and the mixtures were incubated for 5 min at 55 °C. RNA was eluted in nuclease free water and stored until further use at −80 °C. To further remove residual genomic DNA, an in-tube treatment with DNase I was performed using Monarch® DNase I and RNA was purified using the Monarch® RNA Cleanup Kit according to the manufacturer’s instructions (New England Biolabs). Isolated RNA (0.5–1 μg) was reverse transcribed into cDNA using the LunaScript RT Master Mix Kit and d(T)23VN primers according to the manufacturer’s instructions (New England Biolabs). Negative controls lacking the reverse transcriptase (RT) enzyme were performed using the No-RT ControlMix provided by the same manufacturer. One microliter of the cDNA reactions were used as templates for PCR reactions using primers listed in Supplementary Table 3. PCRs of cDNA derived from Bottlenose dolphin tissues were performed with the One Taq 2× Master Mix with Standard Buffer (New England Biolabs) according to the manufacturer’s instructions. PCRs started with an initial denaturation at 94 °C for 30 s, followed by 34 cycles of denaturation for 30 s at 94 °C, annealing at 57 °C for 30 s and extension for 30 s at 68 °C. After a final extension at 68 °C for 5 min, PCR products were mixed with TriTrack loading dye (ThermoFisher, Darmstadt, Germany) and loaded on 2% agarose gels containing 2 µg/100 ml Midori Green (Nippon Genetics, Düren, Germany). PCR amplicons were visualised using a ChemiDoc XRS+ Molecular Imager (Biorad, Feldkirchen, Germany).

Sheep tissues were homogenised with the Precellys Evolution Touch homogeniser, using the parameters for soft tissue samples (15 s at 5600 rpm, 30 s pause, 15 s at 5600 rpm). Homogenisation was repeated four times, with a brief incubation on ice after each cycle. Proteinase K was added to the homogenates and the mixtures were incubated for 20 min at 55 °C. Afterwards, RNA extraction was performed exactly as described above. PCR was performed using Q5® High-Fidelity DNA Polymerase and reactions started with an initial denaturation at 98 °C for 30 s, followed by 30 (gastrointestinal tissues) or 34 (tongue tissue) cycles of denaturation for 10 s at 98 °C, annealing at 69 °C for 30 s and extension at 72 °C for 30 s. After a final extension at 72 °C for 2 min, PCR products were mixed with TriTrack loading loaded on 2% agarose gels containing 2 µg /100 ml Midori Green and PCR amplicons were visualised using a ChemiDoc XRS+ Molecular Imager (Biorad).

All PCR amplicons of appropriate sizes were isolated with the Monarch DNA Extraction Kit (New England Biolabs) and sequenced (Eurofins Genomics, Ebersberg, Germany).

### Behavioural assessment of salt tasting abilities in cetaceans

Behavioural experiments aiming to investigate salt tasting abilities in cetaceans under human care were performed between March and May 2023 at the Oceanogràfic, Ciudad de las Artes y las Ciencias, Valencia, Spain. Experiments were approved by the Oceanogràfic Animal Care & Welfare Committee under the project reference OCE-11-23 and the Generalitat Valenciana under the project reference 2025-VSC-PEA-0040. Experiments to determine the latency of begging behaviour in response to gelatine blocks without or with 500 mM NaCl were performed with 6 adult Bottlenose dolphins (*Tursiops truncatus*), 3 males and 3 females, and 2 Beluga whales (*Delphinapterus leucas*), 1 male and 1 female. Gelatine blocks were prepared by dissolving 100 g gelatine (Promolac, Cornellá del Terri, Spain; Lot. No. 22V2382) with or without 87.7 g of NaCl (Scharlab, Barcelona, Spain) in 1 L of hot deionized water. Afterwards, 2 L of deionized water were added, and the mixture was allowed to solidify over night at 3 °C. This yielded either gelatine with or without 500 mM NaCl. The solidified gelatine was cut into 36 equally sized blocks of 75 g. Each experimental trial started by feeding 3 gelatine blocks at once to one animal. Three blocks were fed simultaneously to enhance the contact time with the oral mucosa. Afterwards, the trainers assumed a neutral position and waited for the animals to display begging behaviour, after which another 3 blocks of gelatine were fed. This procedure was repeated so that each trial consisted of 4 stimuli (2 × 3 gelatine blocks with NaCl; 2 × 3 gelatine blocks without NaCl). The order of stimuli was randomised, and the trainers were blinded. For Bottlenose dolphins, begging behaviour was defined as a clear behavioural response towards the trainer, while the animals had both eyes above the water and the empty mouth was opened (Fig. 5). The same criteria were applied to beluga whales, with the exception that begging behaviour was sometimes displayed with the eyes under water due to the larger size of the animals. The experimental trials were recorded on video and latency times were determined from the time of contact of the gelatine blocks with the oral cavity and the earliest moment begging behaviour was initiated.

To analyse the Bottlenose dolphin data, latency values were logarithmic transformed, and the dataset was modelled with a mixed effects structure in a Bayesian framework, with the following expectation:

$${ln}({latency}) \sim \,	{sex}+{NaCl}+{water}\; {take}\mbox{-}{up}+(1{|individual})\,+\\ 	(1{|session})+({sex}|{trainer})+\varepsilon$$

where *sex* is a separate intercept value for females and males; *NaCl* is the binary factor indicating whether the gelatine block contains *NaCl* or not (absence of NaCl is taken as the reference) with the expectation that it is not statistically distinguishable; *water take-up* is a binary factor indicating whether there is water in the mouth of the cetacean while being fed the block, as this may increase the processing time of the block and therefore increase latency (absence of water is taken as the reference); (1|*individual*) is the random effect of the individual dolphin; (1|*session*) is the random effect of the experiment session, which should account for any bias between days including potential differences in temperature; (*sex*|*trainer*) is the random effect of the trainer, conditional on the sex of the dolphin (i.e. dolphins of different sexes may respond differently to trainers due to social behaviours); and *ϵ* is the residual error that is Gaussian distributed about mean zero. The Bayesian models were run as a single chain with vague priors, for 1,000,000 iterations with a burn-in of 500,000 and a thinning interval of 100, giving a posterior sample size of 5000. Model convergence was checked using the geweke diagnostic which tests for the equality of means between the initial 10% of the final 50% of samples. For the Bottlenose dolphin model, all 28 variables had converged (standard *z*-scores < 2). The models were run in R 4.3.1^67^ using the “MCMCglmm” (v. 2.35)^68^.

The Beluga whale data was modelled in a similar fashion, but as there were only two animals, one of each sex, differences due to sex-based or individual differences are confounded with no replication. Therefore, only individual differences were modelled:

$${ln}({latency}) \sim {individual}+{NaCl}+{water}\, {take}\mbox{-}{up}+(1{|session})+(1{|trainer})+\varepsilon$$

where *individual* is the intercept value separated for the male and female Beluga whale. For the Beluga whale model, all 25 variables had converged (standard *z*-scores < 2), with model setup parameterised the same as the bottlenose dolphin model. Data and statistical models are provided in Supplementary Data 2 and^24^.

### Statistics and reproducibility

#### Sample size

For RT-PCR experiments, RNA was isolated from tissue samples of 3 sheep and 3 Bottlenose dolphins. For animal behaviour data, the sample sizes were not pre-determined statistically, however, the Bayesian mixed effects model revealed significant differences in latency times, indicating that sample sizes were appropriate. A latency measurement was excluded from the analysis when the animal trainer did not remain in neutral position until the animal displayed begging behaviour.

#### Replication

For genomic analyses, all data for a given species that were available in the NCBI databank at the time of the investigation were analysed. Results were confirmed using transcriptomic data available in the NCBI sequence read archive. Accession numbers are provided in Supplementary Table 1 and^24^. For RT-PCR experiments, the number of positive amplicons out of the total number of animals are provided in the figures and all PCR results are provided in ref. ^24^. The animal behaviour (feeding) experiment was repeated over 2 weeks providing 11 or 12 sets of latency measurements (4 feeding stimuli per set) to ensure adequate sample size given logistical constraints. The Bayesian mixed effects model structure accounted for potential differences attributed to feeding day, trainer, and individual animal.

#### Randomisation and blinding

In the animal behaviour (feeding) experiment, the stimulus was randomised based on 4 protocols of random orders of 2 with and 2 without NaCl gelatine blocks fed to cetaceans and trainers who provided the gelatine blocks to the animals were blinded.

#### Statistical analyses

Details on the employed statistical analyses are provided in the corresponding methods section.

### Reporting summary

Further information on research design is available in the Nature Portfolio Reporting Summary linked to this article.

## Supplementary information

Supplementary Information

Supplementary Data 1

Supplementary Data 2

Supplementary Data 3

Description of Additional Supplementary Files

Reporting Summary

## Data Availability

Data are made available in the manuscript, as Supplementary Information, Supplementary Data or as Source Data deposited at the Zenodo data depository^24^, 10.5281/zenodo.15255678.
