# Supplementary material for: The evolutionary path of the epithelial sodium channel δ-subunit in Cetartiodactyla points to a role in sodium sensing
Source: Commun Biol. 2025 Jul 4;8:1004. doi: 10.1038/s42003-025-08436-7 (PMC12227717; doi:10.1038/s42003-025-08436-7)
Supplement: Supplementary file 1 — Supplementary Information [file 42003_2025_8436_MOESM1_ESM.pdf]

Supplementary Information

The evolutionary path of the epithelial sodium channel δ-subunit in Cetartiodactyla points to a role in sodium sensing

Fynn Zahnow[1], Chiara Jäger[1*], Yassmin Mohamed[1*], Gianluca Vogelhuber[1*], Fabian May[1], Alexandra Maria Ciocan[1], Arianna Manieri[1], Stephan Maxeiner[2,3], Gabriela Krasteva-Christ[2,3], Oskar Schnappauf[1], Matthew R. D. Cobain[4], Lars Podsiadlowski[5], José Luis Crespo-Picazo[6], Daniel García-Párraga[6] and Mike Althaus[1]

1 Institute for Functional Gene Analytics, Bonn-Rhein-Sieg University of Applied Sciences, Rheinbach, Germany

2 Institute of Anatomy and Cell Biology, Saarland University, Homburg, Germany

3 Center for Gender-specific Biology and Medicine (CGBM), Saarland University, Homburg, Germany

4 Department of Biological and Environmental Science, University of Jyväskylä, Finland

5 Leibniz Institute for the Analysis of Biodiversity Change (LIB), Bonn, Germany

6 Research Department, Fundación Oceanogràfic de la Comunitat Valenciana, Valencia, Spain

* These authors contributed equally to the study.

\# Correspondence:
Prof. Dr. Mike Althaus
Institute for Functional Gene Analytics
Bonn-Rhein-Sieg University of Applied Sciences
Von-Liebig-Str. 20
53359 Rheinbach, Germany
E-Mail: mike.althaus@h-brs.de

Supplementary Table 1. Employed transcriptomic data from the NCBI Sequence Read Archive (https://www.ncbi.nlm.nih.gov/sra) to validate conclusions derived from comparative genomic analyses (accessed 03-04/2025).

**Species**	**SRA Accession ID**	**Comments**	***Functional SCNN1D* mRNA?**
**Cetacea**			
Globicephala melas	SRX13278362<br>SRX13278363	*SCNN1D:* Splice disruptions in exon 5/6, exons 9/10, exons 10/11 and exons 12/13 confirmed.	No
Lagenorhynchus obliquidens	DRX401349	*SCNN1D:* STOP codon in exon 6 confirmed.	No
Tursiops truncatus	SRR10292058	*SCNN1D:* Disruption of exon/intron structure between exons 2-6 and 8-11.	No
Orcinus orca	SRX18798757	*SCNN1D:* STOP codon in exon 6 confirmed.	No
Phocoena sinus	SRX7696400	*SCNN1D:* STOP codon in exon 6 confirmed.	No
Neophocaena asiaeorientalis	SRX387810	*SCNN1D:* STOP codon in exon 6 confirmed.	No
Delphinapterus leucas	SRX2585929	*SCNN1D:* STOP codon in exon 6 confirmed.	No
Monodon monoceros	SRX5379492	*SCNN1D:* Disruption of exon/intron structure between exons 9-11 confirmed.	No
Pontoporia blainvillei	No SRA data available.		
Inia geoffrensis	No SRA data available.		
Lipotes vexillifer	No SRA data available.		
Hyperoodon ampullatus	ERX11235623	*SCNN1D:* Only 8 reads in total (aligning to exons 8/9). STOP codon in exon 6 confirmed.	No
Mesoplodon bidens	ERX1403318	*SCNN1D:* No reads aligning to putative gene.	Insufficient data
	ERX1403315	*SCNN1D:* No reads aligning to putative gene.	
	ERX12111221	*SCNN1D:* No reads aligning to putative gene.	
Ziphius cavirostris	No SRA data available.		
Platanista gangetica	No SRA data available.		
Platanista minor	No SRA data available.		
Kogia breviceps	SRX24338781	*SCNN1D:* STOP codon in exon 6 confirmed.	No
Physeter macrocephalus (catodon)	SRX9474695	*SCNN1D:* STOP codon in exon 4 confirmed.	No
Balaenoptera musculus	SRX7696402	*SCNN1D:* Splice donor flanking exon 7 identified (causing additional 4 bp deletion in exon 7). No reads aligning to mRNA reconstructed from exons 8-12.	No
Balaenoptera acutorostrata scammony	DRX523282	*SCNN1D:* No reads aligning to mRNA reconstructed from exons 6-11.	No
	DRX523281	*SCNN1D:* Only 6 reads in total. No reads aligning to mRNA reconstructed from exons 3-5.	
	DRX523280	*SCNN1D:* Evidence for splice disruption following exon 5 and exon 10.	
Eubalaena japonica	No SRA data available.		
**Artiodactyla**			
Hippopotamus amphibius	SRX2880553	*SCNN1D:* Confirmed correct sequence and splicing of exons 1-6; exons 7-8 and exons 10-13.	Yes
	SRX5087252	*SCNN1D:* Confirmed correct sequence and splicing of exons 6-7 and exons 8-10.	
Tragulus javanicus	SRX7585805	*SCNN1D:* Confirmed correct sequence and splicing of exons 1-2; exons 3-7 and exons 8-13.	Yes
	SRX7585806	*SCNN1D:* Confirmed correct sequencing splicing exons 2-3 (weak evidence) and exons 6-8.	
Tragulus kanchil	No SRA data available.		
Antilocapra americana	SRX22085502	*SCNN1D:* Confirmed correct sequence and splicing of exons 2-10; no reads in dataset aligning to exons 11-13.	Partial confirmation
Giraffa camelopardalis	SRX2880540	*SCNN1D*: Insufficient data – only 10 reads partially aligning to putative coding sequence.	Insufficient data
Giraffa tippelskirchi	No SRA data available.		
Capreolus pygargus	No SRA data available.		

Cervus elaphus	SRX12706346	*SCNN1D*: Insufficient data – only 3 reads partially aligning to putative coding sequence.	Insufficient data but majority of mRNA confirmed
	SRX12706345	*SCNN1D*: Insufficient data – only 12 reads partially aligning to putative coding sequence. Confirmed correct sequence and splicing of exons 7-8.	
	SRX12706344	*SCNN1D*: Insufficient data – only 2 reads partially aligning to putative coding sequence.	
	SRX2880481	*SCNN1D*: Insufficient data – only 10 reads partially aligning to putative coding sequence. Confirmed correct sequence and splicing of exons 6-7; exons 8-11 and exons 12-13.	
	SRX1060418	*SCNN1D*: Insufficient data – only 16 reads partially aligning to putative coding sequence. Confirmed correct sequence and splicing of exons 4-7; and exons 11 (weak)-13.	
Moschus moschiferus	SRX6924371	*SCNN1D*: Insufficient data – only 2 reads partially aligning to putative coding sequence.	Insufficient data
	SRX2880563	*SCNN1D*: Insufficient data – only 3 reads partially aligning to putative coding sequence.	
Moschus berezovskii	SRX19406986	*SCNN1D*: Confirmed correct sequence and splicing of exons 1-6. No reads covering exons 11-12.	Majority of mRNA confirmed
	SRX13455934	*SCNN1D*: Insufficient data – only 17 reads partially aligning to putative coding sequence. Confirmed correct sequence and splicing of exons 6-11 and exons 12-13. No reads covering exons 11-12.	
	SRX1131841	*SCNN1D*: No reads covering exons 11-12.	
Bos grunniens	SRX18971843	*SCNN1D*: Confirmed correct sequence and splicing of exons 2-13.	Yes
Bos taurus	SRX17564658	*SCNN1D*: Confirmed correct sequence and splicing of exons 2-13.	Yes
Bubalus bubalis	ERX2403575	*SCNN1D*: Confirmed correct sequence and splicing of exons 2-13.	Yes
Nanger granti	No SRA data available.		
Kobus leche	No SRA data available.		
Capra hircus	SRX24936814	*SCNN1D:* Confirmed correct sequence and splicing of exons 2-13, *including exon 11/12 fusion.*	Yes
Ovis aries	SRX20677076	*SCNN1D:* Confirmed correct sequence and splicing of exons 1-13, *including exon 11/12 fusion.*	Yes
Ovis canadensis	SRX25644109<br>SRX25644107	*SCNN1D:* Confirmed correct sequence and splicing of exons 2-13, *including exon 11/12 fusion.*	Yes
Oreamnos americanus	No SRA data available.		
Hippotragus niger	No SRA data available.		
Damaliscus lunatus	No SRA data available.		
**Suidae**			
Sus scrofa	SRX21159113	*SCNN1D*: Confirmed correct sequence and splicing of exons 1-2 and exons 3-13.	Yes
	SRX24362122	*SCNN1D*: Confirmed correct sequence and splicing of exons 2-3.	
**Camelidae**			
Vicugna pacos	SRX7048576	*SCNN1D*: Confirmed correct sequence and splicing of exons 2-3.	Yes
**Equidae**			
Equus caballus	ERX3441445	*SCNN1D*: Confirmed correct sequence and splicing of exons 1-9 and exons 10-13.	Yes
	ERX3439279	*SCNN1D*: Confirmed correct sequence and splicing of exons 9-10.	
**Musteloidea**			
Taxidea taxus	No SRA data available.		
Eira barbara	No SRA data available.		
Gulo gulo luscus	No SRA data available.		

Martes flavigula	SRX17463780	*SCNN1D*: Confirmed correct sequence and splicing of exons 1-2.	Majority of mRNA confirmed
	SRX17463779	*SCNN1D*: Confirmed correct sequence and splicing of exons 4-6.	
	SRX17463783	*SCNN1D*: Confirmed correct sequence and splicing of exons 9-13.	
	SRX17463784	*SCNN1D*: Confirmed correct sequence and splicing of exons 6-9.	
Pteronura brasiliensis	No SRA data available.		
Enhydra lutris kenyoni	No SRA data available.		
Enhydra lutris nereis	No SRA data available.		
Lontra canadensis	No SRA data available.		
Mustela nivalis	ERX13203403	*SCNN1D*: Insufficient data – no reads aligning to putative coding sequence.	Insufficient data
Mustela putorius furo	SRX3316193	*SCNN1D*: Confirmed correct sequence and splicing of exons 1-13 (no reads confirming splicing after exon 3-4).	Yes
	SRX114308	*SCNN1D:* Reads confirming splicing after exon 3-4.	
Mustela erminea	SRX18173626	*SCNN1D*: Confirmed correct sequence and splicing of exons 2-3, exons 5-6 and exons 10-13.	Majority of mRNA confirmed
	ERX13203410	*SCNN1D*: Confirmed correct sequence and splicing of exons 6-7, exons 8-10.	
	SRX18173627	*SCNN1D*: Confirmed correct sequence and splicing of exons 7-9 (including alternative splice donor following exon 7).	
Neogale vison	SRX18955290	*SCNN1D*: Confirmed correct sequence and splicing of exons 1-13 (weak evidence for alternative splicing after exon 11).	Majority of mRNA confirmed
Meles meles	ERX12465321	*SCNN1D*: Confirmed correct sequence and splicing of exons 2-13.	Yes
Mellivora capensis	No SRA data available.		
**Pinnipedia**			
Odobenus rosmarus divergens	No SRA data available.		
Callorhinus ursinus	SRX14129725	*SCNN1D:* Insufficient data – only 20 reads aligning to putative coding sequence.	Insufficient data
Arctocephalus townsendi	No SRA data available.		
Arctocephalus gazella	SRX1619239	***SCNN1G***: Confirmed suspected sequencing error in *SCNN1G* exon 6.	not applicable
	SRX1619239	*SCNN1D*: Confirmed correct sequence and splicing of exons 1-5, exons 7-9 and exons 11-13.	Yes
Eumetopias jubatus	SRX2987959	*SCNN1D*: Confirmed correct sequence and splicing of exons 3-13.	Yes
	SRX2987957	*SCNN1D*: Confirmed correct sequence and splicing of exons 2-3.	
Zalophus californianus	SRX4928796	*SCNN1D*: Confirmed correct sequence and splicing of exons 1-13.	Yes
Haliocherus grypus	No SRA data available.		
Pusa hispida saimensis	SRX14129726	*SCNN1D*: Confirmed correct sequence and splicing of exons 2-7 and 8-13.	Majority of mRNA confirmed
Phoca vitulina	No SRA data available.		
Leptonychotes weddellii	SRX5972987	*SCNN1D:* Confirmed STOP codon in exon 4.	No
Mirounga leonina	No SRA data available.		
Mirounga angustirostris	SRX20833365	*SCNN1D:* Confirmed STOP codon in exon 6.	No
Neomonachus schauinslandi	No SRA data available.		
**Ursidae**			
Ailuropoda melanoleuca	SRX1208772<br>SRX4475882<br>SRX22906964	*SCNN1D*: Confirmed correct sequence and splicing of exons 2-13.	Yes
Tremarctos ornatus	SRX9996964	*SCNN1D:* Insufficient data – only 13 reads aligning to putative coding sequence.	Insufficient data
Ursus tibetanus japonicus	No SRA data available.		

Ursus americanus	No SRA data available.	No reads aligning to *SCNN1D* in the available tissue samples.	Insufficient data
Helarctos malayanus	No SRA data available.		
Ursus arctos horribilis	No SRA data available.	No reads aligning to *SCNN1D* in the available tissue samples.	Insufficient data
Ursus maritimus	No SRA data available.	No reads aligning to *SCNN1D* in the available tissue samples.	Insufficient data
**Afrotheria**			
Heterohyrax brucei	No SRA data available.		Insufficient data
Procavia capensis	No SRA data available.		Insufficient data
Dugon dugon	SRX24708567	*SCNN1D:* Spice donor following exon 12 identified.	Insufficient data
Trichechus manatus latirostris	No SRA data available.		Insufficient data
Loxodonta africana	SRR11484176	No reads aligning to exons 2-7. Alternative splice donor flanking exon 12 identified.	No
Elephas maximus	SRR30223437	No reads aligning to exons 2-7. Alternative splice donor flanking exon 12 identified.	No
Chrysochloris asiatica	No SRA data available.	*SCNN1D:* STOP codon in exon 4 in all reading frames based on genomic data.	No
Nesogale talazaci	No SRA data available.		Insufficient data
Echinops telfairi	No SRA data available.	*SCNN1D*: STOP codon in exon 4 in all reading frames based on genomic data.	No
Elephantulus edwardii	No SRA data available.		Insufficient data
Orycteropus afer afer	No SRA data available.	*SCNN1D*: STOP codon in exon 4 in all reading frames based on genomic data.	No
**Xenarthra**			
Tolypeutes matacus	No SRA data available.		
Cabassous unicinctus	Genomic information for *SCNN1D* incomplete		
Dasypus novemcinctus	SRX8937358	*SCNN1D*: Confirmed correct sequence and splicing of exons 1-13.	Yes
Tamandua tetradactyla	Genomic information for *SCNN1D* incomplete		
Choloepus didactylus	Genomic information for *SCNN1D* incomplete		

Supplementary Table 2. Employed online tools and resources in this study.

Resource	Weblink	Access period (month/year)
NCBI BLAST	https://blast.ncbi.nlm.nih.gov/Blast.cgi	01/2022-04/2025
Multalin	http://multalin.toulouse.inra.fr/multalin/	01/2022-04/2025
Expasy Translate Tool	https://web.expasy.org/translate/	01/2022-04/2025
Clustal Omega v1.2.4	https://www.ebi.ac.uk/Tools/msa/clustalo/	01/2022-04/2024
Timetree	http://timetree.org	11/2023-04/2025
ColabFold v1.5.5	https://colab.research.google.com/github/sokrypton/ColabFold/blob/main/Colabfold.ipynb	01/2024-04/2024
NCBI SRA	https://www.ncbi.nlm.nih.gov/sra	02/2025–04/2025
Alternative Splice Site Predictor	http://wangcomputing.com/assp/	02/2025–04/2025
NCBI Genome Repository	https://ftp.ncbi.nlm.nih.gov/genomes/	04/2025

Supplementary Table 3. Employed primers for RT-PCR reactions.

Target	Species	Primer sequence (5'-3')		Amplicon size (bp)	
				mRNA	genomic
ACTB	*T. truncatus*	Forward	TGGCATCCACGAAACTACCT	202	358
		Reverse	CACGGAGTACTTGCGTTCAG		
SCNN1A	*T. truncatus*	Forward	AGGAAGCATAATTCCTGGGGC	299	3645
		Reverse	AGGGTGACCATCGTGACAGA		
SCNN1B	*T. truncatus*	Forward	TGAAGTTGATCCTGGACGTGG	240	553
		Reverse	TGTAGAGGTTTCGGATGGGGA		
SCNN1G	*T. truncatus*	Forward	GAAGGGCACAGGGTCGAAAT	358	2039
		Reverse	CTCCCCATCAAAGAAGCAGGT		
SCNN1D	*O. aries*	Forward	TACAAGCTTTCTGCCGGGAC	377/278*	620
		Reverse	AGCTCTAGCACCTCCACGA		

* For *O. aries SCNN1D*, primers amplify the sequences between exons 10 and 13. The amplicon size therefore depends on the presence of the exon 11/12 fusion and incorporation of the former intron.

Supplementary Alignment 1

α-ENaC (*SCNN1A*) amino acid sequence alignment created with Clustal Omega (v.1.2.4, accessed 24.03.2024). Key structural motifs are highlighted in the human ENaC subunit based on the Cryo-EM derived structure[1]. Transmembrane domains (TM1/TM2) are highlighted in yellow. An N-Terminal HG-motif affecting ENaC open probability is marked in magenta. Protease cleavage sites are highlighted in blue. Cysteines involved in tertiary structure formation are indicated in red. Amino acids contributing to sodium ion binding in the cation binding pocket[2] are shown in dark blue. Residues putatively forming the selectivity filter within TM2 are shown in red font. The C-terminal PPPxY motif regulating membrane abundance is shown in **gray**.

```
alpha-ENaC_Homo_sapiens                       MEGNKLEEQDSSPPQSTPGLMKGNKREEQGLGPEPAAPQQPTAEEEALIEFHRSYRELFE  60
alpha-ENaC_Globicephala_melas                 --------------------MKGDKHEEPEPGPEPAAPSPSTDEEEPLLEFHHSYRELFQ  40
alpha-ENaC_Lagenorhynchus_obliquidens         --------------------MKGDKHEEPEPGPEPAAPPPSTDEEEPLLEFHHSYRELFQ  40
alpha-ENaC_Tursiops_truncatus                 --------------------MKGDKHEEPEPGPEPAAPPPSTDEEEPLLEFHHSYRELFQ  40
alpha-ENaC_Orcinus_orca                       --------------------MKGDKHEEPEPGPEPAAPPPSTDEEEPLLEFHHSYRELFQ  40
alpha-ENaC_Phocoena_sinus                     --------------------MKGDKHEEPEPGPEPAAPPPSTDEEEPLLEFHHSYRELFQ  40
alpha-ENaC_Neophocaena_asiaeorientalis        --------------------MKGDKHEEPEPGPEPAAPPPSTDEEEPLLEFHHSYRELFQ  40
alpha-ENaC_Monodon_monocerons                 --------------------MKGDKHEEPEPGPEPAAPPPSTDEEEPLLEFHHSYRELFQ  40
alpha-ENaC_Delphinapterus_leucas              --------------------MKGDKHEEPEPGPEPAAPPPSTDEEEPLLEFHHSYRELFQ  40
alpha-ENaC_Pontoporia_blainvillei             --------------------MKGDKREEPEPGPEPAAPPPSTDEEEALLEFHDSYRELFQ  40
alpha-ENaC_Inia_geoffrensis                   --------------------MKGDKREEPEPGPEPAAPPPSTDEEEALLEFHHSYRELFQ  40
alpha-ENaC_Mesoplodon_bidens                  --------------------MKGDKREEPEPGPEPASPPPSTDEEEALLEFHHSYRELFQ  40
alpha-ENaC_Ziphius_cavirostris                --------------------MKGDKREEPEPGPEPAAPPPSTDEEEALLEFHHSYRELFQ  40
alpha-ENaC_Platanista_gangetica               --------------------MKGDKREEPEPGPEPAAPPPPTDEEEALLEFHHSYRELFQ  40
alpha-ENaC_Platanista_minor                   --------------------MKGDKREEPEPGPEPAAPPPPTDEEEALLEFHHSYRELFQ  40
alpha-ENaC_Kogia_breviceps                    --------------------MKGDKREEPEAGPEPAAPAPPMDEEEALLEFHHSYRELFQ  40
alpha-ENaC_Physeter_catodon                   --------------------MKGDEREEPEAGPEPAAPPPPADEQEALLEFHHSYRELFQ  40
alpha-ENaC_Balaenoptera_musculus              --------------------MKGDKREEPEPGPEPAAPPPPTEEEEALLEFHHSYRELFQ  40
alpha-ENaC_Eubalaena_japonica                 --------------------MKGDKREEPEPGPEPAAAPPPTEEEEALLEFHHSYRELFQ  40
alpha-ENaC_Hippopotamus_amphibius             --------------------MKEDKREEPGPGPEPEAPPPPTEEEEALLEFHRSYRELFE  40
alpha-ENaC_Tragulus_javanicus                 --------------------MKGDKREEPGPGPEPAAPPRPTEDEEALLEFHRSYRELFE  40
alpha-ENaC_Antilocapra_americana              --------------------MKGDKPEEPGPGPEPAGPPPRTEEEEALLEFHRSYRELFE  40
alpha-ENaC_Giraffa_camelopardalis             --------------------MKGDKPEEPGLGPEPSGPLPRTEE-EALLEFHRSYRELFE  39
alpha-ENaC_Giraffa_tippelskirchi              --------------------MKGDKPEEPGLGPEPSGPLPRTEE-EALLEFHRSYRELFE  39
alpha-ENaC_Capreolus_pygargus                 --------------------MKGDKPEEPGPGPEPSGPPPPTEEEEALLEFHRSYRELFE  40
alpha-ENaC_Cervus_elaphus                     --------------------MKGDKPEEPGQGPEPSGPPPPTEEEEALLEFHRSYRELFE  40
alpha-ENaC_Moschus_moschiferus                --------------------MKGDKPEELGPGPEPSGPPPPTEEEEALLEFHRSYRELFE  40
alpha-ENaC_Moschus_berezovskii                --------------------MKGDKPEELGPGPEPSGPPPPTEEEEALLEFHRSYRELFE  40
alpha-ENaC_Bos_grunniens                      --------------------MKGDKPEEPGPGPEPSGPPPPTEEEEALLEFHRSYRELFE  40
alpha-ENaC_Bos_taurus                         --------------------MKGDKPEEPGPGPEPSGPPPPTEEEEALLEFHRSYRELFE  40
alpha-ENaC_Bubalus_bubalis                    --------------------MKGDKPEEPGPGPEPSGPLPPTEEEEALLEFHRSYRELFE  40
alpha-ENaC_Nanger_granti                      --------------------MKGDKPEEPGPGPETSGPPPPTEEEEALLEFHRSYRELFE  40
alpha-ENaC_Capra_hircus                       --------------------MKGDKREELGPGPEPSGPLPPTEEEEALLEFHRSYRELFE  40
alpha-ENaC_Ovis_aries                         --------------------MKGDKREELGPGPEPSAPPLPTEEEEALLEFHRSYRELFE  40
alpha-ENaC_Ovis_canadensis                    --------------------MKGDKREELGPGPEPSAPPPPTEEEEALLEFHRSYRELFE  40
alpha-ENaC_Hippotragus_niger                  --------------------MKGDKPEEPGPGSEPSGPPQPTEEEEALLEFHRSYRELFE  40
alpha-ENaC_Damaliscus_lunatus                 --------------------MKGDKPEEPGPGPEPSGPPPPTEEEEALLEFHRSYRELFE  40
alpha-ENaC_Sus_scrofa                         --------------------MKGDKREEPGPGPEPTAPPLPTEEEEALLEFHRSYRELFQ  40
alpha-ENaC_Vicugna_pacos                      --------------------MKGDKREEPGPDPEPAAPQQPTEEEEALIEFHRSYRELFE  40
alpha-ENaC_Equus_callabus                     --------------------MKGDKHEEQELGSEPTAPQQPTEEEEALIEFHRSYRELFE  40
                                                                  ** :: **   . *  .      : * *:*** ******:

                                                                       TM1
alpha-ENaC_Homo_sapiens                       FFCNNTTIHGAIRLVCSQHNRMKTAFWAVLWLCTFGMMYWQFGLLFGEYFSYPVSLNINL  120
alpha-ENaC_Globicephala_melas                 FFCNNTTIHGAIRLVCSQHNRMKTAFWAVLWLCTFGMMYWQFGLLFGEYFSYPVSLNINL  100
alpha-ENaC_Lagenorhynchus_obliquidens         FFCNNTTIHGAIRLVCSQHNRMKTAFWAVLWLCTFGMMYWQFGLLFGEYFSYPVSLNINL  100
alpha-ENaC_Tursiops_truncatus                 FFCNNTTIHGAIRLVCSQHNRMKTAFWAVLWLCTFGMMYWQFGLLFGEYFSYPVSLNINL  100
alpha-ENaC_Orcinus_orca                       FFCNNTTIHGAIRLVCSQHNRMKTAFWAVLWLCTFGMMYWQFGLLFGEYFSYPVSLNINL  100
alpha-ENaC_Phocoena_sinus                     FFCNNTTIHGAIRLVCSQHNRMKTAFWAVLWLCTFGMMYWQFGLLFGDYFSYPVSLNINL  100
alpha-ENaC_Neophocaena_asiaeorientalis        FFCNNTTIHGAIRLVCSQHNRMKTAFWAVLWLCTFGMMYWQFGLLFGDYFSYPVSLNINL  100
alpha-ENaC_Monodon_monocerons                 FFCNNTTIHGAIRLVCSQHNRMKTAFWAVLWLCTFGMMYWQFGLLFGEYFSYPVSLNINL  100
alpha-ENaC_Delphinapterus_leucas              FFCNNTTIHGAIRLVCSQHNRMKTAFWAVLWLCTFGMMYWQFGLLFGEYFSYPVSLNINL  100
alpha-ENaC_Pontoporia_blainvillei             FFCNNTTIHGAIRLVCSQHNRMKTAFWAVLWLCTFGMMYWQFGLLFGEYFSYPVSLNINL  100
alpha-ENaC_Inia_geoffrensis                   FFCSNTTIHGAIRLVCSQHNRMKTAFWAVLWLCTFGMMYWQFGLLFGEYFSYPVSLNINL  100
alpha-ENaC_Mesoplodon_bidens                  FFCNNTTIHGAIRLVCSQHNRMKTAFWAVLWLCTFGMMYWQFGLLFGEYFSYPVSLNINL  100
alpha-ENaC_Ziphius_cavirostris                FFCNNTTIHGAIRLVCSQHNRMKTAFWAVLWLCTFGMMYWQFGLLFGEYFSYPVSLNINL  100
alpha-ENaC_Platanista_gangetica               FFCSNTTIHGAIRLVCSQHNRMKTAFWAVLWLCTFGMMYWQFGLLFGEYFSYPVSLNINL  100
alpha-ENaC_Platanista_minor                   FFCSNTTIHGAIRLVCSQHNRMKTAFWAVLWLCTFGMMYWQFGLLFGEYFSYPVSLNINL  100
alpha-ENaC_Kogia_breviceps                    FFCNNTTIHGAIRLVCSQHNRMKTAFWAVLWLCAFSMMYWQFGLLFGEYFSYPVSLNINL  100
alpha-ENaC_Physeter_catodon                   FFCSNTTIHGAIRLVCSRHNRMKTAFWAALWLCAFGMMYWQFGLLFGEYFSYPVSLNINL  100
alpha-ENaC_Balaenoptera_musculus              FFCNNTTIHGAIRLVCSQHNRMKTAFWAVLWLCTFGMMYWQFGLLFGEYFSYPVSLNINL  100
alpha-ENaC_Eubalaena_japonica                 FFCNNTTIHGAIRLVCSQHNRMKTAFWAVLWLCTFGMMYWQFGLLFGEYFSYPVSLNINL  100
alpha-ENaC_Hippopotamus_amphibius             FFCNNTTIHGAIRLVCSQHNRMKTAFWAVLWLCTFGMMYWQFGLLFGEYFSYPVSLNINL  100
alpha-ENaC_Tragulus_javanicus                 FFCNNTTIHGAIRLVCSRHNRMKTAFWAVLWLCTFGMMYWQFGQLFGEYFSYPVSLNINL  100
alpha-ENaC_Antilocapra_americana              FFCNNTTIHGAIRLVCSQHNRMKTAFWAVLWLCTFGMMYWQFGQLFGEYFSYPVSLNINL  100
alpha-ENaC_Giraffa_camelopardalis             FFCNNTTIHGAIRLVCSQHNRMKTVFWAVLWLCTFGMMYWQFGQLFGEYFSYPVSLNINL  99
```

```
alpha-ENaC_Giraffa_tippelskirchi             FFCNNTTIHGAIRLVCSQHNRMKTVFWAVLWLCTFGMMYWQFGQLFGEYFSYPVSLNINL  99
alpha-ENaC_Capreolus_pygargus                FFCNNTTIHGAIRLVCSQHNRMKTVFWAVLWLCTFGMMYWQFGQLFGEYFSYPVNLNINL  100
alpha-ENaC_Cervus_elaphus                    FFCNNTTIHGAIRLVCSQHNRMKTVFWAVLWLCTFGMMYWQFGQLFGEYFSYPVSLNINL  100
alpha-ENaC_Moschus_moschiferus               FFCNNTTIHGAIRLVCSQHNRMKTVFWAVLWLCTFGMMYWQFGQLFGEYFSYPVSLNINL  100
alpha-ENaC_Moschus_berezovskii               FFCNNTTIHGAIRLVCSQHNRMKTVFWAVLWLCTFGMMYWQFGQLFGEYFSYPVSLNINL  100
alpha-ENaC_Bos_grunniens                     FFCNNTTIHGAIRLVCSQHNRMKTVFWAVLWLCTFGMMYWQFGQLFGEYFSYPVSLNINL  100
alpha-ENaC_Bos_taurus                        FFCNNTTIHGAIRLVCSQHNRMKTVFWAVLWLCTFGMMYWQFGQLFGEYFSYPVSLNINL  100
alpha-ENaC_Bubalus_bubalis                   FFCNNTTIHGAIRLVCSQHNRMKTVFWAVLWLCTFGMMYWQFGQLFGEYFSYPVSLNINL  100
alpha-ENaC_Nanger_granti                     FFCNNTTIHGAIRLVCSQHNRMKTVFWAVLWLCTFGMMYWQFGQLFGEYFSYPVSLNINL  100
alpha-ENaC_Capra_hircus                      FFCNNTTIHGAIRLVCSQHNRMKTVFWAVLWLCTFGMMYWQFGQLFGEYFSYPVSLNINL  100
alpha-ENaC_Ovis_aries                        FFCNNTTIHGAIRLVCSQHNRMKTVFWAVLWLCTFGMMYWQFGQLFGEYFSYPVSLNINL  100
alpha-ENaC_Ovis_canadensis                   FFCNNTTIHGAIRLVCSQHNRMKTVFWAVLWLCTFGMMYWQFGQLFGEYFSYPVSLNINL  100
alpha-ENaC_Hippotragus_niger                 FFCNNTTIHGAIRLVCSQHNRMKTVFWAVLWLCTFGMMYWQFGQLFGEYFSYPVSLNINL  100
alpha-ENaC_Damaliscus_lunatus                FFCNNTTIHGAIRLVCSQHNRMKTVFWAVLWLCTFGMMYWQFGQLFGEYFSYPVSLNINL  100
alpha-ENaC_Sus_scrofa                        FFCNNTTIHGAIRLVCSQHNRMKTAFWAVLWLCTFGMMYWQFGLLFEEYFSYPVSLNINL  100
alpha-ENaC_Vicugna_pacos                     FFCNNTTIHGAIRLVCSQHNRMKTAFWAVLWLCTFGMMYWQFGLLFQEYFSYPVSLNINL  100
alpha-ENaC_Equus_callabus                    FFCNHTTIHGAIRLVCSQHNRMKTAFWAVLWLCTFGMMYWQFGLLFGEYFSYPVSLNINL  100
                                             ***.:************:******.***.****:*.******* ** :******.*****

alpha-ENaC_Homo_sapiens                      NSDKLVFPAVTICTLNPYRYPEIKEELEELDRITEQTLFDLYKYSSFTTLVAGSRSRRDL  180
alpha-ENaC_Globicephala_melas                NSEKLVFPAVTVCTLNPYRYTEMKKDLEELDRTTEQTLFDLYKYNSSNSLVAHARGRRDL  160
alpha-ENaC_Lagenorhynchus_obliquidens        NSEKLVFPAVTVCTLNPYRYTEMKKDLEELDRTTEQTLFDLYKYNSSNSLVAHARGRRDL  160
alpha-ENaC_Tursiops_truncatus                NSEKLVFPAVTVCTLNPYRYTEMKKDLEELDRTTEQTLFDLYKYNSSNSLVAHARGRRDL  160
alpha-ENaC_Orcinus_orca                      NSEKLVFPAVTVCTLNPYRYTEMKKDLEELDRTTEQTLFDLYKYNSSNSLVAHARGRRDL  160
alpha-ENaC_Phocoena_sinus                    NSEKLVFPAVTVCTLNPYRYTEMKKDLEELDRITEQTLFDLYKYNSSNTLVAHARGRRDL  160
alpha-ENaC_Neophocaena_asiaeorientalis       NSEKLVFPAVTVCTLNPYRYTEMKKDLEELDRITEQTLFDLYKYNSSNTLVAHARGRRDL  160
alpha-ENaC_Monodon_monocerons                NSEKLVFPAVTVCTLNPYRYTEMKKDLEELDRITEQTLFDLYKYNSSNTLVAHARGRRDL  160
alpha-ENaC_Delphinapterus_leucas             NSEKLVFPAVTVCTLNPYRYTEMKKDLEELDRITEQTLFDLYKYNSSNTLVAHARGRRDL  160
alpha-ENaC_Pontoporia_blainvillei            NSEKLVFPAVTVCTLNPYRYTEMKKDLEELDRITEQTLFGLYKYNSSNTLVAHARGRRDL  160
alpha-ENaC_Inia_geoffrensis                  NSEKLVFPAVTVCTLNPYRYTEMKKDLEELDRITQTQTLFDLYEYNSSNTLVAHARGRRDL  160
alpha-ENaC_Mesoplodon_bidens                 NSEKLVFPAVTVCTLNPYRYTEIKGVLEELDQITEQTLFDLYKYNSSNTLVAHARGRRDL  160
alpha-ENaC_Ziphius_cavirostris               NSEKLVFPAVTVCTLNPYRYTEIKGELEELDQVTEQTLFDLYKYNSSNTLVAHARGRRDL  160
alpha-ENaC_Platanista_gangetica              NSEKLVFPAVTVCTLNPYRYTEIKEELEELDRITEQTLFDLYKYNSSNTLVAHARGRRDL  160
alpha-ENaC_Platanista_minor                  NSEKLVFPAVTVCTLNPYRYTEIKEELEELDRITEQTLFDLYKYNSSNTLVAHARGRRDL  160
alpha-ENaC_Kogia_breviceps                   NSEKLVFPAVTVCTLNPYRYTEIKEELEELDRITEQTLFDLYKYNSSNTLVAHARGRRDL  160
alpha-ENaC_Physeter_catodon                  NSEKLVFPAVTVCTLNPYRYTEIKEELEELDRITEQTLFDLYKYNSSNTLVAHARGRRDL  160
alpha-ENaC_Balaenoptera_musculus             NSEKLVFPAVTVCTLNPYRYTEIKEELEELDRITEQTLFDLYKYNSSNTLVAHARGRRDL  160
alpha-ENaC_Eubalaena_japonica                NSDKLVFPAVTVCTLNPYRYTEIKEELEELDRITEQTLFDLYKYNSSNTLVAHARGRRDL  160
alpha-ENaC_Hippopotamus_amphibius            NSDKLVFPAVTVCTLNPYRYKEIKEELEELDRITEQTLFDLYKYNSSNTLVAHARGRRDL  160
alpha-ENaC_Tragulus_javanicus                NSDKLVFPAVSICTLNPYRYKEIQDELEELDRITEQTLFDLYKYNSSNTLTAHARPRRDL  160
alpha-ENaC_Antilocapra_americana             NSDKLVFPAVSICTLNPYRYKEIQEELEELDRITEQTLFDLYKYNSSNTLVASARSRRDL  160
alpha-ENaC_Giraffa_camelopardalis            NSDKLIFPAVSICTLNPYRYKEIQEELEELDRITEQTLFDLYKYNSSNTLVA--RSRRDL  157
alpha-ENaC_Giraffa_tippelskirchi             NSDKLIFPAVSICTLNPYRYKEIQEELEELDRITEQTLFDLYKYNSSNTLVA--RSRRDL  157
alpha-ENaC_Capreolus_pygargus                NSDKLIFPAVSICTLNPYRYKEIQEELEELDRITEQTLFDLYEYNSSSSLVARARARRAL  160
alpha-ENaC_Cervus_elaphus                    NSDKLIFPAVSICTLNPYRYKEIQEELEELDRITEQTLFDLYEYNSSNTLVAHARSRRDL  160
alpha-ENaC_Moschus_moschiferus               NSDKLIFPAVSICTLNPYRYKDIQEELEELDRITEQTLFDLYKYNSSNSLVAHARSRREL  160
alpha-ENaC_Moschus_berezovskii               NSDKLIFPAVSICTLNPYRYKDIQEELEELDRITEQTLFDLYKYNSSNSLVAHARSRREL  160
alpha-ENaC_Bos_grunniens                     NSDKLVFPAVSICTLNPYRYKEIQEELEELDRITEQTLFDLYKYNSSKTLVAHARSRRDL  160
alpha-ENaC_Bos_taurus                        NSDKLVFPAVSICTLNPYRYKEIQEELEELDRITEQTLFDLYKYNSSKTLVAHARSRRDL  160
alpha-ENaC_Bubalus_bubalis                   NSDKLVFPAVSICTLNPYRYKEIQEELEELDRITEQTLFDLYKYNSSNTLVAHARSRRDL  160
alpha-ENaC_Nanger_granti                     NSDKLIFPAVSICTLNPYRYKEIQEELEDLDRITEQTLFDLYKYNASHTLVAHARSRRDL  160
alpha-ENaC_Capra_hircus                      NSDKLIFPAVSICTLNPYRYKEIQEELEDLDRITEQTLFDLYKYNASHTLVAHARSRRDL  160
alpha-ENaC_Ovis_aries                        NSDKLIFPAVSICTLNPYRYKEIQEELEDLDRITEQTLFDLYKYNASHTLVAHARSRRDL  160
alpha-ENaC_Ovis_canadensis                   NSDKLIFPAVSICTLNPYRYKEIQEELEDLDRITEQTLFDLYKYNASHTLVAHARSRRDL  160
alpha-ENaC_Hippotragus_niger                 NSDKLIFPAVSICTLNPYRYKEIQEELEDLDRITEQTLFDLYKYNSSHTLVAHARSRRDL  160
alpha-ENaC_Damaliscus_lunatus                NSDKLIFPAVSICTLNPYRYKEIQEELEDLDRITEQTLFDLYKYNSSHTLVAHARSRRDL  160
alpha-ENaC_Sus_scrofa                        NSDKLVFPAVTICTLNPYRYKEIKEELEELDRITEQTLFDLYKYNSSNTLVAHARLRRDL  160
alpha-ENaC_Vicugna_pacos                     NSDKLVFPAVSICTLNPYRYTEIKEELEELDRITEQTLFDLYKYNSSNTLVARARGRRDL  160
alpha-ENaC_Equus_callabus                    NSDKLVFPAVTICTLNPYRYAKIKEELEELDRITEQTLYDLYKYNFSNTLVAHPRGRRDL  160
                                             **:**:****::******** .::  **:**: *:***:.**:*.   :*.*  * ** *

alpha-ENaC_Homo_sapiens                      RGTLPHPLQRLRVPPPPHGARRARSVA-SSLRDNNPQVDWKDWKIGFQLCNQNKSDCFYQ  239
alpha-ENaC_Globicephala_melas                RQSLPHPLQRLPVPAPPHAASRVRRSD-SSLSHSNPKVNRKDWKIGFQLCNQNNSDCFYR  219
alpha-ENaC_Lagenorhynchus_obliquidens        RESLPHPLQRLPVPAPPHAASRVRRSD-SSLSHSNPKVNRKDWKIGFQLCNQNNSDCFYR  219
alpha-ENaC_Tursiops_truncatus                RESLPHPLQRLPVPAPPHAASRVRRSD-SSLSHSNPKVNRKDWMIGFQLCNQNNSDCFYR  219
alpha-ENaC_Orcinus_orca                      RESLPHPLQRLPVPAPPHAASRVRRSD-SSLSHSNPKVNRKDWKIGFQLCNQNNSDCFYR  219
alpha-ENaC_Phocoena_sinus                    RESLPHPLQRLQVPAPPHASSRVRRSN-SSLSDNNPQVNRKDWKIGFQLCNQNNSDCFYR  219
alpha-ENaC_Neophocaena_asiaeorientalis       RESLPHPLQRLQVPAPPHAASRVRRSN-SSLSDNNPQVNRKDWKIGFQLCNQNNSDCFYR  219
alpha-ENaC_Monodon_monocerons                RESLPHPLQRLPVPAPPHAASRVRRSD-SSLRDNNPQVNRKDWKIGFQLCNQNNSDCFYR  219
alpha-ENaC_Delphinapterus_leucas             RESLPHPLQRLPVPAPPHAASRVRRSD-SSLRDNNPQVNRKDWKIGFQLCNQNNSDCFYR  219
alpha-ENaC_Pontoporia_blainvillei            RESLPHPLQRLPVPAPPHAASRVRRSG-SSLSDNNPPVKRKDWNIGFKLCNQNNSDCFYQ  219
alpha-ENaC_Inia_geoffrensis                  RESLPHPLQRLPVPAPPHAASRVRRSG-SSLSDNNPQVNRKDWKIGFQLCNQNNSDCFYQ  219
alpha-ENaC_Mesoplodon_bidens                 RESLPHPLQRLPVPAPPHAASRVRRSG-SSLEENNPPVNRKDWNVGFRLCNQNKSDCFYQ  219
alpha-ENaC_Ziphius_cavirostris               RESLPHPLQRLPVPAPPHAASRVRRSG-SAVEENNPQVNRKDWKVGFQLCSQNASECFYQ  219
alpha-ENaC_Platanista_gangetica              LESLPHPLQRLPVPAPPHAASRVRRSG-SSLRDNNPQVNRKDWKIGFQLCNQNKSDCFYQ  219
alpha-ENaC_Platanista_minor                  LESLPHPLQRLPVPAPPHAASRVRRSG-SSLRDNNPQVNRKDWKIGFQLCNQNKSDCFYQ  219
alpha-ENaC_Kogia_breviceps                   RESLPHPLQLLPVPAPPRAASRVRRSG-SSLRDNNPQVNRKDWKIGFQLCNQNKSDCFYQ  219
alpha-ENaC_Physeter_catodon                  RESLPHPLQRLPVPAPPRAASRVRRSG-SSLRDNNPQVNRKDWKIGFQLCNQNKSDCFYQ  219
alpha-ENaC_Balaenoptera_musculus             RESLPHPLQRLPVPAPPHAASRVRRSG-SSLRDNNPQVNRKDWKIGFQLCNQNKSDCFYQ  219
alpha-ENaC_Eubalaena_japonica                RESLPHPLQRVPVPAPPHAASRVRRSG-SSLRDNNPQVNRKDWKIGFQLCNQNKSDCFYQ  219
alpha-ENaC_Hippopotamus_amphibius            REPLPHPLQRLPVAAPPHAARRVRRAG-SSVRDNNPQVNRKDWKIGFQLCNQNKSDCFYQ  219
alpha-ENaC_Tragulus_javanicus                REPLPHPLQRLPVPAPPHAASRVRRAG-SSMRDNNPQVNREDWKIGFQLCNQNKSDCFYK  219
alpha-ENaC_Antilocapra_americana             REPLPHPLQRLPVPAPPHAAGRVRRAG-SSVLDNNPQVNRKDWKIGFQLCNENKSDCFYQ  219
alpha-ENaC_Giraffa_camelopardalis            REPLPHPLQRLPVPAPPHEARRVRHTG-SSVRDNNPQVNRKDWKIGFQLCNQNKSDCFYQ  216
```

```
alpha-ENaC_Giraffa_tippelskirchi           REPLPHPLQRLPVPAPPHEARRVRHTG-SSVRDNNPQVNRKDWKIGFQLCNQNKSDCFYQ  216
alpha-ENaC_Capreolus_pygargus              RRPLPHPLRRLPVPAPPHAARRARRAG-SSVQDNNPQVNRKDWKIGFQLCNQNKSDCFYQ  219
alpha-ENaC_Cervus_elaphus                  RKPLPHPLQRLPVPAPPHEARKVRRAG-SSVRDNNPQVNRKDWKIGFQLCNQNKSDCFYQ  219
alpha-ENaC_Moschus_moschiferus             REPLPHPLQRLPVRTSPHAARRVRRPG-SSVRDNNPQVNRKDWKIGFQLCNQNKSDCFYQ  219
alpha-ENaC_Moschus_berezovskii             REPLPHPLQRLPVPTSPHAARRVRRPG-SSVRDNNPQVNRKDWKIGFQLCNQNKSDCFYQ  219
alpha-ENaC_Bos_grunniens                   REPLPHPLQRLPVPAPSHAARGVRRAG-SSMRDNNPQVNRKDWKIGFQLCNQNKSDCFYQ  219
alpha-ENaC_Bos_taurus                      REPLPHPLQRLPVPAPPHAARGVRRAG-SSMRDNNPQVNRKDWKIGFQLCNQNKSDCFYQ  219
alpha-ENaC_Bubalus_bubalis                 REPLPHPLQRLPVPAPPHAARGVRRAG-SSVRDNNPQVNRKDWKIGFQLCNQNKSDCFYQ  219
alpha-ENaC_Nanger_granti                   REPLPHPLQRLPIPAPPNAARRARRAG-SSVRDNNPQVNRKDWKIGFQLCNQNKSDCFYQ  219
alpha-ENaC_Capra_hircus                    REPLPHPLQRLPIPAPPHAARRVRRAG-SSVRDNNPQVNRKDWKIGFQLCNQNKSDCFYQ  219
alpha-ENaC_Ovis_aries                      REPLPHPLQRLPIPAPPHAARRVRRAG-SSVRDNNPQVNRKDWKIGFQLCNQNKSDCFYQ  219
alpha-ENaC_Ovis_canadensis                 REPLPHPLQRLPIPAPPHAARRVRRAG-SSVRDNNPQVNRKDWKIGFQLCNQNKSDCFYQ  219
alpha-ENaC_Hippotragus_niger               REPLPHPLQRLPIPAPPHAARRVHRTG-SSVRDNNPQVNRKDWKIGFQLCNQNKSDCFYQ  219
alpha-ENaC_Damaliscus_lunatus              REPLPHPLQRLPIPAPPHAARRVHRAG-SSVRDNNPQVNRKDWKIGFQLCNQNKSDCFYQ  219
alpha-ENaC_Sus_scrofa                      REPLPHPLQRLTVPAPPHSARRVRSATSSSVRDNNPQVNRKDWKIGFQLCNQNKSDCFYQ  220
alpha-ENaC_Vicugna_pacos                   REALPHPLQRLPVPAPPHAARSARSA-TSSVRDNNPKVNRKDWKIGFQLCNRNKSDCFYQ  219
alpha-ENaC_Equus_callabus                  GETLPHPLQRLPGPAPPHEARRARMA--SSVRDNNPQVNRKDWKIGFQLCNQNKSDCFYQ  218
                                              *****: :      . :  .:    *:: ..** *. :** :**:**..* *:***:

alpha-ENaC_Homo_sapiens                    TYSSGVDAVREWYRFHYINILSRLP-ETLPSLEEDTLGNFIFACRFNQVSCNQANYSHFH  298
alpha-ENaC_Globicephala_melas              TYSSGVDAVREWYRFHYINILSRRQ-D-TPMLEEEALGKFIFACRFNQVSCNEANYSHFH  277
alpha-ENaC_Lagenorhynchus_obliquidens      TYSSGVDAVREWYRFHYINILSRRQ-D-TPMLEEEALGKFIFACRFNQVSCNEANYSHFH  277
alpha-ENaC_Tursiops_truncatus              TYSSGVDAVREWYRFHYINILSRRQ-D-TPMLEEEALGKFIFACRFNQVSCNEANYSHFH  277
alpha-ENaC_Orcinus_orca                    TYSSGVDAVREWYRFHYINILSRRQ-D-TPMLEEEALGKFIFACRFNQVSCNEANYSHFH  277
alpha-ENaC_Phocoena_sinus                  TYSSGVDAVREWYRFHYINILSRRQ-D-TPLLEEEALGKFIFACRFNQVSCNEANYSHFH  277
alpha-ENaC_Neophocaena_asiaeorientalis     TYSSGVDAVREWYRFHYINILSRRQ-D-TPLLEEEALGKFIFACRFNQVSCNEANYSHFH  277
alpha-ENaC_Monodon_monocerons              TYSSGVDAVREWYRFHYINILSRRQ-D-TPLLEEEALGKFIFACRFNQVSCNEANYSHFH  277
alpha-ENaC_Delphinapterus_leucas           TYSSGVDAVREWYRFHYINILSRRQ-D-TPLLEEEALGKFIFACRFNQVSCNEANYSHFH  277
alpha-ENaC_Pontoporia_blainvillei          TYSSGVDAVREWYRFHYINILSRRQ-D-TPLLEEEALGDFIFACRFNQVSCDEANYSRFH  277
alpha-ENaC_Inia_geoffrensis                TYSSGVDAVREWYRFHYINILSRRQ-D-TPLLEEEALGDFIFACRFNQVSCNEANYSRFH  277
alpha-ENaC_Mesoplodon_bidens               RYSSGVDAVREWYRFHYINILSRRR-D-SPLLEEEALGKFIFACRFNQFSCNEANYSRFH  277
alpha-ENaC_Ziphius_cavirostris             RYSSGVDAVREWYRFHYINILSRRR-D-SPLLEEEALGKFIFACRFNQFSCNEANYSRFH  277
alpha-ENaC_Platanista_gangetica            TYSSGVDAVREWYRFHYINILSRRQ-DTSPSLEEDALGKFIFACRFNQFSCNEANYSHFH  278
alpha-ENaC_Platanista_minor                TYSSGVDAVREWYRFHYINILSRRQ-DTSPSLEEDALGKFIFACRFNQFSCNEANYSHFH  278
alpha-ENaC_Kogia_breviceps                 TYSSGVDAVREWYRFHYINILSRRR-DPSPVLEEDALGKFIFACRFNQASCNEANYSHFH  278
alpha-ENaC_Physeter_catodon                TYSSGVDAVREWYRFHYINILSRRR-DPSPPLEEDALGKFIFACRFNQASCNEANYSHFH  278
alpha-ENaC_Balaenoptera_musculus           TYSSGVDAVREWYRFHYINILSRRQ-DTSPSLEEDALGKFIFACRFNQASCNEANYSHFH  278
alpha-ENaC_Eubalaena_japonica              TYSSGVDAVREWYRFHYINILSRRQ-DTSPSLEEDALGKFIFACRFNQASCNEANYSHFH  278
alpha-ENaC_Hippopotamus_amphibius          TYSSGVDAVREWYRFHYINILSRRK-DTSSSLEEDVLGKFIFTCRFNQVSCNEANYSHFH  278
alpha-ENaC_Tragulus_javanicus              TYSSGVDAVREWYRFHYINILARRQQDTSPLLEEDVLGKFIFTCRFNQNSCNEANYSHFH  279
alpha-ENaC_Antilocapra_americana           KYSSGVDAVREWYRFHYINILSRRRQDTSPSLEEDVLGKFIFTCRFNQDSCNEANYSHFH  279
alpha-ENaC_Giraffa_camelopardalis          TYSSGVDAVREWYRFHYINILSRRRQDTSPSLEEDVLGKFIFTCRFNQDSCNEANYSHFH  276
alpha-ENaC_Giraffa_tippelskirchi           TYSSGVDAVREWYRFHYINILSRRRQDTSPSLEEDVLGKFIFTCRFNQDSCNEANYSHFH  276
alpha-ENaC_Capreolus_pygargus              TYSSGVDAVREWYRFHYINILSRRKQDTSPSLEEDVLGKFIFTCRFNQDSCNEANYSHFH  279
alpha-ENaC_Cervus_elaphus                  TYSSGVDAVREWYRFHYINILSRRRQDTSPSLEEDVLGKFIFTCRFNQDSCNEANYSHFH  279
alpha-ENaC_Moschus_moschiferus             TYSSGVDAVREWYRFHYINILSRRRQDTSPSLEEDVLGKFIFSCRFNQDSCNEANYSHFH  279
alpha-ENaC_Moschus_berezovskii             TYSSGVDAVREWYRFHYINILSRRRQDTSPSLEEDVLGKFIFSCRFNQDSCNEANYSHFH  279
alpha-ENaC_Bos_grunniens                   TYSSGVDAVREWYRFHYINILSRRRQDTSPSLEDDVLGKFIFTCRFNQDSCSEANYSHFH  279
alpha-ENaC_Bos_taurus                      TYSSGVDAVREWYRFHYINILSRRRQDTSPSLEEDVLGKFIFTCRFNQDSCNEANYSHFH  279
alpha-ENaC_Bubalus_bubalis                 TYSSGVDAVTEWYRFHYINILSRRRQDTSPSLEEDVLGKFIFTCRFNQDSCNEANYSHFH  279
alpha-ENaC_Nanger_granti                   TYSSGVDAVREWYRFHYINILSRRRQDTSPSLEEDVLGKFIFTCRFNQDSCNEANYSHFH  279
alpha-ENaC_Capra_hircus                    TYSSGVDAVREWYRFHYINILSRRRQDTSPSLEEDVLGKFIFTCRFNQDSCNEANYSHFH  279
alpha-ENaC_Ovis_aries                      TYSSGVDAVREWYRFHYINILSRRRQDTSPSLEEDVLGKFIFTCRFNQDSCNEANYSHFH  279
alpha-ENaC_Ovis_canadensis                 TYSSGVDAVREWYRFHYINILSRRRRDTSPSLEEDVLGKFIFTCRFNQDSCNEANYSHFH  279
alpha-ENaC_Hippotragus_niger               TYSSGVDAVREWYRFHYINILSRRRQDTSPSLEEDVLGKFIFTCRFNQDSCNEANYSHFH  279
alpha-ENaC_Damaliscus_lunatus              TYSSGVDAVREWYRFHYINILSRRRQDTSPSLEEDVLGKFIFTCRFNQDSCNEANYSHFH  279
alpha-ENaC_Sus_scrofa                      TYSSGVDAVREWYRFHYINILSRRR-DTSPSLEEDALGKFIFACRFNQVSCNEANYSHFH  279
alpha-ENaC_Vicugna_pacos                   TYSSGVDAVREWYRFHYINILARRQ-DTSPSLEEDALGKFIFACRFNQVSCNEANYSHFH  278
alpha-ENaC_Equus_callabus                  TYSSGVDAVREWYRFHYINILSRLPVDASVSLEKDQLDNFIFACRFNQVSCNQANYSHFH  278
                                            ******** ***********:*   :    **.: *..***:***** **.:****:**

alpha-ENaC_Homo_sapiens                    HPMYGNCYTFNDKNNSNLWMSSMPGINNGLSLMLRAEQNDFIPLLSTVTGARVMVHGQDE  358
alpha-ENaC_Globicephala_melas              HPMYGNCYTFNDKNNSNLWMSFMPGVNNGLSLMLRTEQNDFIPLLSTVTGARVMVHGQDE  337
alpha-ENaC_Lagenorhynchus_obliquidens      HPMYGNCYTFNDKNNSNLWMSFMPGVNNGLSLMLRTEQNDFIPLLSTVTGARVMVHGQDE  337
alpha-ENaC_Tursiops_truncatus              HPMYGNCYTFNDKNNSNLWMSFMPGVNNGLSLMLRTEQNDFIPLLSTVTGARVMVHGQDE  337
alpha-ENaC_Orcinus_orca                    HPMYGNCYTFNDKNNSNLWMSFMPGVNNGLSLMLRTEQNDFIPLLSTVTGARVMVHGQDE  337
alpha-ENaC_Phocoena_sinus                  HPMYGNCYTFNDKNTSNLWMSFMPGVNNGLSLTLRTEQNDFIPLLSTVTGARVMVHGQDE  337
alpha-ENaC_Neophocaena_asiaeorientalis     HPMYGNCYTFNDKNTSNLWMSFMPGVNNGLSLTLRTEQNDFIPLLSTVTGARVMVHGQDE  337
alpha-ENaC_Monodon_monocerons              HPMYGNCYTFNNKNNSNLWMSFMPGVNNGLSLTLRTEQNDFIPLLSTVTGARVMVHGQDE  337
alpha-ENaC_Delphinapterus_leucas           HPMYGNCYTFNNKNNSNLWMSFMPGVNNGLSLTLRTEQNDFIPLLSTVTGARVMVHGQDE  337
alpha-ENaC_Pontoporia_blainvillei          HPMYGNCYTFNHKNSSKLWMSFMPGVNNGLSLTLRTEQNDFIPLLSTVTGARVMVHGQDE  337
alpha-ENaC_Inia_geoffrensis                HPMYGNCYTFNDKNSSNLWMSFMPGVNNGLSLTLRTEQNDFIPLLSTVTGARVMVHGQDE  337
alpha-ENaC_Mesoplodon_bidens               HPMYGNCYTFNDKNSSNLWMSSMPGVNNGLSLTLRTEQNDFIPLLSTVTGARVMVHGQDE  337
alpha-ENaC_Ziphius_cavirostris             HPMYGNCYTFNDKNSSNLWMSSMPGVNNGLSLTLRTEQNDFIPLLSTVTGARVMVHGQDE  337
alpha-ENaC_Platanista_gangetica            HPMYGNCYTFNDKNSSNLWMSSMPGVNNGLSLTLRTEQNDFIPLLSTVTGARVMVHGQDE  338
alpha-ENaC_Platanista_minor                HPMYGNCYTFNDKNSSNLWMSSMPGVNNGLSLTLRTEQNDFIPLLSTVTGARVMVHGQDE  338
alpha-ENaC_Kogia_breviceps                 HPMYGNCYTFNDKNSSNLWMSSMTGVNNGLSLMLRTEQNDFIPPLSTVTGARVMVHGQDE  338
alpha-ENaC_Physeter_catodon                HPMYGNCYTFNDKNSSNLWMSSMTGVNNGLSLTLRTEQNDFIPLLSTVTGARVMVHGQDE  338
alpha-ENaC_Balaenoptera_musculus           HPMYGNCYTFNDKNSSNLWMSSISGVNNGLSLTLRTEQNDFIPLLSTVTGARVMVHGQDE  338
alpha-ENaC_Eubalaena_japonica              HPMYGNCYTFNDKNSSNLWMSSISGVNNGLSLTLRTEQNDFIPLLSTVTGARVMVHGQDE  338
alpha-ENaC_Hippopotamus_amphibius          HPIYGNCYTFNDKNSSNLWMSSMPGVNNGLSLTLRTEQNDFIPLLSTVTGARVMVHGQDE  338
alpha-ENaC_Tragulus_javanicus              HPMYGNCYTFNDKNSSNRWMSSRPGVSNGLSLTLRTEQNDFIPLLSTVTGARVMVHERDE  339
alpha-ENaC_Antilocapra_americana           HPMFGNCYTFNDKNSSNLWMSSMPGVNNGLSLTLRTEQNDFIPLLSTVTGARVMVHERDE  339
alpha-ENaC_Giraffa_camelopardalis          HPMYGNCYTFNDKNSSNLWMSSMPGVNNGLSLTLRTEQNDFIPLLSTVTGARVMVHERDE  336
```

```
alpha-ENaC_Giraffa_tippelskirchi             HPMYGNCYTFNDKNSSNLWMSSMPGVNNGLSLTLRTEQNDFIPLLSTVTGARVMVHERDE  336
alpha-ENaC_Capreolus_pygargus                HPMYGNCYTFNDKNSSNLWMSSMPGVNNGLSLTLRTEQNDFIPLLSTVTGARVMVHERDE  339
alpha-ENaC_Cervus_elaphus                    HPMYGNCYTFNDKNSSNLWMSSMPGVNNGLSLTLRTEQNDFIPLLSTVTGARVMVHERDE  339
alpha-ENaC_Moschus_moschiferus               HPMYGNCYTFNDKNSSTLWMSSMPGVNNGLSLTLRTEQNDFIPLLSTVTGARVMVHERDE  339
alpha-ENaC_Moschus_berezovskii               HPMYGNCYTFNDKNSSTLWMSSMPGVNNGLSLTLRTEQNDFIPLLSTVTGARVMVHERDE  339
alpha-ENaC_Bos_grunniens                     HPMYGNCYTFNDKNSSNLWMSSMPGVNNGLSLTLRTEQNDFIPLLSTVTGARVMVHERDE  339
alpha-ENaC_Bos_taurus                        HPMYGNCYTFNDKNSSNLWMSSMPGVNNGLSLTLRTEQNDFIPLLSTVTGARVMVHERDE  339
alpha-ENaC_Bubalus_bubalis                   HPMYGNCYTFNDKNNSNLWMSSMPGVNNGLSLTLRTEQNDFIPLLSTVTGARVMVHERDE  339
alpha-ENaC_Nanger_granti                     HPMYGNCYTFNDKNSSNLWMSSMPGVNNGLSLTLRTEQNDFIPLLSTVTGARVMVHERDE  339
alpha-ENaC_Capra_hircus                      HPMYGNCYTFNDKNSSNLWMSSMPGVNNGLSLTLRTEQNDFIPLLSTVTGARVMVHERDE  339
alpha-ENaC_Ovis_aries                        HPMYGNCYTFNDKNSSNLWISSVPGVNNGLSLTLRTEQNDFIPLLSTVTGARVMVHERDE  339
alpha-ENaC_Ovis_canadensis                   HPMYGNCYTFNDKNSSNLWISSVPGVNNGLSLTLRTEQNDFIPLLSTVTGARVMVHERDE  339
alpha-ENaC_Hippotragus_niger                 HPMYGNCYTFNDKNSSNLWMSSMAGVNNGLSLTLRTEQNDFIPLLSTVTGARVMVHERDE  339
alpha-ENaC_Damaliscus_lunatus                HPMYGNCYTFNDKNSSNLWMSSVPGVNNGLSLTLRTEQNDFIPLLSTVTGARVMVHERDE  339
alpha-ENaC_Sus_scrofa                        HPIYGNCYTFNDKNSSNLWMSSMPGVNNGLSLTLRTEQNDFIPLLSTVTGARVMVHGQDE  339
alpha-ENaC_Vicugna_pacos                     HPIYGNCYTFNDKNSSNLWMSSMPGVNHGLSLTLRTEQNDFIPLVSTVTGARVMVHGQNE  338
alpha-ENaC_Equus_callabus                    HPMYGNCYTFNDKNNSNLWMSSMPGINNGLSLTLRTEQNDFIPLLSTVTGARVMVHGQDE  338
                                             **::*******.**.*.  *:*   *:.:**** **:******* :*********** ::*

alpha-ENaC_Homo_sapiens                      PAFMDDGGFNLRPGVETSISMRKETLDRLGGDYGDCTKNGSDVPVENLYPSKYTQQVCIH  418
alpha-ENaC_Globicephala_melas                PPFMDDGGFNLRPGMETSISMSKEAVERLGGDYGDCTKNGSDVPVENLYGTKYTQQVCIH  397
alpha-ENaC_Lagenorhynchus_obliquidens        PAFMDDGGFNLRPGMETSISMSKEAVDRLGGDYGDCTKNGSDVPVENLYGTKYTQQVCIH  397
alpha-ENaC_Tursiops_truncatus                PAFMDDGGFNLRPGMETSISMSKEAVDRLGGDYGDCTKNGSDVPVENLYGTKYTQQVCIH  397
alpha-ENaC_Orcinus_orca                      PAFMDDGGFNLRPGMETSISMSKEAVDRLGGDYGDCTKNGSDVPVENLYGTKYTQQVCIH  397
alpha-ENaC_Phocoena_sinus                    PAFMDDGGFNLRPGVETSISMSKEAVDRLGGDYGDCTKNGSDVPVENLYGTKYTQQVCIH  397
alpha-ENaC_Neophocaena_asiaeorientalis       PAFMDDGGFNLRPGVETSISMSKEAVDRLGGDYGDCTKNGSDVPVENLYGTKYTQQVCIH  397
alpha-ENaC_Monodon_monocerons                PAFMDDGGFNLRPGMETSISMSKEAVDRLGGDYGDCTKNGSDVPVENLYGTKYTQQVCIH  397
alpha-ENaC_Delphinapterus_leucas             PAFMDDGGFNLRPGMETSISMSKEAVDRLGGDYGDCTKNGSDVPVENLYGTKYTQQVCIH  397
alpha-ENaC_Pontoporia_blainvillei            PAFMDDGGFNLRPGMETSISMSKEAVGRLGGDYGDCTKNGSDVPVENLYGTKYSQQVCIH  397
alpha-ENaC_Inia_geoffrensis                  PAFMDDGGFNLRPGMETSISMSKEAMDRLGGDYGDCTKNGSDVPVENLYGTKYTQQVCIY  397
alpha-ENaC_Mesoplodon_bidens                 PAFMDDGGFNLRPGMETSISMSKETMDRLGGDYGDCTKNGSDVPVENLYGTKYTQQVCIH  397
alpha-ENaC_Ziphius_cavirostris               PAFMDDGGFNLRPGMETSISMSKETMDRLGGDYGDCTKNGSDVPVENLYGTKYTQQVCIH  397
alpha-ENaC_Platanista_gangetica              PAFMDDGGFNLRPGMETSISMSKETVDRLGGDYGDCTKNGSDVPVENLYGAKYTQQVCIH  398
alpha-ENaC_Platanista_minor                  PAFMDDGGFNLRPGMETSISMSKETVDRLGGDYGDCTKNGSDVPVENLYGAKYTQQVCIH  398
alpha-ENaC_Kogia_breviceps                   PAFMDDGGFNLRPGMETSISMSKEAVKRLGGDYGDCTKNGSDVPVENLYGTKYTQQVCIH  398
alpha-ENaC_Physeter_catodon                  PAFMDDGGFNLRPGMETSISMSKEAVKRLGGDYGDCTKNGSDVPVENLYGTKYTQQVCIH  398
alpha-ENaC_Balaenoptera_musculus             PAFMDDGGFNLRPGMETSISMSKEAVARLGGDYGDCTKNGSDVPVENLYGTKYTQQVCIH  398
alpha-ENaC_Eubalaena_japonica                PAFMDDGGFNLRPGMETSISMSKEAVERLGGNYGDCTKNGSDVPVENLYGTKYTQQVCIH  398
alpha-ENaC_Hippopotamus_amphibius            PAFMDDGGFNLRPGVETSISMSKEAVDRLGGDYGDCTKNGSEVPVKNLYGTKYTQQVCIH  398
alpha-ENaC_Tragulus_javanicus                PAFMDDAGFNLRPGVETSISMRKEVVGRLGDYGDCTRNGSEVPVENLYNTKYTQQVCIR   399
alpha-ENaC_Antilocapra_americana             PAFMDDAGFNLRPGVETSISMSKEAVDRLGGDYGDCTKNGSDIPVENLYNTKYTQQVCIH  399
alpha-ENaC_Giraffa_camelopardalis            PAFMDDAGFNLRPGVETSISMSKEAVDRLGGDYGDCTKNGSEVPVENLYNTKYTQQVCIH  396
alpha-ENaC_Giraffa_tippelskirchi             PAFMDDAGFNLRPGVETSISMSKEAVDRLGGDYGDCTKNGSEVPVENLYNTKYTQQVCIH  396
alpha-ENaC_Capreolus_pygargus                PAFMDDAGFNLRPGVETSISMSKEAVDRLGGDYGDCTKNGSEVPVENLYNTKYTQQVCIH  399
alpha-ENaC_Cervus_elaphus                    PAFMDDSGFNLRPGVETSISMSKEAVDRLGGDYGDCTKNGSEVPVENLYNTKYTQQVCIH  399
alpha-ENaC_Moschus_moschiferus               PAFMDDAGFNLRPGVETSISMSKEAVDRLGGDYGDCTKNGSEVPVENLYNTKYTQQVCIH  399
alpha-ENaC_Moschus_berezovskii               PAFMDDAGFNLRPGVETSISMSKEAVDRLGGDYGDCTKNGSEVPVENLYNTKYTQQVCIH  399
alpha-ENaC_Bos_grunniens                     PAFMDDAGFNLRPGVETSISMSKEAVDRLGGDYGDCTKNGSEVPVENLYNTKYTQQVCIH  399
alpha-ENaC_Bos_taurus                        PAFMDDAGFNLRPGVETSISMSKEAVDRLGGDYGDCTKNGSEVPVENLYNTKYTQQVCIH  399
alpha-ENaC_Bubalus_bubalis                   HPFMDDAGFNLRPGVETSISISKEAVDRLGGDYGDCTKNGSEVPVENLYNTKYTQQVCIH  399
alpha-ENaC_Nanger_granti                     PAFMDDAGFNLRPGVETSISMSKEAVDRLGGDYGDCTKNGSEVPVENLYNTKYTQQVCIH  399
alpha-ENaC_Capra_hircus                      PAFMDDAGFNLRPGVETSISMSKEALDRLGGDYGDCTKNGSEVPVENLYNTKYTQQVCIH  399
alpha-ENaC_Ovis_aries                        PAFMDDAGFNLRPGVETSISMSKEAVDRLGGDYGDCTKNGSEVPVENLYNTKYTQQVCIH  399
alpha-ENaC_Ovis_canadensis                   PAFMDDAGFNLRPGVETSISMSKEAVDRLGGDYGDCTKNGSEVPVENFYNTKYTQQVCIH  399
alpha-ENaC_Hippotragus_niger                 PAFMDDAGFNLRPGVETSISMSKEAVDRLGGDYGDCTKNGSEVPVENLYNTKYTQQVCIH  399
alpha-ENaC_Damaliscus_lunatus                PAFMDDAGFNLRPGVETSISMSKEAVDRLGGDYGDCTKNGSEVPVENLYNTKYTQQVCIH  399
alpha-ENaC_Sus_scrofa                        PAFMDDGGFNLRPGVESSISMSKEAVDRLGGDYSDCTKNGSEVPVKNLYGSKYTQQVCIH  399
alpha-ENaC_Vicugna_pacos                     PAFMDDGGFNLRPGVETSISMSKEAVDRLGDNYGDCTENGSEIPVENLYLTKYTQQVCIH  398
alpha-ENaC_Equus_callabus                    PAFMDDGGFNLRPGVETSISMRKETLDRLGGTYGDCTKNGSDIPVQNLYGSKYTQQVCIY  398
                                               ****.*******:*:***: **.: ***. *.***.***::**:*:* :**:*****

alpha-ENaC_Homo_sapiens                      SCFQESMIKECGCAYIFYPRPQNVEYCDYRKHSSWGYCYYKLQVDFSSDHLGCFTKCRKP  478
alpha-ENaC_Globicephala_melas                SCFQVNMIRECGCAYIFYPQPRGVEFCDYRKHNSWGYCYYKLQDAFSSDRLGCFTKCRKP  457
alpha-ENaC_Lagenorhynchus_obliquidens        SCFQVNMIRECGCAYIFYPQPRGVEFCDYRKHNSWGYCYYKLQDAFSSDRLGCFTKCRKP  457
alpha-ENaC_Tursiops_truncatus                SCFQVNMIRECGCAYIFYPQPRGVEFCDYRKHNSWGYCYYKLQDAFSSDRLGCFTKCRKP  457
alpha-ENaC_Orcinus_orca                      SCFQVNMIRECGCAYIFYPQPRGVEFCDYRKHNSWGYCYYKLQDAFSSDRLGCFTKCRKP  457
alpha-ENaC_Phocoena_sinus                    SCFQVNMIRECGCAYIFYPQPRGVEFCDYRKHNSWGYCYYKLQDAFSSDRLGCFTKCRKP  457
alpha-ENaC_Neophocaena_asiaeorientalis       SCFQVNMIRECGCAYIFYPQPRGVEFCDYRKHNSWGYCYYKLQDAFSSDRLGCFTKCRKP  457
alpha-ENaC_Monodon_monocerons                SCFQVNMIRECGCAYIFYPQPRGVEFCDYRKHNSWGYCYYKLQDAFSSDRLGCFTKCRKP  457
alpha-ENaC_Delphinapterus_leucas             SCFQVNMIRECGCAYIFYPQPRGVEFCDYRKHNSWGYCYYKLQDAFSSDRLGCFTKCRKP  457
alpha-ENaC_Pontoporia_blainvillei            SCFQVNMIRECGCAYIFYPQPRGVEFCDYRKHNSWGYCYYKLQDAFSSDRLGCFTKCRKP  457
alpha-ENaC_Inia_geoffrensis                  SCFQVNMIRECGCAYIFYPQPRGVEFCDYRKHNSWGYCYYKLQDAFSSDRLGCFTKCRKP  457
alpha-ENaC_Mesoplodon_bidens                 SCFQVNMVRECGCAYIFYPPHRGAEFCDYRKHTSWGYCYYKLQDAFSSDRLGCFTKCRKP  457
alpha-ENaC_Ziphius_cavirostris               SCFQVNMVRECGCAYIFYPPHRGAEFCDYRKHTSWGYCYYKLQDAFSSDRLGCFTKCRKP  457
alpha-ENaC_Platanista_gangetica              SCFQVNMIRECGCAYIFYPLHRGVEFCDYRKHNSWGYCYYKLQDAFSSDRLGCFTKCRKP  458
alpha-ENaC_Platanista_minor                  SCFQVNMIRECGCAYIFYPLHRGVEFCDYRKHNSWGYCYYKLQDAFSSDRLGCFTKCRKP  458
alpha-ENaC_Kogia_breviceps                   SCFQVNMVRECGCAYIFYPPHRGAEFCDYRKHNSWGYCYYKLQDAFSSDRLGCFTKCRKP  458
alpha-ENaC_Physeter_catodon                  SCFQVNMIRECGCAYIFYPLHRGVEFCDYRKHSSWGYCYYKLQDAFSSDRLGCFTKCRKP  458
alpha-ENaC_Balaenoptera_musculus             SCFQVNMIRECGCAYIFYPPPRGVEFCDYRKHNSWGYCYYKLQDAFSSDRLGCFTKCRKP  458
alpha-ENaC_Eubalaena_japonica                SCFQVNMIRECGCAYIFYPPHRGVEFCDYRKHNSWGYCYYKLQDAFSSDRLGCFTKCRKP  458
alpha-ENaC_Hippopotamus_amphibius            SCFQESMIRECGCAYIFYPPSNNSEFCDYRKHNSWGYCYYKLQDAFSSDRLGCFTKCRKP  458
alpha-ENaC_Tragulus_javanicus                SCFQKSMIKKCGCAYILYPRPEGVEFCDYRKHSSWGYCYYKLQDAFSSDRLGCFTKCRKP  459
alpha-ENaC_Antilocapra_americana             SCFQESMIKECGCAYIFYPRPDGVEFCDYRKHNSWGYCYYKLQDAFSSDRLGCFTKCRKP  459
alpha-ENaC_Giraffa_camelopardalis            SCFQESMIKECGCAYIFYPRPHGVEFCDYRKHNSWGYCYYKLQDAFSSDRLGCFTKCRKP  456
```

```
alpha-ENaC_Giraffa_tippelskirchi           SCFQESMIKECGCAYIFYPRPHGVEFCDYRKHNSWGYCYYKLQDAFSSDRLGCFTKCRKP  456
alpha-ENaC_Capreolus_pygargus              SCFQESMIQECGCAYIFYPRPDGVEFCDYRKHNSWGYCYYKLQDAFSSDRLGCFTKCRKP  459
alpha-ENaC_Cervus_elaphus                  SCFQESMIKECGCAYIFYPRPDGVEFCDYRKHNSWGYCYYKLQDAFSSDRLGCFTKCRKP  459
alpha-ENaC_Moschus_moschiferus             SCFQESMIKECGCAYIFYPRPDGVEFCDYRKHNSWGYCYYKLQDAFSSDRLGCFTKCRKP  459
alpha-ENaC_Moschus_berezovskii             SCFQESMIKECGCAYIFYPRPDGVEFCDYRKHNSWGYCYYKLQDAFSSDRLGCFTKCRKP  459
alpha-ENaC_Bos_grunniens                   SCFQESMIKECGCAYIFYPRPDGVEFCDYRKHNSWGYCYYKLQDAFSSDRLGCFTKCRKP  459
alpha-ENaC_Bos_taurus                      SCFQESMIKECGCAYIFYPRPDGVEFCDYRKHNSWGYCYYKLQDAFSSDRLGCFTKCRKP  459
alpha-ENaC_Bubalus_bubalis                 SCFQESMIKECGCAYIFYPLPDGVEFCDYRKHSSWGYCYYKLQDAFSSDRLGCFTKCRKP  459
alpha-ENaC_Nanger_granti                   SCFQESMIKKCGCAYIFYPKPEGVEFCDYKKHDSWGYCYYKLQDAFSSDRLGCFTKCRKP  459
alpha-ENaC_Capra_hircus                    SCFQESMIKECGCAYIFYPRPEGVEFCDYKKHNSWGYCYYKLQDAFSSDRLGCFTKCRKP  459
alpha-ENaC_Ovis_aries                      SCFQESMIKECGCAYIFYPRPEGVEFCDYKKHNSWGYCYYKLQDAFSSDRLGCFTKCRKP  459
alpha-ENaC_Ovis_canadensis                 SCFQESMIKECGCAYIFYPRPEGVEFCDYKKHNSWGYCYYKLQDAFSSDRLGCFTKCRKP  459
alpha-ENaC_Hippotragus_niger               SCFQESMIKECGCAYIFYPRPEGVEFCDYKKHNSWGYCYYKLQDAFSSDRLGCFTKCRKP  459
alpha-ENaC_Damaliscus_lunatus              SCFQESMIKECGCAYIFYPRPEGIEFCDYKKHNSWGYCYYKLQDAFSSDRLGCFTKCRKP  459
alpha-ENaC_Sus_scrofa                      SCFQQNMVKECGCAYIFYPLPPGMEFCDYRKHNSWGYCYYKLQDAFSSDRLGCFTKCRKP  459
alpha-ENaC_Vicugna_pacos                   SCFQESMVRECGCAYIFYPRPHNVDFCDYRKHDSWGYCYYKLQDAFSSDRLGCFTKCRKP  458
alpha-ENaC_Equus_callabus                  SCFQENMIKECGCAYLFYPPLPGVDFCDYRKHNSWGYCYYKLQDAFASNRLGCFTKCRKP  458
                                           **** .*:::*****::**   . ::***:** **********  *:*::**********

alpha-ENaC_Homo_sapiens                    CSVTSYQLSAGYSRWPSVTSQEWVFQMLSRQNNYTVNNKRNGVAKVNIFFKELNYKTNSE  538
alpha-ENaC_Globicephala_melas              CNMTTYKLSAGYSRWPSVTSQDWVFQMLSLQNNYTVKNKRDGIAKLNIFFKELNYKTNSE  517
alpha-ENaC_Lagenorhynchus_obliquidens      CNMTTYKLSAGYSRWPSVTSQDWVFQMLSLQNNYTVKNKRDGIAKLNIFFKELNYKTNSE  517
alpha-ENaC_Tursiops_truncatus              CNMTTYKLSAGYSRWPSVTSQDWVFQMLSLQNNYTVKNKRDGIAKLNIFFKELNYKTNSE  517
alpha-ENaC_Orcinus_orca                    CKMTTYKLSAGYSRWPSVTSQDWVFQMLSLQNNYTVKNKRDGIAKLNIFFKELNYKTNSE  517
alpha-ENaC_Phocoena_sinus                  CNMTTYKLSAGYSRWPSVTSQDWVFQMLSLQNNYTVKNKRDGVAKLNIFFKELNYKTNSE  517
alpha-ENaC_Neophocaena_asiaeorientalis     CNMTTYKLSAGYSRWPSVTSQDWVFQMLSLQNNYTVKNKRDGVAKLNIFFKELNYKTNSE  517
alpha-ENaC_Monodon_monocerons              CNMTTYKLSAGYSRWPSVTSQDWVFQMLSLQNNYTVKNKRDGVAKLNIFFKELNYKTNSE  517
alpha-ENaC_Delphinapterus_leucas           CNMTTYKLSAGYSRWPSVTSQDWVFQMLSLQNNYTVKNKRDGVAKLNIFFKELNYKTNSE  517
alpha-ENaC_Pontoporia_blainvillei          CNVTTYKLSAGYSRWPSVTSQDWVFQMLSRQNSYTVKNKRDGVAKLNIFFKELNYKTNSE  517
alpha-ENaC_Inia_geoffrensis                CNVITYKLSAGYSRWPSVTSQDWVFQMLSRQNNYTVKNKRDGVAKLNIFFKELNYKTNSE  517
alpha-ENaC_Mesoplodon_bidens               CRVTTYKLSAGYSRWPSVTSQDWVFRMLSRQNNYTIKNKRDGVAKLNIFFKELNYKTNSE  517
alpha-ENaC_Ziphius_cavirostris             CRVTTYKLSAGYSRWPSVTSQDWVFRMLSRQNNYTIKNKREGVAKLNIFFKELNYKTNSE  517
alpha-ENaC_Platanista_gangetica            CSVTTYKLSAGYSRWPSVTSQDWVFQMLSRQNNYTIKNKRDGVAKLNIFFKELNYKTNSE  518
alpha-ENaC_Platanista_minor                CSVTTYKLSAGYSRWPSVTSQDWVFQMLSRQNNYTIKNKRDGVAKLNIFFKELNYKTNSE  518
alpha-ENaC_Kogia_breviceps                 CSVTTYKLSAGYSRWPSVTSQDWVFQMLSRQNSYTVKNKRDGVAKLNIFFKELNYKTNSE  518
alpha-ENaC_Physeter_catodon                CSVTTYKLSAGYSRWPSVTSQDWVFQMLSRQNNYTIKNKRDGVAKLNIFFKELNYKTNSE  518
alpha-ENaC_Balaenoptera_musculus           CSVTTYKLSAGYSRWPSVTSQDWVFKMLSRQNNYTIKNKRDGVAKLNIFFKELNYKTNSE  518
alpha-ENaC_Eubalaena_japonica              CSVTTYKLSAGYSRWPSVTSQDWVFKMLSRQNNYTIKNKRDGVAKLNIFFKELNYKTNSE  518
alpha-ENaC_Hippopotamus_amphibius          CSVTSYKLSAGYSRWPSVTSQDWVFQMLSRQNNYTIKNKRDGVAKLNIFFKELNYKTNSE  518
alpha-ENaC_Tragulus_javanicus              CSLTIYRLSASYSQWPSVTSQDWVFEMLSRQNNYTIKNKRDGVAKLNIFFKELNYKSNTE  519
alpha-ENaC_Antilocapra_americana           CSVTIYKLSASYSQWPSVTSQDWVFQMLSRQNNYTIKNKRDGVAKLNIFFKELNYKSNSE  519
alpha-ENaC_Giraffa_camelopardalis          CSVTIYKLSASYSQWPSVTSQDWVFQMLSRQNNYTIKNKRDGVAKLNIFFKELNYKSNSE  516
alpha-ENaC_Giraffa_tippelskirchi           CSVTIYKLSASYSQWPSVTSQDWVFQMLSRQNNYTIKNKRDGVAKLNIFFKELNYKSNSE  516
alpha-ENaC_Capreolus_pygargus              CSVTIYKLSASYSQWPSMTSQDWVFQMLSRQNNYTIKNKRDGVAKLNIFFKELNYKSNSE  519
alpha-ENaC_Cervus_elaphus                  CSVTIYKLSASYSQWPSVTSQDWVFQMLSRQNNYTIKNKRDGVAKLNIFFKELNYKSNSE  519
alpha-ENaC_Moschus_moschiferus             CSVTIYKLSASYSQWPSVTSQDWVFQMLSRQNNYTIKNKRDGVAKLNIFFKELNYKSNSE  519
alpha-ENaC_Moschus_berezovskii             CSVTIYKLSASYSQWPSVTSQDWVFQMLSRQNNYTIKNKRDGVAKLNIFFKELNYKSNSE  519
alpha-ENaC_Bos_grunniens                   CSVTIYKLSASYSQWPSATSQDWVFQMLSRQNNYTIKNKRDGVAKLNIFFKELNYKSNSE  519
alpha-ENaC_Bos_taurus                      CSVTIYKLSASYSQWPSATSQDWVFQMLSRQNNYTIKNKRDGVAKLNIFFKELNYKSNSE  519
alpha-ENaC_Bubalus_bubalis                 CSVTIYKLSASYSQWPSVTSQDWVFQMLSRQNNYTVKNKRDGVAKLNIFFKELNYKSNSE  519
alpha-ENaC_Nanger_granti                   CSVTIYKLSASYSQWPSMTSQDWVFQMLSRQNNYTIKNKRDGVAKLNIFFKELNYKSNSE  519
alpha-ENaC_Capra_hircus                    CSVTIYKLSASYSQWPSVTSQDWVFQMLSRQNNYTIKNKRDGVAKLNIFFKELNYKSNSE  519
alpha-ENaC_Ovis_aries                      CSVTIYKLSASYSQWPSVTSQDWVFQMLSRQNNYTIKNKRDGVAKLNIFFKELNYKSNSE  519
alpha-ENaC_Ovis_canadensis                 CSVTIYKLSASYSQWPSVTSQDWVFQMLSRQNNYTIKNKRDGVAKLNIFFKELNYKSNSE  519
alpha-ENaC_Hippotragus_niger               CSVTIYKLSASYSQWPSVTSQDWVFEMLSRQNNYTIKNKRDGVAKLNIFFKELNYKSNSE  519
alpha-ENaC_Damaliscus_lunatus              CSVTIYKLSASYSQWPSVTSQDWVFEMLSRQNNYTIKNKRDGVAKLNIFFKELNYKSNSE  519
alpha-ENaC_Sus_scrofa                      CSVTTYKLSAGYSRWPSVTSQDWVFQMLSRQNNYTIKNKRNGVAKVNIFFKELNYKTNSE  519
alpha-ENaC_Vicugna_pacos                   CSVTMYKLSAGYSRWPSVTSQDWVFEMLSRQNNYTIKDKRNGVAKLNIFFKELNYKTNSE  518
alpha-ENaC_Equus_callabus                  CSVTSYQLSAGYSRWPSVTSQDWVFQMLSLQNNYTINNKRNGVAKLNIFFEELNYKTNSE  518
                                           * :  *:***.**:*** ***:***.*** **.**::**:*:**:****:*****:*:*

                                                TM2
alpha-ENaC_Homo_sapiens                    SPSVTMVTLLSNLGSQWSLWFGSSVLSVVEMAELVFDLLVIMFLMLLRRFRSRYWSPGRG  598
alpha-ENaC_Globicephala_melas              SPSVTMVTLLSNLGSQWSLWFGSSVLSVVEMAELIFDLLAITFFMLLRRFQSQYWSPGRG  577
alpha-ENaC_Lagenorhynchus_obliquidens      SPSVTMVTLLSNLGSQWSLWFGSSVLSVVEMAELIFDLLAITFFMLLRRFQSQYWSPGRG  577
alpha-ENaC_Tursiops_truncatus              SPSVTMVTLLSNLGSQWSLWFGSSVLSVVEMAELIFDLLAITFFMLLRRFQSQYWSPGRG  577
alpha-ENaC_Orcinus_orca                    SPSVTMVTLLSNLGSQWSLWFGSSVLSVVEMAELIFDLLAITFFMLLRRFQSQYWSPGRG  577
alpha-ENaC_Phocoena_sinus                  SPSVTMVTLLSNLGSQWSLWFGSSVLSVVEMAELIFDLLAITFLMLLRRFQSQYWSPGRG  577
alpha-ENaC_Neophocaena_asiaeorientalis     SPSVTMVTLLSNLGSQWSLWFGSSVLSVVEMAELIFDLLAITFLMLLRRFQSQYWSPGRG  577
alpha-ENaC_Monodon_monocerons              SPSVTMVTLLSNLGSQWSLWFGSSVLSVVEMAELIFDLLAITFLMLLRRFQSQYWSPGRG  577
alpha-ENaC_Delphinapterus_leucas           SPSVTMVTLLSNLGSQWSLWFGSSVLSVVEVAELIFDLLAITFLMLLRRFQSQYWSPGRG  577
alpha-ENaC_Pontoporia_blainvillei          SPSVTMVTLLSNLGSQWSLWFGSSVLSVVEMAELVFDLLAITFLMLLRRFQSQYWSPGRG  577
alpha-ENaC_Inia_geoffrensis                SPSVTMVTLLSKLGSQWSLWFGSSVLSVVEMAELVFDLLAITFLMLLRRFQSQYWSPGRG  577
alpha-ENaC_Mesoplodon_bidens               SPSVTMVTLLSNLGSQWSLWFGSSVLSVVEMAELIFDLLAITFLMLLRRFQSQYWSPGRG  577
alpha-ENaC_Ziphius_cavirostris             SASITMVTLLSNLGSQWSLWFGSSVLSVVEMAELIFDLLAITFLMLLRRLQSQYWSPGRG  577
alpha-ENaC_Platanista_gangetica            SPSVTMVTLLSNLGSQWSLWFGSSVLSVVEMAELIFDLLAITFLMLLRRFQSQYWSPGRG  578
alpha-ENaC_Platanista_minor                SPSVTMVTLLSNLGSQWSLWFGSSVLSVVEMAELIFDLLAITFLMLLRRFQSQYWSPGRG  578
alpha-ENaC_Kogia_breviceps                 SPSVTMVTLLSNLGSQWSLWFGSSVLSVVEMAELIFDLLAITFLMLLRRFQSRYWSPGRG  578
alpha-ENaC_Physeter_catodon                SPSVTMVTLLSNLGSQWSLWFGSSVLSVVEMAELIFDLLAITFLMLLRRVQSRYWSPGRG  578
alpha-ENaC_Balaenoptera_musculus           SPSVTTVTLLSNLGSQWSLWFGSSVLSVVEVAELIFDLLAITFLMLLRRFQSQYWSPGRG  578
alpha-ENaC_Eubalaena_japonica              SPSVTTVTLLSNLGSQWSLWFGSSVLSVVEVAELIFDLLAITFLMLLRRFQSQYWSPGRG  578
alpha-ENaC_Hippopotamus_amphibius          SPSVTMVTLLSNLGSQWSLWFGSSVLSVVEMAELLFDLLVITFLMLLRRCRSRYWSPGRG  578
alpha-ENaC_Tragulus_javanicus              SPSVKMATLLSNLGSQWSLWFGSSVLSVVEMAELIFDLLVITILMLLRRLRSRYWSPGRG  579
alpha-ENaC_Antilocapra_americana           SPSVTMVTLLSNLGSQWSLWFGSSVLSVVEMAELIFDLLVITFLMLLRRFQSRYWSPGRG  579
```

```
alpha-ENaC_Giraffa_camelopardalis           SPLVTMVTLLSKLGSQWSLWFGSSVLSVVEMAELIFDLLVITFLMLLRRFRSRYWSPSRG  576
alpha-ENaC_Giraffa_tippelskirchi            SPLVTMVTLLSKLGSQWSLWFGSSVLSVVEMAELIFDLLVITFLMLLRRFRSRYWSPSRG  576
alpha-ENaC_Capreolus_pygargus               SPSVTMVTLLSNLGSQWSLWFGSSVLSVVEMAELIFDLLVITFLMLLRRFRSRYWSPGRG  579
alpha-ENaC_Cervus_elaphus                   SPSVTMVTLLSNLGSQWSLWFGSSVLSVVEMAELIFDLLVITFLMLLRRFRSRYWSPGRG  579
alpha-ENaC_Moschus_moschiferus              SPSVTMVTLLSNLGSQWSLWFGSSVLSVVEMAELIFDLLVITFLMLLRRFRSRYWSPGRG  579
alpha-ENaC_Moschus_berezovskii              SPSVTMVTLLSNLGSQWSLWFGSSVLSVVEMAELIFDLLAITFLMLLRRFRSRYWSPGRG  579
alpha-ENaC_Bos_grunniens                    SPSVTMVTLLSNLGSQWSLWFGSSVLSVVEMAELIIDLLVITFLMLLRRFRSRYWSPGRG  579
alpha-ENaC_Bos_taurus                       SPSVTMVTLLSNLGSQWSLWFGSSVLSVVEMAELIIDLLVITFLMLLRRFRSRYWSPGRG  579
alpha-ENaC_Bubalus_bubalis                  SPSVTMVTLLSNLGSQWSLWFGSSVLSVVEMAELIIDLLVITFLMLLRRFRSRYWSPGRG  579
alpha-ENaC_Nanger_granti                    SPSVTMVTLLSNLGSQWSLWFGSSVLSVVEMAELIFDLLVITFLMLLRRFRSRYWSPGRG  579
alpha-ENaC_Capra_hircus                     SPSVTMVTLLSNLGSQWSLWFGSSVLSVVEMAEFIFDLLVITFLMLLRRFRSRYWSPGRG  579
alpha-ENaC_Ovis_aries                       SPSVTMVTLLSNLGSQWSLWFGSSVLSVVEMAELIFDLLVITFLMLLRRFRSRYWSPGRG  579
alpha-ENaC_Ovis_canadensis                  SPSVTMVTLLSNLGSQWSLWFGSSVLSVVEMAELIFDLLVITFLMLLRRFRSRYWSPGRG  579
alpha-ENaC_Hippotragus_niger                SPSVTMVTLLSNLGSQWSLWFGSSVLSVVEMAELIFDLLVITFLMLLRRFRSRYWSPGRG  579
alpha-ENaC_Damaliscus_lunatus               SPSVTMVTLLSNLGSQWSLWFGSSVLSVVEMAELIFDLLVITFLMLLRRFRSRYWSPGRG  579
alpha-ENaC_Sus_scrofa                       SPSVTMVTLLSNLGSQWSLWFGSSVLSVVEMAELIFDLLVITFLMLLRRFRSRYWSPGRG  579
alpha-ENaC_Vicugna_pacos                    SPSVTMVTLLSNLGGQWSLWFGSSVLSVVEMAELVFDLLAITFLMLIRRVRSRYWSPGRG  578
alpha-ENaC_Equus_callabus                   SPSVTMVTLLSNLGSQWSLWFGSSVLSVVEMAELIFDLLVITFLMLLRRIRSRYWSPGRG  578
                                            *  :. .****:**.***************:**:::***.* ::**:** :*:****.**

alpha-ENaC_Homo_sapiens                     GRGAQEVASTLASSPPSHFCPHPMSLSLSQPGPAPSPALTAPPPAYATLGPRPSPGGSAG  658
alpha-ENaC_Globicephala_melas               GRGAQEVASTPPSSLPSRFCPHPASPSSSAAGPATSLALSAPPPAYATLGPRLAPSGSTE  637
alpha-ENaC_Lagenorhynchus_obliquidens       GRGAQEVASTPPSSLPSRFCPHPASPSSSAAGPATSLALSAPPPAYATLGPRLAPSGSTE  637
alpha-ENaC_Tursiops_truncatus               GRGAQEVASTPPSSLPSRFCPHPASPSSSAAGPATSLALSAPPPAYATLGPRLAPSGSTE  637
alpha-ENaC_Orcinus_orca                     GRGAQEVASTPPSSLPSRFCPHPASPSSSAVGPATSLALSAPPPAYATLGPRLAPSGSTE  637
alpha-ENaC_Phocoena_sinus                   GRGAQEVASTPPSSLPSRFCPHPASPPASLADPATSLALSAPPPAYATLGPRLAPSGSME  637
alpha-ENaC_Neophocaena_asiaeorientalis      GRGAQEVASTPPSSLPSRFCPHPASPPASLADPATSLALSAPPPAYATLGPRLAPSGSME  637
alpha-ENaC_Monodon_monocerons               GRGAQEVASTPASSLPSRFCPHPASPSSSLAGPATSLALSAPPPAYATLGPRLAPSGSME  637
alpha-ENaC_Delphinapterus_leucas            GRGAQEVASTPASSLPSRFCPHPASPSSSLGGPATSLALSAPPPAYATLGPRLAPSGSME  637
alpha-ENaC_Pontoporia_blainvillei           GRGAQEVASTPASSLPSRFCPHPATPSSSPAGPATSLALSAPPPAYATLGPHLAPSGSTE  637
alpha-ENaC_Inia_geoffrensis                 GRGAQEVASTPASSLPSRFCPHPTSPSSSPAGPATSLALSAPPPAYATLGPRLAPSGSTE  637
alpha-ENaC_Mesoplodon_bidens                GRGSQEVASTPASSLPSRFCPHPASPSSSPPGPATSLALSAPPPAYATLGPRLALSGSTE  637
alpha-ENaC_Ziphius_cavirostris              GRGSQEVASTPASSLPSRFCPHPASPSSSPPGPATTLALSAPPPAYATLGPRLALSGSTE  637
alpha-ENaC_Platanista_gangetica             GRGAQEVASTPASSLPSRFCPHPASPSSSPPGPATSLALSAPPPAYATLGPRLAPSGSTE  638
alpha-ENaC_Platanista_minor                 GRGAQEVASTPASSLPSRFCPHPASPSSSPPGPATSLALSAPPPAYATLGPRLAPSGSTE  638
alpha-ENaC_Kogia_breviceps                  GSGAREVASMPTSAFPSRFCPHPASPSSFPPGPATSLALSAPPPAYATLGPRWTPSGSTE  638
alpha-ENaC_Physeter_catodon                 GRGAQEVASTPTSALPSRFCPHPASPSSFPPGPAASLALSAPPPAYATLGPRRAPSGSTE  638
alpha-ENaC_Balaenoptera_musculus            RRGAQEVASTPASSLPSRFCPHPASPSSSPPGPATSLALSAPPPAYATLGPRLAPSGSTE  638
alpha-ENaC_Eubalaena_japonica               RRGAQEVASTPASSLPSRFCPHPASPSSSPLGPATSLALSAPPPAYATLGPRLAPSGSTE  638
alpha-ENaC_Hippopotamus_amphibius           RGGAQEVASTPASSLPSRFCPHPASPSSSLPGPAPSPALSAPPPAYATLGPHPAPSGSTE  638
alpha-ENaC_Tragulus_javanicus               GRGAQEVASTPASSPPSSVCPDPASSSSP-GPATAPALSAPPPAYNTLGPHPAPLGLAE  638
alpha-ENaC_Antilocapra_americana            GRGTQEVASTPAASLPSSFCPHPASSSSSPPEPAISPALSAPPPAYATLGPHPGPSGLAE  639
alpha-ENaC_Giraffa_camelopardalis           GRGAQEVASTPASSLPSSFCPHPASSSSSLPHPAISPALSAPPPAYATLGPHPAPSGLVE  636
alpha-ENaC_Giraffa_tippelskirchi            GRGAQEVASTPASSLPSSFCPHPASSSSSLPHPAISPALSAPPPAYATLGPHPAPSGLVE  636
alpha-ENaC_Capreolus_pygargus               GRGSQEVASTPAASLPSSFCPHLASSSSSPPDPAISPALSAPPPAYATLGPHPVPSGLAE  639
alpha-ENaC_Cervus_elaphus                   GRGTQEVASTPAASLPSSFCPHPASSSSSPPDPAISPALSAPPPAYATLGPHPAPSGLAE  639
alpha-ENaC_Moschus_moschiferus              GRGTQELASTPAASLPSSFCPHPASSSSSPPDPAISPALSAPPPAYATLGPHPAPSGLAE  639
alpha-ENaC_Moschus_berezovskii              GRGTQEVASTPAASLPSSFCPHPASSSSSPPDPAISPALSAPPPAYATLGPHPAPSGLAE  639
alpha-ENaC_Bos_grunniens                    GKGTQEVASTPATSLPSSFCPHPAFSSSYPPDPAISPALSAPPPAYATLGPHPAPSGLAE  639
alpha-ENaC_Bos_taurus                       GKGTQEVASTPAASLPSSFCPHPAFFSSSPPDPAISPALSAPPPAYATLGPHPAPSGLAE  639
alpha-ENaC_Bubalus_bubalis                  GKGTQEVASTLAASLPSSFCPHPASSSSSLPDPAISPALSAPPPAYATLGPHPAPSGLAE  639
alpha-ENaC_Nanger_granti                    GRGTQEVASTPATSLPSSFCPHQASSSSSPLDPAVSPALSAPPPAYATLGPHPAPSGLAE  639
alpha-ENaC_Capra_hircus                     GRGTQEVASTPATSLPSSSCPYQASSSSSPPDPAISPALSAPPPAYSTLGPHPAPSGLAE  639
alpha-ENaC_Ovis_aries                       GRGTQEVASTPATSLPSSSCPYQASSSSFPPDPAISPALSAPPPAYSTLGPHPAPTGLAE  639
alpha-ENaC_Ovis_canadensis                  GRGTQEVASTPATSLPSSSCPYQASSSSFPPDPAISLALSAPPPAYSTLGPHPAPSGLAE  639
alpha-ENaC_Hippotragus_niger                GRGTQEVASTPATSLPSSFCPYQASSSSSPPDPAISLALSAPPPAYATLGPHPAPSGLAE  639
alpha-ENaC_Damaliscus_lunatus               GRGTQEVASTPATSLPSSFCPYQASSSSSSPDPAISLALSAPPPAYATLGPHPAPSGLAE  639
alpha-ENaC_Sus_scrofa                       GRGAQEVASTPPSSLPSRFCPHPASPSSSPPGPAANPALSAPPPAYATLGPCPAPSGSAA  639
alpha-ENaC_Vicugna_pacos                    ARAGQEVASASAPPLPSHFCPHPTSPSSSLPGPAPAPALAAPPPAYATLGPPSSLSGSAA  638
alpha-ENaC_Equus_callabus                   RRGAREVASTPASSLPAHFCPNPASPSSSPPGPAASPALTAPPPAYATLGPCPSLASSAG  638
                                              . :*:**      *:  **           **   **:****** ****     .

alpha-ENaC_Homo_sapiens                     ASSSTCPLGGP    669
alpha-ENaC_Globicephala_melas               ASSSAHSPGEP    648
alpha-ENaC_Lagenorhynchus_obliquidens       ASSSAHTPGEP    648
alpha-ENaC_Tursiops_truncatus               ASSSAHTPGEP    648
alpha-ENaC_Orcinus_orca                     ASSSAHTPGEP    648
alpha-ENaC_Phocoena_sinus                   ASSSAHTPGEP    648
alpha-ENaC_Neophocaena_asiaeorientalis      ASSSAHTPGEP    648
alpha-ENaC_Monodon_monocerons               ASSSAHTPGEP    648
alpha-ENaC_Delphinapterus_leucas            ASSSAHTPGEP    648
alpha-ENaC_Pontoporia_blainvillei           ASSSAHTPGEP    648
alpha-ENaC_Inia_geoffrensis                 ASSSAHTPGEP    648
alpha-ENaC_Mesoplodon_bidens                ASSSAHTPGEP    648
alpha-ENaC_Ziphius_cavirostris              ASSSAHTPGEP    648
alpha-ENaC_Platanista_gangetica             ASSSAHTPGKP    649
alpha-ENaC_Platanista_minor                 ASSSAHTPGKP    649
alpha-ENaC_Kogia_breviceps                  ASASAHTAGEP    649
alpha-ENaC_Physeter_catodon                 ASSSAHTLREP    649
alpha-ENaC_Balaenoptera_musculus            ASSSAHTPGEP    649
alpha-ENaC_Eubalaena_japonica               ASSSAHAPGEP    649
alpha-ENaC_Hippopotamus_amphibius           ASSSAHTPGEP    649
alpha-ENaC_Tragulus_javanicus               ANLSAHAPGEP    649
alpha-ENaC_Antilocapra_americana            ASASAHAPGEP    650
```

```
alpha-ENaC_Giraffa_camelopardalis          ASASAHTPGEP    647
alpha-ENaC_Giraffa_tippelskirchi           ASASAHTPGEP    647
alpha-ENaC_Capreolus_pygargus              ASASAHALGEP    650
alpha-ENaC_Cervus_elaphus                  ASASAHTSGEP    650
alpha-ENaC_Moschus_moschiferus             ASASAHAPGEP    650
alpha-ENaC_Moschus_berezovskii             ASASAHAPGEP    650
alpha-ENaC_Bos_grunniens                   ASTSAHAPGEP    650
alpha-ENaC_Bos_taurus                      ASTSAHAPGEP    650
alpha-ENaC_Bubalus_bubalis                 ASTSAHAPGEP    650
alpha-ENaC_Nanger_granti                   ASIFAHAQGEP    650
alpha-ENaC_Capra_hircus                    ASASAHAPGEP    650
alpha-ENaC_Ovis_aries                      ASASAHAPGEP    650
alpha-ENaC_Ovis_canadensis                 ASASAHGPGEP    650
alpha-ENaC_Hippotragus_niger               ASAP-------    643
alpha-ENaC_Damaliscus_lunatus              ASASAHAPGEP    650
alpha-ENaC_Sus_scrofa                      AGSSAHPLGEP    650
alpha-ENaC_Vicugna_pacos                   ASNPAYTPEEV    649
alpha-ENaC_Equus_callabus                  ASSSACTPGEP    649
                                           *.
```

Supplementary Alignment 2

β-ENaC (*SCNN1B*) amino acid sequence alignment created with Clustal Omega (v.1.2.4, accessed 24.03.2024). Key structural motifs are highlighted in the human ENaC subunit based on the Cryo-EM derived structure[1]. Transmembrane domains (TM1/TM2) are highlighted in yellow. An N-Terminal HG-motif affecting ENaC open probability is marked in magenta. Cysteines involved in secondary tertiary structure formation are indicated in red. Residues putatively forming the selectivity filter within TM2 are shown in red font. The C-terminal PPPxY motif regulating membrane abundance is shown in **gray**.

```
                                                                                           TM1
beta-ENaC_Homo_sapiens                          -MHVKKYLLKGLHRLQKGPGYTYKELLVWYCDNTNTHGPKRIICEGPKKKAMWFLLTLLF    59
beta-ENaC_Globicephala_melas                    -MHIKKYLLKCLHRLQKGPGYTYKELLVWYCDNTNTHGPKRIICEGPKKKAMWFVLTLLF    59
beta-ENaC_Lagenorhynchus_obliquidens            -MHIKKYLLKCLHRLQKGPGYTYKELLVWYCDNTNTHGPKRIICEGPKKKAMWFVLTLLF    59
beta-ENaC_Tursiops_truncatus                    -MHIKKYLLKCLHRLQKGPGYTYKELLVWYCDNTNTHGPKRIICEGPKKKAMWFVLTLLF    59
beta-ENaC_Orcinus_orca                          -MHIKKYLLKCLHRLQKGPGYTYKELLVWYCDNTNTHGPKRIICEGPKKKAMWFVLTLLF    59
beta-ENaC_Phocoena_sinus                        -MHIKKYLLKCLHRLQKGPGYTYKELLVWYCDNTNTHGPKRIICEGPKKKAMWFVLTLLF    59
beta-ENaC_Neophocaena_asiaeorientalis           -MHIKKYLLKCLHRLQKGPGYTYKELLVWYCDNTNTHGPKRIICEGPKKKAMWFVLTLLF    59
beta-ENaC_Monodon_monocerons                    MMHIKKYLLKCLHRLQKGPGYTYKELLVWYCDNTNTHGPKRIICEGPKKKAMWFVLTLLF    60
beta-ENaC_Delphinapterus_leucas                 -MHIKKYLLKCLHRLQKGPGYTYKELLVWYCDNTNTHGPKRIICEGPKKKAMWFVLTLLF    59
beta-ENaC_Pontoporia_blainvillei                -MHIKKYLLKCLHRLQKGPGYTYKELLVWYCDNTNTHGPKRIICEGPKKKAMWFVLTLLF    59
beta-ENaC_Inia_geoffrensis                      -MHIKKYLLKCLHRLQKGPGYTYKELLVWYCDNTNTHGPKRIICEGPKKKAMWFVLTLLF    59
beta-ENaC_Lipotes_vexillifer                    -MHIKRYLLKCLHRLQKGPGYTYKELLVWYCDNTNTHGPKRIICEGPKKKAMWFVLTLLF    59
beta-ENaC_Hyperoodon_ampullatus                 -MHIKKYLLKCLHRLQKGPGYTYKELLVWYCDNTNTHGPKRIICEGPKKKAMWFVLTLLF    59
beta-ENaC_Mesoplodon_bidens                     -MHIKKYLLKCLHRLQKGPGYTYKELLVWYCDNTNTHGPKRIICEGPKKKAMWFVLTLLF    59
beta-ENaC_Ziphius_cavirostris                   -MHIKKYLLKCLHRLQKGPGYTYKELLVWYCDNTNTHGPKRIICEGPKKKAMWFVLTLLF    59
beta-ENaC_Platanista_gangetica                  -MHIKKYLLKCLHRLQKGPGYTYKELLVWYCDNTNTHGPKRIICEGPKKKAMWFVLTLLF    59
beta-ENaC_Platanista_minor                      -MHIKKYLLKCLHRLQKGPGYTYKELLVWYCDNTNTHGPKRIICEGPKKKAMWFVLTLLF    59
beta-ENaC_Kogia_breviceps                       -MHIKKYLLKCLHRLQKGPGYTYKELLVWYCDNTNTHGPKRIICEGPKKKAMWFVLTLLF    59
beta-ENaC_Physeter_catodon                      -MHIKKYLLKCLHRLQKGPGYTYKELLVWYCDNTNTHGPKRIICEGPKKKAMWFVLTLLF    59
beta-ENaC_Balaenoptera_musculus                 -MHIKKYLLKCLHRLQKGPGYTYKELLVWYCDNTNTHGPKRIICEGPKKKAMWFVLTLLF    59
beta-ENaC_Balaenoptera_acutorostrata_scammoni   -MHVKKYLLKCLHRLQKGPGYTYKELLVWYCDNTNTHGPKRIICEGPKKKAMWFVLTLLF    59
beta-ENaC_Eubalaena_japonica                    -MHIKKYLLKCLHRMQKGPGYTYKELLVWYCDNTNTHGPKRIICEGPKKKAMWFVLTLLF    59
beta-ENaC_Hippopotamus_amphibius                MVPIKKYLLKCLHRLQKGPGYTYKELLVWYCDNTNTHGPKRIICEGPKKKAMWFVLTLLF    60
beta-ENaC_Tragulus_javanicus                    -MHVKKYLLKGLHRLQKGPGYTYKELLVWYCDNTNTHGPKRIICEGPKKKAMWFVLTLLF    59
beta-ENaC_Tragulus_kanchil                      -MHVKKYLLKGLHRLQKGPGYTYKELLVWYCDNTNTHGPKRIICEGPKKKAMWFVLTLLF    59
beta-ENaC_Antilocapra_americana                 -MHVKKYLLKGLHRLQKGPGYTYKELLVWYCDNTNTHGPKRIICEGPKKKAMWFVLTLLF    59
beta-ENaC_Giraffa_camelopardalis                -MHVKKYLLKGLHRLQKGPGYTYKELLVWYCDNTNTHGPKRIICEGPKKKAMWFMLTLLF    59
beta-ENaC_Giraffa_tippelskirchi                 -MHVKKYLLKGLHRLQKGPGYTYKELLVWYCDNTNTHGPKRIICEGPKKKAMWFMLTLLF    59
beta-ENaC_Capreolus_pygargus                    MMHVKKYLLKGLHRLQKGPGYTYKELLVWYCDNTNTHGPKRIICEGPKKKAMWFVLTLLF    60
beta-ENaC_Cervus_elaphus                        -MHVKKYLLKGLHRLQKGPGYTYKELLVWYCDNTNTHGPKRIICEGPKKKAMWFVLTLLF    59
beta-ENaC_Moschus_moschiferus                   -MHVKKYLLKGLHRLQKGPGYTYKELLVWYCDNTNTHGPKRIICEGPKKKAMWFVLTLLF    59
beta-ENaC_Moschus_berezovskii                   -MHVKKYLLKGLHRLQKGPGYTYKELLVWYCDNTNTHGPKRIICEGPKKKAMWFVLTLLF    59
beta-ENaC_Bos_grunniens                         -MHVKKYLLKGLHRLQKGPGYTYKELLVWYCDNTNTHGPKRIICEGPKKKAMWFVLTLLF    59
beta-ENaC_Bos_taurus                            -MHVKKYLLKGLHRLQKGPGYTYKELLVWYCDNTNTHGPKRIICEGPKKKAMWFVLTLLF    59
beta-ENaC_Bubalus_bubalis                       -MHVKKYLLKGLHRLQKGPGYTYKELLVWYCDNTNTHGPKRIICEGPKKKAMWFVLTLLF    59
beta-ENaC_Nanger_granti                         -MHVKKYLLKGLHRLQKGPGYTYKELLVWYCDNTNTHGPKRIICEGPKKKAMWFVLTLLF    59
beta-ENaC_Capra_hircus                          -MHLKKYLLKGLHRLQKGPGYTYKELLVWYCDNTNTHGPKRIICEGPKKKAMWFVLTLLF    59
beta-ENaC_Ovis_aries                            -MHLKKYLLKGLHRLQKGPGYTYKELLVWYCDNTNTHGPKRIICEGPKKKAMWFVLTLLF    59
beta-ENaC_Ovis_canadensis                       -MHLKKYLLKGLHRLQKGPGYTYKELLVWYCDNTNTHGPKRIICEGPKKKAMWFVLTLLF    59
beta-ENaC_Hippotragus_niger                     MMHVKKYLLKGLHRLQKGPGYTYKELLVWYCGNTNTHGPKRIICEGPKKKAMWFVLTLLF    60
beta-ENaC_Sus_scrofa                            -MHVKKYLLKCLHRLQKGPGYTYKELLVWYCDNTNTHGPKRIIREGPKKKAMWFVLTLLF    59
beta-ENaC_Vicugna_pacos                         -MHVKKYLLKCLHRLQKGPGYTYKELLVWYCDNTNTHGPKRIICEGPKKKAMWFVITLLF    59
beta-ENaC_Equus_callabus                        -MHVKKYLLKGLHRLQKGPGYTYKELLVWYCDNTNTHGPKRIICEGPKKKAMWFLLTLLF    59
                                                 : :*:**** ***:****************.*********** **********::****

beta-ENaC_Homo_sapiens                          AALVCWQWGIFIRTYLSWEVSVSLSVGFKTMDFPAVTICNASPFKYSKIKHLLKDLDELM    119
beta-ENaC_Globicephala_melas                    TSLVCWQWGVFIRTYLNWEVSVSLSIGFKAMDFPAVTVCNTSPFQYSKVKHLLKDLDELM    119
beta-ENaC_Lagenorhynchus_obliquidens            TSLVCWQWGIFIRTYLNWEVSVSLSIGFKAMDFPAVTVCNTSPFQYSKVKHLLKDLDELM    119
beta-ENaC_Tursiops_truncatus                    TSLVCWQWGVFIRTYLNWEVSVSLSIGFKAMDFPAVTVCNTSPFQYSKVKHLLKDLDELM    119
beta-ENaC_Orcinus_orca                          TSLVCWQWGVFIRTYLNWEVSVSLSIGFKAMDFPAVTVCNTSPFQYSKVKHLLKDLDELM    119
beta-ENaC_Phocoena_sinus                        TSLVCWQWGVFIRTYLSWEVSVSLSIGFKAMDFPAVTICNTSPFQYSKVKHLLKDLDELM    119
beta-ENaC_Neophocaena_asiaeorientalis           TSLVCWQWGVFIRTYLSWEVSVSLSIGFKAMDFPAVTICNTSPFQYSKVKHLLKDLDELM    119
beta-ENaC_Monodon_monocerons                    TSLVCWQWGVFIRTYLSWEVSVSLSIGFKAMDFPAVTICNTSPFQYSKVKHLLKDLDELM    120
beta-ENaC_Delphinapterus_leucas                 TSLVCWQWGVFIRTYLSWEVSVSLSIGFKAMDFPAVTICNTSPFQYSKVKHLLKDLDELM    119
beta-ENaC_Pontoporia_blainvillei                TSLVCWQWGVFIRTYLSWEVSVSLSIGFKAMDFPAVTICNTSPFQYSKVKHLLKDLDELM    119
beta-ENaC_Inia_geoffrensis                      TSLVCWQWGVFIRTYLSWEVSVSLSIGFKAMDFPAVTICNTSPFQYSKAKHLLKDLDELM    119
beta-ENaC_Lipotes_vexillifer                    TSLVCWQWGVFIRTYLSWEVSVSLSIGFKAMDFPAVTICNTSPFQYSKVKHLLKDLDELM    119
beta-ENaC_Hyperoodon_ampullatus                 ASLVCWQWGVFIRTYLSWEVSVSLSIGFKAMDFPAVTVCNTSPFRYSKVKHLLKDLDELM    119
beta-ENaC_Mesoplodon_bidens                     ASLVCWQWGVFIRTYLSWEVSVSLSIGFKAMDFPAVTVCNTSPFRYSKVKHLLKDLDELM    119
beta-ENaC_Ziphius_cavirostris                   ASLVCWQWGVFIRTYLSWEVSVSLSIGFKAMDFPAVTVCNTSPFRYSKVKHLLKDLDELM    119
beta-ENaC_Platanista_gangetica                  ASLVCWQWGVFIRTYLSWEVSVSLSVGFKAMDFPAVTICNTSPFQYSKVKHLLKDLDELM    119
beta-ENaC_Platanista_minor                      ASLVCWQWGVFIRTYLSWEVSVSLSVGFKAMDFPAVTICNTSPFQYSKVKHLLKDLDELM    119
beta-ENaC_Kogia_breviceps                       ASLVCWQWGVFIRTYLSWEVSVSLSIGFKAMDFPAVTICNTNPFQYSKVKHLLKDLDELM    119
beta-ENaC_Physeter_catodon                      TSLVCWQWGVFIRTYLSWEVSVSLSIGFKAMDFPAVTICNTSPFQYSKVKHLLKDLDELM    119
beta-ENaC_Balaenoptera_musculus                 TSLVCWQWGVFIRTYLSWEVSVSLSIGFKAMDFPAVTICNTSPFQYSKVKHLLKDLDELM    119
beta-ENaC_Balaenoptera_acutorostrata_scammoni   TSLVCWQWGVFIRTYLSWEVSVSLSIGFKAMDFPAVTICNTSPFQYSKIKHLLKDLDELM    119
beta-ENaC_Eubalaena_japonica                    TSLVCWQWGVFIRTYLSWEVSVSLSIGFKAMDFPAVTICNTSPFQYSKVKHLLKDLDELM    119
beta-ENaC_Hippopotamus_amphibius                TSLVCWQWGVFIKTYLSWEVSVSLSVGFKAMDFPAVTICNTSPFQYSKVKHLLKDLDELM    120
beta-ENaC_Tragulus_javanicus                    ASIVFWQWGVFIKNYLNWDVSVSLSIGFKTMDFPAVTICNTSPFQYPKVQHLLKDLDELM    119
beta-ENaC_Tragulus_kanchil                      ASIVFWQWGVFIKNYLNWDVSVSLSIGFKTMDFPAVTICNTSPFQYPKVQHLLKDLDELM    119
beta-ENaC_Antilocapra_americana                 ASLVCWQWGLFIKTYLNWEVSVSLSIGFKTMDFPAVTICNASPFQYSKVQHLLKDLDELM    119
beta-ENaC_Giraffa_camelopardalis                ASLVCWQWGLFIKTYLNWEVSVSLSIGFKTMDFPAVTICNASPFQYSKVQHLLKDLDELM    119
beta-ENaC_Giraffa_tippelskirchi                 ASLVCWQWGLFIKTYLNWEVSVSLSIGFKTMDFPAVTICNASPFQYSKVQHLLKDLDELM    119
```

```
beta-ENaC_Capreolus_pygargus                          TSLVCWQWGLFIKTYLNWEVSVSLSIGFKTMNFPAVTICNASPFQYSKVQHLLKDLDELM    120
beta-ENaC_Cervus_elaphus                              TSLVCWQWGLFIKTYLNWEVSVSLSIGFKTMDFPAVTICNASPFQYSKVQHLLKDLDELM    119
beta-ENaC_Moschus_moschiferus                         TSLVCWQWGLFIKTYLNWEVSVSLSIGFKTMDFPAVTICNASPFQYSKVQHLLKDLDELM    119
beta-ENaC_Moschus_berezovskii                         TSLVCWQWGLFIKTYLNWEVSVSLSIGFKTMDFPAVTICNASPFQYSKVQHLLKDLDELM    119
beta-ENaC_Bos_grunniens                               TSLVCWQWGLFIKTYLNWEVSVSLSIGFKTMDFPAVTICNASPFQYSKVQHLLKDLDELM    119
beta-ENaC_Bos_taurus                                  TSLVCWQWGLFIKTYLNWEVSVSLSIGFKTMDFPAVTICNASPFQYSKVQHLLKDLDELM    119
beta-ENaC_Bubalus_bubalis                             TSLVCWQWGLFIKTYLNWEVSVSLSIGFKTMDFPAVTICNASPFQYSKVQHLLKDLDELM    119
beta-ENaC_Nanger_granti                               TSLVCWQWGLFIKTYLNWEVSVSLSIGFKTMDFPAVTICNASPFQYSKVQHLLKDLDALM    119
beta-ENaC_Capra_hircus                                TSLVCWQWGLFIKTYLNWEVSVSLSIGFKTMDFPAVTICNASPFQYSKVQHLLKDLDALM    119
beta-ENaC_Ovis_aries                                  TSLVCWQWGLFIKTYLNWEVSVSLSIGFKTMDFPAVTICNASPFQYSKVQHLLKDLDALM    119
beta-ENaC_Ovis_canadensis                             TSLVCWQWGLFIKTYLNWEVSVSLSIGFKTMDFPAVTICNASPFQYSKVQHLLKDLDALM    119
beta-ENaC_Hippotragus_niger                           TSLVCWQWGLFIKTYLNWEVSVSLSIGFKTMDFPAVTICNASPFQYSKVQHLLKDLDALM    120
beta-ENaC_Sus_scrofa                                  TSLVCWQWGVFIRTYLSWEVSVSLSMGFKTMDFPAVTICNASPFQYSKVQHLLKDLDELM    119
beta-ENaC_Vicugna_pacos                               AAIVCWQWGVFIRTYLSWEVSVSLSIGFKTMDFPAVTICNASPFRYSKVKHLLKDLDELM    119
beta-ENaC_Equus_callabus                              AALVCWQWGIFIRTYLSWEVSVSLSVGFKTMDFPAVTICNASPFQYSKVRHLLKDLDELM    119
                                                      :::* ****:**:.**.*:******:***:*:*****:**:.**:* * :******* **

beta-ENaC_Homo_sapiens                                EAVLERILAPELSHANATRNLNFSIWNHTPLVLIDERNPHHPMVLDLFGDNHNGLTSSS-    178
beta-ENaC_Globicephala_melas                          EAVLARILGPELSHVNATRALNLTIWNHVPLVFIDEQNPRHPVVLDLFEDNYNGSASSTP    179
beta-ENaC_Lagenorhynchus_obliquidens                  EAVLARILGPELSHVNATRALNLTIWNHVPLVFIDEQNPRHPVVLDLFEDNYNGSASSSP    179
beta-ENaC_Tursiops_truncatus                          EAVLARILGPELSHVNATRALNLTIWNHVPLVFIDEQNPHHPVVLDLFEDNYNGSAGSTP    179
beta-ENaC_Orcinus_orca                                EAVLARILGPELSHVNATRALNLTIWNHVPLVFIDEQNPRHPVVLDLFEDNYNGSASSTP    179
beta-ENaC_Phocoena_sinus                              EAVLERILGPELSHVNATRALNLTIWNHVPLVFIDEQNPHHPVVLDLFEDNYNGSASSTP    179
beta-ENaC_Neophocaena_asiaeorientalis                 EAVLERILGPELSHVNTTRALNLTIWNHVPLVFIDEQNPHHPVVLDLFEDNYNGSASSTP    179
beta-ENaC_Monodon_monocerons                          EAVLERILGPELSHVNTTRALNLTIWNHVPLVFINEQNPHHPVVLDLFEDNYSGSASSTP    180
beta-ENaC_Delphinapterus_leucas                       EAVLERILGPELSHVNTTRALNLTIWNHVPLVFINEQNPHHPVVLDLFEDNYNGSASSTP    179
beta-ENaC_Pontoporia_blainvillei                      EAVLERILGPELSPVNATRALNLTIWNHVPLVFINEQNPHHPVVLDLFEDSYNGSASSTP    179
beta-ENaC_Inia_geoffrensis                            EAVLERILGPELSPVNATRALNLTIWNHMPLVFINEQNPHHPVVLDLFEDSYNGSASSTP    179
beta-ENaC_Lipotes_vexillifer                          EAVLERILGPELSHVNATRALNLTLWNHMPLVFINEQNPHHPVVLDLFEDNYNGSASSTP    179
beta-ENaC_Hyperoodon_ampullatus                       EAVLERILGPELSHVNTTRVLNLTIWNHMPLVFINEQNPHHPVVLDLFEDNYNGSASSSP    179
beta-ENaC_Mesoplodon_bidens                           EAVLERILGPELSHVNTTRVLNLTIWNHMPLVFINEQNPHHPVVLDLFEDNYNGSASSSP    179
beta-ENaC_Ziphius_cavirostris                         EAVLERILGPELSHVNTTRVLNLTIWNHMPLVFINEQNPHHPVVLDLFEDNYNGSASSSP    179
beta-ENaC_Platanista_gangetica                        EAVLERILGPELSHVNATRALNLTIWNHMPLVFISEENPHHPVVLDLFEDNYNGSASSSP    179
beta-ENaC_Platanista_minor                            EAVLERILGPELSHVNATRALNLTIWNHMPLVFISEENPHHPVVLDLFEDNYNGSASSSP    179
beta-ENaC_Kogia_breviceps                             EAVLERILGPELSHVNATRALNLTIWNHMPLVFINEQNPHHPVVLDLFEDNYNGSASSSP    179
beta-ENaC_Physeter_catodon                            EAVLERILGPELSHVNATRALNLTIWNHMPLVFINEQNPHHPVVLDLFEDNYNGSASSSP    179
beta-ENaC_Balaenoptera_musculus                       EAVLERILGPELSHGNATRALNLTVWNYMPLVFINEQNPHHPVVLDLFEDDYNGSASSSP    179
beta-ENaC_Balaenoptera_acutorostrata_scammoni         EAVLERILGPELSHVNATRALNLTIWNYMPLVFINEQNPHHPVVLDLFEDNYNGSASSSP    179
beta-ENaC_Eubalaena_japonica                          EAVLERILGPELSHVNTTRALNLTIWNYMPLVFINEQNPHHPVVLDLFEDNYNGSASSSP    179
beta-ENaC_Hippopotamus_amphibius                      EAVLERILGPELSHFNATRALNLTIWNHMPLVFINEQNPHHPVVLELFEDNYNGSASNSS    180
beta-ENaC_Tragulus_javanicus                          GAVLERILGPELSRVNTTRTLNLSIWRHTPLVFINEQNPHHPVVLDLFEDNYNGSTSSSS    179
beta-ENaC_Tragulus_kanchil                            GAVLERILGPELSRVNTTRTLNLSIWRHTPLVFINEQNPHHPVVLDLFEDNYNGSTSSSS    179
beta-ENaC_Antilocapra_americana                       EAVLGRILGPELSQVNATRALNLSIWHHTPLVFINEQNPHHPVVLDLFDDNFNGSASSSP    179
beta-ENaC_Giraffa_camelopardalis                      EAVLGRILGPELSQVNATRALNLSIWHHTPLVFINEQNPHHPLVLDLFEDNFNGSASSSP    179
beta-ENaC_Giraffa_tippelskirchi                       EAVLGRILGPELSQVNATRALNLSIWHHTPLVFINEQNPHHPVVLDLFEDNFNGSASSSP    179
beta-ENaC_Capreolus_pygargus                          EAVLERILGPELSQVNATRTLNLSIWHHMPLVFINEQNPHHPVVLDLFEDNFNGSASSSP    180
beta-ENaC_Cervus_elaphus                              EAVLGRILGPELSQVNATRTLNLSIWHHTPLVFINEQNPHHPVVLDLFEDNFNGSASSSP    179
beta-ENaC_Moschus_moschiferus                         EAVLGRILGPELSQVNATRALNLSIWHHTPLVFINEQNPHHPVVLDLFEDNVNGSASSSP    179
beta-ENaC_Moschus_berezovskii                         EAVLGRILGPELSQVNATRALNLSIWHHTPLVFINEQNPHHPVVLDLFEDNVNGSASSSP    179
beta-ENaC_Bos_grunniens                               EAVLGRILGPELSQVNDTRALNLSIWHHTPLVFINEQNPHHPVVLDLFEDNFNGSASNSP    179
beta-ENaC_Bos_taurus                                  EAVLGRILGPELSQVNDTRALNLSIWHHTPLVFINEQNPHHPVVLDLFEDNFNGSASNSP    179
beta-ENaC_Bubalus_bubalis                             EAVLGRILGPELSQVNATRALNLSIWHHTPLVFINEQNPHHPVVLDLFEDNFNGSASNSP    179
beta-ENaC_Nanger_granti                               EAVLERILGPELSQVNATRALNLSIWHHTPLVFINEQNPHHPVVLDLFEDNVNGSASSSP    179
beta-ENaC_Capra_hircus                                EAVLGRILGPELSQVNATTALNLSIWHHTPLVFINEQNPHHPVVLDLFEDNFNGSASSSP    179
beta-ENaC_Ovis_aries                                  EAVLGRILGPELSQVNATTALNLSIWHHTPLVFINEQNPHHPVVLDLFEDNFNGSASSSP    179
beta-ENaC_Ovis_canadensis                             EAVLGRILGPELSQVNATTALNLSIWHHTPLVFINEQNPHHPVVLDLFEDNFNGSASSSP    179
beta-ENaC_Hippotragus_niger                           EAVLGRILGPELSQVNATRALNLSIWHHTPLVFINEQNPHHPVVLDLFEDNFNGSASSSP    180
beta-ENaC_Sus_scrofa                                  EAVLQRILAPELSHVNATETLNFTIWNHTPLVFINEQNPQHPVVLDLFEDNYNGSASSGP    179
beta-ENaC_Vicugna_pacos                               EAVLERILAPELSHINATRALNFTIWNHTPLVFIDEQNPHQPVVLDLFGDNHNDSASSSL    179
beta-ENaC_Equus_callabus                              DAVLERILAPEPSHANATEALNFTIWNHTPLVLIDEQNPHKPVVLDLFGDNHNSSASSSP    179
                                                       *** ***.** *  * *  **:::*.: ***:*.*.**::*:**:** *. .. :..

beta-ENaC_Homo_sapiens                                ASEKICNA-HGCKMAMRLCSLNRTQCTFRNFTSATQALTEWYILQATNIFAQVPQQELVE    237
beta-ENaC_Globicephala_melas                          APGRTCSA-HGCKVAMKLCSHNGTTCNFRNFSSATQAVTEWYALQATNIFAQVPNQELVA    238
beta-ENaC_Lagenorhynchus_obliquidens                  APGRICSA-HGCKVAMKLCSHNGTTCNFRNFSSATQAVTEWYALQATNIFAQVPNEELVA    238
beta-ENaC_Tursiops_truncatus                          APGRTCSA-HGCKVAMKLCSHNGTTCNFRNFSSATQAVTEWYALQATNIFAQVPNQELVA    238
beta-ENaC_Orcinus_orca                                APGRTCSA-HGCKVAMKLCSHNGTTCNFRNFSSATQAVTEWYALQATNIFAQVPNQELVA    238
beta-ENaC_Phocoena_sinus                              APGRTCSA-HGCKVAMKLCSHNGTTCNFRNFSSATQAVTEWYALQATNIFAQVPNQELVA    238
beta-ENaC_Neophocaena_asiaeorientalis                 APGRTCSA-HGCKVAMKLCSHNGTTCNFRNFSSATQAVTEWYALQATNIFAQVPNQELVA    238
beta-ENaC_Monodon_monocerons                          APGRTCSA-HGCKVAMKLCSHNGTTCNFRNFSSATQAVTEWYALQATNIFAQVPNQELVA    239
beta-ENaC_Delphinapterus_leucas                       APGRTCSA-HGCKVAMKLCSHNGTTCNFRNFSSATQAVTEWYALQATNIFAQVPNQELVA    238
beta-ENaC_Pontoporia_blainvillei                      APGRTCSA-HGCKVAMRLCSHNGTTCSFRNFSSATQAVTEWYTLQATNIFAQVPNQELVA    238
beta-ENaC_Inia_geoffrensis                            APGRTCSA-HGCKVAMRLCSHNGTTCSFRNFSSATQAVTEWYTLQATNIFAQVPNQELVA    238
beta-ENaC_Lipotes_vexillifer                          APGRTCSA-HGCKVAMRLCSHNGTTCNFRNFSSATQAVTEWYTLQATNIFAQVPNKELVA    238
beta-ENaC_Hyperoodon_ampullatus                       APGRTCSA-RECKVAMRLCSHNGTTCNFRNFSSATQAVMEWYTLQATNIFAQVPNQELVA    238
beta-ENaC_Mesoplodon_bidens                           APGRTCSA-RGCKVAMRLCSHNGTTCNFRNFSSATQAVTEWYTLQATNIFAQVPNQELVA    238
beta-ENaC_Ziphius_cavirostris                         APGRTCSA-RGCKVAMRLCSHNGTTCNFRNFSSATQAVMEWYTLQATNIFAQVPHQELVA    238
beta-ENaC_Platanista_gangetica                        APGRTCSA-HGCKVAMRLCSHSGTTCNFRNFSSATQAVTEWYTLQATNIFAQVPNQELVA    238
beta-ENaC_Platanista_minor                            APGRTCSA-HGCKVAMRLCSHSGTTCNFRNFSSATQAVTEWYTLQATNIFAQVPNQELVA    238
beta-ENaC_Kogia_breviceps                             APGRTCSA-HECKVAMRLCSHNGTMCNFRNFSSATQAVTEWYMLQATNIFAQVPNQELVA    238
beta-ENaC_Physeter_catodon                            APGRTCSA-HECKVAMRLCSHNGTMCNFRNFSSATQAVTEWYTLQATNIFAQVPNQELVA    238
beta-ENaC_Balaenoptera_musculus                       APGRTCSA-QGCKVAMRLCSHNGTTCNFRNFSSATQAVTEWYTLQATNIFAQVPKQELVA    238
beta-ENaC_Balaenoptera_acutorostrata_scammoni         APGRTCSA-QGCKVAMRLCSHNGTTCNFRNFSSATQAVTEWYTLQATNIFAQVPKQELVA    238
beta-ENaC_Eubalaena_japonica                          APERTCSA-QGCKVAMRLCSQNGTTCNFRNFSSATQAVTEWYTLQATNIFAQVPKQELVA    238
beta-ENaC_Hippopotamus_amphibius                      TPGKTCSAHHGCKVAMRLCSHNGTTCTFRNFSSATQAVTEWYTLQATNIFAQVPNQELVA    240
beta-ENaC_Tragulus_javanicus                          APGRPCSA-HRCKIAMRLCSHNGTACTFRNFSSATQAVTEWYTLQATNIFAQVPNQELVA    238
beta-ENaC_Tragulus_kanchil                            APGRPCSA-HRCKIAMRLCSHNGTACTFRNFSSATQAVTEWYTLQATNIFAQVPNQELVA    238
beta-ENaC_Antilocapra_americana                       APGRPCSA-HRCKVAMRLCSHNGTMCNFRNFSSATQAVMEWYTLQATNIFAQVPKQELVA    238
beta-ENaC_Giraffa_camelopardalis                      APGRPCSA-HRCKVAMRLCSHNGTTCTFRNFSSATQAVTEWYALQATNIFAQVPNQELVA    238
beta-ENaC_Giraffa_tippelskirchi                       APGRPCSA-HRCKVAMRLCSHNGTTCTFRNFSSATQAVTEWYALQATNIFAQVPNQELVA    238
beta-ENaC_Capreolus_pygargus                          APGRPCSA-HRCKVAIRLCSHNGTTCTFRNFSSATQAVTEWYTLQATNIFAQVPNPELVA    239
```

```
beta-ENaC_Cervus_elaphus                        APGRPCSA-HRCKVAMRLCSHNGTTCTFRNFSSATQAVTEWYTLQATNIFAQVPNQELVA    238
beta-ENaC_Moschus_moschiferus                   APGRPCSA-HRCKVAIRLCSHNGTTCTFRNFSSATQAVTEWYSLQATNIFAQVPNQELVA    238
beta-ENaC_Moschus_berezovskii                   APGRPCSA-HRCKVAIRLCSHNGTTCTFRNFSSATQAVTEWYSLQATNIFAQIPNQELVA    238
beta-ENaC_Bos_grunniens                         APGRPCSA-HRCKVAMRLCSHNGTTCTFRNFSSATQAVTEWYTLQATNIFAQVPNQELVA    238
beta-ENaC_Bos_taurus                            APGRPCSA-HRCKVAMRLCSHNGTTCTFRNFSSATQAVTEWYTLQATNIFAQVPNQELVA    238
beta-ENaC_Bubalus_bubalis                       APGRPCSA-HRCKVAMRLCSHNGTTCTFRNFSSATQAVTEWYTLQATNIFAQVPNQELVA    238
beta-ENaC_Nanger_granti                         APGRPCSA-HRCKVAMRLCSHNGTTCTFRNFSSATQAVTEWYTLQATNIFAQVPNQELVA    238
beta-ENaC_Capra_hircus                          APGRPCSA-HRCKVAMRLCSHNGTTCTFRNFSSATQAVTEWYTLQATNIFAQVPNQELVA    238
beta-ENaC_Ovis_aries                            APGRPCSA-RRCKVAMRLCSHNGTTCTFRNFSSATQAVTEWYTLQATNIFAQVPNQELVA    238
beta-ENaC_Ovis_canadensis                       APGRPCSA-RRCKVAMRLCSHNGTTCTFRNFSSATQAVTEWYTLQATNIFAQVPNQELVA    238
beta-ENaC_Hippotragus_niger                     APGRPCSA-HRCKVAMRLCSHNGTTCTFRNFSSATQAVTEWYTLQATNIFAQVPNQELVA    239
beta-ENaC_Sus_scrofa                            APERTCRA-PGCKIAMKLCSHNGTTCTFRSFSSATRAVTEWYTLQATNIFSQVPNKELVA    238
beta-ENaC_Vicugna_pacos                         APGRSCSA-HACKVAMRLCSRGGTLCTFRNFSSATQAVTEWYTLQATNIFSQVPRRELVT    238
beta-ENaC_Equus_callabus                        APGRTCDA-QGCKVAMRLCSLNGTVCTFRNFTSATQAVMEWYVLQATNIFSQVPRQELVA    238
                                                :  : * *   **:*::*** . * *.**.*:***:*: *** *******:*:*. ***

beta-ENaC_Homo_sapiens                          MSYPGEQMILACLFGAEPCNYRNFTSIFYPHYGNCYIFNWGMTEKALPSANPGTEFGLKL    297
beta-ENaC_Globicephala_melas                    MGYSAEQLILACLFGAEACTYRNFTRIFHPDYGNCYIFNWGMKEKALPSANPGAEFGLKL    298
beta-ENaC_Lagenorhynchus_obliquidens            MGYSAEQLILACLFGAEACTYRNFTRIFHPDYGNCYIFNWGMKEKALPSANPGAEFGLKL    298
beta-ENaC_Tursiops_truncatus                    MGYSAEQLILACLFGAEACTYRNFTRIFHPDYGNCYIFNWGMKEKALPSANPGAEFGLKL    298
beta-ENaC_Orcinus_orca                          MGYSAEQLILACLFGAEACTYRNFTRIFHPDYGNCYIFNWGMKEKALPSANPGAEFGLKL    298
beta-ENaC_Phocoena_sinus                        MGYSAEQLILACLFGAEACTYRNFTRIFHPDYGNCYIFNWGMKEKALPSANPGAEFGLKL    298
beta-ENaC_Neophocaena_asiaeorientalis           MGYSAEQLILACLFGAEACTYRNFTRIFHPDYGNCYIFNWGMKEKALPSANPGAEFGLKL    298
beta-ENaC_Monodon_monocerons                    MGYSAEQLILACLFGAEACTYRNFTPIFHPDYGNCYIFNWGMKEKALPSANPGAEFGLKL    299
beta-ENaC_Delphinapterus_leucas                 MGYSAEQLILACLFGAEACTYRNFTPIFHPDYGNCYIFNWGMKEKALPSANPGAEFGLKL    298
beta-ENaC_Pontoporia_blainvillei                MGYPAEQLILACLFGAEPCTYRNFTPIFHPDYGNCYIFNWGMKEKALPSANPGAEFGLKL    298
beta-ENaC_Inia_geoffrensis                      MGYPAEQLILACLFGAEPCTYRNFTPIFHPDYGNCYIFNWGMTEKALPSANPGAEFGLKL    298
beta-ENaC_Lipotes_vexillifer                    MGYPAEQLILACLFGAEPCTYRNFTPIFNPDYGNCYIFNWGMKEKALPSANPGDDFGLKL    298
beta-ENaC_Hyperoodon_ampullatus                 MGYPAEQLILACLFGAEPCNYRNFTHIFHPEYGNCYIFNWGMREKALPSANPGAEFGLKL    298
beta-ENaC_Mesoplodon_bidens                     MGYPAEQLILACLFGAEPCNYRNFTHIFHPEYGNCYIFNWGMREKALPSANPGAEFGLKL    298
beta-ENaC_Ziphius_cavirostris                   MGYPAERLILACLFGAEPCNYRNFTHIFHPEYGNCYIFNWGMREKALPSANPGAEFGLKL    298
beta-ENaC_Platanista_gangetica                  MGYPAERLILACLFGAEPCNYRNFTPIFHPDYGNCYIFNWGMTEKALPSANPGAEFGLKL    298
beta-ENaC_Platanista_minor                      MGYPAERLILACLFGAEPCNYRNFTPIFHPDYGNCYIFNWGMTEKALPSANPGAEFGLKL    298
beta-ENaC_Kogia_breviceps                       MGYPAERLILACLFGAEPCNYRNFTPIFDPDYGNCYIFNWGMTEKALPSANPGAEFGLKL    298
beta-ENaC_Physeter_catodon                      MGYPAERLILACLFGAEPCNYRNFTPIFDPDYGNCYIFNWGMTEKALPSANPGAEFGLKL    298
beta-ENaC_Balaenoptera_musculus                 MGYPAERLILACLFGAEPCNYRNFTPIFHPDYGNCYIFNWGMREKALPSANPGAEFGLKL    298
beta-ENaC_Balaenoptera_acutorostrata_scammoni   MGYPAERLILACLFGAEPCNYRNFTPIFHPDYGNCYIFNWGMREKALPSANPGAEFGLKL    298
beta-ENaC_Eubalaena_japonica                    MGYPAERLILACLFGAEPCNYRNFTPIFHPDYGNCYIFNWGMREKALPSANPGAEFGLKL    298
beta-ENaC_Hippopotamus_amphibius                MGYPAERLILACLFGAEPCNYRNFTPIFHPDYGNCYIFNWGMTEKALPSANPGAEFGLKL    300
beta-ENaC_Tragulus_javanicus                    MGYPAERLILACLFGAEPCNYRNFTRIFHPDYGNCYIFNWGMTEKALPSANPGTEFGLKL    298
beta-ENaC_Tragulus_kanchil                      MGYPAERLILACLFGAEPCNYRNFTRIFHPDYGNCYIFNWGMTEKALPSANPGTEFGLKL    298
beta-ENaC_Antilocapra_americana                 MGYPAERLILACLFGAEPCNYRNFTPIFHPDYGNCYIFNWGMREKALPSANPGTEFGLKL    298
beta-ENaC_Giraffa_camelopardalis                MGYPAERLILACLFGAEPCNYRNFTPIFHPDYGNCYIFNWGMTEKALPSANPGTEFGLKL    298
beta-ENaC_Giraffa_tippelskirchi                 MGYPAERLILACLFGAEPCNYRNFTPIFHPDYGNCYIFNWGMTEKALPSANPGTEFGLKL    298
beta-ENaC_Capreolus_pygargus                    MGYPAERLILACLFGAEPCNHRNFTPIFHPDYGNCYIFNWGMTEKALPSANPGPEFGLKL    299
beta-ENaC_Cervus_elaphus                        MGYPAERLILACLFGAEPCNYRNFTPIFHPDYGNCYIFNWGMTEKALPSANPGTEFGLKL    298
beta-ENaC_Moschus_moschiferus                   MGYPAERLILACLFGAEPCNYRNFTPIFHPDYGNCYIFNWGMTEKALPSANPGTEFGLKL    298
beta-ENaC_Moschus_berezovskii                   MGYPAERLILACLFGAEPCNYRNFTPIFHPDYGNCYIFNWGMTEKALPSANPGTEFGLKL    298
beta-ENaC_Bos_grunniens                         MGYPAERLILACLFGAEPCNYRNFTPIFHPDYGNCYIFNWGMTEKALPSANPGTEFGLKL    298
beta-ENaC_Bos_taurus                            MGYPAERLILACLFGAEPCNYRNFTPIFHPDYGNCYIFNWGMTEKALPSANPGTEFGLKL    298
beta-ENaC_Bubalus_bubalis                       MGYPAERLILACLFGAEPCNYRNFTPIFHPDYGNCYIFNWGMTEKALPSANPGTEFGLKL    298
beta-ENaC_Nanger_granti                         MGYPAERLILACLFGAEPCNYRNFTPIFHPDYGNCYIFNWGMTEKALPSANPGTEFGLKL    298
beta-ENaC_Capra_hircus                          MGYPAERLILACLFGAEPCNYRNFTPIFHPDYGNCYIFNWGMTEKALPSANPGTEFGLKL    298
beta-ENaC_Ovis_aries                            MGYPAERLILACLFGAEPCNYRNFTPIFHPDYGNCYIFNWGMTEKALPSANPGTEFGLKL    298
beta-ENaC_Ovis_canadensis                       MGYPAERLILACLFGAEPCNYRNFTPIFHPDYGNCYIFNWGMTEKALPSANPGTEFGLKL    298
beta-ENaC_Hippotragus_niger                     MGYPAERLILACLFGAEPCNYRNFTPIFHPDYGNCYIFNWGMTEKALPSANPGTEFGLKL    299
beta-ENaC_Sus_scrofa                            MGYPAERMILACLFGAEPCNYRNFTPIFHPDYGNCYIFNWGMKEKALPSANPGAEFGLKL    298
beta-ENaC_Vicugna_pacos                         MGYSGEQLILACLFGAEPCSYRNFTPIFHPDYGNCYIFNWGMTEKALLSSNPGAEFGLKL    298
beta-ENaC_Equus_callabus                        MSYPGEQLILACLFGAEPCSHRNFTSIFHPDYGNCHIFNWGMTEKALPSSNPGAEFGLKL    298
                                                *.* .*::********* *.:**** ** *.****:****** **** *:*** :*****

beta-ENaC_Homo_sapiens                          ILDIGQEDYVPFLASTAGVRLMLHEQRSYPFIRDEGIYAMSGTETSIGVLVDKLQRMGEP    357
beta-ENaC_Globicephala_melas                    ILDVGQEDYVPFLTSTAGARLMLHEQMSYPFIKEEGIFAMSGMETSIGVLVDKLERKGEP    358
beta-ENaC_Lagenorhynchus_obliquidens            ILDVGQEDYVPFLTSTAGARLMLHEQMSYPFIKEEGIFAMSGMETSIGVLVDKLERKGEP    358
beta-ENaC_Tursiops_truncatus                    ILDVGQEDYVPFLTSTAGARLMLHEQMSYPFIKEEGIFAMSGMETSIGVLVDKLERKGEP    358
beta-ENaC_Orcinus_orca                          ILDVGQEDYVPFLTSTAGARLMLHEQMSYPFIKEEGIFAMSGMETSIGVLVDKLERKGEP    358
beta-ENaC_Phocoena_sinus                        ILDVGQEDYVPFLTSTAGARLMLHEQMSYPFIKEEGIFAMSGMETSIGVLVDKLERKGEP    358
beta-ENaC_Neophocaena_asiaeorientalis           ILDVGQEDYVPFLTSTAGARLMLHEQMSYPFIKEEGIFAMSGMETSIGVLVDKLERKGEP    358
beta-ENaC_Monodon_monocerons                    ILDVGQEDYVPFLTSTAGARLMLHEQMSYPFIKEEGIFAMSGMETSIGVLVDKLERKGEP    359
beta-ENaC_Delphinapterus_leucas                 ILDVGQEDYVPFLTSTAGARLMLHEQMSYPFIKEEGIFAMSGMETSIGVLVDKLERKGEP    358
beta-ENaC_Pontoporia_blainvillei                ILDVDQEDYVPFLTSTAGARLMLHEQMSYPFIKEEGIFAMSGMETSIGVLVDKLERKGEP    358
beta-ENaC_Inia_geoffrensis                      ILDVGQEDYVPFLTSTAGARLMLHEQMSYPFIKEEGIFAMSGMETSIGVLVDKLERKGEP    358
beta-ENaC_Lipotes_vexillifer                    ILDVGQEDYVPFLTSTAGARLMLHEQMSYPFIKEEGIFAMSGMETSIGVLVDKLERKGEP    358
beta-ENaC_Hyperoodon_ampullatus                 ILDMGQEDYVPFLTTTAGARLMLHEQMSYPFIKEEGIFAMSGMETSIGVLVDKLERKGEP    358
beta-ENaC_Mesoplodon_bidens                     ILDMGQEDYVPFLTTTAGARLMLHEQMSYPFIKEEGIFAMSGMETSIGVLVDKLERKGEP    358
beta-ENaC_Ziphius_cavirostris                   ILDMGQEDYVPFLTTTAGARLMLHEQMSYPFIKEEGIFAMSGMETSIGVLVDKLERKGEP    358
beta-ENaC_Platanista_gangetica                  ILDVGQEDYVPFLTPTAGARLMLHEQMSYPFIKEEGIFAMSGMETSIGVLVDKLERKGEP    358
beta-ENaC_Platanista_minor                      ILDVGQEDYVPFLTPTAGARLMLHEQMSYPFIKEEGIFAMSGMETSIGVLVDKLERKGEP    358
beta-ENaC_Kogia_breviceps                       ILDMGQEDYVPFLTSTAGARLMLHEQMSYPFIKEEGIFAMSGMETSIGVLVDKLERKGEP    358
beta-ENaC_Physeter_catodon                      ILDMGQEDYVPFLTSTAGARLMLHEQMSYPFIKEEGIFAMSGMETSIGVLVDKLERKGEP    358
beta-ENaC_Balaenoptera_musculus                 ILDMGQEDYVPFLTSTAGARLMLHEQMSYPFIKEEGIFAMSGMETSIGVLVDRLERKGEP    358
beta-ENaC_Balaenoptera_acutorostrata_scammoni   ILDMGQEDYVPFLTSTAGARLMLHEQMSYPFIKEEGIFAMSGMETSIGVLVDRLERKGEP    358
beta-ENaC_Eubalaena_japonica                    ILDMGQEDYVPFLTSTAGARLMLHEQMSYPFIKEEGIFAMSGMETSIGVLVDRLERKGEP    358
beta-ENaC_Hippopotamus_amphibius                ILDMGQEDYMPFLTSTAGARLMLHEQRSYPFIKEEGIYAMSGMETSIGVLVDKLERKGEP    360
beta-ENaC_Tragulus_javanicus                    ILDMGQEDYVPYLTPTAGARLLLHEQRSYPFIKEEGIYAMAGMETSIGVLVDKLQRKGKP    358
beta-ENaC_Tragulus_kanchil                      ILDMGQEDYVPYLTPTAGARLLLHEQRSYPFIKEEGIYAMAGMETSIGVLVDKLQRKGKP    358
beta-ENaC_Antilocapra_americana                 ILDMGQEDYVPFLTSTAGARLMLHEQRSYPFIKEEGIYAMAGMETSIGVLVDKLQRKGKP    358
beta-ENaC_Giraffa_camelopardalis                ILDMGQEDYVPFLTSTAGARLMLHEQRSYPFIKEEGIYAMVGMETSIGVLVDKLQRKGEP    358
beta-ENaC_Giraffa_tippelskirchi                 ILDMGQEDYVPFLTSTAGARLMLHEQRSYPFIKEEGIYAMVGMETSIGVLVDKLQRKGEP    358
beta-ENaC_Capreolus_pygargus                    ILDMGQEDYVPFLTSTAGARLMLHEQRSYPFIKEEGIYAMAGMETSIGVLVDKLQRKGEP    359
beta-ENaC_Cervus_elaphus                        ILDMGQEDYVPFLTSTAGARLMLHEQRSYPFIKEEGIYAMAGMETSIGVLVDKLQRKGKP    358
```

```
beta-ENaC_Moschus_moschiferus                         ILDMGQEDYVPFLTSTAGARLMLHEQRSYPFIKEEGIYAMAGMETSIGVLVDKLQRKGEP    358
beta-ENaC_Moschus_berezovskii                         ILDMGQEDYVPFLTSTAGARLMLHEQRSYPFIKEEGIYAMAGMETSIGVLVDKLQRKGEP    358
beta-ENaC_Bos_grunniens                               ILDMGQEDYVPFLTSTAGARLMLHEQRSYPFIKEEGIYAMAGMETSIGVLVDKLQRKGEP    358
beta-ENaC_Bos_taurus                                  ILDMGQEDYVPFLTSTAGARLMLHEQRSYPFIKEEGIYAMAGMETSIGVLVDKLQRKGEP    358
beta-ENaC_Bubalus_bubalis                             ILDMGQEDYVPFLTSTAGARLMLHEQRSYPFIKEEGIYAMAGMETSIGVLVDKLQRKGEP    358
beta-ENaC_Nanger_granti                               ILDMGQEDYVPFLTSTAGARLMLHEQRSYPFIKEEGIYAMAGMETSIGVLVDKLQRKGEP    358
beta-ENaC_Capra_hircus                                ILDMGQEDYVPFLTSTAGARLMLHEQRSYPFIKEEGIYAMAGMETSIGVLVDKLQRKGEP    358
beta-ENaC_Ovis_aries                                  ILDMGQEDYVPFLTSTAGARLMLHEQRSYPFIKEEGIYAMAGMETSIGVLVDKLQRKGEP    358
beta-ENaC_Ovis_canadensis                             ILDMGQEDYVPFLTSTAGARLMLHEQRSYPFIKEEGIYAMAGMETSIGVLVDKLQRKGEP    358
beta-ENaC_Hippotragus_niger                           ILDMGQEDYVPFLTSTAGARLMLHEQRSYPFIKEEGIYAMAGMETSIGVLVDKLQRKGEP    359
beta-ENaC_Sus_scrofa                                  ILDMGQKDYVPFLTSTGGVRLMLHEQRSFPFIKDEGIYAMSGTETSIGVLVDKLERKGEP    358
beta-ENaC_Vicugna_pacos                               ILDMGQEDYVPFLTSTAGTRLMLHEQRSYPFIKDEGIYAMSGTETSIGVMVDRLQRKGEP    358
beta-ENaC_Equus_callabus                              ILDIGQEDYVPFLTSTAGARLLLHEQRSYPFIKEEGIYAMSGTETSIGVLVDRLERKGEP    358
                                                      ***:.*:**:*:*: *.*.**:**** *:***::***:** * ******:**:*:* *:*

beta-ENaC_Homo_sapiens                                YSPCTVNGSEVPVQNFYS---DYNTTYSIQACLRSCFQDHMIRNCNCGHYLYPLPRGEKY    414
beta-ENaC_Globicephala_melas                          YSQCTVNGSDVPIRNLYS---DYNTTYSIQACIRSCFQDHMIRNCSCGHYLYPLPRGEKY    415
beta-ENaC_Lagenorhynchus_obliquidens                  YSQCTVNGSDVPIRNLYS---DYNTTYSIQACIRSCFQDHMIRNCSCGHYLYPLPRGEKY    415
beta-ENaC_Tursiops_truncatus                          YSQCTVNGSDVPIRNLYS---DYNTTYSIQACIRSCFQDHMIRNCSCGHYLYPLPRGEKY    415
beta-ENaC_Orcinus_orca                                YSQCTVNGSDVPIRNLYS---DYNTTYSIQACIRSCFQDHMIRNCSCGHYLYPLPRGEKY    415
beta-ENaC_Phocoena_sinus                              YSQCTVNGSDVPIRNLYS---DYNTTYSIQACIRSCFQDHMIRNCSCGHYLYPLPRGEKY    415
beta-ENaC_Neophocaena_asiaeorientalis                 YSQCTVNGSDVPIRNLYS---DYNTTYSIQACIRSCFQDHMIRNCSCGHYLYPLPRGEKY    415
beta-ENaC_Monodon_monocerons                          YSQCTVNGSDVPIRNLYS---DYNTTYSIQACIRSCFQDHMIRNCSCGHYLYPLPRGEKY    416
beta-ENaC_Delphinapterus_leucas                       YSQCTVNGSDVPIRNLYS---DYNTTYSIQACIRSCFQDHMIRNCSCGHYLYPLPRGEKY    415
beta-ENaC_Pontoporia_blainvillei                      YSQCTVNGSDVPIRNLYS---DYNTTYSIQACIRSCFQDHMIHNCSCGHYLYPLPHGEKY    415
beta-ENaC_Inia_geoffrensis                            YSQCTVNGSDVPIRNLYS---DYNTTYSIQACIRSCFQDHMIHNCSCGHYLYPLPHGEKY    415
beta-ENaC_Lipotes_vexillifer                          YSQCTVNGSDVPIRNLYS---DYNTTYSIQACIRSCFQDHMIHNCSCGHYLYPLPRGEKY    415
beta-ENaC_Hyperoodon_ampullatus                       YSQCTMNGSDVPIRNLYS---DYNTTYSIQACIGSCFQDHMIHNCSCGHYLYPLPRGEKY    415
beta-ENaC_Mesoplodon_bidens                           YSQCTMNGSDVPIRNLYS---DYNTTYSIQACIGSCFQDHMIHNCSCGHYLYPLPRGEKY    415
beta-ENaC_Ziphius_cavirostris                         YSQCTMNGSDVPIRNLYS---DYNTTYSIQACIGSCFQDHMIHNCSCGHYLYPLPRGEKY    415
beta-ENaC_Platanista_gangetica                        YSQCTMNGSDVPIRNLYS---DYNTTYSIQACIRSCFQDHMIRNCSCGHYLYPLPRGEKY    415
beta-ENaC_Platanista_minor                            YSQCTMNGSDVPIRNLYS---DYNTTYSIQACIRSCFQDHMIRNCSCGHYLYPLPRGEKY    415
beta-ENaC_Kogia_breviceps                             YSQCTVNGSDVPIRNLYS---DYNTTYSIQACIRSCFQDHMIHNCSCGHYLYPLPRGEKY    415
beta-ENaC_Physeter_catodon                            YSQCTVNGSDVPIRNLYS---DYNTTYSIQACIRSCFQDHMIHNCSCGHYLYPLPRGEKY    415
beta-ENaC_Balaenoptera_musculus                       YSQCTMNGSDVPIRNLYS---DYNTTYSIQACIRSCFQDHMIHNCSCGHYLYPLPRGEKY    415
beta-ENaC_Balaenoptera_acutorostrata_scammoni         YSQCTMNGSDVPIRNLYS---DYNTTYSIQACIRSCFQDHMIRNCSCGHYLYPLPRGEKY    415
beta-ENaC_Eubalaena_japonica                          YSQCTMNGSDVPIRNLYS---DYNTTYSIQACIRSCFQDHMIHNCSCGHYLYPLPRGEKY    415
beta-ENaC_Hippopotamus_amphibius                      YSQCTMNGSDVPIQNLYSS--DYNTTYSIQACIRSCFQDHMIRNCSCGHYLYPLPRGQKY    418
beta-ENaC_Tragulus_javanicus                          YSQCTKNGSDVPVPNLYS---NYNTTYSIQACIRSCFQDRMIRDCGCGHYLYPLPPGRRY    415
beta-ENaC_Tragulus_kanchil                            YSQCTKNGSDVPVPNLYS---NYNTTYSIQACIRSCFQDRMIRDCGCGHYLYPLPPGKRY    415
beta-ENaC_Antilocapra_americana                       YSQCTKNGSDVPIQNLYS---GYNTTYSIQACIRSCFQEHMIRECGCGHYLYPLPHEEKY    415
beta-ENaC_Giraffa_camelopardalis                      YSQCTKNGSDVPIKNLYS---NYNTTYSIQACIRSCFQEYMIRECGCGHYLYPLPHEKKY    415
beta-ENaC_Giraffa_tippelskirchi                       YSQCTKNGSDVPIKNLYS---NYNTTYSIQACIRSCFQEYMIRECGCGHYLYPLPHEKKY    415
beta-ENaC_Capreolus_pygargus                          YSQCTKNGSDVPVQNLYS---SYNTTYSIQACIRSCFQEHMIRECGCGHYLYPLPHKSKY    416
beta-ENaC_Cervus_elaphus                              YSQCTKNGSDVPIPNLYS---SYNTTYSIQACIRSCFQEHMIRECGCGHYLYPLPHKRKY    415
beta-ENaC_Moschus_moschiferus                         YSQCTKNGSDVPIQNLYS---NYNTTYSIQACIRSCFQEHMIRECGCGHYLYPLPRKRKY    415
beta-ENaC_Moschus_berezovskii                         YSQCTKNGSDVPIQNLYS---NYNTTYSIQACIRSCFQEHMIRECGCGHYLYPLPRKRKY    415
beta-ENaC_Bos_grunniens                               YSQCTKNGSDVPIQNLYS---NYNTTYSIQACIRSCFQEHMIRECGCGHYLYPLPHKRKY    415
beta-ENaC_Bos_taurus                                  YSQCTKNGSDVPIQNLYS---NYNTTYSIQACIRSCFQEHMIRECGCGHYLYPLPHKRKY    415
beta-ENaC_Bubalus_bubalis                             YSQCTKNGSDVPIQNLYS---SYNTTYSIQACIRSCFQEHMIRECGCGHYLYPLPHKRKY    415
beta-ENaC_Nanger_granti                               YSQCTKNGSDVPIQNLYS---NYNTTYSIQACIRSCFQEHMIRECGCGHYLYPLPHKRKY    415
beta-ENaC_Capra_hircus                                YSQCTKNGSDVPIQNLYS---SYNTTYSIQACIRSCFQEHMIRECGCGHYLYPLPDKKKY    415
beta-ENaC_Ovis_aries                                  YSQCTKNGSDVPIQNLYS---SYNTTYSIQACIRSCFQEHMIRECGCGHYLYPLPDKRKY    415
beta-ENaC_Ovis_canadensis                             YSQCTKNGSDVPIQNLYS---SYNTTYSIQACIRSCFQEHMIRECGCGHYLYPLPDKRKY    415
beta-ENaC_Hippotragus_niger                           YSQCTKNGSDVPIQNLYS---SYNTTYSIQACIRSCFQEHMIRECGCGHYLYPLPHKRKY    416
beta-ENaC_Sus_scrofa                                  YSQCTMNGSDVPIQNLYSNYGDYNTTYSIQACIRSCFQEHMIRHCSCGHYLYPLPPGEKY    418
beta-ENaC_Vicugna_pacos                               YSQCTVNGSDVPIRNLYS---YYNTTYSIQACIRSCFQDHMITKCSCGHYLYPLPRGEKY    415
beta-ENaC_Equus_callabus                              YSRCTKNGSDVPIPNLYS---DHNTTYSIQACIHSCFQDHMIRNCSCGHYLYPLPRGEKY    415
                                                      ** ** ***:**: *:**    :*********: ****: ** .*.*********   :*

beta-ENaC_Homo_sapiens                                CNNRDFPDWAHCYSDLQMSVAQRETCIGMCKES NDTQYKMTISMADWPSEASEDWIFHV    474
beta-ENaC_Globicephala_melas                          CNNQEFPDWAYCYSALRTSLAQRETCIDVCKESCNDTQYKMTISMAVWPSESSEDWIFHV    475
beta-ENaC_Lagenorhynchus_obliquidens                  CNNQEFPDWAYCYSALRTSLAQRETCIDVCKESCNDTQYKMTISMAVWPSESSEDWIFHV    475
beta-ENaC_Tursiops_truncatus                          CNNQEFPDWAYCYSALRTSLAQRETCIDVCKESCNDTQYKMTISMAVWPSESSEDWIFHV    475
beta-ENaC_Orcinus_orca                                CNNQEFPDWAYCYSALRTSLAQRETCIDVCKESCNDTQYKMTISMAVWPSESSEDWIFHV    475
beta-ENaC_Phocoena_sinus                              CNNQEFPDWAYCYSALRMSLAQRETCIDVCKESCNDTQYKMTISMAVWPSESSEDWIFHV    475
beta-ENaC_Neophocaena_asiaeorientalis                 CNNQEFPDWAYCYSALRMSLAQRETCIDVCKESCNDTQYKMTISMAVWPSESSEDWIFHV    475
beta-ENaC_Monodon_monocerons                          CNNQEFPDWAYCYSALRMSLAQRETCIDVCKESCNDTQYKMTISMAVWPSESSEDWIFHV    476
beta-ENaC_Delphinapterus_leucas                       CNNQEFPDWAYCYSALRMSLAQRETCIDVCKESCNDTQYKMTISMAVWPSESSEDWIFHV    475
beta-ENaC_Pontoporia_blainvillei                      CNNQEFPDWAYCYSALRMSLAQRETCIDVCKESCNDTQYKMTISMAVWPSEASEDWIFHV    475
beta-ENaC_Inia_geoffrensis                            CNNQEFPDWAYCYSALRMSLAQRETCIDVCKESCNDTQYKMTISMAAWPSEASEDWIFHV    475
beta-ENaC_Lipotes_vexillifer                          CNNQEFPDWAYCYSALRMSLAQRETCIDVCKESCNDTQYKMTISMAVWPSEASEDWIFHV    475
beta-ENaC_Hyperoodon_ampullatus                       CNNREFPDWVYCYSALRMSLAQRETCIDICKESCNDTQYKMTISMAVWPSESSEDWIFHV    475
beta-ENaC_Mesoplodon_bidens                           CNNRDFPDWVYCYSALRMSLAQRETCIDICKESCNDTQYKMTISMAVWPSESSEDWIFHV    475
beta-ENaC_Ziphius_cavirostris                         CNNREFPDWVYCYSALRMSLAQRETCIDICKESCNDTQYKMTISMAVWPSESSEDWIFHV    475
beta-ENaC_Platanista_gangetica                        CNNQEFPDWAYCYSALRVSLAQRETCIDVCKESCNDTQYKMTISMAVWPSEASEDWIFHV    475
beta-ENaC_Platanista_minor                            CNNQEFPDWAYCYSALRVSLAQRETCIDVCKESCNDTQYKMTISMAVWPSEASEDWIFHV    475
beta-ENaC_Kogia_breviceps                             CNNQEFPDWAYCYSALRMSLAQRETCIDLCKESCNDTQYKMTISMAVWPSEASEDWIFHV    475
beta-ENaC_Physeter_catodon                            CNNQEFPDWAYCYSALRMSLAQRETCIDVCKESCNDTQYKMTISMAVWPSEASEDWIFHV    475
beta-ENaC_Balaenoptera_musculus                       CNNQEFPDWAYCYSALRMSLAQRETCIDVCKESCNDTQYKMTISMAVWPSEASEDWIFHV    475
beta-ENaC_Balaenoptera_acutorostrata_scammoni         CNNQEFPDWAYCYSALRMSLAQRETCIDVCKESCNDTQYKMTISMAVWPSEASEDWIFHV    475
beta-ENaC_Eubalaena_japonica                          CNNQEFPDWAYCYSALRMSLAQRETCIDVCKESCNDTQYKMTISMAVWPSEASEDWIFHV    475
beta-ENaC_Hippopotamus_amphibius                      CSNQEFPDWALCYSALRMSLAQRETCIDMCKESCNDTQYKMTISMAVWPSEASEDWIFHV    478
beta-ENaC_Tragulus_javanicus                          CHSQEFPDWAHCYSALRISMEQREDCIYTCKESCNDTQYKMTISMAVWPSEASEDWILHV    475
beta-ENaC_Tragulus_kanchil                            CHSQEFPDWAHCYSALRISMEQREDCIYTCKESCNDTQYKMTISMAVWPSEASEDWILHV    475
beta-ENaC_Antilocapra_americana                       CNNQEFPDWAHCYSALRISMAQRETCIYACKESCNDTQYKMTISMAVWPSEASEDWIFHV    475
beta-ENaC_Giraffa_camelopardalis                      CNNQDFPDWAHCYSALRISLAQRETCIYACKESCNDTQYKMTISMAVWPSEASEDWIFHV    475
beta-ENaC_Giraffa_tippelskirchi                       CNNQDFPDWAHCYSALRISLAQRETCIYACKESCNDTQYKMTISMAVWPSEASEDWIFHV    475
beta-ENaC_Capreolus_pygargus                          CNNQEFPDWAHCYSALRISMAQREACIYACKESCNDTQYKMTISMAVWPSEASEDWIFHV    476
beta-ENaC_Cervus_elaphus                              CNNQEFPDWAHCYSALRISMAQREACIYACKESCNDTQYKMTISMAVWPSEASEDWIFHV    475
beta-ENaC_Moschus_moschiferus                         CSNQEFPDWAHCYSALRISMAQRETCIYACKESCNDTQYKMTISMAVWPSEASEDWIFHV    475
```

```
beta-ENaC_Moschus_berezovskii                      CSNQEFPDWAHCYSALRISMAQRETCIYACKESCNDTQYKMTISMAVWPSEASEDWIFHV    475
beta-ENaC_Bos_grunniens                            CNNQEFPDWAHCYSALRISLAQRETCIYACKESCNDTQYKMTISMAVWPSEASEDWIFHV    475
beta-ENaC_Bos_taurus                               CNNQEFPDWAHCYSALRISLAQRETCIYACKESCNDTQYKMTISMAVWPSEASEDWIFHV    475
beta-ENaC_Bubalus_bubalis                          CNNQEFPDWAHCYSALRISMAQRETCIYACKESCNDTQYKMTISMAVWPSEASEDWIFHV    475
beta-ENaC_Nanger_granti                            CNNQEFPDWAHCYSALRISMAQRETCIYTCKESCNDTQYKMTISMAVWPSEASEDWIFHV    475
beta-ENaC_Capra_hircus                             CNNQEFPDWAHCYSALRISMAQRETCIYACKESCNDTQYKMTISMAVWPSEASEDWIFQV    475
beta-ENaC_Ovis_aries                               CNNQEFPDWAHCYSALRISMAQRETCIYACKESCNDTQYKMTISMAVWPSEASEDWIFHV    475
beta-ENaC_Ovis_canadensis                          CNNQEFPDWAHCYSALRISMAQRETCIYACKESCNDTQYKMTISMAVWPSEASEDWIFHV    475
beta-ENaC_Hippotragus_niger                        CNNQEFPDWAHCYSALRVSMAQREACIYTCKESCNDTQYKMTISMAVWPSEASEDWIFHV    476
beta-ENaC_Sus_scrofa                               CNSQEFPDWAYCYSDLRMSLAQRETCIDVCKESCNDTQYKMTISMADWPSESSEDWIFHV    478
beta-ENaC_Vicugna_pacos                            CSNREFPDWAYCYSALRMSLAQRESCIDVCKESCNDTQYKMTISMADWPSEASEDWIFHV    475
beta-ENaC_Equus_callabus                           CNNQDFPDWAYCYSDLRINVAQRETCINLCKESCNDTQYKMTISMAEWPSEASEDWILHV    475
                                                   * .::****. *** *: .: *** **  ***************** ****:*****::*

                                                                                       TM2
beta-ENaC_Homo_sapiens                             LSQERDQSTNITLSRKGIVKLNIYFQEFNYRTIEESAANNIVWLLSNLGGQFGFWMGGSV    534
beta-ENaC_Globicephala_melas                       LSEERDQSPNITMNRKGVIKLNIYFQEYNYRTIEESAANNIVWLLSNLGGQFGFWMGGSV    535
beta-ENaC_Lagenorhynchus_obliquidens               LSEERDQSPNITMNRKGVIKLNIYFQEYNYRTIEESAANNIVWLLSNLGGQFGFWMGGSV    535
beta-ENaC_Tursiops_truncatus                       LSEERDQSPNITMNRKGVIKLNIYFQEYNYRTIEESAANNIVWLLSNLGGQFGFWMGGSV    535
beta-ENaC_Orcinus_orca                             LSEERDQSPNITMNRKGVIKLNIYFQEYNYRTIEESAANNIVWLLSNLGGQFGFWMGGSV    535
beta-ENaC_Phocoena_sinus                           LSEERDQSPNITLNRKGVIKLNIYFQEYNYRTIEESAANNIVWLLSNLGGQFGFWMGGSV    535
beta-ENaC_Neophocaena_asiaeorientalis              LSEERDQSPNITLNRKGVIKLNIYFQEYNYRTIEESAANNIVWLLSNLGGQFGFWMGGSV    535
beta-ENaC_Monodon_monocerons                       LSEERDQSPNITLNRKGIIKLNIYFQEYNYRTIEESAANNIVWLLSNLGGQFGFWMGGSV    536
beta-ENaC_Delphinapterus_leucas                    LSEERDQSPNITLNRKGIIKLNIYFQEYNYRTIEESAANNIVWLLSNLGGQFGFWMGGSV    535
beta-ENaC_Pontoporia_blainvillei                   LSEERDQSPNITLNRKGIIKLNIYFQEYNYRTIEESEANNIVWLLSNLGGQFGFWMGGSV    535
beta-ENaC_Inia_geoffrensis                         LSEERDQSPNITLNRKGIIKLNIYFQEYNYRTIEESAANNIVWLLSNLGGQFGFWMGGSV    535
beta-ENaC_Lipotes_vexillifer                       LSEERDQSPNITLNRKGIIKLNIYFQEYNYRTIEESAANNIVWLLSNLGGQFGFWMGGSV    535
beta-ENaC_Hyperoodon_ampullatus                    LSQERDQSTNITLNRKGVIKLNIYFQEYNYRTIAESAANNIVWLLSNLGGQFGFWMGGSV    535
beta-ENaC_Mesoplodon_bidens                        LSQERDQSTNITLNRKGVIKLNIYFQEYNYRTIAESAANNIVWLLSNLGGQFGFWMGGSV    535
beta-ENaC_Ziphius_cavirostris                      LSQERDHSTNITLNRKGVIKLNIYFQEYNYRTIAESAANNIVWLLSNLGGQFGFWMGGSV    535
beta-ENaC_Platanista_gangetica                     LSQERDQSTNITLNRKGIIKLNIYFQEYNYRTIEESAANNIVWLLSNLGGQFGFWMGGSV    535
beta-ENaC_Platanista_minor                         LSQERDQSTNITLNRKGIIKLNIYFQEYNYRTIEESAANNIVWLLSNLGGQFGFWMGGSV    535
beta-ENaC_Kogia_breviceps                          LSQERDQSTNITLNRKGIIKLNIYFQEYNYRTIEESAANNIVWLLSNLGGQFGFWMGGSV    535
beta-ENaC_Physeter_catodon                         LSQERDQSTNITLNRKGIIKLNIYFQEYNYRTIEESAANNIVWLLSNLGGQFGFWMGGSV    535
beta-ENaC_Balaenoptera_musculus                    LSQERDQSTNITLNRKGIIKLNIYFQEYNYRTIEESAANNIVWLLSNLGGQFGFWMGGSV    535
beta-ENaC_Balaenoptera_acutorostrata_scammoni      LSQERDQSTNITLNRKGIIKLNIYFQEYNYRTIEESAANNIVWLLSNLGGQFGFWMGGSV    535
beta-ENaC_Eubalaena_japonica                       LSQERDQSTNITLNRKGIIKLNIYFQEYNYRTIEESAANNIVWLLSNLGGQFGFWMGGSV    535
beta-ENaC_Hippopotamus_amphibius                   LSQERDQSTNITLSRKGIVKLNIYFQEFNYRTIGESAANNIVWLLSNLGGQFGFWMGGSV    538
beta-ENaC_Tragulus_javanicus                       LSQERDQSTNITISRKGIVKLNIYFQEFNYRTIEESAANNIVWLLSNLGGQFGFWMGGSV    535
beta-ENaC_Tragulus_kanchil                         LSQERDQSTNITISRKGIVKLNIYFQEFNYRTIEESAANNIVWLLSNLGGQFGFWMGGSV    535
beta-ENaC_Antilocapra_americana                    LSQERDQSSNITLSRKGIVKLNIYFQEFNYRTIEESAANNIVWLLSNLGGQFGFWMGGSV    535
beta-ENaC_Giraffa_camelopardalis                   LSQERDQSSNITLSRKGIVKLNIYFQEFNYRTIEESAANNIVWLLSNLGGQFGFWMGGSV    535
beta-ENaC_Giraffa_tippelskirchi                    LSQERDQSSNITLSRKGIVKLNIYFQEFNYRTIEESAANNIVWLLSNLGGQFGFWMGGSV    535
beta-ENaC_Capreolus_pygargus                       LSQERDQSSNITLSRKGIVKLNIYFQEFNYRTIEESAANNIVWLLSNLGGQFGFWMGGSV    536
beta-ENaC_Cervus_elaphus                           LSQERDQSSNITLSRKGIVKLNIYFQEFNYRTIEESAANNIVWLLSNLGGQFGFWMGGSV    535
beta-ENaC_Moschus_moschiferus                      LSQERDQSSNITLSRKGIVKLNIYFQEFNYRTIEESAANNIVWLLSNLGGQFGFWMGGSV    535
beta-ENaC_Moschus_berezovskii                      LSQERDQSSNITLSRKGIVKLNIYFQEFNYRTIEESAANNIVWLLSNLGGQFGFWMGGSV    535
beta-ENaC_Bos_grunniens                            LSQERDQSSNITLSRKGIVKLNIYFQEFNYRTIEESAANNIVWLLSNLGGQFGFWMGGSV    535
beta-ENaC_Bos_taurus                               LSQERDQSSNITLSRKGIVKLNIYFQEFNYRTIEESAANNIVWLLSNLGGQFGFWMGGSV    535
beta-ENaC_Bubalus_bubalis                          LSQERDQSSNITLSRKGIVKLNIYFQEFNYRTIEESAANNIVWLLSNLGGQFGFWMGGSV    535
beta-ENaC_Nanger_granti                            LSQERDQSSNITLSRKGIVKLNIYFQEFNYRTIEESAANNIVWLLSNLGGQFGFWMGGSV    535
beta-ENaC_Capra_hircus                             LSQERDQSSNITLSRKGIVKLNIYFQEFNYRTIEESAANNIVWLLSNLGGQLGFWMGGSV    535
beta-ENaC_Ovis_aries                               LSQERDQSSNITLSRKGIVKLNIYFQEFNYRTIEESAANNIVWLLSNLGGQFGFWMGGSV    535
beta-ENaC_Ovis_canadensis                          LSQERDQSSNITLSRKGIVKLNIYFQEFNYRTIEESAANNIVWLLSNLGGQFGFWMGGSV    535
beta-ENaC_Hippotragus_niger                        LSQERDQSSNITLSRKGIVKLNIYFQEFNYRTIEESAANNIVWLLSNLGGQFGFWMGGSV    536
beta-ENaC_Sus_scrofa                               LSEERDQSTNITLSRQGIVKLNIYFQEFNYRTIEESAANNIVWLLSNLGGQFGFWMGGSV    538
beta-ENaC_Vicugna_pacos                            LSQERDQSTNITLSRKGIVKLNIYFQEFNYRTIEESAANNIVWLLSNLGGQFGFWMGGSV    535
beta-ENaC_Equus_callabus                           LSQERDQSTNITLSRKGIVKLNIYFQEFNYRTIEESAANNIVWLLSNLGGQFGFWMGGSV    535
                                                   **:***:* ***:.*:*::********:***** ** ***************:********

beta-ENaC_Homo_sapiens                             LCLIEFGEIIIDFVWITIIKLVALAKSLRQRRAQASYAGPPPTVAELVEAHTNFGFQPDT    594
beta-ENaC_Globicephala_melas                       LCLIEFAEIIIDFVWITIIKLVALAKSLRRRRTQARYDGPPPTVAELVEAHTNFGFQPDT    595
beta-ENaC_Lagenorhynchus_obliquidens               LCLIEFAEIIIDFVWITIIKLVALAKSLRRRRTQARYDGPPPTVAELVEAHTNFGFQPDT    595
beta-ENaC_Tursiops_truncatus                       LCLIEFAEIIIDFVWITIIKLAALAKSLRRRRTQARYDGPPPTVAELVEAHTNFGFQPDT    595
beta-ENaC_Orcinus_orca                             LCLIEFAEIIIDFVWITIIKLVALAKSLRRKRTQARYDGPPPTVAELVEAHTNFGFQPDT    595
beta-ENaC_Phocoena_sinus                           LCLIEFAEIIIDFVWITIIKLVALAKSLRRRRAQTCYDGPPPTVAELVEAHTNFGFQPDT    595
beta-ENaC_Neophocaena_asiaeorientalis              LCLIEFAEIIIDFVWITIIKLVALAKSLRRRRAQACYDGPPPTVAELVEAHTNFGFQPDT    595
beta-ENaC_Monodon_monocerons                       LCLIEFAEIIIDFVWITIIKLVALAKSLRRRRAQACYDGPPPTVAELVEAHTNFGFQPDT    596
beta-ENaC_Delphinapterus_leucas                    LCLIEFAEIIIDFVWITIIKLVALAKSLRRRRAQACYDGPPPTVAELVEAHTNFGFQPDT    595
beta-ENaC_Pontoporia_blainvillei                   LCLIEFAEIVIDFVWITIIKLVALAKSLWRRRAQARYDGPPPTVAELVEAHTNFGFQPDT    595
beta-ENaC_Inia_geoffrensis                         LCLIEFAEIIIDFVWITIIKLVALAKSLRQRRAQARYDGPPPTVAELVEAHTNFGFQPDT    595
beta-ENaC_Lipotes_vexillifer                       LCLIEFAEIIIDFVWITIIKLVALAKSLRRRRAEARYNGPPPTVAELVEAHTNFGFQPDR    595
beta-ENaC_Hyperoodon_ampullatus                    LCLIEFGEIIIDFLWITIIRLVAFAKRLRQRRSQACYDGPPPTVAELVEAHTNFGFQPDT    595
beta-ENaC_Mesoplodon_bidens                        LCLIEFGEIIIDFLWITIIRLVAFAKRLRQRRAQACYDGPPPTVAELVEAHTNFGFQPDT    595
beta-ENaC_Ziphius_cavirostris                      LCLIEFGEIIIDFLWITIIRLVAFAKRLRQRRAQACYDGPPPTVAELVEAHTNFGFQPDT    595
beta-ENaC_Platanista_gangetica                     LCLIEFGEIIIDFVWITIIRLVALTKSLRQRRAQARYDGPPPTVAELVEAHTNFGFQPDT    595
beta-ENaC_Platanista_minor                         LCLIEFGEIIIDFVWITIIRLVALTKSLRQRRAQARYDGPPPTVAELVEAHTNFGFQPDT    595
beta-ENaC_Kogia_breviceps                          LCLIEFGEIIIDFVWITIIRLVALAKSLRQRRARARYDGPPPTVAELVEAHTNFGFQPDR    595
beta-ENaC_Physeter_catodon                         LCLIEFGEIIIDFVWITIIRLVALAKSLRQRRTRARYDGPPPTVAELVEAHTNFGFQPDT    595
beta-ENaC_Balaenoptera_musculus                    LCLIEFGEIIIDFVWITIIRLVALAKSLRQRRAQACYDGPPPTVAELVEAHTNFGFQPDT    595
beta-ENaC_Balaenoptera_acutorostrata_scammoni      LCLIEFGEIIIDFVWITIIRLVALAKSLRQRRAQACYDGPPPTVAELVEAHTNFGFQPDT    595
beta-ENaC_Eubalaena_japonica                       LCLIEFGEIIIDFVWITIIRLVALAKSLRRRRAQACYDGPPPTVAELVEAHTNFGFQPDT    595
beta-ENaC_Hippopotamus_amphibius                   LCLIEFGEIVIDFVWITIIRLVALAKSLRRKRAQARYDGPPPTVAELVEAHTNFGFQPDV    598
beta-ENaC_Tragulus_javanicus                       LCIIEFGEIIIDFVWITIIKLVALAKGTRQKQAQARYDGPPPTVAELVEAHTNFAFQPDS    595
beta-ENaC_Tragulus_kanchil                         LCIIEFGEIIIDFVWITIIKLVALAKGTRQKQAQARYDGPPPTVAELVEAHTNFAFQPDS    595
beta-ENaC_Antilocapra_americana                    LCLIEFGEIIIDFVWITIIKLVALAKSVRQKRAQARYEGPPPTVAELVEAHTNFGFQPDS    595
beta-ENaC_Giraffa_camelopardalis                   LCLIEFGEIIIDFMWITIIKLVALAKSVRQKRAQARYEGPPPTVAELVEAHTNFGFQPDS    595
beta-ENaC_Giraffa_tippelskirchi                    LCLIEFGEIIIDFMWITIIKLVALAKSVRQKRAQARYEGPPPTVAELVEAHTNFGFQPDS    595
beta-ENaC_Capreolus_pygargus                       LCLIEFGEIIIDFVWITIIKLVALAKSVRQKRAQARYEGPPPTVAELVEAHTNFGFQPDS    596
beta-ENaC_Cervus_elaphus                           LCLIEFGEIIIDFVWITIIKLVALAKSVRQKRAQARYQGPPPTVAELVEAHTNFGFQPDS    595
beta-ENaC_Moschus_moschiferus                      LCLIEFGEIIIDFVWITIIKLVALAKSVRQKRALARYDGPPPTVAELVEAHTNFGFQPDP    595
```

```
beta-ENaC_Moschus_berezovskii                     LCLIEFGEIIIDFVWITIIKLVALAKSVRQKRALARYDGPPPTVAELVEAHTNFGFQPDP    595
beta-ENaC_Bos_grunniens                           LCLIEFGEIIIDFVWITIIKLVALAKSVRQKQAQARYEGPPPTVAELVEAHTNFGFQPDL    595
beta-ENaC_Bos_taurus                              LCLIEFGEIIIDFVWITIIKLVALAKSVRQKRAQARYEGPPPTVAELVEAHTNFGFQPDL    595
beta-ENaC_Bubalus_bubalis                         LCLIEFGEIIIDFVWITIIKLVALAKSVRQKRAQARYDGPPPTVAELVEAHTNFGFQPDL    595
beta-ENaC_Nanger_granti                           LCLIEFGEIIIDFVWITIIKLVALAKGVRQKRAQARYDGPPPTVAELVEAHTNFGFQPDS    595
beta-ENaC_Capra_hircus                            LCLIEFGEIIIDFVWITIIKLVALAQSMRQKRAQARYDGPPPTVAELVEAHTNFGFQPDS    595
beta-ENaC_Ovis_aries                              LCLIEFGEIIIDFVWITIIKLVALAKSVRQKRAQARYNGPPPTVAELVEAHTNFGFQPDS    595
beta-ENaC_Ovis_canadensis                         LCLIEFGEIIIDFVWITIIKLVALAKSVQQKRAQARYDGPPPTVAELVEAHTNFGFQPDS    595
beta-ENaC_Hippotragus_niger                       LCLIEFGEIIIDFVWITIIRLVALAKGVRQKRAQARYDGPPPTVAELVEAHTNFGFQPDL    596
beta-ENaC_Sus_scrofa                              LCLIEFAEIIIDFVWITIIKLVALAKSLRQRRAQARYVGPPPTVAELVEAHTNFSFQPDT    598
beta-ENaC_Vicugna_pacos                           LCLIEFGEIIIDFVWITVIKLVALAKSLRQRRAQARYAGPPPTVAELVEAHTNFSFQPDV    595
beta-ENaC_Equus_callabus                          LCLIEFGEIIIDFVWITVIKLVAFSKSLQRRRAQAHYASPPPTVAELVEAHTNFGFQPDV    595
                                                  **:***.**:***:***:*:*.*:::   :::: : * .***************.****

beta-ENaC_Homo_sapiens                            APRSPNTGPYPSEQALPIPGTPPPNYDSLRLQPLDVIESDSEGDAI    640
beta-ENaC_Globicephala_melas                      ATHGPDAGPHPHEQTPPIPGTPPPNYDSLRLQPLDVIESDNEGDAI    641
beta-ENaC_Lagenorhynchus_obliquidens              ATHGPDAGPHPHEQTPPIPGTPPPNYDSLRLQPLDVIESDNEGDAI    641
beta-ENaC_Tursiops_truncatus                      ATHGPDAGPHPHEQTPPIPGTPPPNYDSLRLQPLDVIESDNEGDAI    641
beta-ENaC_Orcinus_orca                            ATHGPDAGPHPHEQTPPIPGTPPPNYDSLRLQPLDVIESDNEGDAI    641
beta-ENaC_Phocoena_sinus                          APHGPDAGPHAHEQTPPIPGTPPPNYDSLRLQPLDVTESDNEGDAI    641
beta-ENaC_Neophocaena_asiaeorientalis             APHGPDAGPHPHEQTPPIPGTPPPNYDSLRLQPLDVTESDNEGDAI    641
beta-ENaC_Monodon_monocerons                      ATHGPDAGPHPHEQTPPIPGTPPPNYDSLRLQPLDVTESDNEGDAI    642
beta-ENaC_Delphinapterus_leucas                   ATHGPDAGPHPHEQTPPIPGTPPPNYDSLRLQPLDVTESDNEGDAI    641
beta-ENaC_Pontoporia_blainvillei                  ATHGPDAGPHPHEQTPPIPGTPPPNYDSLRLQPLDVIESDNEGDAI    641
beta-ENaC_Inia_geoffrensis                        ATHSPDAGPHPHKQTPPIPGTPPPNYDSLRLQPLDVIESDNEGDAI    641
beta-ENaC_Lipotes_vexillifer                      ATHGPDAGPHPHEQTPPIPGTPPPNYDSLRLRPLDVIESDNEGDAI    641
beta-ENaC_Hyperoodon_ampullatus                   ATHGPDAGPHPHEQTPPIPGTPPPNYDSLRLQPLDIIESDNEGDAI    641
beta-ENaC_Mesoplodon_bidens                       ATHGPDAGPHPHEQTPPIPGTPPPNYDSLRLQPLDIIESDNEGDAI    641
beta-ENaC_Ziphius_cavirostris                     ATHGPDAGPHPHEQTLPIPGTPPPNYDSLRLQPLDTIESDNEGDAI    641
beta-ENaC_Platanista_gangetica                    ATHGPDAGPHPREQTPSIPGTPPPNYDSLRLQPLDVIESDNEGDAI    641
beta-ENaC_Platanista_minor                        ATHGPDAGPHPREQTPSIPGTPPPNYDSLRLQPLDVIESDNEGDAI    641
beta-ENaC_Kogia_breviceps                         ATHGPDARPHPHEQAPPIPGTPPPNYDSLRLQPLDVIESDNEDDAI    641
beta-ENaC_Physeter_catodon                        ATHGPDARPHPHEQTPPIPGTPPPNYDSLRLQPLDVIESDNEGDAI    641
beta-ENaC_Balaenoptera_musculus                   ATHGPDAGPHPHEQTPPIPGTPPPNYDSLRLQPLDVIESDNEGDAI    641
beta-ENaC_Balaenoptera_acutorostrata_scammoni     ATHGPDAGPHPHEQTPPIPGTPPPNYDSLRLQPLDVIESDNEGDAI    641
beta-ENaC_Eubalaena_japonica                      ATHGPDAGPHPHEQTPPIPGTPPPNYDSLRLQPLDVIESDNEGDAI    641
beta-ENaC_Hippopotamus_amphibius                  ATRGPSAGPHSHEPTPPIPGTPPPNYDSLRLQPLDVIESDSEGDAV    644
beta-ENaC_Tragulus_javanicus                      ATPGPDTGPYTQEQTLPIPGTPPPNYDSLRLQPLDVIESDSEADAI    641
beta-ENaC_Tragulus_kanchil                        ATPGPDTGPYTQEQTLPIPGTPPPNYDSLRLQPLDVIESDSEADAI    641
beta-ENaC_Antilocapra_americana                   ATPGPDAGAYPHEQNPPIPGTPPPNYDSLRLQPLDIIESDSEGDAI    641
beta-ENaC_Giraffa_camelopardalis                  AMPGPNVGAYPHEQNPPIPGTPPPNYDSLRLQPLDVIESDSEGDAI    641
beta-ENaC_Giraffa_tippelskirchi                   AMPGPNVGAYPHEQNPPIPGTPPPNYDSLRLQPLDVIESDSEGDAI    641
beta-ENaC_Capreolus_pygargus                      ATTSPDAGTYTREQNPPIPGTPPPNYDSLRLQPLDVIESDSEGDAI    642
beta-ENaC_Cervus_elaphus                          ATPGPDAGAYPHEQNPPIPGTPPPNYDSLRLQPLDVIESDSEGDAI    641
beta-ENaC_Moschus_moschiferus                     ATPGPDAGAYPHEQNPPIPGTPPPNYDSLRLQPLDVIESDSDGDAI    641
beta-ENaC_Moschus_berezovskii                     ATPGPDAGAYPHEQNPPIPGTPPPNYDSLRLQPLDVIESDSDGDAI    641
beta-ENaC_Bos_grunniens                           ATPGPDVGAYPHEQNPPIPGTPPPNYDSLRLQPLDVIESDSEGDAI    641
beta-ENaC_Bos_taurus                              ATPGPDVEAYPHEQNPPIPGTPPPNYDSLRLQPLDVIESDSEGDAI    641
beta-ENaC_Bubalus_bubalis                         ATPGPDVGAYPHEQNPPIPGTPPPNYDSLRLQPLDVIESDSEGDAI    641
beta-ENaC_Nanger_granti                           ATPGPDAGAYPHEQNPPIPGTPPPNYESLRLQPLDVIESDSEGDAI    641
beta-ENaC_Capra_hircus                            AMPGPDAGAYSREQNPPIPGTPPPNYDSLRLQPLDVIESDSEGDAI    641
beta-ENaC_Ovis_aries                              AMPGPDAGAYRREQNPPIPGTPPPNYDSLRLQPLDVIESDSEGDAI    641
beta-ENaC_Ovis_canadensis                         AMPGPDAGAYRREQNPPIPGTPPPNYDSLRLQPLDVIESDSEGDAI    641
beta-ENaC_Hippotragus_niger                       ATPGPDTGAYPREQNPPIPGTPPPNYDSLRLQPLDVIESDSEGDAI    642
beta-ENaC_Sus_scrofa                              PAPSADPGPYVQEQAPPIPGTPPPNYDSLRLQPLDVIESDGEGDAV    644
beta-ENaC_Vicugna_pacos                           VTPGPSAGPYPDEQLPPIPGTPPPNYDSLRLQPLDVIESDTEGDAV    641
beta-ENaC_Equus_callabus                          ISHSPTTGTYPDEQPVPIPGTPPPNYDSLRLQPLDVVESDNEGDAI    641
                                                     .     :  :    *********:****:***  *** : **:
```

Supplementary Alignment 3

γ-ENaC (*SCNN1G*) amino acid sequence alignment created with Clustal Omega (v.1.2.4, accessed 24.03.2024). Key structural motifs are highlighted in the human ENaC subunit based on the Cryo-EM derived structure[1]. Transmembrane domains (TM1/TM2) are highlighted in yellow. An N-Terminal HG-motif affecting ENaC open probability is marked in magenta. Protease cleavage sites are highlighted in blue. Cysteines involved in tertiary structure formation are indicated in red. Residues putatively forming the selectivity filter within TM2 are shown in red font. The C-terminal PPPxY motif regulating membrane abundance is shown in **gray**.

```
                                                                                                      TM1
gamma-ENaC_Homo_sapiens                     MAPGEKIKAKIKKNLPVTGPQAPTIKELMRWYCLNTNTHGCRRIVVSRGRLRRLLWIGFT  60
gamma-ENaC_Globicephala_melas               MAAGEKIKAKIKKNLPVRGPQAPTIKELMRWYCLNTNTHGCRRIVVSRGRLRRLLWILFT  60
gamma-ENaC_Lagenorhynchus_obliquidens       MAAGEKIKAKIKKNLPVRGPQAPTIKELMRWYCLNTNTHGCRRIVVSRGRLRRLLWILFT  60
gamma-ENaC_Tursiops_truncatus               MAAGEKIKAKIKKNLPVRGPQAPTIKELMRWYCLNTNTHGCRRIVVSRGRLRRLLWILFT  60
gamma-ENaC_Orcinus_orca                     MAAGEKIKAKIKKNLPVRGPQAPTIKELMRWYCLNTNTHGCRRIVVSRGRLRRLLWILFT  60
gamma-ENaC_Phocoena_sinus                   MAAGEKIKAKIKKNLPVRGPQAPTIKELMRWYCLNTNTHGCRRIVVSRGRLRRLLWILLT  60
gamma-ENaC_Neophocaena_asiaeorientalis      MAAGEKIKAKIKKNLPVRGPQAPTIKELMRWYCLNTNTHGCRRIVVSRGRLRRLLWILLT  60
gamma-ENaC_Monodon_monocerons               MAAGEKIKAKIKKNLPVRGPQAPTIKELMRWYCLNTNTHGCRRIVVSRGRLRRLLWILFT  60
gamma-ENaC_Delphinapterus_leucas            MAAGEKIKAKIKKNLPVRGPQAPTIKELMRWYCLNTNTHGCRRIVVSRGRLRRLLWILFT  60
gamma-ENaC_Pontoporia_blainvillei           MAAGEKIKAKIKKNLPVRGPQAPTIKELMRWYCLNTNTHGCRRIVVSRGRLRRLLWILFT  60
gamma-ENaC_Inia_geoffrensis                 MAAGEKIKAKIKKNLPVRGPQAPTIKELMRWYCLNTNTHGCRRIVVSRGRLRRLLWILFT  60
gamma-ENaC_Lipotes_vexillifer               MAAGEKIKAKIKKNLPVRGPQAPTIKELMRWYCLNTNTHGCRRIVVSRGRLRRLLWILFT  60
gamma-ENaC_Hyperoodon_ampullatus            MAAGEKIKAKIKENLPVRGPQAPTIKELMRWYCLNTNTHGCRRIVVSRGRLRRLLWILFT  60
gamma-ENaC_Mesoplodon_bidens                MAAGEKIKAKIKENLPVRGPQAPTIKELMRWYCLNTNTHGCRRIVVSRGRLRRLLWILFT  60
gamma-ENaC_Ziphius_cavirostris              MAAGEKIKAKIKENLPVRGPQAPTIRELMRWYCLNTNTHGCRRIVVSRGRLRRLLWILFT  60
gamma-ENaC_Platanista_gangetica             MAAGEKIKAKIKKNLPVRGPQAPTIKELMRWYCLNTNTHGCRRIVVSRGRLRRLLWILFT  60
gamma-ENaC_Platanista_minor                 MAAGEKIKAKIKKNLPVRGPQAPTIKELMRWYCLNTNTHGCRRIVVSRGRLRRLLWILFT  60
gamma-ENaC_Kogia_breviceps                  MAAGEKIKAKIKKNLPVRGPQAPTIKELMRWYCLNTNTHGCRRIVVSRGRLRRLLWILFT  60
gamma-ENaC_Physeter_catodon                 MAAGEKIKAKIKKNLPVRGPQAPTIKELMRWYCLNTNTHGCRRIVVSRGRLRRLLWILFT  60
gamma-ENaC_Balaenoptera_musculus            MAAGEKIKAKIKKNLPVRGPQAPTIKELMQWYCLNTNTHGCRRIVVSRGRLRRLLWILFT  60
gamma-ENaC_Eubalaena_japonica               MAAGEKIKAKIKKNLPVRGPQAPTIKELMQWYCLNTNTHGCRRIVVSRGRLRRLLWILFT  60
gamma-ENaC_Hippopotamus_amphibius           MAPGEKIKAKIKKNLPVTGPQAPTIKELMRWYCLNTNTHGCRRIVVSRGRLRRLLWILFT  60
gamma-ENaC_Tragulus_javanicus               MAPGEKIKAKIKKNLPVTGPQAPTIKELMRWYCLNTNTHGCRRIVVSRGRLRRLLWILFT  60
gamma-ENaC_Tragulus_kanchil                 MAPGEKIKAKIKKNLPVTGPQAPTIKELMRWYCLNTNTHGCRRIVVSRGRLRRLLWILFT  60
gamma-ENaC_Antilocapra_americana            MAPGEKIKAKIKKNLPVTGPQAPNIKELMQWYCLNTNTHGCRRIVVSRGRLRRLLWILFT  60
gamma-ENaC_Giraffa_camelopardalis           MAPGEKIKAKIKKNLPVTGPQAPNIKELMQWYCLNTNTHGCRRIVVSRGRLRRLLWILFT  60
gamma-ENaC_Giraffa_tippelskirchi            MAPGEKIKAKIKKNLPVTGPQAPNIKELMQWYCLNTNTHGCRRIVVSRGRLRRLLWILFT  60
gamma-ENaC_Capreolus_pygargus               MAPGEKIKAKIKKNLPVTGPQAPNIKELMQWYCLNTNTHGCRRIVVSRGRLRRLLWILFT  60
gamma-ENaC_Cervus_elaphus                   MAPGEKIKAKIKKNLPVTGPQAPNIKELMQWYCLNTNTHGCRRIVVSRGRLRRLLWILFT  60
gamma-ENaC_Moschus_moschiferus              MAPGEKIKAKIKKNLPVTGPQAPNIKELMQWYCLNTNTHGCRRIVVSRGRLRRLLWILFT  60
gamma-ENaC_Moschus_berezovskii              MAPGEKIKAKIKKNLPVTGPQAPNIKELMQWYCLNTNTHGCRRIVVSRGRLRRLLWILFT  60
gamma-ENaC_Bos_grunniens                    MAPGEKIKAKIKKNLPVTGPQAPNIKELMQWYCLNTNTHGCRRIVVSRGRLRRLLWILFT  60
gamma-ENaC_Bos_taurus                       MAPGEKIKAKIKKNLPVTGPQAPNIKELMQWYCLNTNTHGCRRIVVSRGRLRRLLWILFT  60
gamma-ENaC_Bubalus_bubalis                  MAPGEKIKAKIKKNLPVTGPQAPNIKELMQWYCLNTNTHGCRRIVVSRGRLRRLLWILFT  60
gamma-ENaC_Nanger_granti                    MAPGEKIKAKIKKNLPVTGPQAPNIKELMQWYCLNTNTHGCRRIVVSRGRLRRLLWILFT  60
gamma-ENaC_Kobus_leche                      MAPGEKIKAKIKKNLPVTGPQAPSIKELMHWYCLNTNTHGCRRIVVSRGRLRRLLWILFT  60
gamma-ENaC_Capra_hircus                     MAPGEKIKAKIKKNLPVTGPQAPNIKELMQWYCLNTNTHGCRRIVVSRGRLRRLLWILFT  60
gamma-ENaC_Ovis_aries                       MAPGEKIKAKIKKNLPVTGPQAPNIKELMQWYCLNTNTHGCRRIVVSRGRLRRLLWILFT  60
gamma-ENaC_Ovis_canadensis                  MAPGEKIKAKIKKNLPVTGPQAPNIKELMQWYCLNTNTHGCRRIVVSRGRLRRLLWILFT  60
gamma-ENaC_Oreamnos_americanus              MAPGEKIKAKIKKNLPVTGPQAPNIKELMQWYCLNTNTHGCRRIVVSRGRLRRLLWILFT  60
gamma-ENaC_Hippotragus_niger                MAPGEKIKAKIKKNLPVTGPQAPNIKELMQWYCLNTNTHGCRRIVVSRGRLRRLLWILFT  60
gamma-ENaC_Damaliscus_lunatus               MAPGEKIKAKIKKNLPVTGPQAPNIKELMQWYCLNTNTHGCRRIVVSRGRLRRLLWILFT  60
gamma-ENaC_Sus_scrofa                       MAPGEKIKAKIKKNLPVTGPQAPTIKELMQWYCLNTNTHGCRRIVVSRGRLRRLLWILFT  60
gamma-ENaC_Vicugna_pacos                    MAPGEKIKAKIKKNLPVKGPQAPTIKELMRWYCLNTNTHGCRRIVVSRGRLRRLLWILFT  60
gamma-ENaC_Equus_callabus                   MAPGEKIKAKIKKNLPVTGPQAPTIKELMRWYCLNTNTHGCRRIVVSRGRLRRLLWILFT  60
                                            ** *********:**** *****.*:***:*************************** :*


gamma-ENaC_Homo_sapiens                     LTAVALILWQCALLVFSFYTVSVSIKVHFRKLDFPAVTICNINPYKYSTVRHLLADLEQE  120
gamma-ENaC_Globicephala_melas               LTAVALIFWQCALLISSFYTVSVSIKVHFQKLDFPAVTICNINPYRYSAVRDLLAELEQE  120
gamma-ENaC_Lagenorhynchus_obliquidens       LTAVALIFWQCALLISSFYTVSVSIKVHFQKLDFPAVTICNINPYRYSAVRDLLAELEQE  120
gamma-ENaC_Tursiops_truncatus               LTAVALIFWQCALLISSFYTVSVSIKVHFQKLDFPAVTICNINPYRYSAVRDLLAELEQE  120
gamma-ENaC_Orcinus_orca                     LTAVALIFWQCALLISSFYTVSVSIKVHFQKLDFPAVTICNINPYRYSAVRDLLAELEQE  120
gamma-ENaC_Phocoena_sinus                   LTAVALIFWQCALLISSFYTVSVSIKVHFQKLDFPAVTICNINPYRYSAVRDLLAELEQE  120
gamma-ENaC_Neophocaena_asiaeorientalis      LTAVALIFWQCALLISSFYTVSVSIKVHFQKLDFPAVTICNINPYRYSAVRDLLAELEQE  120
gamma-ENaC_Monodon_monocerons               LTAVALIFWQCALLISSFYTVSVSIKVHFQKLDFPAVTICNINPYRYSAVRDLLAELEQE  120
gamma-ENaC_Delphinapterus_leucas            LTAVALIFWQCALLISSFYTVSVSIKVHFQKLDFPAVTICNINPYRYSAVRDLLAELEQE  120
gamma-ENaC_Pontoporia_blainvillei           LTAVALIFWQCALLISSFYTVSVSIKVHFQKLDFPAVTICNINPYRYSAVRDLLAELEQE  120
gamma-ENaC_Inia_geoffrensis                 LTAVALIFWQCALLISSFYTVSVSIKVHFQKLDFPAVTICNINPYRYSAVRDLLAELEQE  120
gamma-ENaC_Lipotes_vexillifer               LTAVALIFWQCALLISTFYTVSVSIKVHFQKLDFPAVTICNINPYRYSAVRHLLAELEQE  120
gamma-ENaC_Hyperoodon_ampullatus            LTAVALIFWQCALLISSFYTVSVSIKVHFQKLDFPAVTICNINPYRYSAVRDLLADLEQE  120
gamma-ENaC_Mesoplodon_bidens                LTAVALIFWQCALLISSFYTVSVSIKVHFQKLDFPAVTICNINPYRYSAVRDLLADLEQE  120
gamma-ENaC_Ziphius_cavirostris              LTAVALIFWQCALLISSFYTVSVSIKVHFQKLDFPAVTICNINPYRYSAVRDLLADLEQE  120
gamma-ENaC_Platanista_gangetica             LTAVALIFWQCALLISSFYTVSVSIKVHFQKLDFPAVTICNINPYRYSAVRDLLADLEQE  120
gamma-ENaC_Platanista_minor                 LTAVALIFWQCALLISSFYTVSVSIKVHFQKLDFPAVTICNINPYRYSAVRDLLADLEQE  120
gamma-ENaC_Kogia_breviceps                  LTAVALIFWQCALLISSFYTVSVSIKVHFQKLDFPAVTICNINPYRYSAVRDLLADLEQE  120
```

```
gamma-ENaC_Physeter_catodon                 LTAVALIFWQCALLISSFYTVSVSIKVHFQKLDFPAVTICNINPYRYSAVRDLLADLEQE  120
gamma-ENaC_Balaenoptera_musculus            LTAVALIFWQCALLISSFYTVSVSIKVHFQKLDFPAVTICNINPYRYSAVRDLLADLEKE  120
gamma-ENaC_Eubalaena_japonica               LTAVALIFWQCALLISSFYTVSVSIKVHFQKLDFPAVTICNINPYRYSAVQDLLADLEKE  120
gamma-ENaC_Hippopotamus_amphibius           LTAVALIFWQCALLISSFYTVSVSIKVHFQKLDFPAVTICNINPYKYSAVRDLLADLERE  120
gamma-ENaC_Tragulus_javanicus               LTAVALIFWQCALLISSFYTVSVSIKVHFQKLDFPAVTICNMNPFKYSAVQHLLADLEQE  120
gamma-ENaC_Tragulus_kanchil                 LTAVALIFWQCALLISSFYTVSVSIKVHFQKLDFPAVTICNMNPFKYSAVQHLLADLEQE  120
gamma-ENaC_Antilocapra_americana            LTAVALIFWQCALLISSFYTVSVSIKVHFQKLDFPAVTICNINPYKYSAVRPLLADLEQE  120
gamma-ENaC_Giraffa_camelopardalis           LTAVALILWQCALLISSFYTVSVSIKVHFQKLDFPAVTICNINPYKYSAVRHLLADLEQE  120
gamma-ENaC_Giraffa_tippelskirchi            LTAVALILWQCALLISSFYTVSVSIKVHFQKLDFPAVTICNINPYKYSAVRHLLADLEQE  120
gamma-ENaC_Capreolus_pygargus               LTAVALIFWQCALLISSFYTVSVSIKVHFQKLDFPAVTICNINPYKYSAVRHLLADLEQE  120
gamma-ENaC_Cervus_elaphus                   LTAVALIFWQCALLISSFYTVSVSIKVHFQKLDFPAVTICNINPYKYSAVRHLLADLEQE  120
gamma-ENaC_Moschus_moschiferus              LTAVALIFWQCALLISSFYTVSVSIKVHFQKLDFPAVTICNINPYRYSAVRHLLADLEQE  120
gamma-ENaC_Moschus_berezovskii              LTAVALIFWQCALLISSFYTVSVSIKVHFQKLDFPAVTICNINPYRYSAVRHLLADLEQE  120
gamma-ENaC_Bos_grunniens                    LTAVALIFWQCALLISSFYTVSVSIKVHFQKLDFPAVTICNINPYKYSAVRHLLADLEQE  120
gamma-ENaC_Bos_taurus                       LTAVALIFWQCALLISSFYTVSVSIKVHFQKLDFPAVTICNINPYKYSAVRHLLADLEQE  120
gamma-ENaC_Bubalus_bubalis                  LTAVGLIFWQCALLISSFYTVSVSIKVHFQKLDFPAVTICNINPYKYSAVRHLLADLEQE  120
gamma-ENaC_Nanger_granti                    LTAVALIFWQCSLLISSFYTVSVSIKVHFQKLDFPAVTICNINPYKYSAVRHLLADLERE  120
gamma-ENaC_Kobus_leche                      LTAVALIFWQCALLISSFYTVSVSIKVHFQKLDFPAVTICNINPYKYSAVRHLLGDLEQE  120
gamma-ENaC_Capra_hircus                     LTAVALIFWQCALLISSFYTVSVSIKVHFQKLDFPAVTICNINPYKYSAVRHLLADLEQE  120
gamma-ENaC_Ovis_aries                       LTAVALIFWQCALLISSFYTVSVSIKVHFQKLDFPAVTICNINPYKYSAVRHLLADLEQE  120
gamma-ENaC_Ovis_canadensis                  LTAVALIFWQCALLISSFYTVSVSIKVHFQKLDFPAVTICNINPYKYSAVRHLLADLEQE  120
gamma-ENaC_Oreamnos_americanus              LTAVALIFWQCALLISSFYTVSVSIKVHFQKLDFPAVTICNINPYKYSAVRHLLADLEQE  120
gamma-ENaC_Hippotragus_niger                LTAVALIFWQCALLISSFYTVSVSIKVHFQKLDFPAVTICNINPYKYSAVRHLLADLEQE  120
gamma-ENaC_Damaliscus_lunatus               LTAVALIFWQCALLISSFYTVSVSIKVHFQKLDFPAVTICNINPYKYSAVRHLLADLEQE  120
gamma-ENaC_Sus_scrofa                       LTAVALIFWQCALLILSFYTVSVSIKVHFQKLDFPAVTICNINPYKYSAVRDLLAELEQE  120
gamma-ENaC_Vicugna_pacos                    LTAVALIFWQCALLILSFYTVSVSIKVHFQKLDFPAVTICNINPYKYSAVRDLLADLEQE  120
gamma-ENaC_Equus_callabus                   LTAVALIFWQCALLVISFYTVSVSIKIHFQKLDFPAVTICNINPYKYSAVRDLLADLEQE  120
                                            ****.**:***:**: :*********:**:***********:**::**:*: **.:**:*

gamma-ENaC_Homo_sapiens                     TREALKSLYGFPE--SRKRREAESWNSVSEGKQPRFSHRIPLLIFDQDEKGKARDFFTGR  178
gamma-ENaC_Globicephala_melas               TRGSLKTLFGFSEITSRKRREAESWSSARKGTGSKFLNLIPLLAFEKGETSKARDFRTGR  180
gamma-ENaC_Lagenorhynchus_obliquidens       TRGSLKTLFGFSEITSRKRREAESWSSARKGTGSKFLNLIPLLAFEKGETSKARDFRTGR  180
gamma-ENaC_Tursiops_truncatus               TRGSLKTLFGFSEITSRKRREAESWSSARKGTGSKFLNLIPLLAFEKGETSKARDFRTGR  180
gamma-ENaC_Orcinus_orca                     TRGSLKTLFGFSEITSRKRREAESWSSARKGTGSKFLNLIPLLAFEKGETSKARDFRTGR  180
gamma-ENaC_Phocoena_sinus                   TRGSLKTLFGFSEITSRKRREAESWSSARKGTGSKFLNLIPLLAFDKGETSKARDFRTGR  180
gamma-ENaC_Neophocaena_asiaeorientalis      TRGSLKTLFGFSEITSRKRREAESWSSARKGTGSKFLNLIPLLAFDKGETSKARDFRTGR  180
gamma-ENaC_Monodon_monocerons               TRGSLKTLFGFSEITSRKRREAESWSSARKGTGSKFLNLIPLLAFDKGETSKARDFRTGR  180
gamma-ENaC_Delphinapterus_leucas            TRGSLKTLFGFSEITSRKRREAESWSSARKGTGSKFLNLIPLLAFDKGETSKARDFRTGR  180
gamma-ENaC_Pontoporia_blainvillei           TRGSLKTLFGFSEITSRKRREAESWSSAREGTGSKFLNLIPLLAFDKGETSKARDFRTGR  180
gamma-ENaC_Inia_geoffrensis                 TRGNLKTLFGFSEITSRKRREAESWSLAREGTGSKFLNLIPLLAFDKGETSKARDFRTGR  180
gamma-ENaC_Lipotes_vexillifer               TRGSLKTLFGFSEITSRKRREAESWSSAKEGTGSKFLNLIPLLAFDKGETSKARDFRTGR  180
gamma-ENaC_Hyperoodon_ampullatus            TRGTLKTLYGFSEIISRKRREAESWSSAREGTGSKFLNLIPLLAFDKGETSKARDFRTGR  180
gamma-ENaC_Mesoplodon_bidens                TRGTLKTLYGFSEIISRKRREAESWSSAREGTGSKFLNLIPLLAFDKGETSKARDFRTGR  180
gamma-ENaC_Ziphius_cavirostris              TRGTLKTLYGFSEIISRKRREAESWSSAREGTGSKFLNLIPLLAFDKGETSKARDFRTGR  180
gamma-ENaC_Platanista_gangetica             TRGTLKTLYGFPEITSRKRREAESWSSAREGTGSKFLNLIPLLAFDKGETSKARDFRTGR  180
gamma-ENaC_Platanista_minor                 TRGTLKTLYGFPEITSRKRREAESWSSAREGTGSKFLNLIPLLAFDKGETSKARDFRTGR  180
gamma-ENaC_Kogia_breviceps                  TRGNLKTLYGFSEITSRKRRDAESWSSAREDTGSKFLNLVPLLAFDKGETSKARDFRTGR  180
gamma-ENaC_Physeter_catodon                 TRGTLKTLYGFSEITSRKRREAESWSSAREGTGSTFLNLVPLLAFEKGETSKATDFRTGR  180
gamma-ENaC_Balaenoptera_musculus            TRGTLKTLYGFSEITSRKRREAESWSSAREGTGSKFLNLIPLLAFDKGETSKARDFRTGR  180
gamma-ENaC_Eubalaena_japonica               TRGTLKTLYGFSEITSRKRREAESWSSAREGTGSKFLNLIPLLAFDKGETSKARDFRTGR  180
gamma-ENaC_Hippopotamus_amphibius           TRAALKTLYGFSEITHRKRREAESRSSARE---GKFLNLVPLLTFDTSETGKARDFRTGR  177
gamma-ENaC_Tragulus_javanicus               TRAALKTLYGFEEITSRKCREAESWSSARKDLQPKFLNLAPLMAFEKGETSKARDFFTGQ  180
gamma-ENaC_Tragulus_kanchil                 TRAALKTLYGFEEITSRKRREAESWSSARKDLQPKFLNLAPLMAFEKGETSKARDFFTGQ  180
gamma-ENaC_Antilocapra_americana            TRAALTTLYGFSEITSRKRREAQSWSSAREGTDPRFLNLAPLMAFEKGDKGKARDFFTGR  180
gamma-ENaC_Giraffa_camelopardalis           TRAALKTLYGFSEITSRKRREAQSWSSAREGNDPKFLNLAPLMAFEKGDTGKARDFFTGR  180
gamma-ENaC_Giraffa_tippelskirchi            TRAALKTLYGFSEITSRKRREAQSWSSAREGNDPKFLNLAPLMAFEKGDTGKARDFFTGR  180
gamma-ENaC_Capreolus_pygargus               TRAALKTLYGFSEITSRKRREAQSWSSVRKGTDPKFLNLAPLMAFEKGDTDKARDFFTGR  180
gamma-ENaC_Cervus_elaphus                   TRAALKTLYGFSEIKSRKRREAQPWSSVRKGADPKFLNLAPLMAFEKGDTGKARDFFTGR  180
gamma-ENaC_Moschus_moschiferus              TRAALKTLYGFSEITSRKRREAQSWSSARKGTDPKFLNLAPLMAFEKGDTGKARDFFTGR  180
gamma-ENaC_Moschus_berezovskii              TRAALKTLYGFSEITSRKRREAQSWSSARKGTDPKFLNLAPLMAFEKGDTGKARDFFTGR  180
gamma-ENaC_Bos_grunniens                    TRAALKTLYGFSEITSRKRREAQSWSSVRKGTDPKFLNLAPLMAFEKGDTGKARDFFTGR  180
gamma-ENaC_Bos_taurus                       TRAALKTLYGFSEITSRKRREAQSWSSVRKGTDPKFLNLAPLMAFEKGDTGKARDFFTGR  180
gamma-ENaC_Bubalus_bubalis                  TRAALKTLYGFSEITSRKRREAQSWSSVRRGTDPKFLNLAPLMAFEKGDTGKARDFFTGR  180
gamma-ENaC_Nanger_granti                    TRAALKTLYGFSEITSRKRREAQSQSSVRKDTDPKFLNLAPLMAFEEGDTGKARDFFTGR  180
gamma-ENaC_Kobus_leche                      TRAALKNLYGFSEITSRKRREAQSQSSVRKGTDPKFLNLAPLMAFEKGDTGKARDFITGR  180
gamma-ENaC_Capra_hircus                     TRAALKHLYGFSEITSRKRREAQSRSSVRKGTDPKFLNLAPLMAFEKGDMGKARDFFTGR  180
gamma-ENaC_Ovis_aries                       TRAALKHLYGFSEITSRKRREAQSRSSVRKGTDPRFLNLAPLMALEKGDMGKARDFFTGR  180
gamma-ENaC_Ovis_canadensis                  TRAALKHLYGFSEITSRKRREAQSRSSVRKGTDPRFLNLAPLMAFEKGDMGKARDFFTGR  180
gamma-ENaC_Oreamnos_americanus              TRAALKHLYGFSEITSRKRREAQSRSSVRKGTDPKFLNLAPLMAFEKGDMGKARDFFTGR  180
gamma-ENaC_Hippotragus_niger                TRAALKTLYGFSEITSRKRREAQSRSSVRKGTDPKFLNLAPLMAFEKGDTGKARDFFTGR  180
gamma-ENaC_Damaliscus_lunatus               TREALKTLYGFSEITSRKRREAQSRSSVRKSTDPKFLNLAPLMAFEQGDTGKARDFFTGR  180
gamma-ENaC_Sus_scrofa                       TRGALKTLYGFSEITSRKRREAESQNSAWEGTGSKFLNLIPLLVFNQGETGKARDFLTGR  180
gamma-ENaC_Vicugna_pacos                    TRAALKTLYGFPEITSRKRRESESWRSASEGSGSKFLNLVPLLAFNQSERNKARDFLTGR  180
gamma-ENaC_Equus_callabus                   TRGALKTLYGFSEIKSRKRRDAESWSSVWEGTRPKFLNLVPLLAFKEGEMGKARDFLTGR  180
                                            **  *. *:** *   ** *:::    . .     * :  **: :. .: .** ** **:

gamma-ENaC_Homo_sapiens                     KRKVGGSIIHKASNVMHIE-SKQVVGFQLCSNDTSDCATYTFSSGINAIQEWYKLHYMNI  237
gamma-ENaC_Globicephala_melas               KRKVSGRIVHTASDVVHVYESKGLVGFQLCSNDTSSCAVYTFTSGVNAIREWYKLHYMNI  240
gamma-ENaC_Lagenorhynchus_obliquidens       KRKVSGRIVHTASDVVHVYESKGLVGFQLCSNDTSSCAVYTFTSGVNAIREWYKLHYMNI  240
gamma-ENaC_Tursiops_truncatus               KRKVSGRIVHTASDVVHVYESKGLVGFQLCSNDTSSCAVYTFTSGVNAIREWYKLHYMNI  240
gamma-ENaC_Orcinus_orca                     KRKVSGRIVHTASDVVHVYESKGLVGFQLCSNDTSSCAVYTFTSGVNAIREWYKLHYMNI  240
gamma-ENaC_Phocoena_sinus                   KRKVSGRIIHTASDVVHVYESKGSVGFQLCSNDTSSCAVYTFTSGVNAIREWYKLHYMNI  240
gamma-ENaC_Neophocaena_asiaeorientalis      KRKVSGRIIHTASDVVHVYESKGSVGFQLCSNDTSSCAVYTFTSGVNAIREWYKLHYMNI  240
gamma-ENaC_Monodon_monocerons               KRKVSGRIIHTASDVVHVYESKGLVGFQLCSNDTSSCAVYTFTSGVNAIREWYKLHYMNI  240
```

```
gamma-ENaC_Delphinapterus_leucas            KRKVSGRIIHTASDVVHVYESKGLVGFQLCSNDTSSCAVYTFTSGVNAIREWYKLHYMNI  240
gamma-ENaC_Pontoporia_blainvillei           KRQVSGRIIHKASDVVHVYESKDVVGFQLCLNDTSNCTVYTFSSGVNAIREWYKLHYMNI  240
gamma-ENaC_Inia_geoffrensis                 KRKVSGRIIHKASDVMHVYESKDVVGFQLCSNDTSSCAVYTFSSGVNAIREWYKLHYMNI  240
gamma-ENaC_Lipotes_vexillifer               KRKVNGRIIHKASDVVHVYESKDMVGFQLCSNDTSSCAVYTFSSGINAIREWYKLHYMNI  240
gamma-ENaC_Hyperoodon_ampullatus            KRKVRGRIIHKASDVMHVHDSKEVVGFQLCPNDTSSCTVYTFNSGVNAIREWYKLHYMNI  240
gamma-ENaC_Mesoplodon_bidens                KRKVRGRIIHKASDVMHVHDSKEVVGFQLCPNDTSSCTVYTFNSGVNAIREWYKLHYMNI  240
gamma-ENaC_Ziphius_cavirostris              KRKVRGKIIHKASDVMHVHDSKEVVGFQLCPNDTSRCTVYTFNSGVNAIREWYKLHYMNI  240
gamma-ENaC_Platanista_gangetica             KRKVSGRIIHKASDVMHVHESKEVVGFQLCSNDTSSCAVYTFSSGVNAIREWYKLHYMNI  240
gamma-ENaC_Platanista_minor                 KRKVSGRIIHKASDVMHVHESKEVVGFQLCSNDTSSCAVYTFSSGVNAIREWYKLHYMNI  240
gamma-ENaC_Kogia_breviceps                  KRKVSGRIIHEASDVMHVHES-EVVGFQLCSNDTSSCAVYTFSSGVNAIQEWYKLHYMNI  239
gamma-ENaC_Physeter_catodon                 KRKVSGRIIHKSSDVMHVHESKEVVGFQLCSNDTSSCAVYTFSSGVNAIQEWYKLHYMNI  240
gamma-ENaC_Balaenoptera_musculus            KRKVSGRIIHKASDVMHVHESKEVVGFQLCSNDTSSCAVYTFSSGVNAIREWYKLHYMNI  240
gamma-ENaC_Eubalaena_japonica               KRKVSGRIIHEASDVMHVHESKEVVGFQLCSNDTSSCAVYTFSSGVNAIREWYKLHYMNI  240
gamma-ENaC_Hippopotamus_amphibius           KRKVNGKIIHKASDVMHVHDSKEVVGFQLCSNDTSSCAVYTFSSGINAIQEWYKLHYMNI  237
gamma-ENaC_Tragulus_javanicus               KRRVRGRIVHKASDVMHIHNSKEVVGFQLCSNDTSDCAVYTFSSGVNAIQEWYKLHYMNI  240
gamma-ENaC_Tragulus_kanchil                 KRRVRGRIVHKASDVMHIHNSKEVVGFQLCSNDTSDCAVYTFSSGVNAIQEWYKLHYMNI  240
gamma-ENaC_Antilocapra_americana            KRKVNARIIHKASDVMHIHSSKEVVGFQLCSNDTSDCAVYTFSSGINAIQEWYKLHYMNI  240
gamma-ENaC_Giraffa_camelopardalis           KRKVNARIIHKASDVMHIHNSKEVVGFQLCSNDTSDCAVYTFSSGVNAIQEWYKLHYMNI  240
gamma-ENaC_Giraffa_tippelskirchi            KRKVNARIIHKASDVMHIHNSKEVVGFQLCSNDTSDCAVYTFSSGVNAIQEWYKLHYMNI  240
gamma-ENaC_Capreolus_pygargus               KRKVNARIIHKASDVMHIHNSKEVVGFQLCSNDTSDCAVYTFSSGVNAIQEWYKLHYMNI  240
gamma-ENaC_Cervus_elaphus                   KRRVNAKIIHKASDVMHIHNSKEVVGFQLCSNDTSDCAVYTFSSGVNAIQEWYKLHYMNI  240
gamma-ENaC_Moschus_moschiferus              KRRVNARIIHKASDVMHIHNSKEVVGFQLCSNDTSDCAVYTFSSGVNAIQEWYKLHYMNI  240
gamma-ENaC_Moschus_berezovskii              KRRVNARIIHKASDVMHIHNSKEVVGFQLCSNDTSDCAVYTFSSGVNAIQEWYKLHYMNI  240
gamma-ENaC_Bos_grunniens                    KRKVNARIIHKASDVMHIHNSKEVVGFQLCSNDTSDCAVYTFSSGVNAIQEWYKLHYMNI  240
gamma-ENaC_Bos_taurus                       KRKVNARIIHKASDVMHIHNSKEVVGFQLCSNDTSDCAVYTFSSGVNAIQEWYKLHYMNI  240
gamma-ENaC_Bubalus_bubalis                  KRKVNARIIHKASDVMHIHNSKEVVGFQLCSNDTSDCAVYTFSSGVNAIQEWYKLHYMNI  240
gamma-ENaC_Nanger_granti                    KRKVNAKIIHKASDVMHIHNSKEVVGFQLCSNDTSDCAVYTFSSGVNAIQEWYKLHYMNI  240
gamma-ENaC_Kobus_leche                      KRKVNARIIHKASDVMHIHNSKEVVGFQLCSNDTSDCAVYTFSSGVNAIQEWYKLHYMNI  240
gamma-ENaC_Capra_hircus                     KRKVNARIIHKASDVMHIHNSKEVVGFQLCSNDTSDCAVYTFSSGVNAIQEWYKLHYMNI  240
gamma-ENaC_Ovis_aries                       KRKVNARIIHKASDVMHIHNSKEVVGFQLCSNDTSDCAVYTFSSGVNAIQEWYKLHYMNI  240
gamma-ENaC_Ovis_canadensis                  KRKVNARIIHKASDVMHIHNSKEVVGFQLCSNDTSDCAVYTFSSGVNAIQEWYKLHYMNI  240
gamma-ENaC_Oreamnos_americanus              KRKVNARIIHKASDVMHIHNSKEVVGFQLCSNDTSDCAVYTFSSGVNAIQEWYKLHYMNI  240
gamma-ENaC_Hippotragus_niger                KRKVNARIIHKASDVMHIHNSKEVVGFQLCSNDTSDCAVYTFSSGVNAIQEWYKLHYMNI  240
gamma-ENaC_Damaliscus_lunatus               KRKVNARIIHKASDVMHIHNSKEVVGFQLCSNDTSDCAVYTFSSGVNAIQEWYKLHYMNI  240
gamma-ENaC_Sus_scrofa                       KRKVSGSIIHKASDVMHVHDSKEVVGFQLCANDTSNCAVYTFSSGVNAIQEWYKLHYMNI  240
gamma-ENaC_Vicugna_pacos                    KRKVSGSIIHKASDVMHGHESKEVVGFQLCSNDTSDCAVYTFSSGVNAIREWYKLHYMNI  240
gamma-ENaC_Equus_callabus                   KRKVSGSIIRKESDVMNVHESKEVVGFQLCSNDTSNCAVYTFSSGVNAIQEWYKLHYMNI  240
                                            **:* . *::  *:*::   *   ****** **** *:.***.**:***:**********

gamma-ENaC_Homo_sapiens                     MAQVPLEKKINMSYSAEELLVTCFFDGVSCDARNFTLFHHPMHGNCYTFNNRENETILST  297
gamma-ENaC_Globicephala_melas               MAQVSLEKKINMSYSAEELLVTCFFDGESCDARNFTRFHHPMYGNCYTFNNRQNETILST  300
gamma-ENaC_Lagenorhynchus_obliquidens       MAQVSLEKKINMSYSAEELLVTCFFDGESCDARNFTRFHHPMYGNCYTFNNRQNETILST  300
gamma-ENaC_Tursiops_truncatus               MAQVSLEKKINMSYSAEELLVTCFFDGESCDARNFTRFHHPMYGNCYTFNNRQNETILST  300
gamma-ENaC_Orcinus_orca                     MAQVSLEKKINMSYSAEELLVTCFFDGESCDARNFTRFHHPMYGNCYTFNNRQNETILST  300
gamma-ENaC_Phocoena_sinus                   MAQVSLEKKINMSYSAEELLVTCFFDGESCDARNFTRFHHPMYGNCYTFNNRQNETILST  300
gamma-ENaC_Neophocaena_asiaeorientalis      MAQVSLEKKINMSYSAEELLVTCFFDGESCDARNFTRFHHPMYGNCYTFNNRQNETILST  300
gamma-ENaC_Monodon_monocerons               MAQVSLEKKINMSYSAEELLVTCFFDGESCDARNFTRFHHPMYGNCYTFNNRQNETILST  300
gamma-ENaC_Delphinapterus_leucas            MAQVSLEKKINMSYSAEELLVTCFFDGESCDARNFTRFHHPMYGNCYTFNNRQNETILST  300
gamma-ENaC_Pontoporia_blainvillei           MAQVSLEKKINMSYSAEELLVTCFFDGESCDARNFTRFHHPMYGNCYTFNNRQNETILST  300
gamma-ENaC_Inia_geoffrensis                 MAQVSLEKKINMSYSAEELLVTCFFDGESCDARNFTRFHHPMYGNCYTFNNRQNETILST  300
gamma-ENaC_Lipotes_vexillifer               MAQVSLEKKINMSYSAEELLVTCFFDGESCDARNFTRFHHPMYGNCYTFNNRQNETVLST  300
gamma-ENaC_Hyperoodon_ampullatus            MARVSPEKKINMSYSAEELLVTCFFDGESCDARNFTRFHHPMYGNCYTFNNGQNETMLST  300
gamma-ENaC_Mesoplodon_bidens                MARVSPEKKINMSYSAEELLVTCFFDGESCDARNFTRFHHPMYGNCYTFNNGQNETMLST  300
gamma-ENaC_Ziphius_cavirostris              MARVSPEKKINMSYSAEELLVTCFFDGESCDARNFTRFHHPMYGNCYTFNNGQNETMLST  300
gamma-ENaC_Platanista_gangetica             MAQVSPEKKINMSYSAEELLVTCFFDGESCDARNFTRFHHPMYGNCYTFNNRQNETILST  300
gamma-ENaC_Platanista_minor                 MAQVSPEKKINMSYSAEELLVTCFFDGESCDARNFTRFHHPMYGNCYTFNNRQNETILST  300
gamma-ENaC_Kogia_breviceps                  MAQVSPEKKINMSYSAEELLVTCFFDGESCDARNFTRFHHPMYGNCYTFNNRENETILST  299
gamma-ENaC_Physeter_catodon                 MAQVSPEKKINMSYSAEELLVTCFFDGESCDARNFTRFHHPMYGNCYTFNKGENETILST  300
gamma-ENaC_Balaenoptera_musculus            MAQVSPEKKINMSYSAEELLVTCFFDGESCDARNFTRFHHPMYGNCYTFNNGQNETILST  300
gamma-ENaC_Eubalaena_japonica               MAQVPPEKKINMSYSAEELLVTCFFDGESCDARNFTRFHHPMYGNCYTFNNGQNETILST  300
gamma-ENaC_Hippopotamus_amphibius           MAQVSLEKKINMSYSAEELLVTCFFDGVSCDARNFTRSHHPMYGNCYTFNNRQNETILST  297
gamma-ENaC_Tragulus_javanicus               MAQVSREKKINMSYSAEELLITCFFDGMSCDARNFTLFHHPMYGNCYTFNNRQNETILST  300
gamma-ENaC_Tragulus_kanchil                 MAQVSREKKINMSYSAEELLITCFFDGMSCDARNFTLFHHPMYGNCYTFNNRQNETILST  300
gamma-ENaC_Antilocapra_americana            MAQVSQEKKINMSYSAEELLVTCFFDGVSCDARNFTLFHHPMYGNCYTFNNRQNETILST  300
gamma-ENaC_Giraffa_camelopardalis           MAQVSQEKKINMSYSADELLVTCFFDGVSCDARNFTLFHHPMYGNCYTFNNRQNETILST  300
gamma-ENaC_Giraffa_tippelskirchi            MAQVSQEKKINMSYSADELLVTCFFDGVSCDARNFTLFHHPMYGNCYTFNNRQNETILST  300
gamma-ENaC_Capreolus_pygargus               MAQVSQEKKINMSYSADELLITCFFDGVSCDARNFTLFHHPMYGNCYTFNNRQNETILST  300
gamma-ENaC_Cervus_elaphus                   MAQVSQEKKINMSYSADELLITCFFDGVSCDARNFTLFHHPMYGNCYTFNNRQNETTLST  300
gamma-ENaC_Moschus_moschiferus              MAQVSQEKKINMSYSADELLVTCFFDGVSCDARNFTLFHHPMYGNCYTFNNRQNETILST  300
gamma-ENaC_Moschus_berezovskii              MAQVSQEKKINMSYSADELLVTCFFDGVSCDARNFTLFHHPMYGNCYTFNNRQNETILST  300
gamma-ENaC_Bos_grunniens                    MAQVSQEKKINMSYSADELLVTCFFDGVSCDARNFTLFHHPMYGNCYTFNNRQNETILST  300
gamma-ENaC_Bos_taurus                       MAQVSQEKKINMSYSADELLVTCFFDGVSCDARNFTLFHHPMYGNCYTFNNRQNETILST  300
gamma-ENaC_Bubalus_bubalis                  MAQVSQEKKINMSYSADELLVTCFFDGVSCDARNFTLFHHPMYGNCYTFNNRQNETILST  300
gamma-ENaC_Nanger_granti                    MAQVSQEKKINMSYSADELLVTCFFDGMSCDARNFTLFHHPMYGNCYTFNNRQNETILST  300
gamma-ENaC_Kobus_leche                      MAQVSQEKKINMSYSADELLVTCFFDGVSCDARNFTLFHHPMYGNCYTFNNRQNETILST  300
gamma-ENaC_Capra_hircus                     MAQVSQEKKINMSYSADELLVTCFFDGVSCDARNFTLFHHPMYGNCYTFNNRQNETILST  300
gamma-ENaC_Ovis_aries                       MAQVSQEKKINMSYSADELLVTCFFDGVSCDARNFTLFHHPMYGNCYTFNNRQNETILST  300
gamma-ENaC_Ovis_canadensis                  MAQVSQEKKINMSYSADELLVTCFFDGVSCDARNFTLFHHPMYGNCYTFNNRQNETILST  300
gamma-ENaC_Oreamnos_americanus              MAQVSQEKKINMSYSADELLVTCFFDGVSCDARNFTLFHHPMYGNCYTFNNRQNETILST  300
gamma-ENaC_Hippotragus_niger                MAQVSQEKKINMSYSADELLVTCFFDGVSCDARNFTLFHHPMYGNCYTFNNRQNETILST  300
gamma-ENaC_Damaliscus_lunatus               MAQVSQEKKINMSYSADELLVTCFFDGVSCDARNFTLFHHPMYGNCYTFNNRQNETILST  300
gamma-ENaC_Sus_scrofa                       MAQVPLEKKINMSYSAEELLVTCFFDGVSCDARNFTLFHHPMYGNCYTFNNRENETTLST  300
gamma-ENaC_Vicugna_pacos                    MAQVSLEKKINMSYSAEELLVNCFFDGVSCDARNFTLFHHPMYGNCYTFNNRQNETVLST  300
gamma-ENaC_Equus_callabus                   MAQVSLEKKINMSYSAEELLVTCFFDGVSCDARNFTLFHHPMYGNCYTFNNRQNETILST  300
```

```
                                             **:*  **********:***:.***** ********  ****:*******: :*** ***

gamma-ENaC_Homo_sapiens                      SMGGSEYGLQVILYINEEEYNPFLVSSTGAKVIIHRQDEYPFVEDVGTEIETAMVTSIGM  357
gamma-ENaC_Globicephala_melas                SMGGSEYGLQVILYLNEEEYNPFLVSSTGAKVIVHRQFEYPFIEDVGTEIETAMATSIGM  360
gamma-ENaC_Lagenorhynchus_obliquidens        SMGGSEYGLQVILYLNEEEYNPFLVSSTGAKVIVHRQFEYPFIEDVGTEIETAMATSIGM  360
gamma-ENaC_Tursiops_truncatus                SMGGSEYGLQVILYLNEEEYNPFLVSSTGAKVIVHRQFEYPFIEDVGTEIETAMATSIGM  360
gamma-ENaC_Orcinus_orca                      SMGGSEYGLQVILYLNEEEYNPFLVSSTGAKVIVHRQFEYPFIEDVGTEIETAMATSIGM  360
gamma-ENaC_Phocoena_sinus                    SMGGSEYGLQVILYLNEEEYNPFLVSSTGAKVIVHRQYEYPFIEDVGTEIETAMATSIGM  360
gamma-ENaC_Neophocaena_asiaeorientalis       SMGGSEYGLQVILYLNEEEYNPFLVSSTGAKVIVHRQYEYPFIEDVGTEIETAMATSIGM  360
gamma-ENaC_Monodon_monocerons                SMGGSEYGLQVILYLNEEEYNPFLVSSTGAKVIVHRQYEYPFIEDVGTEIETAMATSIGM  360
gamma-ENaC_Delphinapterus_leucas             SMGGSEYGLQVILYLNEEEYNPFLVSSTGAKVIVHRQYEYPFIEDVGTEIETAMATSIGM  360
gamma-ENaC_Pontoporia_blainvillei            SMGGSEYGLQVILYINEEEYNPFLVSSTGAKVIVHRQDEYPFIEDVGTEIETAMATSIGM  360
gamma-ENaC_Inia_geoffrensis                  SMGGSEYGLQVILYINEEEYNPFLVSSTGAKVIVHRQDEYPFIEDVGTEIETAMATSIGM  360
gamma-ENaC_Lipotes_vexillifer                SVGGSEYGLQVILYINEEEYNPFLVSSTGAKVVVHRQDEYPFIEDVGTEIETAMATSIGM  360
gamma-ENaC_Hyperoodon_ampullatus             SMGGSDYGLQVILYIDEEEYNPFLVSSTGAKVIVHRQDEYPFIEDVGTEIETAMATSIGM  360
gamma-ENaC_Mesoplodon_bidens                 SMGGSDYGLQVILYIDEEEYNPFLVSSTGAKVIVHRQDEYPFIEDVGTEIETAMATSIGM  360
gamma-ENaC_Ziphius_cavirostris               SMGGSDYGLQVILYIDEEEYNPFLVSSTGAKVIVHRQDEYPFIEDVGTEIETAMATSIGM  360
gamma-ENaC_Platanista_gangetica              SMGGSEYGLQVILYINEEEYNPFLVSSTGAKVIVHQQDEYPFIEDVGTEIETAMATSIGM  360
gamma-ENaC_Platanista_minor                  SMGGSEYGLQVILYINEEEYNPFLVSSTGAKVIVHQQDEYPFIEDVGTEIETAMATSIGM  360
gamma-ENaC_Kogia_breviceps                   SMGGSEYGLQVILYINEEEYNPFLVSSTGAKVIVHRQDEYPFIEDVGTEIETAMATSIGM  359
gamma-ENaC_Physeter_catodon                  SMGGSEYGLQVILYINEEEYNPFLVSSTGAKVIVHRQDEYPFIEDVGTEIETAMATSIGM  360
gamma-ENaC_Balaenoptera_musculus             SMGGSEYGLQVILYINEEEYNPFLVSSTGAKVIVHRQDEYPFIEDVGTEIETAMATSIGM  360
gamma-ENaC_Eubalaena_japonica                SMGGSEYGLQVILYINEEEYNPFLVSSTGAKVIVHRQDEYPFIEDVGTEIETAMATSIGM  360
gamma-ENaC_Hippopotamus_amphibius            SMGGSEYGLQVILYINEEEYNPFLVSSTGAKVIVHRQDQYPFIEDMGTEIETAMATSIGM  357
gamma-ENaC_Tragulus_javanicus                SMGGSEFGLQVILYINEEEYNPFLVSSTGAKVIVHRQDEYPFVEEMGTEIETAMATSIGM  360
gamma-ENaC_Tragulus_kanchil                  SMGGSEFGLQVILYINEEEYNPFLVSSTGAKVIVHRQDEYPFVEEMGTEIETAMATSIGM  360
gamma-ENaC_Antilocapra_americana             SMGGSEFGLQVILYINEEEYNPFLVSSTGAKVIVHRQDEYPFVEDVGTEIETAMATSIGM  360
gamma-ENaC_Giraffa_camelopardalis            SMGGSEFGLQVILYVNEEEYNPFLVSSTGAKVIVHRQDEYPFVEDMGTEIETAMATSIGM  360
gamma-ENaC_Giraffa_tippelskirchi             SMGGSEFGLQVILYVNEEEYNPFLVSSTGAKVIVHRQDEYPFVEDMGTEIETAMATSIGM  360
gamma-ENaC_Capreolus_pygargus                SMGGSEFGLQVILYINEEEYNPFLVSSTGAKVIVHRQDEYPFAEDVGTEIETAMATSIGM  360
gamma-ENaC_Cervus_elaphus                    SMGGSEFGLQVILYINEEEYNPFLVSSTGAKVIVHRQDEYPFVEDVGTEIETAMATSIGM  360
gamma-ENaC_Moschus_moschiferus               SMGGSEFGLQVILYINEEEYNPFLVSSTGAKVIVHRQDEYPFVEDVGTEIETAMATSIGM  360
gamma-ENaC_Moschus_berezovskii               SMGGSEFGLQVILYINEEEYNPFLVSSTGAKVIVHRQDEYPFVEDVGTEIETAMATSIGM  360
gamma-ENaC_Bos_grunniens                     SMGGSEFGLQVILYINEEEYNPFLVSSTGAKVIIHRQDEYPFVEDVGTEIETAMATSIGM  360
gamma-ENaC_Bos_taurus                        SMGGSEFGLQVILYINEEEYNPFLVSSTGAKVIIHRQDEYPFVEDVGTEIETAMATSIGM  360
gamma-ENaC_Bubalus_bubalis                   SMGGSEFGLQVILYINEEEYNPFLVSSTGAKVIVHRQDEYPFIEDVGTEIETAMATSIGM  360
gamma-ENaC_Nanger_granti                     SMGGSEFGLQVILYINEEEYNPFLVSSTGAKVIVHRQDEYPFVEDVGTEIETAMATSIGM  360
gamma-ENaC_Kobus_leche                       SMGGSEFGLQVILYINEEEYNPFLVSSTGAKVIVHRQDEYPFVENMGTEIETAMATSIGM  360
gamma-ENaC_Capra_hircus                      SMGGSEFGLQVILYINEEEYNPFLVSSTGAKVIVHRQDEYPFIEDMGTEIETAMATSIGM  360
gamma-ENaC_Ovis_aries                        SMGGSEFGLQVILYINEEEYNPFLVSSTGAKVIVHRQDEYPFIEDMGTEIETAMATSIGM  360
gamma-ENaC_Ovis_canadensis                   SMGGSEFGLQVILYINEEEYNPFLVSSTGAKVIVHRQDEYPFIEDMGTEIETAMATSIGM  360
gamma-ENaC_Oreamnos_americanus               SMGGSEFGLQVILYINEEEYNPFLVSSTGAKVIVHRQDEYPFVEDVGTEIETAMATSIGM  360
gamma-ENaC_Hippotragus_niger                 SMGGSEFGLQVILYINEEEYNPFLVSSTGAKVIVHRQDEYPFVEDMGTEIETAMATSIGM  360
gamma-ENaC_Damaliscus_lunatus                SMGGSEFGLQVILYINEEEYNPFLVSSTGAKVIVHRQDEYPFVEDAGTEIETAMATSIGM  360
gamma-ENaC_Sus_scrofa                        SMGGSEYGLQVILYVNEEEYNPFLVSSTGAKVIVHRQDEYPFIEDVGTEIETAMATSIGM  360
gamma-ENaC_Vicugna_pacos                     SMGGSEYGLQVILHINEEEYNPFLVSSTGAKVIVHRQDEYPFIEDVGTEIETAMATSIGM  360
gamma-ENaC_Equus_callabus                    SMGGSEYGLQVILHINEEEYNPFLVSSTGAKVIIHRQDEYPFVEDVGTEIETAMATSIGM  360
                                             *:***::******::****************::*:* :*** *: ********.*****

gamma-ENaC_Homo_sapiens                      HLTESFKLSEPYSQCTEDGSDVPIRNIYNAAYSLQICLHSCFQTKMVEKCGCAQYSQPLP  417
gamma-ENaC_Globicephala_melas                HLTESFKLSEPYSRCTEDWNGVLITNIYNATYSLQICLHSCFQAKMVEKCGCAQFSQPLP  420
gamma-ENaC_Lagenorhynchus_obliquidens        HLTESFKLSEPYSRCTEDWNGVLITNIYNATYSLQICLHSCFQAKMVEKCGCAQFSQPLP  420
gamma-ENaC_Tursiops_truncatus                HLTESFKLSEPYSRCTEDWNGVLITNIYNATYSLQICLHSCFQAKMVEKCGCAQFSQPLP  420
gamma-ENaC_Orcinus_orca                      HLTESFKLSEPYSRCTEDWNGVLITNIYNATYSLQICLHSCFQAKMVEKCGCAQFSQPLP  420
gamma-ENaC_Phocoena_sinus                    HLTESFKLSEPYSRCTEDWNGVLITNIYNATYSLQICLHSCFQAKMVEKCGCAQFSQPLP  420
gamma-ENaC_Neophocaena_asiaeorientalis       HLTESFKLSEPYSRCTEDWNGVLITNIYNATYSLQICLHSCFQAKMVEKCGCAQFSQPLP  420
gamma-ENaC_Monodon_monocerons                HLTESFKLSEPYSRCTEDWNGVLITNIYNATYSLQICLHSCFQAKMVEKCGCAQFSQPLP  420
gamma-ENaC_Delphinapterus_leucas             HLTESFKLSEPYSRCTEDWNGVLITNIYNATYSLQICLHSCFQAKMVEKCGCAQFSQPLP  420
gamma-ENaC_Pontoporia_blainvillei            HLTESFKLSEPYSQCTEDWSDVLITNIYNATYSLQICLHSCFQAKMVEKCGCAQFSQPLP  420
gamma-ENaC_Inia_geoffrensis                  HLTESFKLSEPYSQCTEDWSDVLITNIYNATYSLQICLHSCFQAKMVEKCGCAQFSQPLP  420
gamma-ENaC_Lipotes_vexillifer                HLTESFKLSEPYSQCKEDWSNVLITNIYNAPYSLQICLHSCFQAKMVEKCGCAQFSQPLP  420
gamma-ENaC_Hyperoodon_ampullatus             HLTESFKLSDPYSRCMEDWSDVLITNIYNATYSLRICLHSCFQAKMVEKCGCAQFSQPLP  420
gamma-ENaC_Mesoplodon_bidens                 HLTESFKLSDPYSRCTEDWSDVLITNIYNATYSLRICLHSCFQAKMVEKCGCAQFSQPLP  420
gamma-ENaC_Ziphius_cavirostris               HLTESFKLSDPYSRCMEDWSDVLITNIYNATYSLRICLHSCFQAKMVEKCGCAQFSQPLP  420
gamma-ENaC_Platanista_gangetica              HLTESFKLSEPYSQCTEDWSDVLITNIYNATYSLQICLHSCFQAKMVEKCGCAQFSQPLP  420
gamma-ENaC_Platanista_minor                  HLTESFKLSEPYSQCTEDWSDVLITNIYNATYSLQICLHSCFQAKMVEKCGCAQFSQPLP  420
gamma-ENaC_Kogia_breviceps                   HLTESFKLSEPYSQCTEDWSDVLITNIYNATYSLQICLHSCFQAKMVEKCGCAQFSQPLP  419
gamma-ENaC_Physeter_catodon                  HLTESFKLSEPYSRCTEDWSDVLIKNIYNATYSLQICLHSCFQAKMVEKCGCAQFNQPLP  420
gamma-ENaC_Balaenoptera_musculus             HLTESFKLSEPYSKCTEDWSDVLITNIYNATYSLQICLHSCFQAKMVEKCGCAQFSQPLP  420
gamma-ENaC_Eubalaena_japonica                HLTESFKLSEPYSKCTEDWSDVLITNIYNATYSLQICLHSCFQAKMVEKCGCAQFSQPLP  420
gamma-ENaC_Hippopotamus_amphibius            HLTESFKLSEPYSQCTEDWRDVPVMNIYNATYSLQICLHSCFQAKMVENCGCAQYSQPLP  417
gamma-ENaC_Tragulus_javanicus                HLTESFKLSDPYSQCTEDWGDVQIKNIYNATYSLQICLHSCFQTKMVEKCGCAQYSQPLP  420
gamma-ENaC_Tragulus_kanchil                  HLTESFKLSDPYSQCTEDWGDVQIKNIYNATYSLQICLHSCFQTKMVEKCGCAQYSQPLP  420
gamma-ENaC_Antilocapra_americana             HLTESFKLSDPYSQCTEDWSDVQIRNIYNATYSLQICLHSCFQAKMVENCGCAQYSQPLP  420
gamma-ENaC_Giraffa_camelopardalis            HLTESFKLSDPYSQCTEDWSDVQITNIYNATYSLQICLHSCFQAKMVENCGCAQYSQPLP  420
gamma-ENaC_Giraffa_tippelskirchi             HLTESFKLSDPYSQCTEDWSDVQITNIYNATYSLQICLHSCFQAKMVENCGCAQYSQPLP  420
gamma-ENaC_Capreolus_pygargus                HLTESFKLSDPYSQCTEDWSDVQITNIYNATYSSQICLHSCFQAKMVENCGCAQYSQPLP  420
gamma-ENaC_Cervus_elaphus                    HLTESFKLSDPYSQCTEDWSDVQITNIYNATYSPQICLHSCFQAKMVENCGCAQYSQPLP  420
gamma-ENaC_Moschus_moschiferus               HLTESFKLSDPYSQCTEDWSDVQITNIYNATYSLQICLHSCFQAKMVENCGCAQYSQPLP  420
gamma-ENaC_Moschus_berezovskii               HLTESFKLSDPYSQCTEDWSDVQITNIYNATYSLQICLHSCFQAKMVENCGCAQYSQPLP  420
gamma-ENaC_Bos_grunniens                     HLTESFKLSDPYSQCTEDWSDVQITNIYNATYSLQICLHSCFQAKMVENCGCAQYSQPLP  420
gamma-ENaC_Bos_taurus                        HLTESFKLSDPYSQCTEDWSDVQITNIYNATYSLQICLHSCFQAKMVENCGCAQYSQPLP  420
gamma-ENaC_Bubalus_bubalis                   HLTESFKLSDPYSQCTEDWSDVQITNIYNATYSLQICLHSCFQAKMVENCGCAQYSQPLP  420
gamma-ENaC_Nanger_granti                     HLTESFKLSDPYSHCTEDWSDVQITNIYNATYSLQICLHSCFQAKMVENCGCAQYSQPLP  420
```

```
gamma-ENaC_Kobus_leche                        HLTESFKLSDPYSHCTEDWSDVQITNIYNATYSLQICLHSCFQAKMVENCGCAQYSQPLP  420
gamma-ENaC_Capra_hircus                       HLTESFKLSDPYSRCTEDWSDVQITNIYNATYSLQICLHSCFQAKMVENCGCAQYSQPLP  420
gamma-ENaC_Ovis_aries                         HLTESFKLSDPYSRCTEDWSDVQITNIFNATYSLQICLHSCFQAKMVENCGCAQYSQPLP  420
gamma-ENaC_Ovis_canadensis                    HLTESFKLSDPYSRCTEDWSDVQITNIFNATYSLQICLHSCFQAKMVENCGCAQYSQPLP  420
gamma-ENaC_Oreamnos_americanus                HLTESFKLSDPYSHCTEDWSDVQITNIYNATYSLQICLHSCFQAKMVENCGCAQYSQPLP  420
gamma-ENaC_Hippotragus_niger                  HLTESFKLSDPYSHCTEDWSDVQITNIYNATYSLQICLHSCFQAKMVENCGCAQYSQPLP  420
gamma-ENaC_Damaliscus_lunatus                 HLTESFKLSDPYSHCTEDWSDVQITNIYNATYSLQICLHSCFQAKMVENCGCAQYSQPLP  420
gamma-ENaC_Sus_scrofa                         HLTESFKLGEPYSQCTEDGSDVPVENIYGAAYSLQICLNSCFQAKMVEKCGCAQYSKPLP  420
gamma-ENaC_Vicugna_pacos                      HLTESFKLSEPYSQCTEDGSEVPIQNIYNASYSLQICLHSCFQAKMVEKCGCAQYSKPLP  420
gamma-ENaC_Equus_callabus                     HLTESFKLSEPYSQCTEDGSDVPVENIYKAAYSLKICLHSCFQTKMVEKCGCAQYSQPLP  420
                                              ********.:***:* **   * : **: * ** :***:****:****:*****:.:***

gamma-ENaC_Homo_sapiens                       PAANYCNYQQHPNWMYCYYQLHRAFVQEELGCQSVCKEACSFKEWTLTTSLAQWPSVVSE  477
gamma-ENaC_Globicephala_melas                 QGANYCNYRQHPNWMYCYYELHQAFVWGELGCQAMCKEACSFKEWTLTTSLAQWPSEVSE  480
gamma-ENaC_Lagenorhynchus_obliquidens         QGANYCNYRQHPNWMYCYYELHQAFVWGELGCQSMCKEACSFKEWTLTTSLAQWPSEVSE  480
gamma-ENaC_Tursiops_truncatus                 QGANYCNYRQHPNWMYCYYELHQAFVWGELGCQSMCKEACSFKEWTLTTSLAQWPSEVSE  480
gamma-ENaC_Orcinus_orca                       QGANYCNYRQHPNWMYCYYELHQAFVWGELGCQSMCKEACSFKEWTLTTSLAQWPSEVSE  480
gamma-ENaC_Phocoena_sinus                     QGANYCNYRQHPNWMYCYYELHQAFVWGELGCQSVCKEACSFKEWTLTTSLAQWPSEVSE  480
gamma-ENaC_Neophocaena_asiaeorientalis        QGANYCNYRQHPNWMYCYYELHQAFVWGELGCQSVCKEACSFKEWTLTTSLAQWPSEVSE  480
gamma-ENaC_Monodon_monocerons                 QGANYCNYRQHPNWMYCYYELHQAFVWGELGCQSVCKEACSFKEWTLTTSLAQWPSEVSE  480
gamma-ENaC_Delphinapterus_leucas              QGANYCNYRQHPNWMYCYYELHQAFVWGELGCQSVCKEACSFKEWTLTTSLAQWPSEVSE  480
gamma-ENaC_Pontoporia_blainvillei             RGASYCNYRQHPNWMYCYYRLHQAFVWGELGCQSVCKEACSFKEWTLTTSLAQWPSEVSE  480
gamma-ENaC_Inia_geoffrensis                   RGASYCNYRQHPNWMYCYYELHQAFVWGELGCQSVCKEACSFKEWTLTTSLAQWPSEVSE  480
gamma-ENaC_Lipotes_vexillifer                 QGANYCNYRQHPNWMYCYYELHQAFVWGELGCQSVCKEACSFKEWTLTTSLAQWPSEVSE  480
gamma-ENaC_Hyperoodon_ampullatus              QGANYCNYQQHPNWMYCYYELHKDFVRGELGCQSVCKEACSFKEWTLTTSLAQWPSEVSE  480
gamma-ENaC_Mesoplodon_bidens                  QGANYCNYQQHPNWMYCYYELHKDFVRGELGCQSVCKEACSFKEWTLTTSLAQWPSEVSE  480
gamma-ENaC_Ziphius_cavirostris                QGANYCNYQQHPNWMYCYYELHEDFVRGELGCQSVCKEACSFKEWTLTTSLAQWPSEVSE  480
gamma-ENaC_Platanista_gangetica               RGANYCNYQQHPNWMYCYYELHQAFVREELGCQSLCKEACSFKEWTLTTSLAQWPSEVSE  480
gamma-ENaC_Platanista_minor                   RGANYCNYQQHPNWMYCYYELHQAFVREELGCQSLCKEACSFKEWTLTTSLAQWPSEVSE  480
gamma-ENaC_Kogia_breviceps                    QGVNYCNYQQHPNWMYCYYELHQDFVREELGCQSLCKEACSFKEWTLTTSLAQWPSEVSE  479
gamma-ENaC_Physeter_catodon                   QGVNYCNYQQHPNWMYCYYELHQDFVREELGCQSMCKEACSFKEWTLTTSLAQWPSEVSE  480
gamma-ENaC_Balaenoptera_musculus              RGANYCNYQQHPNWMYCYYELHQDFVREELGCQALCKEACSFKEWTLTTSLAQWPSEVSE  480
gamma-ENaC_Eubalaena_japonica                 RGANYCNYQQHPNWMYCYYELHQDFVREELGCQVLCKEACSFKEWTLTTSLAQWPSEVSE  480
gamma-ENaC_Hippopotamus_amphibius             QGANYCNYQQHPNWMYCYYQLHQAFVREELGCQSVCKEACSFKEWTLTTSLAQWPSEVSE  477
gamma-ENaC_Tragulus_javanicus                 RGADYCNYQQHPNWMYCYYQLHQAFVREELGCQSVCKEACSFKEWTLTTSLAQWPSEVSE  480
gamma-ENaC_Tragulus_kanchil                   RGADYCNYQQHPNWMYCYYQLHQAFVREELGCQSVCKEACSFKEWTLTTSLAQWPSEVSE  480
gamma-ENaC_Antilocapra_americana              QGADYCNYQQHPNWMYCYYQLHQAFVREELGCQSVCKEACSFKEWTLTTSLAQWPSEVSE  480
gamma-ENaC_Giraffa_camelopardalis             QGANYCNYQQHPNWMYCYYQLHQAFVREELGCQSVCKEACSFKEWTLTTSLAQWPSEVSE  480
gamma-ENaC_Giraffa_tippelskirchi              QGANYCNYQQHPNWMYCYYQLHQAFVREELGCQSVCKEACSFKEWTLTTSLAQWPSEVSE  480
gamma-ENaC_Capreolus_pygargus                 QGADYCNYQQHPNWMYCYYKLHQAFVREELGCQSVCKEACSFKEWTLTTSLAQWPSEVSE  480
gamma-ENaC_Cervus_elaphus                     QGADYCNYQQHPNWMYCYYQLHQAFVREELGCQSVCKEACSFKEWTLTTSLAQWPSEVSE  480
gamma-ENaC_Moschus_moschiferus                QGADYCNYQQHPNWMYCYYQLHQAFVREELGCQSVCKEACSFKEWTLTTSLAQWPSEVSE  480
gamma-ENaC_Moschus_berezovskii                QGADYCNYQQHPNWMYCYYQLHQAFVREELGCQSVCKEACSFKEWTLTTSLAQWPSEVSE  480
gamma-ENaC_Bos_grunniens                      RGADYCNYQQHPNWMYCYYQLHQAFVREELGCQSVCKEACSFKEWTLTTSLAQWPSEVSE  480
gamma-ENaC_Bos_taurus                         RGADYCNYQQHPNWMYCYYQLHQAFVREELGCQSVCKEACSFKEWTLTTSLAQWPSEVSE  480
gamma-ENaC_Bubalus_bubalis                    RGADYCNYQQHPNWMYCYYQLHQAFVREELGCQSVCKEACSFKEWTLTTSLAQWPSEVSE  480
gamma-ENaC_Nanger_granti                      QGADYCNYQQHPNWMYCYYQLHQAFVREELGCQSVCKEACSFKEWTLTTSLAQWPSEVSE  480
gamma-ENaC_Kobus_leche                        QGADYCNYQQHPNWMYCYYQLHQAFVREELGCQSVCKEACSFKEWTLTTSLAQWPSEVSE  480
gamma-ENaC_Capra_hircus                       QGADYCNYQQHPNWMYCYYQLHQAFVREELGCQSVCKEACSFKEWTLTTSLAQWPSEVSE  480
gamma-ENaC_Ovis_aries                         QGANYCNYQQHPNWMYCYYQLHQAFVREELGCQSVCKEACSFKEWTLTTSLAQWPSEVSE  480
gamma-ENaC_Ovis_canadensis                    QGANYCNYQQHPNWMYCYYQLHQAFVREELGCQSVCKEACSFKEWTLTTSLAQWPSEVSE  480
gamma-ENaC_Oreamnos_americanus                QGADYCNYQQHPNWMYCYYQLHQAFVREELGCQSVCKEACSFKEWTLTTSLAQWPSEVSE  480
gamma-ENaC_Hippotragus_niger                  QGADYCNYQQHPNWMYCYYQLHQAFVREELGCQSVCKEACSFKEWTLTTSLAQWPSEVSE  480
gamma-ENaC_Damaliscus_lunatus                 QGADYCNYQQHPNWMYCYYQLHQAFVREELGCQSVCKEACSFKEWTLTTSLAQWPSEVSE  480
gamma-ENaC_Sus_scrofa                         PPANYCNYQQHPNWMYCYYQLSQAFVREELGCQSVCKEACSFKEWTLTTSLAQWPSEVSE  480
gamma-ENaC_Vicugna_pacos                      PKVNYCNYQQHPNWMYCFYQLHQAFVREELGCQSVCREACSFKEWSLTTSLAQWPSEVSE  480
gamma-ENaC_Equus_callabus                     PAANYCNYQQHPNWMYCYYQLHQAFVREELGCQSVCREACSFKEWTLTTSLAQWPSEVSE  480
                                                ..****:********:*.* . **  ***** :*:********:********** ***

                                                                                           TM2
gamma-ENaC_Homo_sapiens                       KWLLPVLTWDQGRQVNKKLNKTDLAKLLIFYKDLNQRSIMESPANSIEMLLSNFGGQLGL  537
gamma-ENaC_Globicephala_melas                 KWLLSVLTWDQG-QIKKKLNKTDLAKLLIFYKDLNQRSIVESPANSIEMLLSNIGGQLGL  539
gamma-ENaC_Lagenorhynchus_obliquidens         KWLLSVLTWDQG-QIKKKLNKTDLAKLLIFYKDLNQRSIVESPANSIEMLLSNIGGQLGL  539
gamma-ENaC_Tursiops_truncatus                 KWLLSVLTWDQG-QIKKKLNKTDLAKLLIFYKDLNQRSIVESPANSIEMLLSNIGGQLGL  539
gamma-ENaC_Orcinus_orca                       KWLLSVLTWDQG-QIKKKLNKTDLAKLLIFYKDLNQRSIVESPANSIEMLLSNIGGQLGL  539
gamma-ENaC_Phocoena_sinus                     KWLLSVLTWDQG-QIKKKLNKTDLAKLLIFYKDLNQRSIVESPANSIEMLLSNIGGQLGL  539
gamma-ENaC_Neophocaena_asiaeorientalis        KWLLSVLTWDQG-QIKKKLNKTDLAKLLIFYKDLNQRSIVESPANSIEMLLSNIGGQLGL  539
gamma-ENaC_Monodon_monocerons                 KWLLSVLTWDQG-QIKKKLNKTDLAKLLIFYKDLNQRSIVESPANSIEMLLSNIGGQLGL  539
gamma-ENaC_Delphinapterus_leucas              KWLLSVLTWDQG-QIKKKLNKTDLAKLLIFYKDLNQRSIVESPANSIEMLLSNIGGQLGL  539
gamma-ENaC_Pontoporia_blainvillei             KWLLSVLTWDQG-QIKKKLNKTDLAKLLIFYKDLNQRSIVESPANSIEILLSNIGGQLGL  539
gamma-ENaC_Inia_geoffrensis                   KWLLSVLTWDQG-QIKRKLNKTDLAKLLIFYKDLNQRSIVESPANSIEMLLSNIGGQLGL  539
gamma-ENaC_Lipotes_vexillifer                 KWLLSVLTWDQG-QIKKKLNKTDLAKLLIFYKDLSHRSIVESPANSIEMLLSNIGGQLGL  539
gamma-ENaC_Hyperoodon_ampullatus              KWLLSVLTWDQG-QIKKKLNKTDLAKLLIFYKDLNQRSIVESPANSIEMLLSNIGGQLGL  539
gamma-ENaC_Mesoplodon_bidens                  KWLLSVLTWDQG-QIKKKLNKTDLAKLLIFYKDLNQRSIVESPANSIEMLLSNIGGQLGL  539
gamma-ENaC_Ziphius_cavirostris                KWLLSVLTWDQG-QIKKKLNKTDLAKLLIFYKDLNQRSIVESPANSIEMLLSNIGGQLGL  539
gamma-ENaC_Platanista_gangetica               KWLLSLLTWDQG-QIKKKLNKTDLAKLLIFYKDLNQRSIVESPANSIEMLLSNIGGQLGL  539
gamma-ENaC_Platanista_minor                   KWLLSLLTWDQG-QIKKKLNKTDLAKLLIFYKDLNQRSIVESPANSIEMLLSNIGGQLGL  539
gamma-ENaC_Kogia_breviceps                    KWLLSVLTWDQG-KIKKKLNKTDLAKLLIFYKDLNQRSIVESPANSIEMLLSNIGGQLGL  538
gamma-ENaC_Physeter_catodon                   KWLLSVLTWDQG-QIKKKLNKTDLAKLLIFYKDLNQRSIVESPANSIEMLLSNIGGQLGL  539
gamma-ENaC_Balaenoptera_musculus              KWLLSVLTWDQG-QIKKKLNKTDLAKLLIFYKDLNQRSIVESPANSIEMLLSNIGGQLGL  539
gamma-ENaC_Eubalaena_japonica                 KWLLSVLTWDQG-QIKKKLNKTDLAKLLIFYKDLNQRSIVESPANSIEMLLSNIGGQLGL  539
gamma-ENaC_Hippopotamus_amphibius             KWLLSVLTWDQRQLINRKLNKTDLAKLLIYYKDLNQRSIMESPANSIEMLLSNIGGQLGL  537
gamma-ENaC_Tragulus_javanicus                 KWLLSLLTWDQSQQIKKKLNKTDLAQLLIFYKDLNQRSIMENPANSIEQLLSNIGGQLGL  540
gamma-ENaC_Tragulus_kanchil                   KWLLSLLTWDQSQQIKKKLNKTDLAQLLIFYKDLNQRSIMENPANSIEQLLSNIGGQLGL  540
```

```
gamma-ENaC_Antilocapra_americana          KWLLSVLTWDQSQQIKKKLNKTDLAKLLIFYKDLNQRSIMENPANSIEQLLSNIGGQLGL  540
gamma-ENaC_Giraffa_camelopardalis         KWLLSVLTWDQSQQIKKKLNKTDLAKLLIFYKDLNQRSIMENPANSIEQLLSNIGGQLGL  540
gamma-ENaC_Giraffa_tippelskirchi          KWLLSVLTWDQSQQIKKKLNKTDLAKLLIFYKDLNQRSIMENPANSIEQLLSNIGGQLGL  540
gamma-ENaC_Capreolus_pygargus             KWLLSVLTWDQSQQIKKKLNKTDLAKLLIFYKDLNQRSIMENPANSIEQLLSNIGGQLGL  540
gamma-ENaC_Cervus_elaphus                 KWLLSVLTWDQSQQIKKKLNKTDLAKLLIFYKDLNQRSIVENPANSIEQLLSNIGGQLGL  540
gamma-ENaC_Moschus_moschiferus            KWLLSVLTWDQSQQIKKKLNKTDLAKLLIFYKDLNQRSIMENPANSIEQLLSNIGGQLGL  540
gamma-ENaC_Moschus_berezovskii            KWLLSVLTWDQSQQIKKKLNKTDLAKLLIFYKDLNQRSIMENPANSIEQLLSNIGGQLGL  540
gamma-ENaC_Bos_grunniens                  KWLLSILTWDQSQQIKKKLNKTDLAKLLIFYKDLNQRSIMENPANSIEQLLSNIGGQLGL  540
gamma-ENaC_Bos_taurus                     KWLLSILTWDQSQQIKKKLNKTDLAKLLIFYKDLNQRSIMENPANSIEQLLSNIGGQLGL  540
gamma-ENaC_Bubalus_bubalis                KWLLSILTWDQSQQIKKKLNKTDLAKLLIFYKDLNQRSIMENPANSIEQLLSNIGGQLGL  540
gamma-ENaC_Nanger_granti                  KWLLSVLTWDQSQQIKKKLNKTDLAKLLIFYKDLNQRSIMENPANSIEQLLSNIGGQLGL  540
gamma-ENaC_Kobus_leche                    KWLLSVLTWDQSQQIKKKLNKTDLAKLLIFYKDLNQRSIMENPANSIEQLLSNIGGQLGL  540
gamma-ENaC_Capra_hircus                   KWLLSVLTWDQSQQIKKKLNKTDLAKLLIFYKDLNQRSIMENPANSIEQLLSNIGGQLGL  540
gamma-ENaC_Ovis_aries                     KWLLSVLTWDQSQQIKKKLNKTDLAKLLIFYKDLNQRSIMENPANSIEQLLSNIGGQLGL  540
gamma-ENaC_Ovis_canadensis                KWLLSVLTWDQSQQIKKKLNKTDLAKLLIFYKDLNQRSIMENPANSIEQLLSNIGGQLGL  540
gamma-ENaC_Oreamnos_americanus            KWLLSVLTWDQSQQIKKKLNKTDLAKLLIFYKDLNQRSIMENPANSIEQLLSNIGGQLGL  540
gamma-ENaC_Hippotragus_niger              KWLLSVLTWDQSQQIKKKLNKTDLAKLLIFYKDLNQRSIMENPANSIEQLLSNIGGQLGL  540
gamma-ENaC_Damaliscus_lunatus             KWLLSVLTWDQSQQIKKKLNKTDLAKLLIFYKDLNQRSIMENPANSIEQLLSNIGGQLGL  540
gamma-ENaC_Sus_scrofa                     KWLLSVLTWDQGQQIKKKLNKTDLAKLLIFYKDLNQRSIMESPANSIEMLLSNIGGQLGL  540
gamma-ENaC_Vicugna_pacos                  KWLLSVLTWDQSQQIKKKLNKTDLAKLLIFYKDLNQRSIMESPANSIEMLLSNIGGQLGL  540
gamma-ENaC_Equus_callabus                 KWLLSVLTWDQGLQIKKKLNKTDLAKLLIFYKDLNQRSIMENPANSIEMLLSNFGGQLGL  540
                                          **** :*****   :::********:***:****.:***:*.****** ****:******

gamma-ENaC_Homo_sapiens                   WMSCSVVCVIEIIEVFFIDFFSIIARRQWQKAKEWWAWKQAPPCPEAP-RSPQGQDNPAL  596
gamma-ENaC_Globicephala_melas             WMSCSIVCIIEIIEVFFIDSLSIVARHQWHKAKEWWARRQASPCPEAPP-SPQGQDNLGL  598
gamma-ENaC_Lagenorhynchus_obliquidens     WMSCSIVCIIEIIEVFFIDSLSIVARHQWHKAKEWWARRQASPCPEAPP-SPQGQDNLGL  598
gamma-ENaC_Tursiops_truncatus             WMSCSIVCIIEIIEVFFIDSLSIVARHQWHKAKQWWARRQASPCPEAPP-SPQGQDNLGL  598
gamma-ENaC_Orcinus_orca                   WMSCSIVCIIEIIEVFFIDSLSIVARHQWHKAKEWWARRQASPCPEAPP-SPQGQDNLGL  598
gamma-ENaC_Phocoena_sinus                 WMSCSIVCIIEIIEVFFIDSLSIVARHQWHKAKEWWARRQASPCPEAPP-SPQGQDNLGL  598
gamma-ENaC_Neophocaena_asiaeorientalis    WMSCSIVCIIEIIEVFFIDSLSIVARHQWHKAKEWWARRQASPCPEAPP-SPQGQDNLGL  598
gamma-ENaC_Monodon_monocerons             WMSCSIVCIIEIIEVFFIDSLSIVARHQWHKAKEWWARRQASPCPEAPP-SPQGQDNLGL  598
gamma-ENaC_Delphinapterus_leucas          WMSCSIVCIIEIIEVFFIDSLSIVARHQWHKAKEWWARRQASPCPEAPP-SPQGQDNLGL  598
gamma-ENaC_Pontoporia_blainvillei         WMSCSIVCIIEIIEVFFIDSLSIVARHQWHKAKEWWARRQASPCPEAPH-SPQGQDNLGL  598
gamma-ENaC_Inia_geoffrensis               WMSCSIVCIIEIIEVFFIDSLSIVARHQWHKAKEWWARRQATPCPEAPP-SPQGQDNLGL  598
gamma-ENaC_Lipotes_vexillifer             WMSCSIICIIEIIEVFFIDSLSIVARHQWHKAKEWWARRQASPCPEAPP-SPQGQDNLGL  598
gamma-ENaC_Hyperoodon_ampullatus          WLSCSIVCVIEIIEVFFIDSLSIVARHQWHKAKEWWARRQASPRPEAPP-SPQGQDNLGL  598
gamma-ENaC_Mesoplodon_bidens              WLSCSIVCVIEIIEVFFIDSLSIVARHQWHKAKEWWARRQASPHPEAPP-SPQGQDNLGL  598
gamma-ENaC_Ziphius_cavirostris            WLSCSIVCVIEIIEVFFIDSLSIVARHQWHKAKEWWARRQASPRPEAPP-SPQGQDNLGL  598
gamma-ENaC_Platanista_gangetica           WMSCSIVCVIEIIEVFFIDSLSIVARHQWHKAKEWWARRQASPCPEAPP-SPQGQDNLGL  598
gamma-ENaC_Platanista_minor               WMSCSIVCVIEIIEVFFIDSLSIVARHQWHKAKEWWARRQASPCPEAPP-SPQGQDNLGL  598
gamma-ENaC_Kogia_breviceps                WMSCSIVCIIEIIEVFFIDSLSIVARHQWHKAKEWWARRQATTCPEAPP-SPQGRDNLGL  597
gamma-ENaC_Physeter_catodon               WMSCSIVCVIEIIEVFFIDSLSIVARHQWHKAKEWWARRQASTCPEAPP-SPQGQDNLGL  598
gamma-ENaC_Balaenoptera_musculus          WMSCSVVCVIEIVEVFLVDSLSIVARHQWRKAKEWWARRQAPPWPEAPP-SPQGRDNLGL  598
gamma-ENaC_Eubalaena_japonica             WMSCSVVCIIEIVEVFLVDSLSIVARHQWRKAKEWWARRQAPPWPEATH-SPQGRDNLGL  598
gamma-ENaC_Hippopotamus_amphibius         WMSCSVVCVIEIIEVFFIDSLSIIARHQWHKAKGWWARRQAPPCPEAP-PSPQGQDNPGL  596
gamma-ENaC_Tragulus_javanicus             WMSCSVVCVIEIIEVFFIDSLSIIARHQWHKARDWWARRRAPPCPEAP-RSPQGQDNPGL  599
gamma-ENaC_Tragulus_kanchil               WMSCSVVCVIEIIEVFFIDSLSIIARHQWHKARDWWARRRAPPCPEAP-RSPQGQDNPGL  599
gamma-ENaC_Antilocapra_americana          WMSCSVVCVIEIIEVFFIDSLSIIARHQWHKAKGWWARRQAPTCPEAP-RRPQGQDNAGL  599
gamma-ENaC_Giraffa_camelopardalis         WMSCSVVCVIEIIEVFFIDSLSIIARHQWHKAKGWWARRRAPTCPEAP-RSPQGQDNPGL  599
gamma-ENaC_Giraffa_tippelskirchi          WMSCSVVCVIEIIEVFFIDSLSIIARHQWHKAKGWWARRRAPTCPEAP-RSPQGQDNPGL  599
gamma-ENaC_Capreolus_pygargus             WMSCSVVCVIEIIEVFFIDSLSIIARQQWHKAKGWWARRRAPACPEAP-RTRQGRVNPGL  599
gamma-ENaC_Cervus_elaphus                 WMSCSIVCVIEIIEVFFIDSLSIIARQQWHKAKGWWARRRAPACPEAP-RTRQGRVNPGL  599
gamma-ENaC_Moschus_moschiferus            WMSCSVVCVIEIIEVFFIDSLSIIARHQWHKAKGWWAQRRAPACPEAP-HTPQGRDNPGL  599
gamma-ENaC_Moschus_berezovskii            WMSCSVVCVIEIIEVFFIDSLSIIARHQWHKAKGWWAQRRAPACPEAP-HTPQGRDNPGL  599
gamma-ENaC_Bos_grunniens                  WMSCSVVCVIEIIEVFFIDSLSIIARHQWHKAKGWWARRRAPACPEAP-RAPQGRDNPSL  599
gamma-ENaC_Bos_taurus                     WMSCSVVCVIEIIEVFFIDSLSIIARHQWHKAKGWWARRRAPACPEAP-RAPQGRDNPSL  599
gamma-ENaC_Bubalus_bubalis                WMSCSVVCIIEIIEVFFIDSLSIIARHQWHKAKRWWARRRAPACPEAP-RAPQGRDNPSL  599
gamma-ENaC_Nanger_granti                  WMSCSVVCVIEIIEVFFIDSLSIIARHQWHKAKGWWARRRAPTCPEAP-RIPQGRDNPGL  599
gamma-ENaC_Kobus_leche                    WMSCSVVCVIEIIEVFFIDSLSIIARHQWHKAKGWWARRRAPACPEVP-RIPQGRDNPGL  599
gamma-ENaC_Capra_hircus                   WMSCSVVCVIEIIEVFFIDSLSIIARHQWHKAKGWWARRRAPACPEAP-HIPQGRDNPGL  599
gamma-ENaC_Ovis_aries                     WMSCSVVCVIEIIEVFFIDSLSIIARHQWHKAKGWWARRRAPACPEAP-HIPQGRDNPGL  599
gamma-ENaC_Ovis_canadensis                WMSCSVVCVIEIIEVFFIDSLSIIARHQWHKAKGWWARRRAPACPEAP-HIPQGRDNPGL  599
gamma-ENaC_Oreamnos_americanus            WMSCSIVCVIEIIEVFFIDSLSIIARHQWHKAKGWWARRRAPACPEAP-HIPHGRDNPGL  599
gamma-ENaC_Hippotragus_niger              WMSCSVVCVIEIIEVFFIDSLSIIARHQWHKAKGWWARRRAPTCHEAP-HIPQGRDNPGL  599
gamma-ENaC_Damaliscus_lunatus             WMSCSVVCVIEIIEVFFIDSLSIIARHQWHKAKGWWARRRAPTCCEAP-RIPQGRDNPGL  599
gamma-ENaC_Sus_scrofa                     WMSCSVVCVIEIIEVFFIDSLSIIARHQWHKAKGWWARRQAPPCPEAP-PSPQGRDNPAL  599
gamma-ENaC_Vicugna_pacos                  WMSCSVVCVIEIIEVFFIDSLSIIARHQWHKAKEWWARRQAPACPEAPPRSPQGQVNPVL  600
gamma-ENaC_Equus_callabus                 WMSCSIVCIIEIVEVFFIDSLSIIARQQWQKAKQWWAQRQAPPCPEAPP-SPQGQDNPVL  599
                                          *:***::*:***:***::* :**:**:**:**: *** ::*    *.     :*: *  *

gamma-ENaC_Homo_sapiens                   DIDDDLPTFNSALHLPPALGTQVPGTPPPKYNTLRLERAFSNQLTDTQMLDEL---      649
gamma-ENaC_Globicephala_melas             NIDDDLPTFTSALCLPPAPAAQVPGTPPPRYNTLRLERAFSRQLTGTEVSAES---      651
gamma-ENaC_Lagenorhynchus_obliquidens     NIDDDLPTFTSALCLPPAPAAQVPGTPPPRYNTLRLERAFSRQLTGTEVSAES---      651
gamma-ENaC_Tursiops_truncatus             NIDDDLPTFTSALCLPPAPAAQVPGTPPPRYNTLRLERAFSRQLTGTEVSAES---      651
gamma-ENaC_Orcinus_orca                   HIDDDLPTFTSALCLPPAPAAQVPGTPPPRYNTLRLERAFSRQLTGTEVSAES---      651
gamma-ENaC_Phocoena_sinus                 NIDDDLPTFTSALCLPPAPAAQVPGTPPPRYNTLSLERAFSRQLTGTEVPAES---      651
gamma-ENaC_Neophocaena_asiaeorientalis    NIDDDLPTFTSALCLPPAPAAQVPGTPPPRYNTLSLERAFSRQLTGTEVPAES---      651
gamma-ENaC_Monodon_monocerons             NIDDDLPTFTSALCLPPAPAAQVPGTPPPRYNTLRLERTFSRQLTGTEVPAES---      651
gamma-ENaC_Delphinapterus_leucas          NMDDDLPTFTSALCLPPAPAAQVPGTPPPRYNTLRLERTFSRQLTGTEVPAES---      651
gamma-ENaC_Pontoporia_blainvillei         NIDDDLPTFTSALGLPPAPAAQVPGTPPPRYNTLRLERAFSRQLTDTEVPAES---      651
gamma-ENaC_Inia_geoffrensis               NIDDDLPTFTSALCLPPAPAAQVPGTPPPRYNTLRLERAFSRQLTDTEVPAES---      651
gamma-ENaC_Lipotes_vexillifer             NIDDDLPTFTSALCLPPAPAAQVPGTPPPRYNTLRLERAFSRQLTGTEVPAES---      651
gamma-ENaC_Hyperoodon_ampullatus          HIDDDLPTFTSALCLPPAPAAQVPGTPPPRYNTLRLERAFSRQLTGTEVPAES---      651
gamma-ENaC_Mesoplodon_bidens              HIDDDLPTFTSALCLPPAPAAQVPGTPPPRYNTLRLERAFSRQLTGTEVPAES---      651
```

```
gamma-ENaC_Ziphius_cavirostris           HIDDDLPTFTSALCLPPAPAAQVPGTPPPRYNTLSLERAFSRQLTGTEVPAES---     651
gamma-ENaC_Platanista_gangetica          NIDDDLPTFTSALCLPPAPAAQVPGTPPPRYNTLRLERAFSRQLTDTEVPAES---     651
gamma-ENaC_Platanista_minor              NIDDDLPTFTSALCLPPAPAAQVPGTPPPRYNTLRLERAFSRQLTDTEVPAES---     651
gamma-ENaC_Kogia_breviceps               DVDDDLPTFTSALCLPPAPAAQVPGTPPPRYNTLRLDRAFSRQLTDTEVPAES---     650
gamma-ENaC_Physeter_catodon              DVDDDLPTFTSALCLPPAPGGQVPGTPPPRYNTLRLDRTFSRQLTDTEVPAES---     651
gamma-ENaC_Balaenoptera_musculus         DIDDDLPTFTSALCLPPAPAAQVPGTPPPRYNTLRLERDFPSQLADTEVPAES---     651
gamma-ENaC_Eubalaena_japonica            DIDDDLPTFTSALCLPPAPAAQVPGTPPPRYNTLRLERDFPSQLADTEVPAES---     651
gamma-ENaC_Hippopotamus_amphibius        VIDDDLPTFTSALRLPPAPAAQVPGTPPPRYNTLRLERAFASQLTDTQVPAES---     649
gamma-ENaC_Tragulus_javanicus            EIDNDLPTFTSALSLPPAPGAQVPGTPPPRYNTLRLERAFSSQLTDTQAPAES---     652
gamma-ENaC_Tragulus_kanchil              EIDNDLPTFTSALSLPPAPGAQVPGTPPPRYNTLRLERAFSSQLTDTQAPAES---     652
gamma-ENaC_Antilocapra_americana         DIDDDLPTFTSALSLPPAPGAQVPGTPPPRYNTLRLERAFSSQLTDTQVPAES---     652
gamma-ENaC_Giraffa_camelopardalis        YIDDDLPTFTSALSLPPAPGAQVPGTPPPRYNTLRLERAFSSQLTDTQVPTES---     652
gamma-ENaC_Giraffa_tippelskirchi         YIDDDLPTFTSALSLPPAPGAQVPGTPPPRYNTLRLERAFSSQLTDTQVPTES---     652
gamma-ENaC_Capreolus_pygargus            NIDDDLPTFTSALSLPPAPGAQVPGTPPPRYNTLRLERAFSSQLTDTQEAAES---     652
gamma-ENaC_Cervus_elaphus                DIDDDLPTFTSALSLPPAPGAQVPGTPPPRYNTLRLERAFSSQLTDTQEAAES---     652
gamma-ENaC_Moschus_moschiferus           NIDDDLPTFTSALSLPPAPGAQVPGTPPPRYNTLRLERAFSSQLTDTQVPAES---     652
gamma-ENaC_Moschus_berezovskii           NIDDDLPTFTSALSLPPAPGAQVPGTPPPRYNTLRLERAFSSQLTDTQVPAES---     652
gamma-ENaC_Bos_grunniens                 DIDDDLPTFTSALSLPPAPGSQVPGTPPPRYNTLRLERAFSSQLTDTQVPAES---     652
gamma-ENaC_Bos_taurus                    DIDDDLPTFTSALSLPPAPGSQVPGTPPPRYNTLRLERAFSSQLTDTQVPAES---     652
gamma-ENaC_Bubalus_bubalis               DIDDDLPTFTSALSLPPAPGSQVPGTPPPRYNTLRLERAFSSQLSDTQVPAES---     652
gamma-ENaC_Nanger_granti                 NIDDDLPTFTSALSLPPAPGAQVPGTPPPRYNTLRLERAFSSQLTDTQVPAES---     652
gamma-ENaC_Kobus_leche                   NIDDDLPTFTSALSLPPAPGAQVPGTPPPRYNTLRLERAFSSQLTDTQVPAES---     652
gamma-ENaC_Capra_hircus                  NIDDDLPTFTSALSLPPAPGPQVPGTPPPRYNTLRLERAFSSQLTDTQVPAES---     652
gamma-ENaC_Ovis_aries                    NIDDDLPTFTSALSLPPAPGPQVPGTPPPRYNTLRLERAFSSQLTDTQVPAES---     652
gamma-ENaC_Ovis_canadensis               NIDDDLPTFTSALSLPPAPGPQVPGTPPPRYNTLRLERAFSSQLTDTQVPAES---     652
gamma-ENaC_Oreamnos_americanus           NIDDDLPTFTSALSLPPAPGPQVPGTPPPRYNTLRLERAFSSQLTDTQVPAES---     652
gamma-ENaC_Hippotragus_niger             NIDDDLPTFTSALSLPPAPGPQVPGTPPPRYNTLRLERAFSSQLTDTQVPAES---     652
gamma-ENaC_Damaliscus_lunatus            NIDDDLPTFTSALSLPPAPGPQVPGTPPPRYNTLRLERAFSSQLTDTQVPAES---     652
gamma-ENaC_Sus_scrofa                    NIDDDLPTFTSALRLPPAPAAQVPGTPPPRYNTLRLERAFSSQLSDTQVPAES---     652
gamma-ENaC_Vicugna_pacos                 ELDDDLPTFTSALCLPPAPGAQVPGTPPPKYNTLRLERTFSSQLTDTQVPPESPES     656
gamma-ENaC_Equus_callabus                DIDDDLPTFTSALHLPPAPGTQVPGTPPPKYNTLRLERAFSNQLTDTQVPAEF---     652
                                          :*:*****.*** **** . ********:**** *:* *  **:.*:   *
```

Supplementary Alignment 4

δ-ENaC (*SCNN1D*) amino acid sequence alignment created with Clustal Omega (v.1.2.4, accessed 24.03.2024). Putative structural motifs are highlighted based on the Alphafold2 prediction of the human ENaC subunit (https://alphafold.ebi.ac.uk/entry/A0A024R0D5; accessed 24.03.2024). Transmembrane domains (TM1/TM2) are highlighted in yellow. An N-Terminal HG-motif affecting ENaC open probability is marked in magenta. Residues putatively forming the selectivity filter within TM2 are shown in red font. The incoporated amino acids due to exon 11/12 fusion in the Antilopinae is highlighted in blue.

```
delta-ENaC_Homo_sapiens                MAEHRSMDGRMEAATRGGSHLQAA-----------------AQTPPRP---------GPP       34
delta-ENaC_Hippopotamus_amphibius      ----------MEMPTQGGSSLE--AGALAQAAGRGPGTCTCLQLPPPPPP----------       38
delta-ENaC_Tragulus_javanicus          ----------MEAPARGGSSLK--AEGAAQAAGGGSGTCMCPQPPPRPPR----------       38
delta-ENaC_Tragulus_kanchil            ----------MEAPAR-----------------GGSSLKMCPQPPPRPPR----------       23
delta-ENaC_Antilocapra_americana       ----------MEAPAQGGCSLE--AEGTGQTVGGGPGTWTCPQAP---PL----------       35
delta-ENaC_Giraffa_camelopardalis      ----------MEAHAQGGCSFEMQAEGTGQTVGGGPGTWTCPQAP---PP----------       37
delta-ENaC_Giraffa_tippelskirchi       ----------MEAHAQGGCSFE--AEGTGQTVGGGPGTWTCPQAP---PP----------       35
delta-ENaC_Capreolus_pygargus          ----------MDARAQGGCSLEMQTEGTGQTVGGGPGTWTCSQAP---PP----------       37
delta-ENaC_Cervus_elaphus              ----------MDAHAQGGCSLEMQAEGTGQTVGGGPGTWMCSQAP---PP----------       37
delta-ENaC_Moschus_moschiferus         ----------MDAHAQGGCSLEM-----GQTVGGGPGTWTCPQAP---PP----------       32
delta-ENaC_Moschus_berezovskii         ----------MDAHAQGGCSLEM-----GQTVGGGPGTWTCPQAP---PP----------       32
delta-ENaC_Bos_grunniens               ----------MDAHAQGGCSLEMQAEGTGQTVGGGPGTWTCPQAS---PP----------       37
delta-ENaC_Bos_taurus                  ----------MDAHAQGGCSLEMQAEGTGQTVGGGPGTWTCPQAS---PP----------       37
delta-ENaC_Bubalus_bubalis             ----------MDAHAQGGCSLKMQAEGTGQTVGGGPGTWTCPQAS---PP----------       37
delta-ENaC_Nanger_dama                 ----------MDAHPQGGCSLEMQAEGTGQTVGGGPGTWTCPQAS---PP----------       37
delta-ENaC_Kobus_leche                 ----------MDAHAQGGCSLEMQAEGTGQTVGGGPGTWTCPQAS---PP----------       37
delta-ENaC_Capra_hircus                ----------MDADAQGGCSLEMQAEGTGQTVGEGPGTWMCPQAS---PP----------       37
delta-ENaC_Ovis_aries                  ----------MDAHAQGGCSLEMQAEGTGQTVGDGPGTWTCPQAS---PP----------       37
delta-ENaC_Ovis_canadensis             ----------MDAHAQGGCSLEMQAEGTGQTVGDGPGTWTCPQAS---PP----------       37
delta-ENaC_Oreamnos_americanus         ----------MDAHVQGGCSLEMQAEGTGQ-VGEGPGTWMCPQAS---PP----------       36
delta-ENaC_Hippotragus_niger           ----------MDAHAQGGCSLEMQAEGTGQTVGGGAGTWTCPQAS---PP----------       37
delta-ENaC_Damaliscus_lunatus          ----------MDAHAQGGCSLEMQAEGTGQTVGGGPGTWMCPQAS---PP----------       37
delta-ENaC_Sus_scrofa                  ----------MEEPAQGGSSLK--AGASAQAAGGGPGAGRCPQPL---------------       33
delta-ENaC_Vicugna_pacos               ----------MEAPAQGGCRLE--AGARAPEAGGGPGTRKL-------------------       29
delta-ENaC_Equus_callabus              ----------MEASAHRGSCFK--AGTVVQTTSEGLGIWKYPQGQPPLPPLPEDTPPRPE       48
                                                 *:   :

                                                                                    TM1?
delta-ENaC_Homo_sapiens                SAPPPPPKEGHQEGLVELPASFRELLTFFCTNATIHGAIRLVCSRGNRLKTTSWGLLSLG       94
delta-ENaC_Hippopotamus_amphibius      ----PAPEEERRERLVELHTSFREMITFFCTNATIHGTIRLVCSSQNRLKTASWGLLLVG       94
delta-ENaC_Tragulus_javanicus          ----PLPEEEPGERLVEPHASFRELVTFFCTNSTIHGTIRLVCSSQNRLKTASWGLLLAG       94
delta-ENaC_Tragulus_kanchil            ----PLPEEEPGERLVEPHASFRELVTFFCTNSTIHGTIRLVCSSQNRLKTASWGLLLAG       79
delta-ENaC_Antilocapra_americana       ----TLPEEEHGERLVELHASFRELITFFCTNSTIHGTIRLVCSSQNRLKTASWGLLLGG       91
delta-ENaC_Giraffa_camelopardalis      ----MLPKEERGERLVELHASYRELVTFFCTNSTIHGTIRLVCSSQNRLKTASWGLLLAG       93
delta-ENaC_Giraffa_tippelskirchi       ----TLPKEERGERLVELHASYRELVTFFCTNSTIHGTIRLVCSSQNRLKTASWGLLLAG       91
delta-ENaC_Capreolus_pygargus          ----TLPEEERGERLVELHTSFRELVTFFCTNSTIHGTIRLVCSSQNRLKTASWGLLLAG       93
delta-ENaC_Cervus_elaphus              ----TLPEEERGERLIELHASFRELVTFFCTNSTIHGTIRLVCSSQNRLKTASWGLLLAG       93
delta-ENaC_Moschus_moschiferus         ----TLPEEEHGERLVELHTSFRELVTFFCTNSTIHGTIRLVCSSQNHLKTASWGLLLAG       88
delta-ENaC_Moschus_berezovskii         ----TLPEEEHGERLVELHTSFRELVTFFCTNSTIHGTIRLVCSSQNHLKTASWGLLLAG       88
delta-ENaC_Bos_grunniens               ----TLPEEEHGERLVELHASFRELVTFFCTNSTIHGTIRLVCSSQNRLKTASWGLLLAG       93
delta-ENaC_Bos_taurus                  ----TLPEEEHGERLVELHASFRELVTFFCTNSTIHGTIRLVCSSQNRLKTASWGLLLAG       93
delta-ENaC_Bubalus_bubalis             ----TLPEEEHGERLVELHASFRELVTFFCTNSTIHGTIRLVCSSQNRLKTASWGLLLAG       93
delta-ENaC_Nanger_dama                 ----TLPEEEHGERLVELHASFRELVTFFCTNSTIHGTIRLVCSSQNRLKTASWGLLLAG       93
delta-ENaC_Kobus_leche                 ----TLSEEERGERLVELHSSFRELVTFFCTNSTIHGTIRLVCSSQNRLKTASWGLLLAG       93
delta-ENaC_Capra_hircus                ----TLPEEEHGERLVELHTSFRELVTFFCTNSTIHGTIRLVCSSQNRLKTASWGLLLAG       93
delta-ENaC_Ovis_aries                  ----TLPEEEHGERLVELHTSFRELVTFFCTNSTIHGTIRLVCSSQNRLKTASWGLLLAG       93
delta-ENaC_Ovis_canadensis             ----TLPEEEHGERLVELHTSFRELVTFFCTNSTIHGTIRLVCSSQNRLKTASWGLLLAG       93
delta-ENaC_Oreamnos_americanus         ----TLPEEEHGERLVELHTSFRELVTFFCTNSTIHGTIRLVCSSQNRLKTAFWGLLLAG       92
delta-ENaC_Hippotragus_niger           ----TLPEEEHGERLVELHASFRELVTFFCTNSTIHGTIRLVCSSQNRLKTASWGLLLAG       93
delta-ENaC_Damaliscus_lunatus          ----MLPEEEHRERLVELHASFRELVTFFCTNSTIHGTIRLVCSSQNRLKTASWGLLLAG       93
delta-ENaC_Sus_scrofa                  --PLPPSEEERREGLVELHTSFRELVTFFCTNATIHGTIRLVCSSQNRLKTATWALLLAG       91
delta-ENaC_Vicugna_pacos               -------EEECGERLVEFHASFRELVTFFCTNTTIHGSIRLICSSQNRLKTVSWGLLLVG       82
delta-ENaC_Equus_callabus              DRPL-PPEDRPGERLVELHTSFRELFTFFCTNATIHGTIRLVCSRQNRLKTASWGLLLVG       107
                                              ::   * *:*  :*:**:.******:****:***:**  *:***. *.**  *

delta-ENaC_Homo_sapiens                ALVALCWQLGLLFERHWHRPVLMAVSVHSERKLLPLVTLCDGNPRRPSPVLRHLELLDEF       154
delta-ENaC_Hippopotamus_amphibius      SLGMLYWQFGLLFEQYWRYPVSMTVSVRSERKLFPSVTLCDMNPHRPHAARHHLWALDDF       154
delta-ENaC_Tragulus_javanicus          ALGVLYWQFGLLFEQYWRYPVIMTVSVHSERKLLPSVTLCDMNPRWPRSAGRHLRALDDF       154
delta-ENaC_Tragulus_kanchil            ALGVLYWQFGLLFEQYWRYPVIMTVSVHSERKLLPSVTLCDMNPRWPRSAGRHLRALDDF       139
delta-ENaC_Antilocapra_americana       ALGVLYWQFALLLEQYWRYPVIMTVSVHSERKLFPSVTLCDMNPHRPHLARYHLRVLDDF       151
delta-ENaC_Giraffa_camelopardalis      ALGVLYWQFALLFEQYWRYPVIMTVSVHSERKLFPSVTLCDMNPHRPHLARHHLRVLDDF       153
delta-ENaC_Giraffa_tippelskirchi       ALGVLYWQFALLFEQYWRYPVIMTVSVHSERKLFPSVTLCDMNPHRPHLARHHLRVLDDF       151
delta-ENaC_Capreolus_pygargus          ALGVLYWQFALLFEQYWRYPVIMTVSVHSERKLFPSVTLCDMNPHRPHLARHHLRVLDDF       153
delta-ENaC_Cervus_elaphus              ALGVLYWQFALLFEQYWRYPVIMTVSVHSERKLFPSVTLCDMNPHRPHLARHHLRVLDDF       153
delta-ENaC_Moschus_moschiferus         ALGVLYWQFALLFEQYWRYPVIMTVSVHSERKVFPSVTLCDMNPHRPHLARHHLRALDDF       148
delta-ENaC_Moschus_berezovskii         ALGVLYWQFALLFEQYWRYPVIMTVSVHSERKVFPSVTLCDMNPHRPHLARHHLRALDDF       148
delta-ENaC_Bos_grunniens               ALGVLYWQFALLFEQYWRYPVIMTVSVHSERKLFPSVTLCDMNPHRPHLARHHLRVLDDF       153
delta-ENaC_Bos_taurus                  ALGVLYWQFALLFEQYWRYPVIMTVSVHSERKLFPSVTLCDMNPHRPHLARHHLRVLDDF       153
delta-ENaC_Bubalus_bubalis             ALGVLYWQFALLFEQYWRYPVIMTVSVHSERKLFPSVTLCDMNPHRPHLARHHLRVLDDF       153
```

```
delta-ENaC_Nanger_dama                  ALSVLYWQFALLFEQYWRYPVIMTVSVHSERKLFPSVTLCDMNPHRPHLARHHLRVLDDF      153
delta-ENaC_Kobus_leche                  ALGVLYWQFALLFEQYWRYPVIMTVSVHSERKLFPSVTLCDMNPHRPHLARHHLRVLDDF      153
delta-ENaC_Capra_hircus                 ALGVLYWQFALLFEQYWHYPVIMTVSVHSERKLFPSVTLCDMNPHRPHLARHHLRILDDF      153
delta-ENaC_Ovis_aries                   ALGVLYWQFALLFEQYWRYPVIMTVSVHSERKLFPSVTLCDMNPHRPHLARHHLRILDDF      153
delta-ENaC_Ovis_canadensis              ALGVLYWQFALLFEQYWRYPVIMTVSVHSERKLFPSVTLCDMNPHRPHLARHHLRILDDF      153
delta-ENaC_Oreamnos_americanus          ALGVLYWQFALLFEQYWRYPVIMTVSVHSERKIFPSVTLCDMNPHRPHLARHHLRILDDF      152
delta-ENaC_Hippotragus_niger            ALGVLYWQFALLFEQYWRYPVIMTVSVHSERKLFPSVTLCDMNPHRPHLARHHLRVLDDF      153
delta-ENaC_Damaliscus_lunatus           ALGVLYWQFALLFEQYWRYPVIMTVSVHSERKLFPSVTLCDMNPHRPHLARHHLRVLDDF      153
delta-ENaC_Sus_scrofa                   ALSALYWQFGLLFQQYWRYPVVMAVSVHSERKLFPSVTLCDMNPHRPRPARRHLRALDDF      151
delta-ENaC_Vicugna_pacos                TLGMLYWQFGLLFEQYWRYPVIMTVSLHSERKLFPSVTLCDMNPHRPRPARRHLQALDDF      142
delta-ENaC_Equus_callabus               ALGALYWQFGLLFEQYWRYPVIMTVSVHSERKLFPSVTLCDMNPHRPSPARRHLQALDEF      167
                                        :*  * **:.**::::*: ** *:**::****::* ***** **: *  .  **  **:*

delta-ENaC_Homo_sapiens                 ARENIDSLYNVNLSKGRAALSATVPRHEPPFHLDREIRLQRLSHSGSRVRVGFRLCNSTG      214
delta-ENaC_Hippopotamus_amphibius       ARENIYSLYRFNFSESRDAPGAEAPGPEPIFQLDRRVCLQRLSPPGGQNRVGFKLCNSAG      214
delta-ENaC_Tragulus_javanicus           AQENIYSLYGFNFSDSRDVPGAQAPGPEPTFQLDRGIRLQRLSPLDGQNRVGFRLCNSSG      214
delta-ENaC_Tragulus_kanchil             AQENIYSLYGFNFSDSRDVPGAQAPGPEPTFQLDRGIRLQRLSPLDGQNRVGFRLCNSSG      199
delta-ENaC_Antilocapra_americana        AQENIYSLYRFNFSDSRDTLGAEVPGPETAFQLDRRIRLQRLRPLNGQNRVGFKLCNSTG      211
delta-ENaC_Giraffa_camelopardalis       ARENIYSLYRFNFSDSRDALGAEVPGPEPAFQLDRRIRLQRLRPLDGQNRVGFKLCNSTS      213
delta-ENaC_Giraffa_tippelskirchi        ARENIYSLYRFNFSDSRDALGAEVPGPEPAFQLDRRIRLQRLRPLDGQNRVGFKLCNSTS      211
delta-ENaC_Capreolus_pygargus           ARENIYSLYQFNFSDSREALGAEVPGPEPAFQLDRRIRLQRLRPLEGQNRVGFKLCNSTG      213
delta-ENaC_Cervus_elaphus               ARENIYSLYQFNFSDSRDALGAEVPGPEPAFQLDRRIRLQRLRPLDGQNRVGFKLCNSTG      213
delta-ENaC_Moschus_moschiferus          ARESIYSLYRFNFTDSRDALGAEVPSPEPAFQLDRRIRLQRLRPLDGQNRVGFKLCNSTG      208
delta-ENaC_Moschus_berezovskii          ARESIYSLYRFNFSDSRNALGAEVPGPEPVFQLDRRIRLQRLRPLDGQNRVGFKLCNSTG      208
delta-ENaC_Bos_grunniens                ARESIYSLYRFNFSDSMDALGAEVPGPEPAFHLDRRIRLQRLRPLDGQNRVGFKLCNSTG      213
delta-ENaC_Bos_taurus                   ARESIYSLYRFNFSDSMDALGAEVPGPEPAFHLDRRIRLQRLRPLDGQNRVGFKLCNSTG      213
delta-ENaC_Bubalus_bubalis              ARESIYSLYRFNFSNSMDALGAEVPGPEPAFHLDRRIRLQRLRPLHGQNRVGFKLCNSSG      213
delta-ENaC_Nanger_dama                  ARESIYSLYRFNFSDSRDALGAKVPGPEPAFQLDRRIHLQRLRPLDGQNRVGFRLCNSTG      213
delta-ENaC_Kobus_leche                  ARENIYSLYRFNFSDSRDALGAEVPGPEPAFQLDRRIYLQRLRPLDGQNRVGFKLCNSTG      213
delta-ENaC_Capra_hircus                 ARENIYSLYRFNFSDSKDALGAEVPGPEPAFQLDRRIHLQRLRPLDGQNRVGFKLCNSTG      213
delta-ENaC_Ovis_aries                   ARENIYSLYRFNFSDSRDALGAEVPGPEPAFQLDRRIHLQRLRPLDGQNRVGFKLCNSTG      213
delta-ENaC_Ovis_canadensis              ARENIYSLYRFNFSDSRDALGAEVPGPEPAFQLDRRIHLQRLRPLDGQNRVGFKLCNRTG      213
delta-ENaC_Oreamnos_americanus          ARENIYSLYRFNFSDSRDALGAEVLGPEPAFQLDRRIHLQRLRPLDGQNRVGFKLCNSTG      212
delta-ENaC_Hippotragus_niger            ARENIYSLYRFNFRDSRDALGAEAPGPEPAFQLDRSIHLQRLRPLDGQNRVGFKLCNSTG      213
delta-ENaC_Damaliscus_lunatus           ARENIYSLYRFNFSDSRDALGAEVPGPEPAFQLDRRIHLQRLRPLDGRNRVGFKLCNSTG      213
delta-ENaC_Sus_scrofa                   ARENIYSLYGFNFTESEEDPGAEALGSEPAFQLDRGIRLQRLSPLGGQNRVGFRLCNRTG      211
delta-ENaC_Vicugna_pacos                AQENIYSLYGVSVSKTQDPLAAEDLGPEPHFQLDRGVRLQKLSPLGGQNRVGFRLCNSSG      202
delta-ENaC_Equus_callabus               AQENIYSLYKFNFSESWEGPFAEDLGPEPSFQLDRGIRLQRLNHPGRQSKVGFRLCNSTG      227
                                        *:*.* *** ... .      *     *  *:*** : **:*     : :***:*** :.

delta-ENaC_Homo_sapiens                 GDCFYRGYTSGVAAVQDWYHFHYVDILALLPAAWEDSHGSQDGHFVLSCSYDGLDCQARQ      274
delta-ENaC_Hippopotamus_amphibius       SDCIQRAYSSGVAATREWYRFHYVNILASLPATHKDSRHSHGSRFILSCRYDGQDCQARH      274
delta-ENaC_Tragulus_javanicus           GDCVQRAYSSGVLAAREWYRFHYINILALLPATHKD---SPSSRFILSCLYDGQDCYVQH      271
delta-ENaC_Tragulus_kanchil             GDCVQRAYSSGVLAAREWYRFHYINILALLPATHKD---SPSSRFILSCLYDGQDCYVQH      256
delta-ENaC_Antilocapra_americana        GDCVQRAYSSGVVATREWYHFHYINILALLPAAHED---SHSSHFVFSCRYDDQDCHAQH      268
delta-ENaC_Giraffa_camelopardalis       GDCVQQAYSSGVVAAREWYRFHYINILALLPAAHED---SHGSHFVFSCRYDDRDCHAQH      270
delta-ENaC_Giraffa_tippelskirchi        SDCVQQAYSSGVVAAREWYRFHYINILALLPAAHED---SHGSHFVFSCRYDDRDCHAQH      268
delta-ENaC_Capreolus_pygargus           GDCVQRAYSSGMVAAQEWYRFHYINILALLPAAHED---SHSSHFVFSCRYDDRDCHAQH      270
delta-ENaC_Cervus_elaphus               GDCVQQAYSSGIIAAQEWYRFHYINILALLPTAHED---SHGSHFVFSCRYDDRDCHAQH      270
delta-ENaC_Moschus_moschiferus          GDCVQRAYSSGVVAAREWYRFHYMNILALLPAAHEE---SHGSHFVFSCRYNGRDCHAQH      265
delta-ENaC_Moschus_berezovskii          GDCVQRAYSSGVVAAREWYRFHYMNILALLPAAHEE---SHGSHFVFSCRYDGRDCHAQH      265
delta-ENaC_Bos_grunniens                GDCVERAYSSGVVAAREWYRFHYINILALLPAAHED---SHGSHFVFSCRYDDRDCHAQH      270
delta-ENaC_Bos_taurus                   GDCVERAYSSGVVAAREWYRFHYINILALLPAAHED---SHGSHFVFSCRYDDRDCHAQH      270
delta-ENaC_Bubalus_bubalis              GDCVERAYSSGVVAAREWYRFHYINILALLPAAHED---SHGSHFVFSCRYDDRDCHAQH      270
delta-ENaC_Nanger_dama                  GDCVQRAYSSGVVAAREWYRFHYINILALLPADHED---SHDSHFVFSCRYDDQDCHAQH      270
delta-ENaC_Kobus_leche                  GDCVQRAYSSGVVAAREWYRFHYINILALLPAAHED---SHGSHFVLSCRYDDRDCHARH      270
delta-ENaC_Capra_hircus                 GDCVQRAYSSGVVAAREWYRFHYINILALLPAAHED---SHGSHFVFSCRYDDRDCHARH      270
delta-ENaC_Ovis_aries                   GDCVQRAYSSGVVAAREWYRFHYINILALLPAAHED---SHGSHFVFSCRYDDRDCHARH      270
delta-ENaC_Ovis_canadensis              GDCVQRAYSSGVVAAREWYRFHYINILALLPAAHED---SHGSHFVFSCRYDDRDCHARH      270
delta-ENaC_Oreamnos_americanus          GDCVQRAYSSGVVAAREWYRFHYINILALLPAAHED---SHGSHFVFSCRYDDRDCHARH      269
delta-ENaC_Hippotragus_niger            GDCVQRAYSSGVVAAREWYHFHYINILALLPAAHED---SHGSHFVFSCRYDDQDCHARH      270
delta-ENaC_Damaliscus_lunatus           GDCVQRAYSSGVVAAREWYRFHYINILALLPAAHED---SHGSHFVFSCRYDDRDCHARH      270
delta-ENaC_Sus_scrofa                   GDCVHRAYSSGVTAAQEWYRFHYVNILALLPNAPEDSHRSHGGHLVSSCRYDGQDCQAGH      271
delta-ENaC_Vicugna_pacos                GDCIHRAYSSGVTAAREWYRFHYVNILALLPPAHEDHHRSLGSRFIFSCLYDGQDCQARH      262
delta-ENaC_Equus_callabus               GDCFYRVYSSGVTAAREWYRFHYMNILALLPPTREDSHRSHGGHFVFSCRYNGRDCQARH      287
                                        .**. : *:**: *.::**:***::*** **   ::   * ..::: ** *:. ** . :

delta-ENaC_Homo_sapiens                 FRTFHHPTYGSCYTVDGVWTAQRPGITHGVGLVLRVEQQPHLPLLSTLAGIRVMVHGRNH      334
delta-ENaC_Hippopotamus_amphibius       FQTSQHPTYGSCYTFNGVWAAQRPGVTHGISLVLSTERQDHLPLLSTEAGVKVMIHRRGH      334
delta-ENaC_Tragulus_javanicus           LQASHHPTYGSCYTFNGVWAAQRPGITHGISLVLRAEQQEHLPLLSMKASVQLIIHPRGH      331
delta-ENaC_Tragulus_kanchil             LQASHHPTYGSCYTFNGVWAAQRPGITHGISLVLRAEQQEHLPLLSMKASVQLIIHPRGH      316
delta-ENaC_Antilocapra_americana        FQTSHHPTYGSCYTFNGVWAAQRPGVTHRISLVLRAEQQDHLPLLSTRAGIKVMIHPQDH      328
delta-ENaC_Giraffa_camelopardalis       FQTSHHPTYGSCYTFNGVWAAQRPGVTHRISLVLRAEQQDHLPLLSTKAGIKVMIHPQDH      330
delta-ENaC_Giraffa_tippelskirchi        FQTSHHPTYGSCYTFKGVWAAQRPGVTHRISLVLRAEQQDHLPLLSTKAGIKVMIHPQDH      328
delta-ENaC_Capreolus_pygargus           FQTSHHPTYGSCYTFNSVWATQRPGVTHRISLVLRAEQQDHLPLLSTKAGIKVMIHPQDH      330
delta-ENaC_Cervus_elaphus               FQTSHHPTYGSCYTFNSVWATHRPGVTHRISLVLRAEQQDHLPLLSTKAGIKVMIHPQDH      330
delta-ENaC_Moschus_moschiferus          FQMSHHPTYGSCYTFNGVWAAPQPGVTHRISLVLRAEQQDHLPLLSTKAGIKVMIHPQDH      325
delta-ENaC_Moschus_berezovskii          FQTSHHPTYGSCYTFNGVWAAPQPGVTHRISLVLRAEQQDHLPLLSTKAGIKVMIHPQDH      325
delta-ENaC_Bos_grunniens                FQTSHHPTYGSCYTFNGVWAAQRPGVTHRISLVLRAEQLDHLPLLSTKAGIKVMIHPQDH      330
delta-ENaC_Bos_taurus                   FQTSHHPTYGSCYTFNGVWAAQRPGVTHRISLVLRAEQLDHLPLLSTKAGIKVMIHPQDH      330
delta-ENaC_Bubalus_bubalis              FQTSHHPTYGSCYTFNSVWAAQRPGVTHRISLVLRAEQLDHLPLLSTKAGIKVMIHPQDH      330
delta-ENaC_Nanger_dama                  FQTSHHPTYGSCYTFNGVWATQQPGVTHRISLVLKAEQQDHLPLLSTKAGIKVMIHPQDH      330
delta-ENaC_Kobus_leche                  FQTSHHPTYGSCYTFNGVWAAQRPGVTHRISLVLRAEQQDHLPLLSTKAGIKVMIHPQDH      330
delta-ENaC_Capra_hircus                 FQTSHHPTYGSCYTFNGVWAAQRPGVTHRISLVLRAEQQDHLPLLSTKAGIKVMIHPQDH      330
```

```
delta-ENaC_Ovis_aries                    FQTSHHPTYGSCYTFNGVWAAQRPGVTHRISLVLRAEQQDHLPLLSTKAGIKVMIHPQDH      330
delta-ENaC_Ovis_canadensis               FQTSHHPTYGSCYTFNGVWATQRPGVTHRISLVLRAEQQDHLPLLSTKAGIKVMIHPQDH      330
delta-ENaC_Oreamnos_americanus           FQTSHHPTYGSCYTFNGVWAAPRPGVTHRISLVLRAEQQDHLPLLSTKAGIKVMIHPQDH      329
delta-ENaC_Hippotragus_niger             FQTSHHPTYGSCYTFNGVWAAQRPGVTHRISLVLKAEQQDHLPLLSTKAGIKVMIHPQDH      330
delta-ENaC_Damaliscus_lunatus            FQTSHHPTYGSCYTFNGVWAAQRPGVTHRISLVLKAEQQDHLPLLSTKAGIKVMIHPQDH      330
delta-ENaC_Sus_scrofa                    FQTSHHPTYGSCYTFHGIWAAQHPGITHGISLVLRAELQDHLPLLFTEAGIKVLIHRHDQ      331
delta-ENaC_Vicugna_pacos                 FQTSHHPTYGSCYTFDGIWATQCPGITHGISLVLRTEQQDHLPLLSTEAGIKIMIHKRDH      322
delta-ENaC_Equus_callabus                FQTFHHPTYGSCYTFNGAWAAQHPGITHGISLVLRTEQQDHLPLLSTENGIKVMIHRRNQ      347
                                         ::  :*********... *::  **:** :.*** .*   *****    .:::::* :.:

delta-ENaC_Homo_sapiens                  TPFLGHHSFSVRPGTEATISIREDEVHRLGSPYGHCTAGGEGVEVELLHNTSYTRQACLV      394
delta-ENaC_Hippopotamus_amphibius        APFLEHQGFSVRPGTETTIGIREDEVHRLGSPYGHCTDGTG-VDVRLLYNTSYTRQACLA      393
delta-ENaC_Tragulus_javanicus            TPFLEHQGFSVRPGTETTISIREDEVHRLGSPYGQCVDGAGGTDGRPLYNTSYTRQACLV      391
delta-ENaC_Tragulus_kanchil              TPFLEHQGFSVRPGTETTISIREDEVHRLGSPYGQCVDGAGGTDGRPLYNTSYTRQACLV      376
delta-ENaC_Antilocapra_americana         TPFLEHQGFSIRPGTETTIDIREDEVHRLGSPYGQCMDSIGSVDVQLLYNTSYTRQACLV      388
delta-ENaC_Giraffa_camelopardalis        TPFLEHQGFSIRPGTETTIDIREDEVHRLGSPYGQCMDSTGTVDVQLLYNTSYTRQACLV      390
delta-ENaC_Giraffa_tippelskirchi         TPFLEHQGFSIRPGTETTIDIREDEVHRLGSPYGQCMDSTGTVDVQLLYNTSYTRQACLV      388
delta-ENaC_Capreolus_pygargus            TPFLEHQGFSIRPGTETTIDIREDEVHRLGSPYGQCMDSTGSVDVQLLYNTSYTRQACLV      390
delta-ENaC_Cervus_elaphus                TPFLEHQGFSIRPGTETTIDIREDEVHRLGSPYGQCMDSTGSVDVQLLYNTSYTRQACLV      390
delta-ENaC_Moschus_moschiferus           TPFLEHQGFSIRPGTETTIDIREDEVHRLGSPYGQCMDSTGSVDMQLLYNTSYTRQACLV      385
delta-ENaC_Moschus_berezovskii           TPFLEHQGFSIRPGTETTIDIREDEVHRLGSPYGQCMDSTGSVDVQLLYNTSYTRQACLV      385
delta-ENaC_Bos_grunniens                 TPFLEHQGFSIRPGTETTIDIREDEVHRLGSPYGQCMDSTGSVDVQLLYNTSYTRQACLV      390
delta-ENaC_Bos_taurus                    TPFLEHQGFSIRPGTETTIDIREDEVHRLGSPYGQCMDSTGSVDVQLLYNTSYTRQACLV      390
delta-ENaC_Bubalus_bubalis               TPFLEHQGFSIRPGTETTIDIREDEVHRLGSPYGQCMVSTGSVDVQLLYNTSYTRQACLV      390
delta-ENaC_Nanger_dama                   TPFLEHQGFSIRPGTETTIDIREDEVHRLGSPYGQCMDSTGSVDVQLLYNTSYTRQACLA      390
delta-ENaC_Kobus_leche                   TPFLEHQGFSIRPGTETTIDIREDEVHRLGSPYGQCMDSTGSVDVQLLYNTSYTRQACLA      390
delta-ENaC_Capra_hircus                  TPFLEHQGFSIRPGTETTIDIREDEVHRLGSPYGQCMDSTGSMDVQLLYNTSYTRQACLA      390
delta-ENaC_Ovis_aries                    TPFLEHQGFSIRPGTETTIDIREDEVHRLGSPYGQCMDSTGNMDVQLLYNTSYTRQACLA      390
delta-ENaC_Ovis_canadensis               TPFLEHQGFSIRPGTETTIDIREDEVHRLGSPYGQCMDSTGSMDVQLLYNTSYTRQACLA      390
delta-ENaC_Oreamnos_americanus           TPFLEHQGFSIRPGTETTIDIREDEVHRLGSPHGQCMDSTGSMDVQLLYNTSYTRQACLA      389
delta-ENaC_Hippotragus_niger             TPFLEHQGFSIRPGTETTIDIREDEVHRLGSPYGQCMDSTGSMDVQLLYNTSYTRQACLA      390
delta-ENaC_Damaliscus_lunatus            TPFLEHQGFSIRPGTETTIDIREDEVHRLGSPYGQCMDSTSSMDVQLLYNTSYTRQACLA      390
delta-ENaC_Sus_scrofa                    TPFLEHQGFSIRPGTETTIGIREDEVHRLGSPYGHCTHSAGSVDVRLLYKASYTKQACLV      391
delta-ENaC_Vicugna_pacos                 TPFLEHRGFSVRPGTETTIGIREDEVHWLGSPYGQCTDSTEGVDVPLLYNASYTMQACLV      382
delta-ENaC_Equus_callabus                TPFLEHRGFSIRPGTETTIGIREDEVHRLGSPYGHCSDGMEAVDVPLLYNATYTMQACLV      407
                                         :*** *:.**:*****:**.******* ****:*:*  .    :   *::::** ****.

delta-ENaC_Homo_sapiens                  SCFQQLMVETCSCGYYLHPLPAGAEYCSSARHPAWGHCFYRLYQDLETHRLPCTSRCPRP      454
delta-ENaC_Hippopotamus_amphibius        SCFQQLMVETCSCGYFFYPLPAGAQYCSYTRHPAWGHCFHRLYKDLQTHRLPCASRCPQL      453
delta-ENaC_Tragulus_javanicus            SCFQQLMVETCSCGYFFYPLPAGAVYCSYGRHPAWGHCFHRLYQKLEAQQLPCATRCPRP      451
delta-ENaC_Tragulus_kanchil              SCFQQLMVETCSCGYFFYPLPAGAVYCSYGRHPAWGHCFHRLYQKLEAQQLPCATRCPRP      436
delta-ENaC_Antilocapra_americana         SCFQQLMVETCSCGYFFYPLPAEAEYCSYTRHPAWGHCFHHLYQKLKTHQLSCTTRCPRP      448
delta-ENaC_Giraffa_camelopardalis        SCFQQLMVETCSCGYFFYPLPAGAEYCSYMRHPAWGHCFHRLYQKLKTHQLPCATRCPRP      450
delta-ENaC_Giraffa_tippelskirchi         SCFQQLMVETCSCGYFFYPLPAGAEYCSYMRHPAWGHCFHRLYQKLKTHQLPCATRCPRP      448
delta-ENaC_Capreolus_pygargus            SCFQQLMVQTCSCGYFFYPLPAGAEYCSYMRHPAWGHCFHHLYQKLKTHQLPCTTRCPQP      450
delta-ENaC_Cervus_elaphus                SCFQQLMVQTCSCGYFFYPLPAGAEYCSYMRHPAWGHCFHHLYQKLKTHQLPCTTRCPQP      450
delta-ENaC_Moschus_moschiferus           SCFQQLMVETCSCGYFFYPLPAGAEYCSYMRHPAWGHCFHRLYQKLKTHQLPCTTRCPRP      445
delta-ENaC_Moschus_berezovskii           SCFQQLMVETCSCGYFFYPLPAGAEYCSYMRHPAWGHCFHRLYQKLKTHQLPCTTRCPRP      445
delta-ENaC_Bos_grunniens                 SCFQHLMVETCSCGYFFYPLPAGAEYCSYMRHPAWGHCFHHLYQKLKTHQLPCTTRCPRP      450
delta-ENaC_Bos_taurus                    SCFQHLMVETCSCGYFFYPLPAGAEYCSYMRHPAWGHCFHHLYQKLKTHQLPCTTRCPRP      450
delta-ENaC_Bubalus_bubalis               SCFQHLMVETCSCGYFFYPLPAGAEYCSYMRHPAWGHCFHHLYQKLKTHQLPCTTRCPRP      450
delta-ENaC_Nanger_dama                   SCFQHLMVETCSCGYFFYPLPAGAEYCSYMRHPAWGHCFHRLYQKLKTHQLPCTTRCPRP      450
delta-ENaC_Kobus_leche                   SCFQHLMVETCSCGYFFYPLPAGAEYCSYMRHPAWGHCFHRLYQKLKTHQLPCNTRCPRP      450
delta-ENaC_Capra_hircus                  SCFQHLMVETCSCGYFFYPLPVGAEYCSYMRHPAWGHCFHRLYQKLKTHQLPCTTRCPRP      450
delta-ENaC_Ovis_aries                    SCFQHLMVETCSCGYFFYPLPVGAEYCSYMRHPAWGHCFHRLYQKLKTHQLPCTTRCPRP      450
delta-ENaC_Ovis_canadensis               SCFQHLMVETCSCGYFFYPLPVGAEYCSYMRHPAWGHCFHRLYQKLKTHQLPCTTRCPRP      450
delta-ENaC_Oreamnos_americanus           SCFQHLMVETCSCGYFFYPLPVGAEYCSYMRHPAWGHCFHHLYQKLKTHQLPCTTRCPRP      449
delta-ENaC_Hippotragus_niger             SCFQHLMVETCSCGYFFYPLPAGAEYCSYMRHPAWGHCFHRLYQKLKTHQLPCTTRCPRP      450
delta-ENaC_Damaliscus_lunatus            SCFQHLMVETCSCGYFFYPLPVGAEYCSYMRHPAWGHCFHRLYQKLKTHQLPCTIRCPRP      450
delta-ENaC_Sus_scrofa                    SCFQQLMVDTCSCGYYLYPLPAGAEYCSYTRHPAWGHCFYRLYQDLETHRLPCTSRCPRP      451
delta-ENaC_Vicugna_pacos                 SCFQQLMVQTCSCGYYFYPLPAGAEYCSYVRHPAWGHCFYRLYRDLGTHRLPCASRCPRP      442
delta-ENaC_Equus_callabus                SCFQQLMVETCSCGYYLYPLPVGAEYCSYTRHPAWGHCFYRLYRDLETHRLPCASRCPRP      467
                                         ****:***:******:::***. * ***  *********:**:.* :::* *  ***:

delta-ENaC_Homo_sapiens                  CRESAFKLSTGTSRWPSAKSAGWTLATLGEQGLPHQSHR--------------------      493
delta-ENaC_Hippopotamus_amphibius        CRESSYKLSAGTSRWPSSKSAGWILALLDTPGHRSPRPGRAQL-----------------      496
delta-ENaC_Tragulus_javanicus            CRESSYKLSAGTSRWPSSKSADWVLAVLGEPGPRSPSPS------WVQP-----------      494
delta-ENaC_Tragulus_kanchil              CRESSYKLSAGTSRWPSSKSADWVLAVLGEPGPRSPSPS------WVQP-----------      479
delta-ENaC_Antilocapra_americana         CRESSYKLSAGTSRWPSSTSSDWVLAVLGEPSRRNPWPPSASI-----------------      491
delta-ENaC_Giraffa_camelopardalis        CRESSYKLSAGTSRWPSSTSADWVLAVLGEPSGRNPWLPSASV-----------------      493
delta-ENaC_Giraffa_tippelskirchi         CRESSYKLSAGTSRWPSSTSADWVLAVLGEPSGRNPWPPSASV-----------------      491
delta-ENaC_Capreolus_pygargus            CRESSYKLSAGTSRWPSSTSADWVLAVLGEPSRRNPWPPSASI-----------------      493
delta-ENaC_Cervus_elaphus                CRESSYRLSAGTSRWPSSTSADWVLAVLGETSRRNPWPPSASI-----------------      493
delta-ENaC_Moschus_moschiferus           CRESSYKLSAGTSRWPSSTSADWVLAVLGEPSRRNPWVPSASV-----------------      488
delta-ENaC_Moschus_berezovskii           CRESSYKLSAGTSRWPSSTSADWVLAVLGEPSRRNPWVPSASV-----------------      488
delta-ENaC_Bos_grunniens                 CRESSYKLSAGTSRWPSSTSADWVLAVLGEPSRRNPWPSSASI-----------------      493
delta-ENaC_Bos_taurus                    CRESSYKLSAGTSRWPSSTSADWVLAVLGEPSRRNPWPSSASI-----------------      493
delta-ENaC_Bubalus_bubalis               CRESSYKLSAGTSRWPSSTSADWVLAVLGEPSRRNPWPPSASI-----------------      493
delta-ENaC_Nanger_dama                   CRESSYKLSAGTSRWPSSTSADWVLAVLSEPSRRNPWPPSASIKSWPLPLPSPSRACTEG      510
delta-ENaC_Kobus_leche                   CRESSYKLSAGTSRWPSSTSADWVLAVLGEPSRRNPWPPSASIKSWPLPLPSPSRACTEG      510
delta-ENaC_Capra_hircus                  CRESSYKLSAGTSRWPSSTSADWVLAVLGEPSRWNPWPPSASIKSWPLSLPSPSRACTEG      510
delta-ENaC_Ovis_aries                    CRESSYKLSAGTSRWPSSTSADWVLAVLGEPSRWNPWPPSASIKSWPLSLPSPSRACTEG      510
delta-ENaC_Ovis_canadensis               CRESSYKLSAGTSRWPSSTSADWVLAVLGEPSRWNPWPPSASIKSWPLSLPSPSRACTEG      510
delta-ENaC_Oreamnos_americanus           CRESSYKLSAGTSRWPSSTSADWVLAVLGEPSRWNPWPPSASIKSWPLSLPSPSRACTEG      509
```

```
delta-ENaC_Hippotragus_niger        CRESSYKLSAGTSRWPSSTSADWVLAVLGEPSRRNPWPPSASIKSWPLPLPSPSRACTEG    510
delta-ENaC_Damaliscus_lunatus       CRESSYKLSAGTSRWPSSTSADWVLAVLGEPSRRNPWPPSASIKSWPLPLPSPSRACTEG    510
delta-ENaC_Sus_scrofa               CRESTYKLTAGVSRWPSSKSADWIPAVLDRPGGRQ-SPS---------------------    489
delta-ENaC_Vicugna_pacos            CRESSFRLSAGTSRWPSSKSADWILSVLGKPGH-R-SPS---------------------    479
delta-ENaC_Equus_callabus           CRESSYKLSAGTSRWPSSKSADWILAVLGKRGHQKPSQS---------------------    506
                                    ****::*::*.*****:.*:.*  : *.   .

                                                                                  TM2?
delta-ENaC_Homo_sapiens             ---------------QRSSLAKINIVYQELNYRSVEEAPVYSVPQLLSAMGSLCSLWFGA    538
delta-ENaC_Hippopotamus_amphibius   -----------PPAKATGTLAKVNIFYQELNYRTVDETPVYSAPQLLSAMGSLWSLWFGS    545
delta-ENaC_Tragulus_javanicus       -----------PSAAVPSSLAKVNIFYQELNYRAVNETPVYSVPQLLSAMGSLWSLWFGS    543
delta-ENaC_Tragulus_kanchil         -----------PSAAVPSSLAKVNIFYQELNYRAVNETPVYSVPQLLSAMGSLWSLWFGS    528
delta-ENaC_Antilocapra_americana    ----------------KISLAKVNIFYQELNYRTVDETPVYSVPQLLSAMGSLWSLWFGS    535
delta-ENaC_Giraffa_camelopardalis   ----------------KISLAKVNIFYQELNYRTVDETPVYSVPQLLSAMGSLWSLWFGS    537
delta-ENaC_Giraffa_tippelskirchi    ----------------KISLAKVNIFYQELNYRTVDETPVYSVPQLLSAMGSLWSLWFGS    535
delta-ENaC_Capreolus_pygargus       ----------------KISLAKVNIFYQELNYRMVNETPVYSVSQLLSAMGSLWSLWLGS    537
delta-ENaC_Cervus_elaphus           ----------------KISLAKVNIFYQELNYRMVNETPVYSVSQLLSAMGSLWSLWLGS    537
delta-ENaC_Moschus_moschiferus      ----------------KISLAKVNIFYQELNYRTVDEMPVYSVPQLLSAMGSLWSLWFGS    532
delta-ENaC_Moschus_berezovskii      ----------------KISLAKVNIFYQELNYRTVDEMPVYSVPQLLSAMGSLWSLWFGS    532
delta-ENaC_Bos_grunniens            ----------------NISLAKVNIFYQELNYRTVDETPVYSVPQLLSAMGSLWSLWFGS    537
delta-ENaC_Bos_taurus               ----------------NISLAKVNIFYQELNYRTVDETPVYSVPQLLSAMGSLWSLWFGS    537
delta-ENaC_Bubalus_bubalis          ----------------NISLAKVNIFYQELNYRTVDETPVYSVPQLLSAMGSLWSLWFGS    537
delta-ENaC_Nanger_dama              LTSRSGAQPLSPAPCPRISLAKVNIFYQELNYRTVDETPVYSVPQLLSAMGSLWSLWFGS    570
delta-ENaC_Kobus_leche              PASRSRAQTLSPAPCPRISLAKVNIFYQELNYRTVDETPVYSVPQLLSAMGSLWSLWFGS    570
delta-ENaC_Capra_hircus             PTGRSGAQPLSPAPCPRISLAKVNIFYQELNYRTVDETPVYSVPQLLSAMGSLWSLWFGS    570
delta-ENaC_Ovis_aries               PTGRSGAQPLSPAPCPRISLAKVNIFYQELNYRTVDETPVYSVPQLLSAMGSLWSLWFGS    570
delta-ENaC_Ovis_canadensis          PTGRSGAQPLSPAPCPRISLAKVNIFYQELNYRTVDETPVYSVPQLLSAMGSLWSLWFGS    570
delta-ENaC_Oreamnos_americanus      PTGRSGAQPLSPAPCPRISLAKLNIFYQELNYRTVDETPVYSVPQLLSAMGSLWSLWFGS    569
delta-ENaC_Hippotragus_niger        PTSRSGAQPLSPAPCPRISLAKVNIFYQELNYRTVDETPVYSVPQLLSAMGSLWSLWFGS    570
delta-ENaC_Damaliscus_lunatus       PTNRSGAQPLSPAPCPRISLAKVNIFYQELNYRTVDETPVYSVPQLLSAMGSLWSLWFGS    570
delta-ENaC_Sus_scrofa               ---------------PRSRLAKVNIFYRELNYRTVDEMPVYSVPQLLSAMGSLWSLWFGS    534
delta-ENaC_Vicugna_pacos            ---------------PRSNLAKVNILYQELNYRTVDETPIYSVPRLLSAMGSLWSLWFGS    524
delta-ENaC_Equus_callabus           ---------------PRSNIAKMNIFYQELNYRTVDETPVYSVPQLLSAMGSLWSLWFGS    551
                                                       :**:**.*:***** *:* *:**. :******** ***:*:

delta-ENaC_Homo_sapiens             SVLSLLELLELLLDASALTLVLGGRRLRRAWFSWPRASPASGASSIKPEASQMPPPAGGT    598
delta-ENaC_Hippopotamus_amphibius   SVLSVVEVLELLLDAAALALLLCCRRLRGA-RGQPGTATGAPAPSQRP--AGCPAAAGMT    602
delta-ENaC_Tragulus_javanicus       SVLSVVEVLELLLDATALALLLGHHWLCGA-GGRPRAAPGVPAPSQRL--AGGPVAASPR    600
delta-ENaC_Tragulus_kanchil         SVLSVVEVLELLLDATALALLLGHHWLCGA-GGRPRAAPGVPAPSQRL--AGGPVAASPR    585
delta-ENaC_Antilocapra_americana    SVLSVVEVLELLLDAMALTLLLCCRWLRGS-RGHPRVATRVPSSSQRP--ASGPVAARTM    592
delta-ENaC_Giraffa_camelopardalis   SVLSVVEVLELLLDAMALTLLLCCRWLRGS-RRQPGAATRVPSPSQRP--ASGPVAAGTT    594
delta-ENaC_Giraffa_tippelskirchi    SVLSVVEVLELLLDAMALTLLLCCRWLRGS-RRQPGAATRVPSPSQRP--ASGPVAAGTT    592
delta-ENaC_Capreolus_pygargus       SVLSVVEVLELLLDAMALTLLLCCRRLRGS-QGQPRAAARVPPPSQRP--ASGPVAAGTT    594
delta-ENaC_Cervus_elaphus           SVLSVVEVLELLLDAMALTLLLCCRWLRRS-QGQPRAAMRVPPPSQRP--ASGPVAAGTT    594
delta-ENaC_Moschus_moschiferus      SVLSVVEVLELLLDAMALTLLLCCRWLRGS-QRQPRAATRVPPPSQRP--ASGPVAAGTT    589
delta-ENaC_Moschus_berezovskii      SVLSVVEVLELLLDAMALTLLLCCRWLRGS-QRQPRAATRVPPPSQRP--ASGPVAAGTT    589
delta-ENaC_Bos_grunniens            SVLSVVEVLELLLDAIALTLLLCCRWLCGS-RGQPRAATRVHPPSQRP--ASGPVAADTT    594
delta-ENaC_Bos_taurus               SVLSVVEVLELLLDAIALTLLLCCRWLCGS-RGQPRAATRVHPPSQRP--ASGPVAADTT    594
delta-ENaC_Bubalus_bubalis          SVLSVVEVLELLLDAMALTLLLCCRWLRGS-RGQPRAATRVHPPSQRP--ASGPVAAGTT    594
delta-ENaC_Nanger_dama              SVLSVVEVLELLLDAMALTLLLCCRWLRGS-WGQPRAATSVPPPSQRP--ASGPVPAGTT    627
delta-ENaC_Kobus_leche              SVLSVVEVLELLLDAMALTLLLCCRWLRGS-RGQPRAATRVPPPSQRP--ASGPVAAGTT    627
delta-ENaC_Capra_hircus             SVLSVVEVLELLLDTMALALLLCCRWLRGS-QGQPRAATRVPPPSQRP--ASRPVAAGT-    626
delta-ENaC_Ovis_aries               SVLSVVEVLELLLDTMALALLLCCRWLRGS-QGQPRAATRVPPPSQRP--ASGPVAAGTT    627
delta-ENaC_Ovis_canadensis          SVLSVVEVLELLLDTMALALLLCFRWLRGS-QGQPRAATRVPPPSQRP--ASGPVAAGTT    627
delta-ENaC_Oreamnos_americanus      SVLSVVEVLELLLDTMALALLLCCRWLRRS-QGQPRVATRVPPPSQRP--ASGPVATGTT    626
delta-ENaC_Hippotragus_niger        SVLSVVEVLELLLDAMALALLLCCRWLRGS-RGQPRAATRVPPPSQRP--ASGPVAVGTT    627
delta-ENaC_Damaliscus_lunatus       SVLSVVEVLELLLDAMALALLLCCRWLRGS-RGQPRAATRVPPPSQRP--ASGPVAAGTT    627
delta-ENaC_Sus_scrofa               SVLSVLEVLELLLDAMVLTLLLCCRQLHRA-RGQLGAAPGEPTPSQRP--TCCPAAAGMT    591
delta-ENaC_Vicugna_pacos            SVLSVLEVLELLLDATALTFLLCCRRLHAA-RGQPRAATRAPTPSQRP--ASCPVAGGTS    581
delta-ENaC_Equus_callabus           SVLSVLELLELLLDATALALLLCCRRLRRAWRSQPGAATRASGPRPEA--TPLPSDCGPD    609
                                    ****::*:******: .*:::*  : *  :      .:         .   :  *

delta-ENaC_Homo_sapiens             SDDPEPSGPHLPRVMLPGVL--A-GVSAE------------   624
delta-ENaC_Hippopotamus_amphibius   SHAPGPSWLHLRRGCQGSSRSLG------------------   625
delta-ENaC_Tragulus_javanicus       SHAPGPAAFASHNAAGAPAGVLAAELSQVWAPETPNT----   637
delta-ENaC_Tragulus_kanchil         SHAPGPAAFASHNAAGAPAGVLAAELSQVWAPETPNT----   622
delta-ENaC_Antilocapra_americana    SNTPGPGCLHLPRCCWGFSR----SLS--------------   615
delta-ENaC_Giraffa_camelopardalis   SNTPGPGCLHLPGLQQESRL----RNSAKCGPQRPLTPEPV   631
delta-ENaC_Giraffa_tippelskirchi    SNTPGPGCLHLPGLQQESRL----RNSAKCGPQRPLTPEPV   629
delta-ENaC_Capreolus_pygargus       SNAPGPGCLRLPRCCRDFSR----SLG--------------   617
delta-ENaC_Cervus_elaphus           SNAPGPGCLRLPRCCRDFSR----SLG--------------   617
delta-ENaC_Moschus_moschiferus      SNAPGPGCLHLPRCCRDFSR----SLG--------------   612
delta-ENaC_Moschus_berezovskii      SNAPGPGCLHLPRCCRDFSR----SLG--------------   612
delta-ENaC_Bos_grunniens            SNAPGPGCLHLPRCCRDFSR----SLG--------------   617
delta-ENaC_Bos_taurus               SNAPGPGCLHLPRCCRDFSR----SLG--------------   617
delta-ENaC_Bubalus_bubalis          SNAPGPGCLHLPRCCRDFGR----SLD--------------   617
delta-ENaC_Nanger_dama              SNAPGPSCLHLPRCCRDFSR----SLG--------------   650
delta-ENaC_Kobus_leche              SNALGPSCLHLPQCCQDFSR----SLG--------------   650
delta-ENaC_Capra_hircus             SNALGPSCLHLPRCCRDFSR----SLS--------------   649
delta-ENaC_Ovis_aries               SNALGPSCLHLPRCCRDFSR----SLG--------------   650
delta-ENaC_Ovis_canadensis          SNALGPSCLHLPRCCRDFSR----SLG--------------   650
delta-ENaC_Oreamnos_americanus      SNALGPSCLHLPRCCRDFSR----SLG--------------   649
delta-ENaC_Hippotragus_niger        SNAPGPGCLHLPRCCRDFSR----SLS--------------   650
delta-ENaC_Damaliscus_lunatus       SNAPGPGCLHLPRCCRDFSR----SLG--------------   650
```

```
delta-ENaC_Sus_scrofa               SNARGPSWPRFP-----------------------------  603
delta-ENaC_Vicugna_pacos            -DVQGPG----------------------------------  587
delta-ENaC_Equus_callabus           --LKCSGTPAGASAEESRL----------------------  626
                                          .
```

Supplementary Figure 1

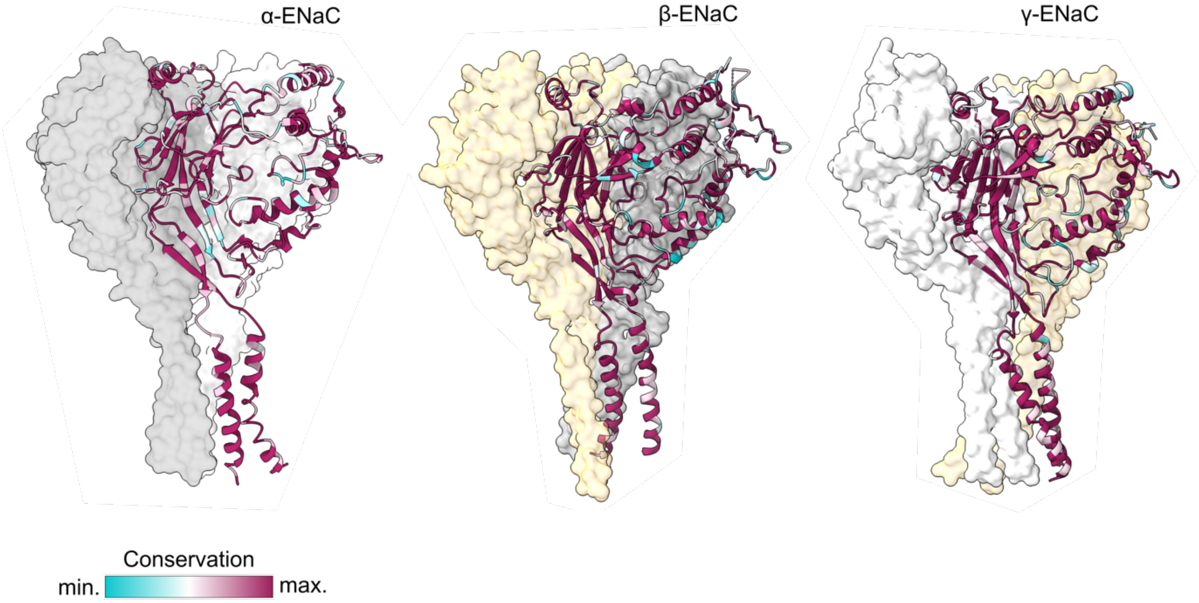

Conservation of ENaC subunit sequences. The Cryo-EM derived structure of human ENaC (PDB: 6BQN)[1] is displayed and each ENaC subunit is coloured by the conservation in the multiple sequence alignments provided in Supplemental Data 2. Molecular graphics and analyses were performed with UCSF ChimeraX [3], developed by the Resource for Biocomputing, Visualization, and Informatics at the University of California, San Francisco, with support from National Institutes of Health R01-GM129325 and the Office of Cyber Infrastructure and Computational Biology, National Institute of Allergy and Infectious Diseases.

Supplementary References

[1] Noreng S, Bharadwaj A, Posert R, Yoshioka C, Baconguis I. 2018. Structure of the human epithelial sodium channel by cryo-electron microscopy. *Elife* 7:e39340. https://doi.org/10.7554/eLife.39340

[2] Noreng S, Posert R, Bharadwaj A, Houser A, Baconguis I. 2020. Molecular principles of assembly, activation, and inhibition in epithelial sodium channel.Aldrich RW, Subramaniam S, Palmer L, Cao E, editors. *Elife* 9:e59038. https://doi.org/10.7554/eLife.59038

[3] Meng EC, Goddard TD, Pettersen EF, Couch GS, Pearson ZJ, Morris JH, Ferrin TE. 2023. UCSF ChimeraX: Tools for structure building and analysis. *Protein Sci* 32.
