# Supplementary material for: The evolutionary path of the epithelial sodium channel δ-subunit in Cetartiodactyla points to a role in sodium sensing
Source: Commun Biol. 2025 Jul 4;8:1004. doi: 10.1038/s42003-025-08436-7 (PMC12227717; doi:10.1038/s42003-025-08436-7)
Supplement: Supplementary file 5 — Description of Additional Supplementary Files [file 42003_2025_8436_MOESM5_ESM.pdf]

Description of Additional Supplementary Files

File name: Supplementary Data 1

Description: The excel file provides the exon and intron sizes (in bases) of the ENaC genes of all analysed Cetartiodactyla and provides source data behind Figures 1 and 2. Exon sizes are provided in comparison with the corresponding exon sequence of the Alpaca (Vicugna pacos) and insertions/deletions with respect to these references were mapped.

File name: Supplementary Data 2

Description: Datafile containing cetacean feeding latencies in response to salt-containing or salt-free gelatin blocks and source data behind Figure 5.

File name: Supplementary Data 3

Description: The excel file provides the exon and intron sizes (in bases) of the ENaC genes of all analysed Afrotheria, Xenarthra and Carnivora and provides source data behind Figure 6. Exon sizes are provided in comparison with the corresponding exon sequence of the Alpaca (Vicugna pacos) and insertions/deletions with respect to these references were mapped.
