# Supplementary material for: The evolutionary path of the epithelial sodium channel δ-subunit in Cetartiodactyla points to a role in sodium sensing
Source: Commun Biol. 2025 Jul 4;8:1004. doi: 10.1038/s42003-025-08436-7 (PMC12227717; doi:10.1038/s42003-025-08436-7)
Supplement: Supplementary file 6 — Reporting Summary [file 42003_2025_8436_MOESM6_ESM.pdf]

Corresponding author(s): Mike Althaus

Last updated by author(s): Jun 8, 2025

Reporting Summary

Nature Portfolio wishes to improve the reproducibility of the work that we publish. This form provides structure for consistency and transparency in reporting. For further information on Nature Portfolio policies, see our Editorial Policies and the Editorial Policy Checklist.

Statistics

For all statistical analyses, confirm that the following items are present in the figure legend, table legend, main text, or Methods section.

n/a	Confirmed	
☐	☒	The exact sample size (*n*) for each experimental group/condition, given as a discrete number and unit of measurement
☐	☒	A statement on whether measurements were taken from distinct samples or whether the same sample was measured repeatedly
☐	☒	The statistical test(s) used AND whether they are one- or two-sided<br>*Only common tests should be described solely by name; describe more complex techniques in the Methods section.*
☐	☒	A description of all covariates tested
☐	☒	A description of any assumptions or corrections, such as tests of normality and adjustment for multiple comparisons
☐	☒	A full description of the statistical parameters including central tendency (e.g. means) or other basic estimates (e.g. regression coefficient) AND variation (e.g. standard deviation) or associated estimates of uncertainty (e.g. confidence intervals)
☐	☒	For null hypothesis testing, the test statistic (e.g. *F*, *t*, *r*) with confidence intervals, effect sizes, degrees of freedom and *P* value noted<br>*Give P values as exact values whenever suitable.*
☐	☒	For Bayesian analysis, information on the choice of priors and Markov chain Monte Carlo settings
☐	☒	For hierarchical and complex designs, identification of the appropriate level for tests and full reporting of outcomes
☐	☒	Estimates of effect sizes (e.g. Cohen's *d*, Pearson's *r*), indicating how they were calculated

Our web collection on statistics for biologists contains articles on many of the points above.

Software and code

Policy information about availability of computer code

Data collection	ColabFold v1.5.5 was employed to predict structures of sheep delta-ENaC (https://colab.research.google.com/github/sokrypton/ColabFold/blob/main/Colabfold.ipynb). Conservation of ENaC sequences was visualised using UCSF ChimeraX v.1.7.1.
Data analysis	Bayesian mixed effects models to analyse cetacean latencies and to test whether pseudogenisation of the SCNN1D is related to the return of saltwater habitats in mammals were run in R statistical software version 4.3.1 interfaced through RStudio, using the software package "MCMCglmm" version 2.35. Additional statistical analysis was done using GraphPad Prism (v. 10) (GraphPad Software, San Diego, USA). HyPhy and CodeML of the PAML software package (v.4) were used to analyse selection pressure on SCNN1 genes.

For manuscripts utilizing custom algorithms or software that are central to the research but not yet described in published literature, software must be made available to editors and reviewers. We strongly encourage code deposition in a community repository (e.g. GitHub). See the Nature Portfolio guidelines for submitting code & software for further information.

Data

Policy information about availability of data

All manuscripts must include a data availability statement. This statement should provide the following information, where applicable:

- Accession codes, unique identifiers, or web links for publicly available datasets
- A description of any restrictions on data availability
- For clinical datasets or third party data, please ensure that the statement adheres to our policy

Data are made available in the manuscript, as Supplemental Data or as Source Data deposited at the Zenodo data depository, doi: 10.5281/zenodo.15255678

Research involving human participants, their data, or biological material

Policy information about studies with human participants or human data. See also policy information about sex, gender (identity/presentation), and sexual orientation and race, ethnicity and racism.

Reporting on sex and gender	not applicable
Reporting on race, ethnicity, or other socially relevant groupings	not applicable
Population characteristics	not applicable
Recruitment	not applicable
Ethics oversight	not applicable

Note that full information on the approval of the study protocol must also be provided in the manuscript.

Field-specific reporting

Please select the one below that is the best fit for your research. If you are not sure, read the appropriate sections before making your selection.

☒ Life sciences ☐ Behavioural & social sciences ☐ Ecological, evolutionary & environmental sciences

For a reference copy of the document with all sections, see nature.com/documents/nr-reporting-summary-flat.pdf

Life sciences study design

All studies must disclose on these points even when the disclosure is negative.

Sample size	PCR data: RNA was isolated from tissue samples of 3 sheep and 3 Bottlenose dolphins.<br>Animal behaviour data: Sample sizes were not pre-determined statistically. The Bayesian mixed effects model revealed significant differences in latency times, indicating that sample sizes were appropriate.
Data exclusions	Animal behaviour data: A latency measurement was excluded from the analysis when the animal trainer did not remain in neutral position until the animal displayed begging behaviour.
Replication	Genomic data: All data for a given species, avaiable in the NCBI databank at the time of the investigation, were analysed. Results were confirmed using transcriptomic data avialbale in the NCBI sequence read archive.<br>PCR data: Positive amplicons out of the total number of animals are provided in the figures.<br>Animal behaviour data: Feeding experiment was repeated over two weeks providing 11 or 12 sets of latency measurements (4 feeding stimuli per set) to ensure adequate sample size given logistical constraints. Model structure accounted for potential differences attributed to feeding day, trainer, and individual animal.
Randomization	Animal behaviour data: Stimulus was randomised based on 4 protocols of random orders of 2 with and 2 without salt gelatin blocks fed to cetaceans.
Blinding	Animal behaviour data: The trainers that provided the gelatin blocks to the animals were blinded and did not know the order of stimuli.

Reporting for specific materials, systems and methods

We require information from authors about some types of materials, experimental systems and methods used in many studies. Here, indicate whether each material, system or method listed is relevant to your study. If you are not sure if a list item applies to your research, read the appropriate section before selecting a response.

Materials & experimental systems

n/a	Involved in the study
☒	☐ Antibodies
☒	☐ Eukaryotic cell lines
☒	☐ Palaeontology and archaeology
☐	☒ Animals and other organisms
☒	☐ Clinical data
☒	☐ Dual use research of concern
☒	☐ Plants

Methods

n/a	Involved in the study
☒	☐ ChIP-seq
☒	☐ Flow cytometry
☒	☐ MRI-based neuroimaging

Animals and other research organisms

Policy information about studies involving animals; ARRIVE guidelines recommended for reporting animal research, and Sex and Gender in Research

Laboratory animals	The study did not involve laboratory animals. It involved cetaceans (Bottlenose dolphins and Beluga whales) under human care at the Oceanogràfic, Ciudad de las Artes y las Ciencias, Valencia, Spain.
Wild animals	The study did not involve wild animals. It involved cetaceans (Bottlenose dolphins and Beluga whales) under human care at the Oceanogràfic, Ciudad de las Artes y las Ciencias, Valencia, Spain.
Reporting on sex	Animal behaviour data: For Bottlenose dolphins, sex has been included in the Bayesian mixed effects model . The Beluga whale data was modelled in a similar fashion, but as there were only two animals, one of each sex, differences due to sex-based or individual differences are confounded with no replication. Sexes are also indicated in Figure 5.
Field-collected samples	The study did not involve samples collected from the field.
Ethics oversight	Experiments were approved by the Oceanogràfic Animal Care & Welfare Committee, a legally approved body by the Regional Government of Valencia to evaluate and approve research activity with animals, under the project reference OCE-11-23 as well as the Generalitat Valenciana under the project reference 2025-VSC-PEA-0040.

Note that full information on the approval of the study protocol must also be provided in the manuscript.

Plants

Seed stocks	not applicable
Novel plant genotypes	not applicable
Authentication	not applicable
